# Resveratrol-Based MTDLs to Stimulate Defensive and Regenerative Pathways and Block Early Events in Neurodegenerative Cascades

**DOI:** 10.1021/acs.jmedchem.1c01883

**Published:** 2022-03-04

**Authors:** Clara Herrera-Arozamena, Martín Estrada-Valencia, Patricia López-Caballero, Concepción Pérez, José A. Morales-García, Ana Pérez-Castillo, Eric del Sastre, Cristina Fernández-Mendívil, Pablo Duarte, Patrycja Michalska, José Lombardía, Sergio Senar, Rafael León, Manuela G. López, María Isabel Rodríguez-Franco

**Affiliations:** † Instituto de Química Médica, Consejo Superior de Investigaciones Científicas (IQM-CSIC), C/ Juan de la Cierva 3, E-28006 Madrid, Spain; ‡ Programa de Doctorado en Química Orgánica, Facultad de Química, Universidad Complutense de Madrid, Avda. Complutense s/n, E-28040 Madrid, Spain; § Instituto de Investigaciones Biomédicas (CSIC-UAM), C/Arturo Duperier, 4, E-28029 Madrid, Spain; ∥ Centro de Investigación Biomédica en Red sobre Enfermedades Neurodegenerativas (CIBERNED), C/Valderrebollo 5, E-28031 Madrid, Spain; ⊥ Departamento de Biología Celular, Facultad de Medicina, Universidad Complutense de Madrid, Avda. Complutense s/n, E-28040 Madrid, Spain; # Instituto Teófilo Hernando de I+D del Medicamento, Departamento de Farmacología y Terapéutica, Facultad de Medicina, Universidad Autónoma de Madrid, C/Arzobispo Morcillo 4, E-28029 Madrid, Spain; ¶ DrTarget Machine Learning, C/Alejo Carpentier 13, E-28806 Alcalá de Henares, Madrid, Spain; ∇ Instituto de Investigación Sanitaria del Hospital Universitario de la Princesa (IIS-IP), C/Diego de León 62, E-28006 Madrid, Spain

## Abstract

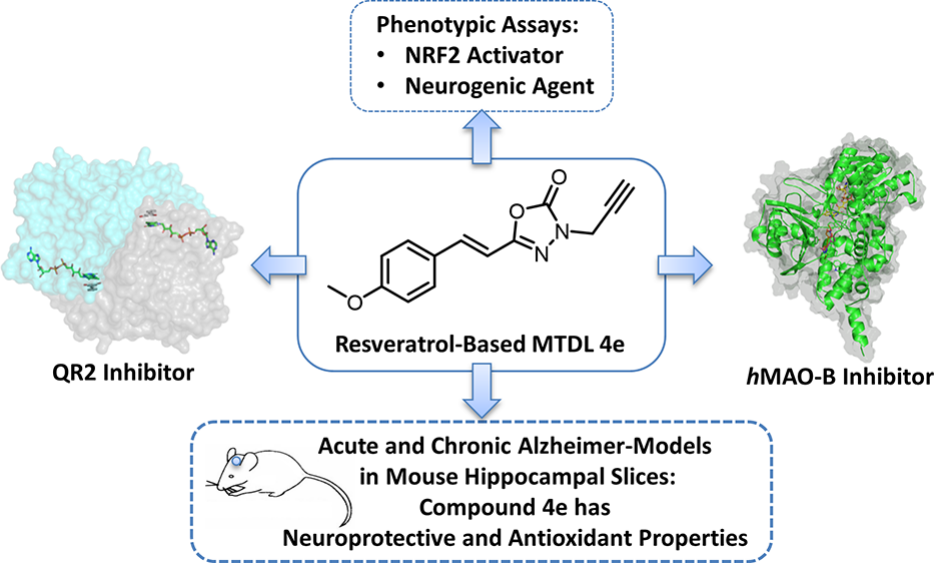

By replacing a phenolic ring of (*E*)-resveratrol with an 1,3,4-oxadiazol-2(3*H*)-one heterocycle, new resveratrol-based multitarget-directed ligands (MTDLs) were obtained. They were evaluated in several assays related to oxidative stress and inflammation (monoamine oxidases, nuclear erythroid 2-related factor, quinone reductase-2, and oxygen radical trapping) and then in experiments of increasing complexity (neurogenic properties and neuroprotection *vs* okadaic acid). 5-[(*E*)-2-(4-Methoxyphenyl)ethenyl]-3-(prop-2-yn-1-yl)-1,3,4-oxadiazol-2(3*H*)-one (**4e**) showed a well-balanced MTDL profile: cellular activation of the NRF2-ARE pathway (CD = 9.83 μM), selective inhibition of both hMAO-B and QR2 (IC_50_s = 8.05 and 0.57 μM), and the best ability to promote hippocampal neurogenesis. It showed a good drug-like profile (positive in vitro central nervous system permeability, good physiological solubility, no glutathione conjugation, and lack of PAINS or Lipinski alerts) and exerted neuroprotective and antioxidant actions in both acute and chronic Alzheimer models using hippocampal tissues. Thus, **4e** is an interesting MTDL that could stimulate defensive and regenerative pathways and block early events in neurodegenerative cascades.

## Introduction

In the context of greater longevity, neurodegenerative disorders (NDs) are the main cause of disability in the elderly, which adds an increasing pressure on health and social care systems. Alzheimer’s disease (AD) and Parkinson’s (PD) disease are the most frequent age-associated NDs, characterized by the progressive loss of intellectual and/or motor abilities, which result in patients being unable to carry out the most basic daily activities and finally speed up death. Both pathologies show a massive loss of different types of neuronal populations, cholinergic neurons in AD, and dopaminergic cells in PD, along with the accumulation of abnormally aggregated proteins, namely, amyloid β-peptide (Aβ) and hyperphosphorylated tau for AD^1^ and α-synuclein for PD.^2^

Given the complex and interconnected pathological cascades found in NDs,^3,4^ the current research focuses on the design of multitarget directed ligands (MTDLs) capable of acting on more than one biological target, hoping to obtain better therapeutic rewards than molecules acting by a single mechanism of action.^5^ In the design of these MTDLs, a holistic strategy should be followed based on systems pharmacology and on the known connections and interactions between biological targets.^6−8^ Ideally, MTDLs aimed at treating NDs should stimulate the body’s own regenerative and/or defensive pathways and also stop neurodegeneration by acting on the targets located upstream in neurotoxic cascades.^9^

For years, the existence of neural stem cells (NSCs) in the adult human brain has been known, which could generate and integrate new cells in the neuronal circuitry.^10^ Recent evidence that neuronal plasticity is abundant in the human hippocampus throughout the life of healthy subjects but decreases dramatically in those affected by neurological diseases paves the way for the development of neurogenic drugs for the treatment of NDs.^11^ Until now, many targets involved in neurogenesis have been identified in the central nervous system (CNS) and different regenerative candidates such as the activators of transcription factors (e.g., erythroid 2-related factor 2, NRF2, among others) and antioxidant and anti-inflammatory agents have been evaluated.^12,13^

Although NDs’ etiologies are not fully understood, recent findings have revealed that in the most affected nervous regions, there is a considerable increase in the peroxidation of biomolecules (lipids, proteins, and nucleic acids) and extensive neuroinflammation, hallmarks that precede the appearance of abnormal protein aggregates.^14^ Therefore, oxidative stress and chronic neuroinflammation are considered early events in these pathologies.^6^

As a consequence of the progressive failure of the antioxidant defensive systems with aging, oxidative stress dramatically increases. Uncontrolled free radical oxygen species (ROS) production induces the oxidation of biomolecules, leading to mitochondrial dysfunction, neuroimmune system activation, and protein misfolding and aggregation—processes whose combinations induce neuronal death. In AD, oxidative stress increases Aβ production and tau phosphorylation to generate aberrant protein aggregates.^15^ In turn, these aggregates induce more ROS, leading to mitochondrial dysfunction and generating an exacerbated oxidative stress status.^16^ PD patients show reduced mitochondrial complex I activity that has been related to ROS overproduction and higher neuronal susceptibility.^17^

Among the endogenous defensive systems, NRF2 (also known as NFE2L2) plays an essential role.^18,19^ When NRF2 is activated, it binds to antioxidant response elements (AREs) that lead to the expression of a plethora of genes involved in antioxidant and antiinflammatory responses. Subsequently, higher levels of defensive proteins, such as heme oxygenase 1 (HMOX-1), NAD(P)H quinone oxidoreductase 1, and glutathione *S*-transferase (GST), provide cellular protection in many pathological conditions.^20^ Indeed, several NRF2 inducers are already approved (dimethyl fumarate) or being tested in clinical trials (curcumin, oltipraz, omaveloxolone, resveratrol, sulforaphane, etc.) for the treatment of NDs.^21,22^

The scientific literature supporting NRF2 as a target for NDs is vast; thus, we have carried out a data mining study using the Open Targets database (DB)^23^ and two different sources: Expression Atlas (mRNA expression) and Europe PubMed Central (Europe PMC, literature). The literature results from the Open Targets DB are summarized in Figures S1 and S2 (Supporting Information 1), and full bibliographic data is compiled in Supporting Information 2. Expression Atlas returns a strong association between AD and NRF2 based on a microarray analysis of six brain areas from AD patients and normal individuals,^24^ published in two articles.^25,26^

Europe PMC affords 1577 co-occurrences in 737 articles with high scores and multiple examples of the high potential of NRF2-targeted therapies for NDs, including AD and PD. Many references describe how NRF2 activation ameliorates NDs’ symptoms by acting through the antioxidant–anti-inflammatory axis in a plethora of experimental models (for recent reviews, see refs (19), (27), and (28)). Specifically, recent studies have shown that activation of NRF2 counteracts toxicity of abnormal Aβ and tau proteins in AD^28^ and mitochondrial dysfunction in PD.^29^ Of great importance is the recent finding that NRF2 ablation in APP/PS1 transgenic mice promotes AD-like pathology.^30^ Similarly, in a mouse experimental model that combines amyloidopathy and tauopathy with either wild-type (AT-NRF2-WT) or NRF2-deficiency (AT-NRF2-KO), it was observed that AT-NRF2-KO brains presented increased markers of oxidative stress and neuroinflammation, as well as higher levels of toxic Aβ and tau proteins compared to the AT-NRF2-WT mice.^31^ Among the most recent and best-ranked articles by Open Targets, it has been reported that the intranasal application of the CNS-permeable polysaccharide mini-GAGR increases NRF2 nuclear translocation and subsequent transcriptional activity in 3xTg-AD mice. This resulted in the intensification of activities of NRF2-dependent antioxidant enzymes, an effect that was reverted by NRF2 knockdown by interference RNA. Moreover, 3xTg-AD mice exhibited significantly reduced levels of hyperphosphorylated tau and Aβ in hippocampal neurons, which enhanced memory and learning behaviors.^32^ Another study, performed in astrocytes differentiated from induced pluripotent stem cells derived from AD patients carrying the presenilin-1 PSEN1ΔE9 mutation, showed that lentiviral activation of the NRF2 pathway reduced amyloid secretion, normalized cytokine release, and increased glutathione (GSH) secretion.^33^ Additional Europe PMC-scored articles by Open Targets linking NFE2L2 to NDs can be accessed in Supporting Information 2.

Together with mitochondrial dysfunction, several enzymes have been described as essential contributors to increased oxidative stress at early stages of NDs’, including FAD-dependent quinone reductase-2 (QR2, also known as NQO2) and monoamine oxidases (MAO-A and MAO-B).

QR2 catalyzes the reduction of 1,2- and 1,4-quinones into unstable semiquinones, generating ROS.^34^ It is recognized as a melatonin binding site, and indeed, melatonin is a QR2 inhibitor, which would explain many of its protective properties against oxidative stress.^35^ Text mining on Europe PMC reveals the overexpression of NQO2 in AD patients,^36^ genetic polymorphisms associated with PD,^37^ an inhibitory role in memory formation and consolidation,^38^ and activity of NQO2 inhibitors in neuroprotective assays^39^ (see bibliographic data in Supporting Information 2). Recently, although not yet in the Open Targets DB, the neuroprotective role of NQO2 inhibitors has been documented by reducing ROS production and the percentage of apoptotic cells in HT-22 murine hippocampal neuronal cells.^40^ Although Open Targets NQO2 association scores for NDs are few and small compared to those for NFE2L2 or MAOB, the PhenoDigm algorithm, which prioritizes disease gene candidates based on phenotype information, assigns high scores for direct NQO2 association to early onset autosomal AD, and establishes strong indirect associations based on observed phenotypes stored in the Mouse Genome Informatics Database (Figure S3, Supporting Information 1). Specifically, the PhenoDigm DB retrieves a KO mouse model carrying an intragenic deletion of exons 2 and 3 of NQO2.^41^ Compared to wild-type animals, adult QR2 knock-out mice showed clear behavioral and neurological improvements in motor, spatial, and learning memory capacities, including Morris water maze, object recognition, and rotarod performance test. Furthermore, downregulation of the QR2 function by selective inhibitors has beneficial effects, both in *in vitro* and *in vivo* experiments. In cultured rat embryonic hippocampal neurons, the selective QR2 inhibitors S26695 and S29434 protected against menadione-induced cell death and S26695 also significantly inhibited scopolamine-induced amnesia in live mice.^42^ Overall association scores and evidence counts between NQO2 and NDs in Europe PMC and PhenoDigm databases are summarized in Figure S3 (Supporting Information 1). Interestingly, DisGeNet consultation shows similar records related to memory disorders while keeping low scores for direct associations with AD or PD. Data from any source (DisGeNet or Open Targets) show a wide overlap (Figure S4, Supporting Information 1).

Monoamine oxidases (MAO-A and MAO-B) catalyze the oxidative deamination of some neurotransmitters, triggering oxidative stress due to the generation of hydrogen peroxide and ROS. MAO-B mainly catalyzes the deamination of dopamine, which in addition to the increased levels of this enzyme found in the substantia nigra of PD patients has favored the development of its inhibitors as antiparkinsonian drugs, such as the selective MAO-B inhibitor rasagiline.^43^ Text mining in the Open Targets DB identifies associations between MAO-B and NDs from two data sources: Europe PMC (literature)^44^ and ChEMBL (clinical trials).^45^ In Europe PMC, there are 1156 co-occurrences from 706 articles. It is stated that increased MAO-B expression is an early event in AD and that there is a correlation between MAO-B levels and Aβ peptide pathology in AD.^46^ Several reports describe beneficial effects in AD murine models and patients treated with the MAO-B inhibitors sembragiline and selegiline.^47^ The number of evidence is extensive and of good quality overall. Literature results from Open Targets are summarized in Figures S5 and S6 (Supporting Information 1), and the comprehensive bibliographic information is gathered in Supporting Information 2. Open Targets ChEMBL reports high association scores for MAO-B association to PD, mainly supported by rasagiline formulations and other MAO-B inhibitors having completed phase IV, as selegiline and safinamide. A summary of MAO-B inhibitors in clinical trials is depicted in Figure S7 (Supporting Information 1).

**Figure 1:**
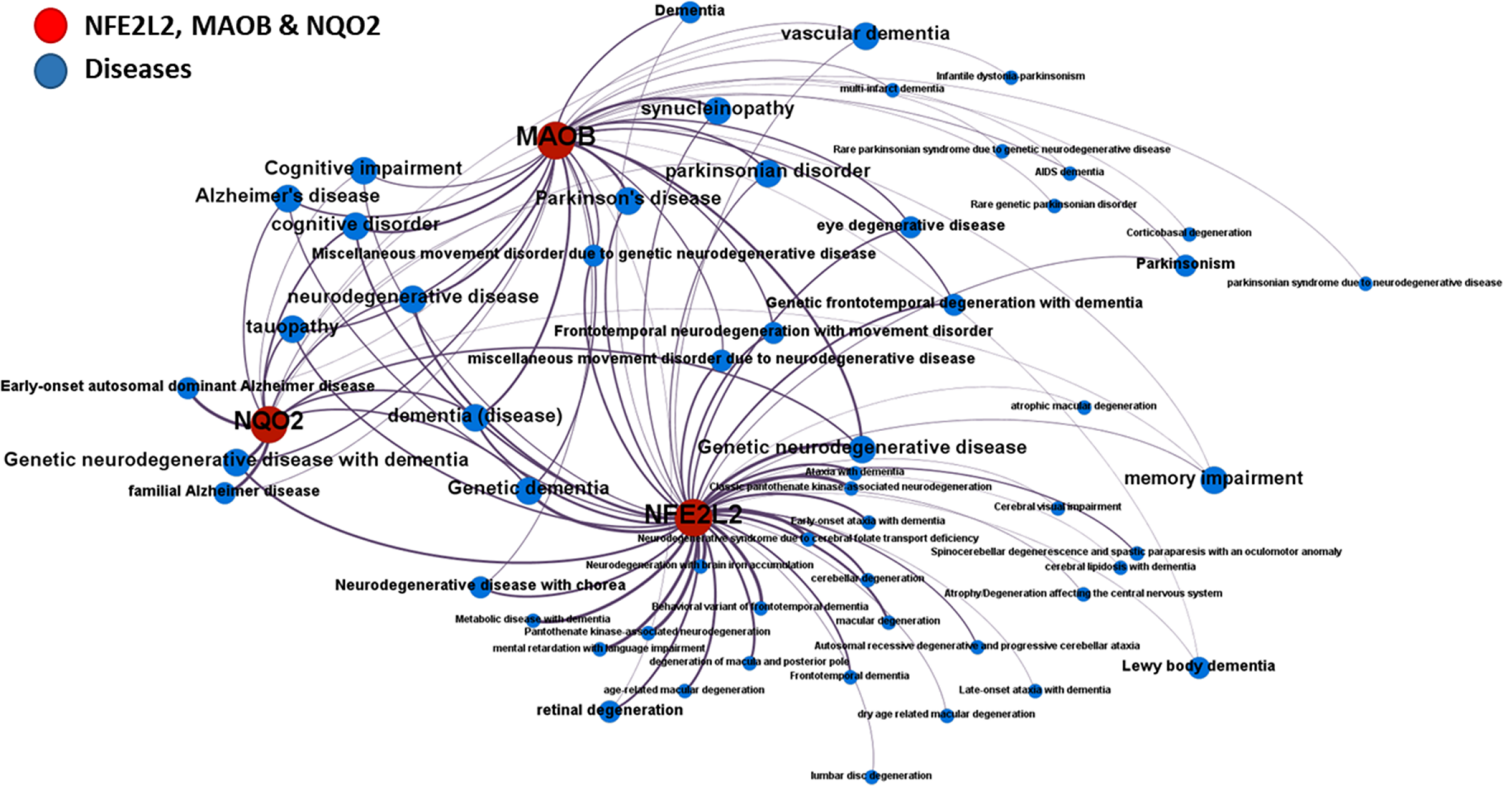
Target–disease associations for NFE2L2, NQO2, MAO-B, and NDs identified in the Open Targets DB. Node size is based on the centrality, i.e., the number of connected nodes. Red nodes represent genes, and blue ones correspond to diseases that are connected by edges. Boundary width represents the average Open Targets association score from direct and indirect pieces of evidence.

Using a systems biology approach, the relevance of NFE2L2, NQO2, and MAO-B as potential targets for NDs has been assessed by accessing the pertinent records in Open Targets and ChEMBL databases. The Open Targets DB reveals common association of NFE2L2, NQO2, and MAO-B with several NDs, displaying visible convergence for PD and AD phenotypes as tauopathies and synucleinopathies (Figure 1).

**Figure 2:**
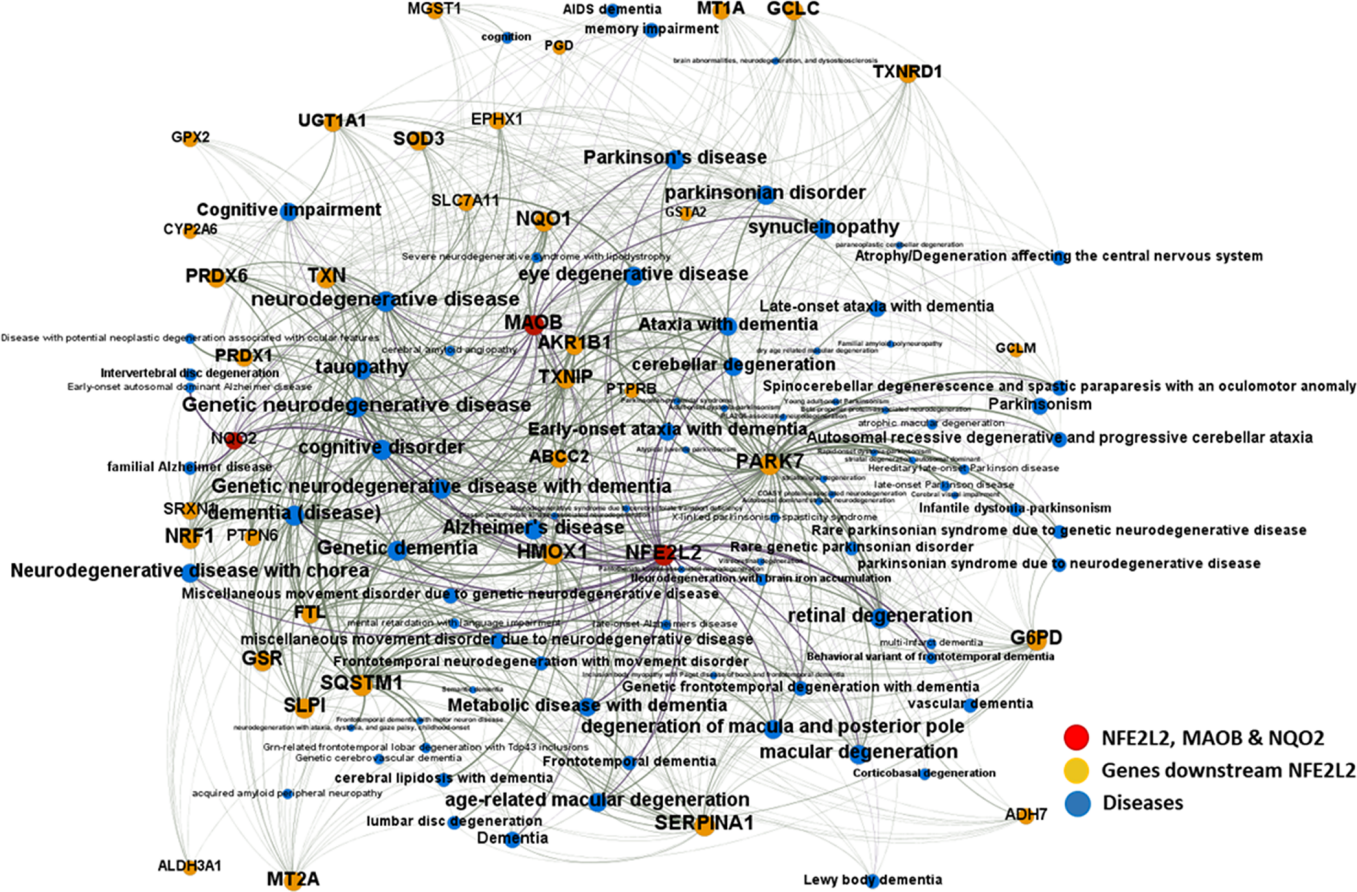
Target–disease associations for NFE2L2, NQO2, and MAO-B and its downstream AREs with NDs identified in the Open Targets DB. Node size is based on the centrality, i.e., the number of connected nodes. Gene and disease nodes are connected by edges. Edge width represents the averaged Open Targets association score from direct and indirect pieces of evidence. Node color represents gene class or disease, as described in the legend (target genes were taken from ref (48)).

To check possible synergies between the selected targets, we incorporated the downstream NFE2L2 genes into the target-disease interaction graph (Figure 2). All NFE2L2, NQO2, and MAO-B converge in AD and PD disorders, as well as associated tauopathy and synucleinopathy labels. Most of the gene downstream NFE2L2 activation showed associations with most of the same diseases. In particular, the defensive HMOX-1, the antioxidant proteins TXN (thioredoxin), TXNIP (thioredoxin interacting protein), and PARK7 (also known as protein deglycase DJ-1), the autophagy receptor SQSTM1 (sequestosome 1), and the proteinase inhibitor SERPINA1, showed the highest association scores for all NDs.

Resveratrol displays a plethora of biological activities: a potent NRF2-ARE pathway inducer,^49^ a QR2 inhibitor,^50^ and an efficient ROS scavenger,^51^ among others. As a result, this natural polyphenol exhibits an interesting pharmacological profile that includes neuroprotective and neurogenic effects.^52,53^ Resveratrol has a high oral absorption but a very low bioavailability because phenols are highly reactive points for metabolic transformations. Therefore, their partial or total replacement by other more stable groups could be a valid therapeutic approach.^54^

**Figure 3:**
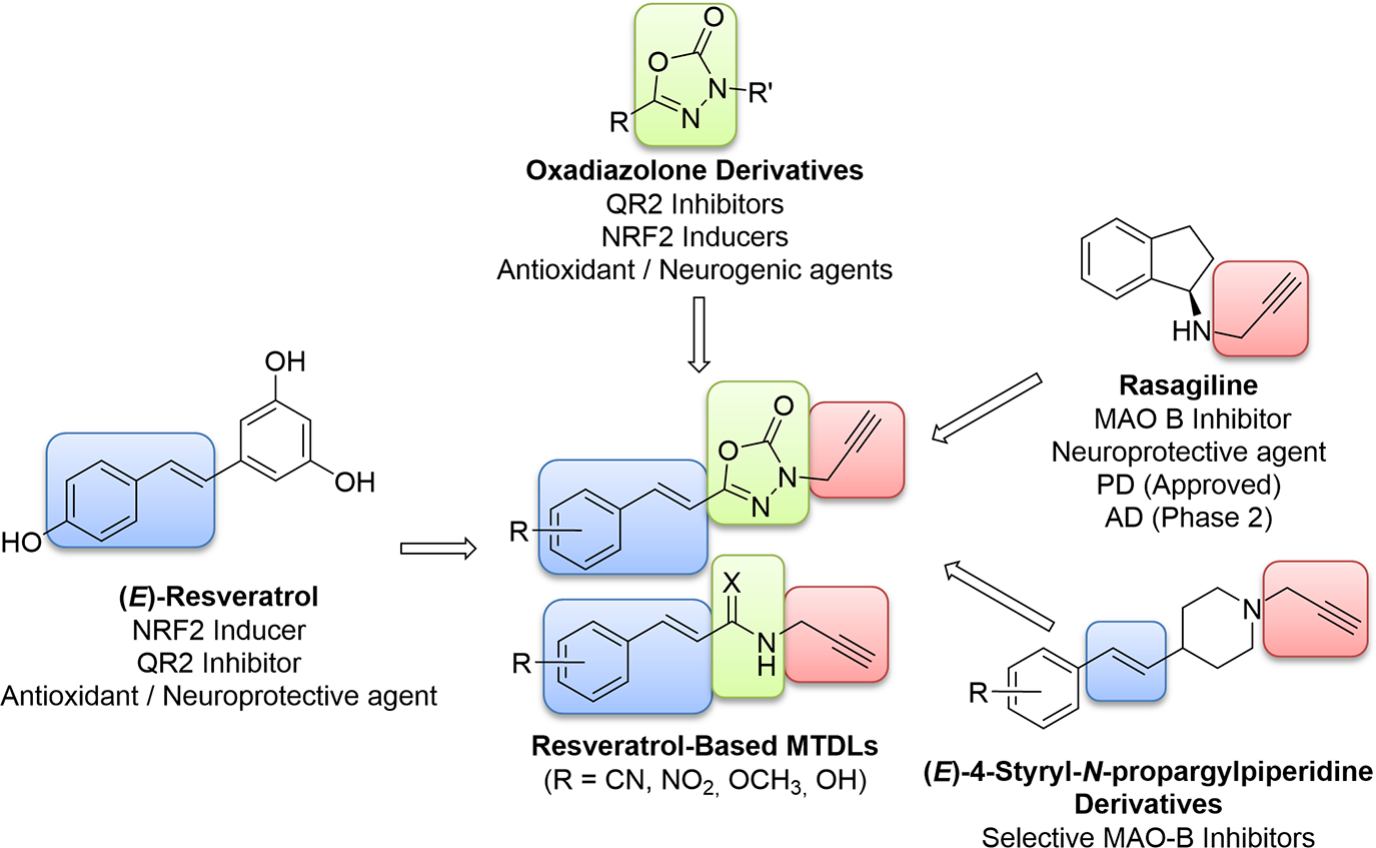
Design strategy for the new resveratrol-based MTDLs.

Based on these precedents, in this work, we developed new resveratrol-based MTDLs, which combine the activation of the NRF2 pathway with the inhibition of enzymes involved in NDs, such as QR2 and MAO-B. We planned to replace a phenolic ring from (*E*)-resveratrol with fragments less prone to metabolic degradation, such as a 1,3,4-oxadiazol-2(3*H*)-one heterocycle or a bioisosteric amide/amine group. Such fragments were successfully used in previous studies to produce NRF2 inducers and QR2 inhibitors with antioxidant and neurogenic properties.^55−57^ We considered using different substituents on the remaining phenyl ring, such as cyano, nitro, methoxy, or hydroxyl groups, to study their influence on the biological responses. We also envisaged to increase their potency and selectivity toward MAO-B by introducing a propargyl amine group, present in many MAO-B inhibitors such as rasagiline, currently approved for treating PD and in phase II clinical trials for AD.^58^ Selection of the trans-configuration for the linker has been reinforced by recent findings in an analogous family of 4-styryl-*N*-propargylpiperidine derivatives, in which the (*E*)-isomers were found to selectively target the MAO-B enzyme *versus* the MAO-A isoform^59^ (Figure 3).

**Figure 4:**
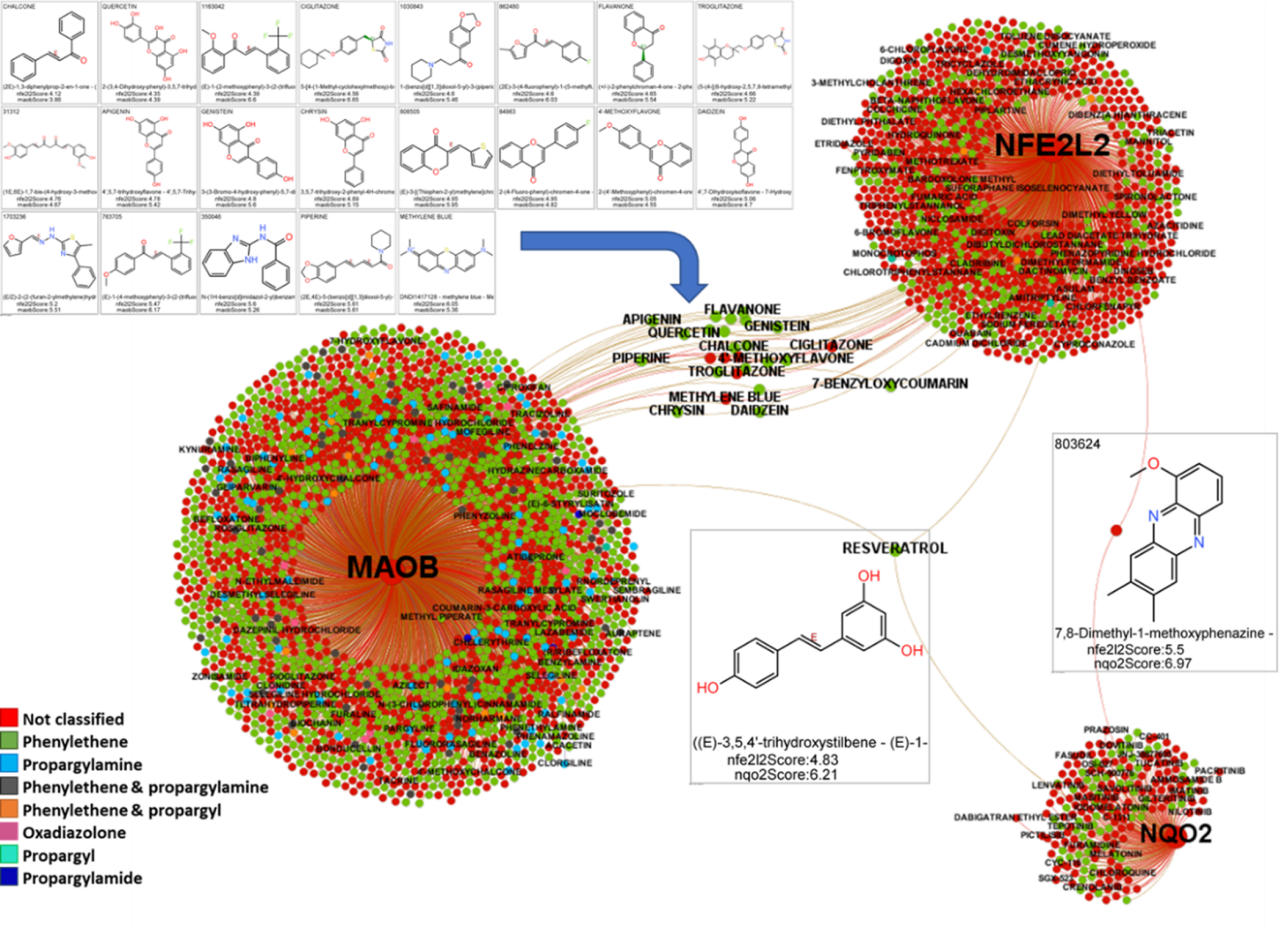
Compounds interacting with NQO2, NFE2L2, and MAO-B. Color by the substructure type. Graph only labels the compounds with a drug name. When ChEMBL only provides a registry number, the label has been suppressed to facilitate visualization.

To support our structural design, we have looked in the ChEMBL DB for the occurrence of substructures of our interest and the interactions with NFE2L2, NQO2, and MAO-B (Figure S8, Supporting Information 1). Only resveratrol resulted in activity in the three targets, a molecule (7,8-dimethyl-1-methoxyphenazine) was reported with activity for both NQO2 and NFE2L2, and 26 molecules resulted in activity for NFE2L2 and MAO-B (Figure 4). Interestingly, phenylethene was the most represented substructure within this group of molecules.

To check the 3D-structural similarity of our proposed molecules with resveratrol, a preliminary modeling study was performed. As shown in Figure S9 (Supporting Information 1), minimized structures of phenyl-*N*-propargyloxadiazolone and phenyl-*N*-propargylamide derivatives practically emulate the planarity of resveratrol, important for its interaction with QR2.^50^ Furthermore, resveratrol and phenyl-*N*-propargyloxadiazolone derivatives share a common pharmacophore consisting of two aromatic areas bearing donor/acceptor H-bonds linked by a hydrophobic double bond (Supporting Information 1, Figure S10).

## Results and Discussion

### Chemistry

**Scheme 1:**
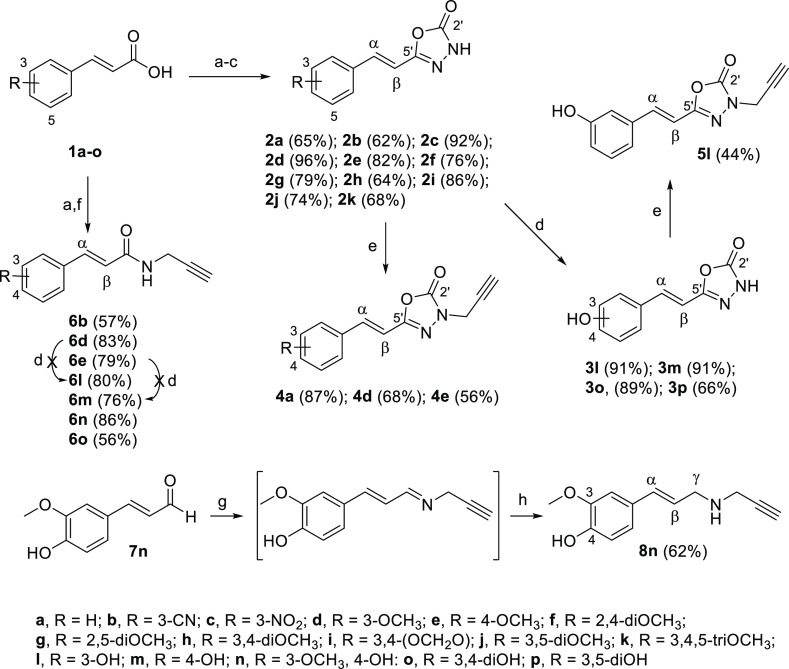
Reagents and Conditions (a) HOBt (1.2 equiv), EDC·HCl (1.2 equiv), and DMAP (0.12 equiv), ACN, N_2_, rt (30–180 min); (b) N_2_H_4_·H_2_O, rt (1–15 min); (c) CDI (1.2 equiv), DMF, MW, 120 °C, 25 min; (d) BBr_3_ (5–6 equiv), DCM, rt, overnight; (e) propargyl bromide (1.2 equiv), K_2_CO_3_, acetone, MW, 120 °C, 10 min or rt overnight; (f) propargylamine (1.2 equiv), ACN, N_2_, rt (15 min); (g) propargylamine (5 equiv), THF, rt, overnight; (h) NaBH_4_ (1.1 equiv), MeOH, rt, 30 min

The synthesis of resveratrol–oxadiazolone compounds was carried out from (*E*)-cinnamic acid derivatives, bearing diverse functional groups (nitrile, nitro, and methoxy) at different positions of cycle **1(a–k)** (Scheme 1). Many of these α,β-unsaturated acids were commercially available at common suppliers, whereas **1b** and **1i** were obtained in high yields (>90%) through a Knoevenagel–Doebner condensation using malonic acid and the corresponding aldehyde by overnight heating at 70 °C in pyridine and piperidine.

Acids **1(a–k)** were transformed into the corresponding hydrazides in quantitative yields by activation with 1-hydroxybenzotriazole (HOBt), 1-ethyl-3-(3-dimethylaminopropyl)carbodiimide hydrochloride (EDC·HCl), and 4-dimethylaminopyridine (DMAP), followed by reaction with hydrazine hydrate at rt. Without further purification, hydrazides were treated with 1,1′-carbonyldiimidazole (CDI) under microwave (MW) irradiation to give 1,3,4-oxadiazol-2(3*H*)-one heterocycles **2(a–k)** in moderate-to-good overall yields (62–96%) (Scheme 1).

Hydroxylated derivatives **3l**, **3m**, **3o**, and **3p** were obtained *via* deprotection of the corresponding methoxylated compounds by overnight treatment with boron tribromide (BBr_3_) in DCM at rt. To improve chemical yields, it was necessary to use one BBr_3_ equivalent for each ether group to be cleaved, and an additional equivalent for each heteroatom present in the molecule, due to the well-known complexation ability of the boron atom.^60^

Introduction of the propargyl group in derivatives **2** was achieved by alkylation of the NH group of the oxadiazolone ring with propargyl bromide in basic media (K_2_CO_3_) and acetone. In the case of the unsubstituted derivative (**2a**) and 3- and 4-methoxylated benzenes (**2d** and **2e**), reactions were performed in a MW reactor at 120 °C during 10 min, obtaining the desired *N*-propargyl derivatives **4a**, **4d**, and **4e**, in moderate-to-good yields (56–87%). However, alkylation of the 3-hydroxylated derivative **3l** was carried out overnight at room temperature (rt) in order to avoid possible secondary reactions through the phenolic group (experimental p*K*_a_ for *NH*-oxadiazolone, 7.04 ± 0.02; for phenol 8.72 ± 0.02, Supporting Information 1, Table S1 and Figure S11). Under these softer conditions, the *N*-propargyl-oxadiazolone derivative **5l** was isolated in 44% yield (Scheme 1).

Replacement of the oxadiazolone ring by an amide group was performed from differently substituted acids by condensation with propargylamine at rt in the presence of EDC·HCl, HOBt, and DMAP. In the case of 3-cyano, 3-, and 4-methoxy cinnamic acids, transformations were successfully accomplished in a MW reactor at 120 °C during 10 min, giving the corresponding propargyl amides **6b**, **6d**, and **6e** in reasonable yields (57–83%) (Scheme 1). The subsequent demethylation of the 3- and 4-methoxyphenyl propargyl amides **6d** and **6e** with BBr_3_ did not afford good yields of the desired phenolic products as only conversions around 20% were detected by HPLC–MS. Given that these low conversions could be due to unwanted reactions between BBr_3_ and the terminal alkyne, we tried the direct activation of the commercially available cinnamic acids bearing hydroxyl groups **1(l–o)** using habitual conditions (HOBt, EDC·HCl, and DMAP), followed by the treatment with propargylamine at rt overnight. Under these conditions, propargyl derivatives **6(l–o)** were obtained in acceptable yields (56–86%).

Reduction of amide **6n** to the corresponding amine was first attempted with lithium aluminum hydride (LiAlH_4_) with poor results as the desired product could hardly be detected in HPLC–MS. Thus, we used a reductive amination by the treatment of the commercially available ferulic aldehyde **7n** with propargylamine, giving an intermediate imine that was not isolated but reduced with sodium borohydride (NaBH_4_) to give the amine **8n** in a moderate yield (62%) (Scheme 1).

Resveratrol-based compounds were isolated and purified using flash column chromatography (IsoleraOne-Biotage system), and the purity (>95%) was analyzed by HPLC–MS. The chemical structures were characterized by spectroscopic data (^1^H NMR and ^13^C NMR), and complete NMR assignments were made by two-dimensional NMR experiments, mainly homonuclear correlation spectroscopy (COSY), heteronuclear single quantum correlation (HSQC) spectroscopy, and heteronuclear multiple bond correlation (HMBC) spectroscopy. All resveratrol-based MTDLs were obtained in their (*E*)-configuration, as deduced from the high coupling constant (*J* ∼ 16.5 Hz) between α and β protons observed in the ^1^H NMR spectra (see the Experimental Section part).

## Biological Evaluation

Resveratrol-based derivatives were first evaluated in a battery of biological assays related to oxidative stress and inflammation and then in cell- and tissue-based experiments of increasing complexity.

### Inhibition of Human Monoamine Oxidases

The hMAO-A/B inhibition of all compounds was determined using the Amplex Red MAO assay kit. (*E*)-Resveratrol, iproniazid, and moclobemide were also tested for comparative purposes (Table 1).

**Table 1:**
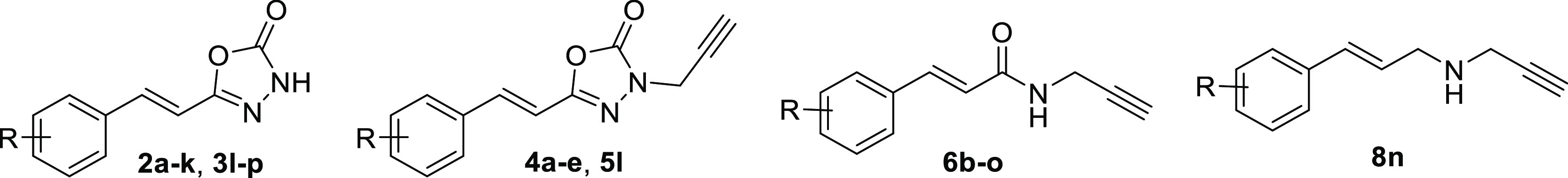
Basic Biological Evaluation of Resveratrol-Based MTDLs^a^

compd.	R	hMAO-A (IC_50_, μM)	hMAO-B (IC_50_, μM)	hMAOs SI	NRF2 (CD, μM)	ORAC (trolox equiv)	QR2 (IC_50_, μM)	PAMPA-BBB (*P*_e_, 10^–6^ cm s^–1^)
**2b**	CN	>50	>50 (38%)	n.a.	13.7 ± 2.2	<0.1	n.d.	4.5 ± 0.3 (cns+)
**2c**	NO_2_	>50	1.06 ± 0.22	>47.2	8.42 ± 0.82	<0.1	∼10 (56%)	4.7 ± 0.2 (cns+)
**2d**	3-OCH_3_	>50 (24%)	>50 (33%)	n.a.	>60	<0.1	∼10 (76%)	4.6 ± 0.2 (cns+)
**2e**	4-OCH_3_	>50	>50 (42%)	n.a.	>60	<0.1	∼10 (68%)	6.5 ± 0.5 (cns+)
**2f**	2,4-diOCH_3_	>50	>50	n.a.	n.d.	0.6 ± 0.08	n.d.	3.6 ± 0.2 (cns+/−)
**2g**	2,5-diOCH_3_	>50	>50	n.a.	n.d.	<0.1	n.d.	3.0 ± 0.2 (cns+/−)
**2h**	3,4-diOCH_3_	>50	>50	n.a.	>60	<0.1	n.d.	2.4 ± 0.3(cns+/−)
**2i**	3,4-(OCH_2_O)	>50	27.6 ± 0.9	>1.8	>60	<0.1	n.d.	3.8 ± 0.2 (cns+/−)
**2j**	3,5-diOCH_3_	>50	>50	n.a.	n.d.	<0.1	0.51 ± 0.05	2.1 ± 0.2 (cns+/−)
**2k**	3,4,5-triOCH_3_	>50	>50	n.a.	n.d.	<0.1	n.d.	≤0.5 (cns−)
**3l**	3-OH	>50	>50	n.a.	>60	3.8 ± 0.1	n.d.	≤0.5 (cns−)
**3m**	4-OH	41.5 ± 3.4	35.7 ± 3.0	1.2	22.7 ± 8.4	3.2 ± 0.1	n.d.	≤0.7 (cns−)
**3o**	3,4-diOH	3.4 ± 0.2	5.8 ± 0.4	0.6	21.3 ± 1.6	1.7 ± 0.1	n.d.	≤0.2 (cns−)
**3p**	3,5-diOH	>50	>50	n.a.	>60	1.9 ± 0.1	n.d.	≤0.1 (cns−)
**4a**	H	>50	9.87 ± 1.22	>5.1	16.9 ± 0.4	<0.1	>10 (44%)	13.6 ± 0.5 (cns+)
**4d**	3-OCH_3_	>50	0.64 ± 0.06	>78.1	7.44 ± 0.34	0.3 ± 0.06	0.40 ± 0.03	7.5 ± 0.3 (cns+)
**4e**	4-OCH_3_	>50	8.05 ± 1.82	>6.2	9.83 ± 0.6	<0.1	0.57 ± 0.04	8.0 ± 0.5 (cns+)
**5l**	3-OH	>50	3.53 ± 0.15	>14.2	8.05 ± 1.41	2.7 ± 0.2	2.50 ± 0.12	4.7 ± 0.2 (cns+)
**6b**	3-CN	>50	>50	n.a.	>60	<0.1	n.d.	n.d.
**6d**	3-OCH_3_	>50	>50	n.a.	n.d.	<0.1	∼10 (76%)	4.1 ± 0.2 (cns+)
**6e**	4-OCH_3_	>50	>50	n.a.	n.d.	<0.1	n.d.	n.d.
**6l**	3-OH	>50	>50	n.a.	n.d.	3.0 ± 0.3	n.d.	n.d.
**6m**	4-OH	>50	31.1 ± 1.57	>1.6	>60	3.0 ± 0.2	n.d.	n.d.
**6n**	3-OCH_3_, 4-OH	>50 (33%)	>50 (33%)	n.a.	>60	0.5 ± 0.03	n.d.	≤0.5 (cns−)
**6o**	3,4-diOH	47.0 ± 0.9	>50 (48%)	<0.9	19.2 ± 3.7	1.9 ± 0.1	n.d.	≤0.4 (cns−)
**8n**	3-OCH_3_, 4-OH	30.3 ± 2.5	>50 (45%)	<0.6	22.3 ± 2.9	2.1 ± 0.2	0.20 ± 0.04	8.7 ± 0.6 (cns+)
(*E*)-resveratrol		4.54 ± 0.37	29.9 ± 1.8	0.2	21.0 ± 2.0	4.0 ± 0.1	0.45 ± 0.03	n.d.
iproniazid		6.7 ± 0.8	7.5 ± 0.4	0.9	n.d.	n.d.	n.d.	n.d.
moclobemide		161.4 ± 19.4	>100	<1.6	n.d.	n.d.	n.d.	n.d.
sulforaphane		n.d.	n.d.	n.a.	1.24 ± 0.28	n.d.	n.d.	n.d.
melatonin		n.d.	n.d.	n.a.	n.d.	2.3 ± 0.1	0.077 ± 0.001	n.d.

a Inhibition (IC_50_, μM) or percentage of inhibition (in brackets) at the specified concentration of human monoamine oxidases (hMAO-A and hMAO-B) and quinone reductase 2 (QR2); NRF2 induction ability (CD, μM); ROS scavenger capability (ORAC, trolox equiv); and CNS-permeability prediction (PAMPA-BBB, *P*_e_, 10^–6^ cm s^–1^). Results are shown as the mean ± SEM of three independent experiments; results are shown as the mean ± SEM of four independent experiments in duplicate; results are shown as the mean ± SD of three independent experiments; SI: hMAO-B selectivity index = IC_50_(hMAO-A)/IC_50_(hMAO-B); n.a., not applicable; n.d., not determined.

(*E*)-Resveratrol revealed a slight selectivity toward hMAO-A, in accordance with previously described data.^61^ In contrast, most of our active resveratrol-based derivatives showed selective inhibition of hMAO-B in the micromolar and sub-micromolar range. This selectivity could be an advantage for our molecules because selective MAO-B inhibitors are preferred for the treatment of PD and AD.^62^

The nature, number, and position of the substituents had a marked influence on the results. In the series of N-unsubstituted oxadiazolones, methoxyphenyl derivatives did not display good inhibition of hMAOs, with the exception of the 3,4-dioxolanphenyl derivative **2i** that is a moderate MAO-B inhibitor (IC_50_ = 27.6 μM). Conversely, the 3-nitrophenyl-*NH*-oxadiazolone **2c** was a potent and selective MAO-B inhibitor with an IC_50_ in the low-micromolar range (IC_50_ = 1.06 μM).

The introduction of hydroxyl groups in the benzene ring generally improved the IC_50_s of *N*-unsubstituted oxadiazolones. The 4-hydroxyphenyl-*NH*-oxadiazolone **3m** displayed a moderate inhibition in both MAO isoforms, hMAO-A (IC_50_ = 41.5 μM) and hMAO-B (IC_50_ = 35.7 μM). Introduction of an additional phenolic group at the 3-position gave 3,4-dihydroxyphenyl-*NH*-oxadiazolone **3o**, which showed simultaneous inhibition of hMAO-A and hMAO-B, with IC_50_s in the low-micromolar range, namely, 3.4 and 5.8 μM, respectively.

The addition of a propargyl group in the oxadiazolone ring markedly improved the inhibition of hMAO-B, while the activity on hMAO-A was completely lost. Specifically, phenyl-, 4-methoxyphenyl-, and 5-hydroxyphenyl- *N*-propargyloxadiazolones **4a**, **4e**, and **5l** were potent and selective inhibitors of hMAO-B, with IC_50_ values in the low-micromolar range (IC_50_s = 9.87, 8.05, and 3.53 μM, respectively), reaching the sub-micromolar magnitude in the case of the 3-methoxyphenyl-*N*-propargyloxadiazolone **4d** (IC_50_ = 0.64 μM).

In the phenyl-propargylamide series, only compounds with a 4-phenolic group showed MAO inhibition. The 4-hydroxyphenyl-propargylamide **6m** is a modest hMAO-B inhibitor (IC_50_ = 31.1 μM), whereas adding an extra 3-methoxy group reversed this selectivity, giving amide **6o** and amine **8n**, which were moderate hMAO-A inhibitors (IC_50_ = 47.0 and 30.3 μM, respectively).

### Monoamine Oxidase B Binding Mode Elucidation: Reversibility Assays and Molecular Docking Studies

**Figure 5:**
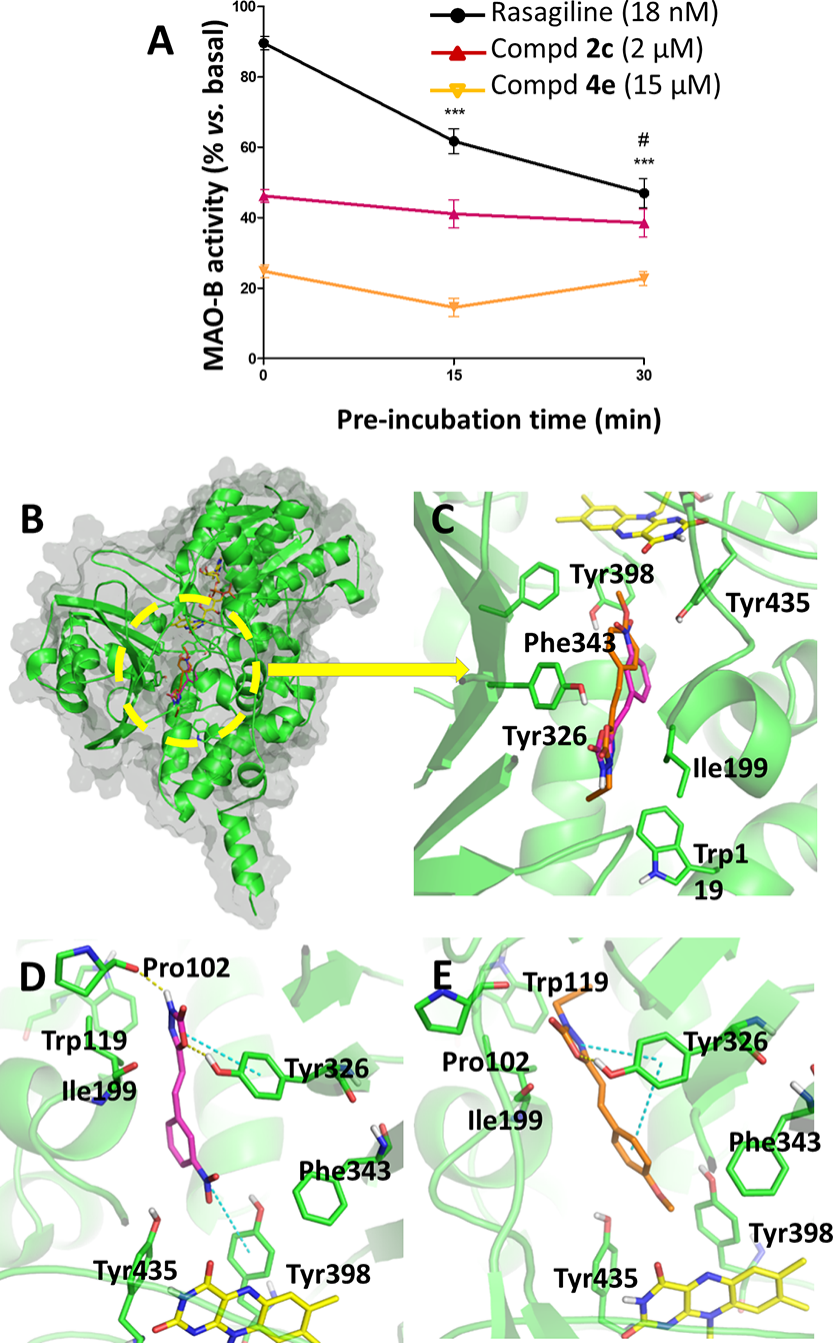
Reversibility assays and molecular docking studies in MAO-B of compounds **2c** and **4e**. (A) Reversibility assays. MAO-B activity variation along different preincubation times with compounds, showing irreversible inhibition of rasagiline and reversible inhibition of **2c** and **4e**. Data are the mean ± SEM of four independent experiments. Statistical analysis was performed following one-way ANOVA (*p* < 0.05). ***p < 0.001 *vs* 0 min preincubation time and #*p* < 0.05 *vs* 15 min preincubation time conditions after the Tukey posthoc test. (B) MAO-B structure (PDB-ID 6FW0)^65^ with **2c** and **4e** docked at the protein binding site. The MAO-B protein is represented as a green cartoon and gray surface, with key residues as green sticks; **2c** and **4e** are represented as purple and orange sticks, respectively. The FAD coenzyme is represented as yellow sticks. (C) Zoomed-in view of the docking results with predicted poses for compounds at the MAO-B bipartite cavity. (D,E) Detail of the proposed binding modes for **2c** and **4e**; π interactions and hydrogen bonds are shown as blue and yellow dotted lines, respectively.

Two potent and selective hMAO-B inhibitors, namely, the 3-nitrophenyl-*NH*-oxadiazolone derivative **2c** (IC_50_ = 1.06 μM) and the 4-methoxyphenyl-*N*-propargyl-oxadiazolone derivative **4e** (IC_50_ = 8.05 μM), were selected to evaluate their potential binding mode in this enzyme (Figure 5).

Given that long-term treatments with irreversible MAO-B inhibitors have shown disappointing results in patients with AD, reversible inhibitors are currently preferred.^63^ To study whether **2c** and **4e** act reversibly or irreversibly as MAO-B inhibitors, we measured MAO-B activity at different preincubation times of the tested compounds using the well-known irreversible inhibitor rasagiline as reference.^64^ Experimental results with rasagiline showed greater inhibition as the preincubation time increased, in contrast to the behavior observed with **2c** and **4e**, which kept the MAO-B activity almost constant (Figure 5A). Therefore, these experiments pointed out that derivatives **2c** and **4e** could act as reversible MAO-B inhibitors.

To suggest a binding mode of **2c** and **4e** at the hMAO-B active site, we performed molecular docking studies using its crystal structure in complex with the reversible inhibitor *N*-(3-chlorophenyl)-4-oxo-4*H*-chromene-3-carboxamide (PDB-ID 6FW0).^65^ Validation of the study was performed by comparison between the docking results and crystallographic data of the described inhibitor, obtaining similar poses with little atomic deviations between both structures (Supporting Information-1, Figure S12A).

Compounds **2c** and **4e** showed similar binding modes occupying both entrance and substrate cavities (Figure 5B,C), with a comparable docking score (∼−5.8 kcal/mol). This common binding mode is comparable to that of the chromone-based reversible MAO-B inhibitor (Supporting Information-1, Figure S12B). Our derivatives were observed to interact with the key residues of the protein, establishing π–π stacking interactions with Tyr326 (Figure 5D,E). Tyr326 is considered a “gating residue” together with Ile199 as their side chains mark the separation between the entrance and substrate cavities of the MAO-B active site and they have been described as key residues for substrate and inhibitor recognition.^66^

In addition to Tyr326 interaction, compound **2c** showed a hydrogen bond with Pro102 and a cation−π interaction with Tyr398 (Figure 5D). As this aromatic residue is described to be involved in catalysis and substrate specificity,^67^ the presence of the nitro group in this area could be a determining factor in the inhibition potency of **2c**.

Derivative **4e** placed its alkyne moiety at the cavity entrance widely separated from the FAD coenzyme, a result that is in agreement with its reversible mechanism of action. In addition, the alkyne moiety could interact with the aromatic side chain of Trp119 stabilizing this position (Figure 5E).

### Induction of the NRF2-ARE Signaling Pathway

Compounds that had previously shown hMAO activity were then tested as NRF2-ARE signaling pathway inducers in the AREc32 cell line, and data were expressed as the concentration needed to duplicate the specific activity of the luciferase reporter (CD).^68^ (*E*)-Resveratrol and sulforaphane were used as the reference and positive control, respectively (Table 1).

Like (*E*)-resveratrol (CD = 21.0 μM) and sulforaphane (CD = 1.24 μM), most of the tested compounds showed NRF2 activation with CD values in the micromolar range (CD = 7.44–22.7 μM). Considering phenyl-*NH*-oxadiazolone derivatives, the most active NRF2 activators were the 3-nitrophenyl **2c** (CD = 8.42 μM), 3-cyanophenyl **2b** (CD = 13.7 μM), 3,4-dihydroxyphenyl **3o** (CD = 21.3 μM), and 4-hydroxyphenyl **3m** (CD = 22.6 μM) derivatives. Introduction of a propargyl fragment in the NH of the oxadiazolone heterocycle produced an increase in the activity, obtaining values in the low micromolar range for the 3-methoxyphenyl **4d** (CD = 7.44 μM), 3-hydroxyphenyl **5l** (CD = 8.05 μM), 4-methoxyphenyl **4e** (CD = 9.83 μM), and phenyl **4a** (CD = 16.9 μM) derivatives. Among the phenyl–propargyl amides, catechol **6o** was the only active compound (CD = 19.2 μM). Amine **8n** displayed a moderate activity (CD = 22.3 μM), better than its amide analogue **6n** (CD > 60 μM).

From these results, we can deduce that the presence of the *N*-propargyl-oxadiazolone fragment is favorable for the activation of the NRF2-ARE signaling pathway.

### Evaluation of the Oxygen Radical Absorbance Capacity

Considering the scavenger capacity of resveratrol and the structural similarities of our derivatives, we advanced the potential antioxidant capacity of these compounds. Thus, the oxygen radical absorbance capacity (ORAC) values of new compounds were determined as a measure of their antioxidant properties, following described protocols.^55,69^ Trolox[(±)-6-hydroxy-2,5,7,8-tetramethylchromane-2-carboxylic acid], the aromatic part of vitamin E responsible for its scavenging properties, was used as the internal standard with the arbitrary value of ORAC = 1.0. The results are expressed as trolox equiv in a comparative scale that shows if a compound is a better (ORAC > 1.0) or a worse ROS scavenger (ORAC < 1.0) than vitamin E. (*E*)-Resveratrol and melatonin were used as standards, showing ORAC values 4.0- and 2.3-fold higher than trolox, respectively (Table 1).

As expected, only compounds with phenolic groups exhibited remarkable antioxidant capacity (ORAC = 1.7–3.8 trolox equiv). The best results were obtained in resveratrol-based derivatives bearing only one hydroxyl group in position 3- or 4- of the benzene ring, namely, **3l**, **3m**, **5l**, **6l**, and **6m** (ORAC = 2.7–3.8 trolox equiv). Surprisingly, these values were reduced with the introduction of a second phenolic group as it was found for derivatives **3o**, **3p**, and **6o** (ORAC = 1.7–1.9 trolox equiv).

### Evaluation in Melatonin Receptors: MT_1_R, MT_2_R, and QR2

As explained previously, QR2 is a major contributor to exacerbated oxidative stress in NDs^39^ and a target for melatonin.^35^ Bearing in mind the possible similarities of the QR2 binding site with G protein-coupled melatonin receptors, we envisaged to probe the activity of our derivatives, not only in QR2 but also in MT_1_R and MT_2_R. (*E*)-Resveratrol and melatonin were also tested for comparison.

In human melatonin receptors (*h*MT_1_R and *h*MT_2_R), only the 3-methoxyphenyl-propargylamide **6d** showed a moderate binding affinity toward *h*MT_2_R (*K*_i_ = 1.4 μM), while the rest of the compounds did not show substantial activity at the maximum tested concentration (10 μM).

In the case of hamster QR2, several compounds showed sub-micromolar IC_50_s, close to that of (*E*)-resveratrol (IC_50_ = 0.45 μM) (Table 1). In general, N-unsubstituted oxadiazolone derivatives showed poor activity at 10 μM, with the exception of the 3,5-dimethoxyphenyl-*NH*-oxadiazolone **2j** (IC_50_ = 0.51 μM). In contrast, the presence of propargyl and methoxyphenyl fragments appears to favor the QR2 inhibition; the 3- and 4-methoxyphenyl *N*-propargyloxadiazolones (**4d** and **4e**) and the 3-methoxy-4-hydroxyphenyl propargylamine **8n** demonstrated to be potent and selective ligands of QR2 with IC_50_s in the sub-micromolar range (IC_50_s = 0.40, 0.57, and 0.20 μM, respectively). Unlike these, the 3-hydroxyphenyl-*N*-propargyloxadiazolone **5l** is a micromolar QR2 inhibitor (IC_50_ = 2.50 μM), slightly worse than its 3-methoxyphenyl counterpart **4d** (IC_50_ = 0.40 μM) (Table 1 and Figure S13 in Supporting Information-1).

### *In Vitro* Blood–Brain Barrier Permeation Assay (PAMPA-BBB)

Moreover, the capability of the new compounds to cross the blood–brain barrier (BBB) was evaluated by the in vitro parallel artificial membrane permeability assay for the BBB (PAMPA-BBB) described by Di et al.^70^ and modified by our group for testing molecules with partial water solubility.^55,71−73^ Commercial standards with known BBB permeability were included in each experiment for validation and comparison (Supporting Information-1, Table S2). As previously established in the literature, compounds with *P*_e_ > 4.0 × 10^–6^ cm·s^–1^ would cross the BBB (cns+) whereas those displaying *P*_e_ < 2.0 × 10^–6^ cm·s^–1^ would not reach the CNS (cns−). Between these values, the predicted CNS permeability was uncertain (cns +/−).^70^

In the phenyl-*NH*-oxadiazolone series, compounds bearing a cyano-, nitro-, or methoxy-group (**2b–e**) were found to be CNS-permeable (*P*_e_ = 4.5, 4.7, 4.6, and 6.5 10^–6^ cm s^–1^, respectively), whereas the presence of two or more methoxy fragments gave compounds with an uncertain CNS permeation (cns+/−); hydroxyl derivatives were predicted not to cross the BBB (cns−). However, all derivatives bearing a propargyl group in the *NH*-oxadiazolone ring are predicted to enter into the CNS by passive permeation, even bearing a hydroxyl group such as in the case of **5l** (*P*_e_ = 4.7 10^–6^ cm s^–1^). Regarding propargyl-amide or -amine derivatives, only those with methoxy groups were predicted to cross the BBB; 3-methoxy-4-hydroxyphenyl propargylamine **8n** showed a better *P*_e_ value (8.7 10^–6^ cm s^–1^) than its amide analogue **6n** (*P*_e_ < 2.0 10^–6^ cm s^–1^), pointing out that the presence of a carbonyl group decreased the CNS permeability.

### Molecular Docking Studies in QR2

**Figure 6:**
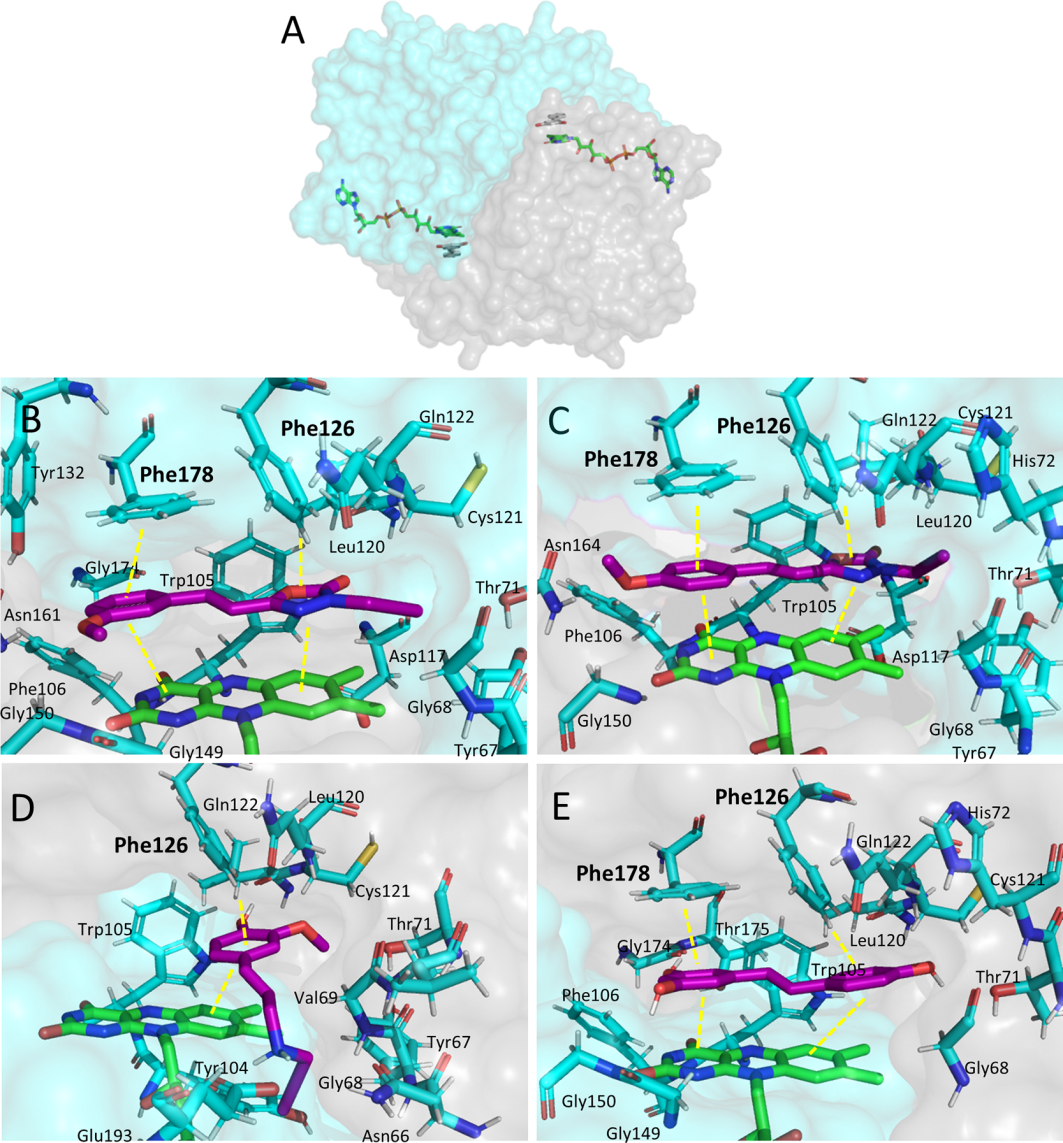
(A) Crystal structure of FAD-QR2 in complex with resveratrol (PDB-ID 4QOH).^50^ (B–E) Molecular docking of compounds **4d**, **4e**, **8n**, and resveratrol in the active site of QR2. FAD is shown in green sticks, inhibitors in purple sticks, and amino acids less than 4 Å away from the compounds in cyan sticks. Yellow dotted lines show polar interactions between the compounds, cofactor, and amino acids. Amino acids that could interact with the compounds are highlighted in bold.

Sub-micromolar QR2 inhibitors **4d**, **4e**, and **8n** with positive CNS penetration were subjected to molecular modeling studies to propose their binding modes, using the crystal structure of FAD-QR2 in complex with resveratrol (PDB-ID 4QOH).^50^ QR2 is a cytoplasmic homodimer protein with two active sites, in which FAD is a required cofactor to develop its physiological function (Figure 6A). The sequence of both monomer chains is equal, but the amino acids that participate in the catalytic sites are different depending on the position they adopt. Since the QR2–resveratrol complex has two different crystallization states, docking studies have been performed in quadruplicate considering each binding site and two different conformations on FAD-QR2.

Compounds **4d**, **4e**, and **8n** interact with the cofactor FAD and the aromatic amino acids Phe178 and Phe126 of the catalytic site, similarly to resveratrol (Figure 6B–E, respectively). Compounds **4d** and **4e**, with a 3-methoxyphenyl- and 4-methoxyphenyl- moiety, respectively, adopted similar poses and intermolecular interactions; π–π stacking with Phe178, π-dipole with Phe126, and dipole-induced dipole interaction with FAD rings. Our results show that the FAD cofactor interacts by dipole-induced dipole interaction with the oxadiazolone ring of **4d** and **4e** and by π–π stacking with the benzenes of compound **8n** and resveratrol. These intermolecular forces increase the stability of the protein–compound complex and should be considered in drug design directed to inhibit QR2.

### Neuroprotection and Neurogenic Studies in Cell Cultures

Compounds showing the MAO and/or QR2 inhibition and NRF2 activation profile were selected to study their neuroprotective and neurogenic abilities using different cell models.

### Effect of Resveratrol-Based MTDLs on Cell Viability in the Human Neuroblastoma SH-SY5Y Cell Line

Before evaluating if the selected resveratrol-based MTDLs were neuroprotective and/or neurogenic agents, a study of cell viability was carried out by incubating the compounds alone in the human neuroblastoma cell line SH-SY5Y during 24 h at 100 μM. In these experiments, resveratrol was included for comparative purposes. Cell viability was determined by the (3-(4,5-dimethylthiazol-2-yl)-2,5-diphenyltetrazolium bromide (MTT) assay, normalizing data to the basal condition (100% viability). None of the tested compounds was toxic at 100 μM except compound **4a**, which reduced the viability by around 30%. Interestingly, resveratrol reduced over 50% cell viability per se (Supporting Information-1, Figure S14); this result indicates that our resveratrol-based MTDLs are potentially safer than their reference compound in terms of neurotoxicity.

### Neuroprotection against OA-Induced Toxicity in Rat Primary Cortical Neuronal Cultures

Once neurotoxicity per se was discarded, the neuroprotective ability of the compounds was evaluated in rat primary cortical neuronal cell cultures exposed to okadaic acid (OA). This toxin is a potent inhibitor of protein phosphatases that induces tau hyperphosphorylation, one of the major histopathological hallmarks of AD,^74^ and is widely used in vitro or in vivo as an AD-related model.^75^

**Figure 7:**
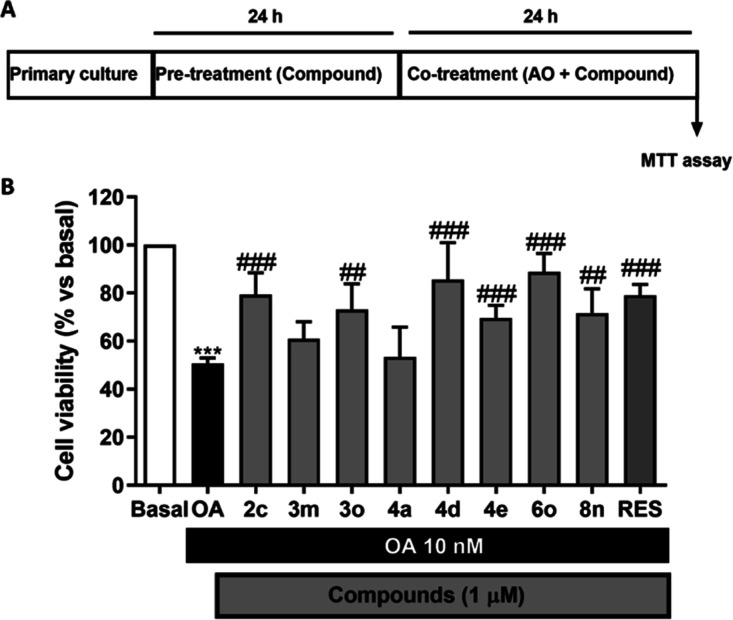
Neuroprotection assay against OA-induced toxicity in rat primary neuronal cultures. (A) Experimental protocol used. (B) Neuroprotective effect of the resveratrol-based MTDLs at 1 μM against OA-induced toxicity. The cell viability was determined by the MTT assay. RES = resveratrol. Data is represented as the percentage of cell viability normalized to the basal condition (100%). Bars show mean ± SEM. *N* = 5 for each experiment that was performed in triplicate. Statistical analysis was performed following one-way ANOVA (*p* < 0.05). ***p < 0.001 *vs* basal condition, #*p* < 0.05, ##*p* < 0.01, and ###*p* < 0.001 *vs* OA after the Tukey posthoc test.

Cortical neurons were pretreated with the resveratrol-based MTDLs at a concentration of 1 μM, and then, the cells were coincubated for another 24 h with OA and the compound (Figure 7A). While OA (10 nM) reduced the cell viability by 50%, almost all resveratrol-based MTDLs significantly increased the cell viability to 70–89%, except compounds **4a** and **3m** (Figure 7B). The most neuroprotective compounds **4d** and **6o** showed a slightly higher neuroprotection profile than resveratrol.

Regarding their potential mechanism of action, a direct correlation was not observed between the different biological activities evaluated and the neuroprotective capacity, which indicates that the neuroprotection exerted by these MTDL compounds is more related to a combination of mechanisms of action than to the interaction with a single biological target. In this line, the best neuroprotective compound **6o** was not the most potent in any of the tested targets as it is a poor MAO-B inhibitor (48% inhibition at 50 μM), a moderate NRF2 inducer (CD = 19.2 ± 3.7 μM), and a good but not the best ROS scavenger (ORAC = 1.9 ± 0.1 trolox equiv). Remarkably, compound **4d** showed the second highest neuroprotective capacity (neuronal viability increased to 86% at 1 μM), being the most potent MAO-B inhibitor (IC_50_ = 0.64 ± 0.06 μM), the best NRF2 inducer (CD = 7.44 ± 0.34 μM), and the second most potent QR2 inhibitor (IC_50_ = 0.40 ± 0.03 μM); however, compound **4d** was one of the poorest ROS scavengers showing an ORAC value of 0.3 ± 0.06 trolox equiv. In contrast, compounds **3m** and **4a** that were not able to significantly reduce neuronal death, showed moderate MAO-B inhibition, moderate NRF2 induction, and poor QR2 inhibition; moreover, compound **3m** showed the best ROS scavenger effect (ORAC = 3.2 ± 0.1 trolox equiv). These results further confirm our hypothesis toward a mixed neuroprotection mechanism of action since a potent action toward one of the evaluated targets does not correlate with a higher neuroprotective capacity.

### Drug-like Properties

Next, we evaluated the potential toxicological alerts and physicochemical properties of resveratrol-based MTDLs using the KNIME software.^76^ Derivatives were filtered according to the pan-assay interference rules (PAINS) and Lipinski guideline (Supporting Information-1, Figure S15). The evaluated derivatives did not show any toxicological alert, except compounds **3o** and **6o** due to the presence of a catechol fragment in their structures.^77^ The calculated physicochemical properties according to the KNIME software are gathered in Table S3 (Supporting Information-1).

### Neurogenic Studies

Compounds that have shown simultaneous cellular activation of NRF2, selective inhibition of both hMAO-B and QR2, neuroprotective properties against OA-induced toxicity in primary neuronal cultures, and a favorable drug-like profile were prioritized to study their neurogenic properties in the primary cultures of NSCs. These derivatives were the *N*-propargyloxadiazolones **4d** (3-methoxyphenyl) and **4e** (4-methoxyphenyl). Other structure-related counterparts were also tested for comparative purposes, namely, the *NH*-oxadiazolones **2c** (3-nitrophenyl), **2d** (3-methoxyphenyl), and **3l** (3-hydroxyphenyl), as well as the phenyl-*N*-propargyloxadiazolone **4a**.

**Figure 8:**
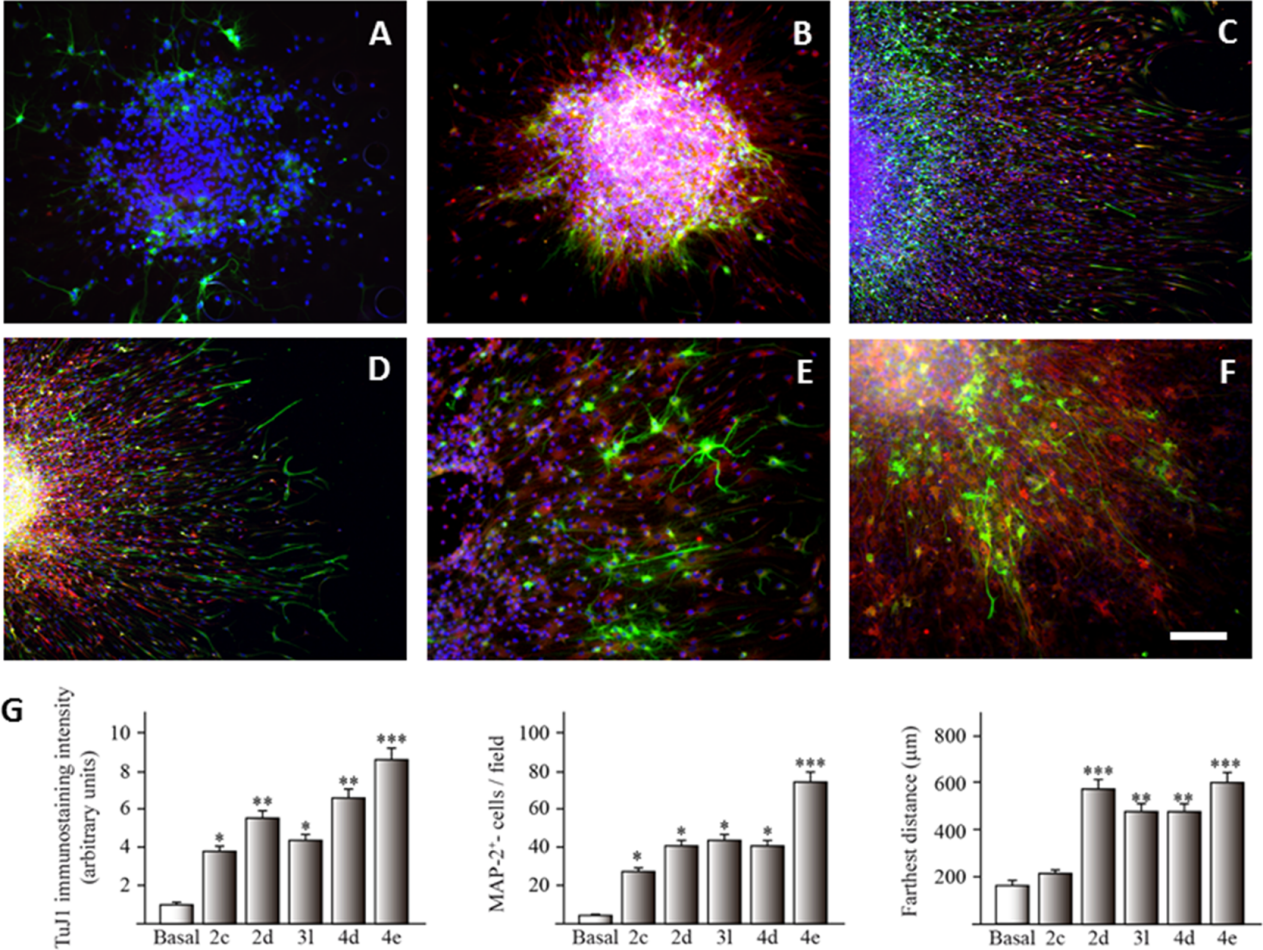
Confocal images showing the expression of neuronal markers in cultured SGZ-derived NS under (A) basal conditions and in the presence of different resveratrol-based compounds at 10 μM: (B) **2c**, (C) **2d**, (D) **3l**, (E) **4d**, and (F) **4e**. TuJ-1 (immature neurons) is shown in green and MAP-2 (mature neurons) in red. DAPI (blue) was used as a nuclear marker (images of inactive compounds are not shown). Calibration bar, 100 μm. (G) Quantification of TuJ-1- and MAP-2-expressing cells, and the farthest distance of cell migration is shown. Statistical differences are represented as *P ≤ 0.05, **P ≤ 0.01, ***P ≤ 0.001 in comparison to the basal condition.

Adult mice NSCs were isolated from SGZ of the dentate gyrus of the hippocampus and grown as free-floating neurospheres (NS).^72,78^ The neurogenic potential of each compound was determined using fluorescence confocal microscopy by quantifying the expression of two well-known neuronal markers: human β-III-tubulin (TuJ-1 clone) and microtubule-associated protein 2 (MAP-2). TuJ-1 is expressed in immature neurons, whereas the expression of MAP-2 indicates a consolidated mature neuronal stage.^79^ Given that neurogenesis involves not only the proliferation of NSC but also the migration of newly generated cells, the greatest migration distance promoted by each compound was also measured (Figure 8).

The control experiments (vehicle-treated cultures) showed a few positive cells for TuJ-1 or MAP-2 and scarce cell migration. However, in cultures treated with **2c**, **2d**, **3l**, **4d**, and **4e**, the number of both TuJ-1 and MAP-2 marked cells clearly increased and the cell migration distance was also extended (Figure 8). In contrast, **4a** showed poor capacity to differentiate NSCs (data not shown).

The best results were obtained with the 4-methoxyphenyl-*N*-propargyloxadiazolone **4e**, which showed the highest expression of young cells (TuJ-1) and mature neurons (MAP-2), as well as the greatest distance of cell migration. All these events indicate that this compound has a great neurogenic potential due to its ability to promote different aspects involved in neurogenesis.

### Selection of a Candidate for Tissue AD Models

The analysis of all available data prompted us to select the 4-methoxyphenyl-*N*-propargyloxadiazolone derivative **4e** to take it forward in more complex AD models. In primary cell cultures, **4e** showed good neuroprotective properties against OA-induced toxicity and the best ability to stimulate different aspects related to the neurogenic process. Moreover, **4e** displayed simultaneous cellular activation of NRF2 (CD = 9.83 μM) and inhibition of both hMAO-B (IC_50_ = 8.05 μM) and QR2 (IC_50_ = 0.57 μM), lack of cellular toxicity, favorable drug-like properties, and good CNS permeability (*P*_e_ = 8.0 ± 0.5 10^–6^ cm s^–1^).

### Aqueous Solubility and Potential GSH Conjugation of **4e**

Before proceeding with the evaluation of compound **4e** in more complex AD models, we studied other characteristics relevant to drug discovery, such as aqueous solubility and potential binding to GSH. Following a described method,^80^ the thermodynamic solubility of **4e** in a physiologic-like medium (phosphate buffer pH 7.4) was found to be 76.1 ± 0.1 μM (19.5 ± 0.1 mg L^–1^), higher than the concentrations already used in neuroprotective and neurogenic assays and those expected to use in more complex experiments.

To examine if **4e** could act as an electrophilic compound with indiscriminate activity, we studied the reaction of this compound with GSH in the presence of glutathione-*S*-transferase (GST).^81^ Compound **4e** was incubated at 37 °C with GSH in the presence of the enzyme GST in phosphate-buffered saline (PBS). As the control, nonenzymatic reaction (mixture lacking GST) was also performed. When the reactions were analyzed by MS, no peaks corresponding to GS-**4e** adducts were found, suggesting that the resveratrol-based MTDL **4e** did not act as an electrophilic compound under these conditions (Supporting Information-1, Figure S16).

### Neuroprotection Studies of the Resveratrol-Based MTDL **4e** in Tissue AD Models

#### Resveratrol-Based MTDL **4e** Reduced OA Toxicity and OA-Related Oxidative Stress in an Acute-Tissue *In Vitro* Model of AD

Further on, we studied the neuroprotective and antioxidant properties of compound **4e** in mouse hippocampal slices against OA-induced toxicity. This is a more complex in vitro AD model used to induce tau pathology in the hippocampus.

**Figure 9:**
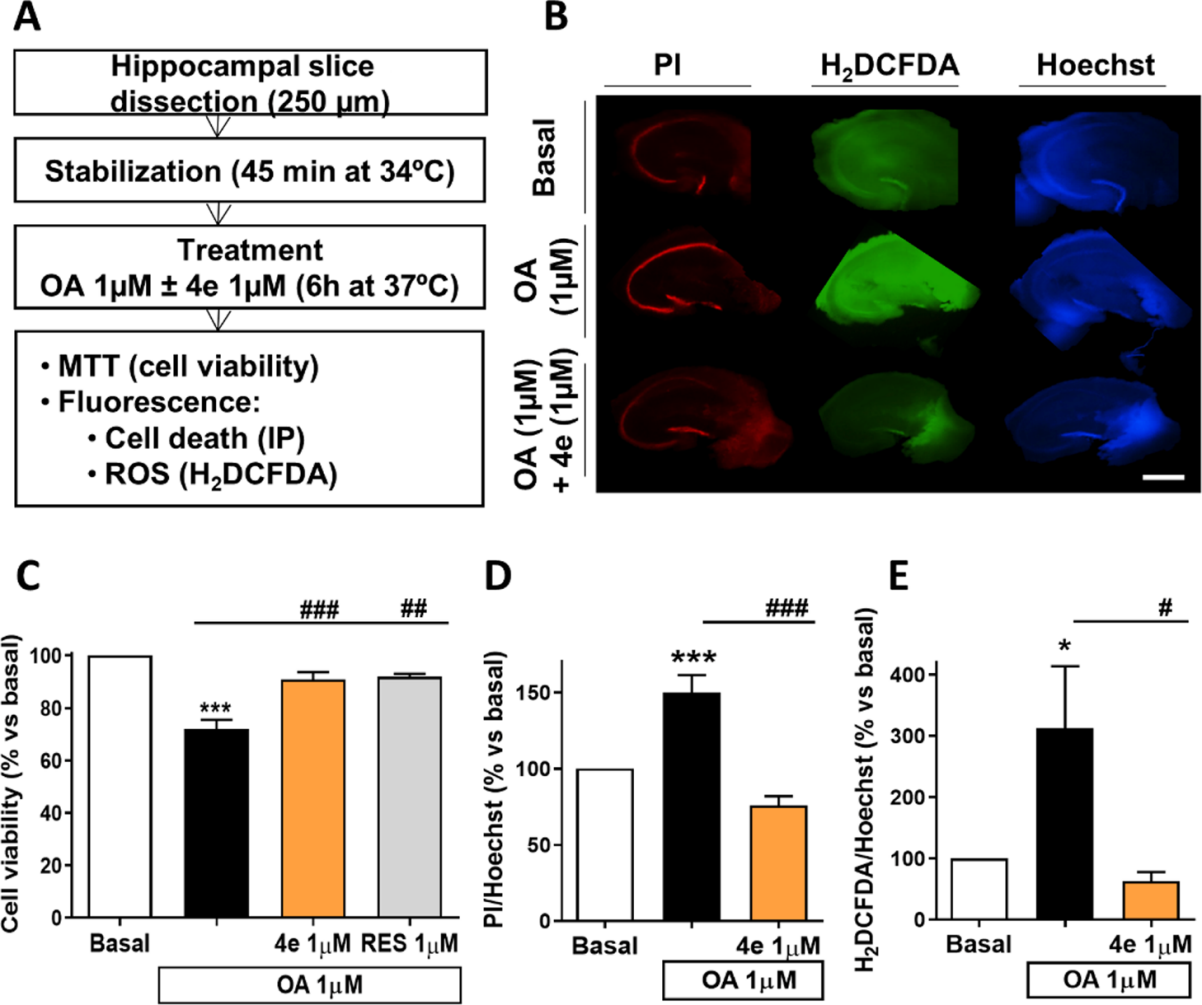
Compound **4e** conferred neuroprotection and reduced ROS production in mice hippocampal slices exposed to OA. (A) Protocol scheme followed for treatment with OA (1 μM) with or without tested compounds (**4e** and resveratrol, 1 μM). (B) Representative images for cell death (PI) and ROS production (H_2_DCFDA) normalized to Hoechst fluorescence (scale bar = 1000 μm). (C) Cell viability measured with MTT colorimetric assay. (D) Cell death measured with the fluorescent dye PI. (E) ROS production determined with the H_2_DCFDA dye. One-way ANOVA followed by Tukey’s posthoc test. Statistical differences are represented as *p < 0.05, **p < 0.01, and ***p < 0.001 in comparison to the basal condition. #*p* < 0.05, ##*p* < 0.01, and ###*p* < 0.001 in comparison to OA. Results are displayed as mean ± SEM (*N* = 5–6).

As shown in Figure 9A, hippocampal slices (250 μm thick) were treated with saline or OA (1 μM) with or without compound **4e** (1 μM) or resveratrol (RES, 1 μM) for 6 h. OA treatment increased cell death and ROS production, as previously reported.^75^ Compound **4e** significantly increased the cell viability to almost basal levels, in a similar way to resveratrol treatment, measured by the MTT method (Figure 9C). Alternatively, compound **4e** also reduced cell death in this model, measured as propidium iodide (PI) uptake fluorescence (Figure 9B,D). Remarkably, compound **4e** reduced ROS production to basal levels, determined using the fluorescent probe 2′,7′-dichlorodihydrofluorescein diacetate (H_2_DCFDA) (Figure 9B,E). Therefore, compound **4e** reduced OA toxicity and OA-related oxidative stress in an acute tissue model of AD.

To gain insight on the potential mechanism of action of **4e**, different proteins related to inflammation and oxidative stress (iNOS, p65, and HMOX-1) were further evaluated using western blotting (WB) experiments (Supporting Information-1, Figure S17). Treatment with OA produced a slight increase in the inducible nitric oxide synthase (iNOS), an effect that was reduced to basal levels when the slices were treated with compound **4e**. As iNOS can be produced through the NF-κB (nuclear factor kappa-light-chain-enhancer of activated B cells) pathway, we analyzed the levels of p65, one of the components that form the NF-κB transcription factor family.^82^ As observed with iNOS, OA treatment caused a slight increase in the p65 levels, whereas compound **4e** slightly reduced p65 below to the basal value. Taken together, these results indicate that compound **4e** could be attenuating the proinflammatory response by regulating the NF-κB pathway. We also measured HO-1, an inducible enzyme transcribed by NRF2, known for its anti-inflammatory, antioxidant, and neuroprotective effects. We observed a trend to increase HO-1 in the hippocampal slices treated with OA, which further increased in the presence of compound **4e** (Supporting Information-1, Figure S17). This effect can be related to the ability of **4e** to induce NRF2 and its antioxidant and neuroprotective actions. NRF2 and NF-κB signaling pathways cooperate to maintain the physiological homeostasis of cellular redox status and to regulate the cellular response to stress and inflammation.^19,83^ NRF2 has been shown to negatively control NF-κB signaling by different mechanisms such as by reducing intracellular ROS levels, by preventing IkB-a proteasome degradation and inhibiting the translocation of NF-kB, and by blocking the degradation of IkB-a by HO-1.^84,85^ This last mechanism could be related to our resveratrol-based MTDL **4e**.

#### Compound **4e** Has Neuroprotective and Antioxidant Properties against AO in a Chronic In Vitro Model of AD

**Figure 10:**
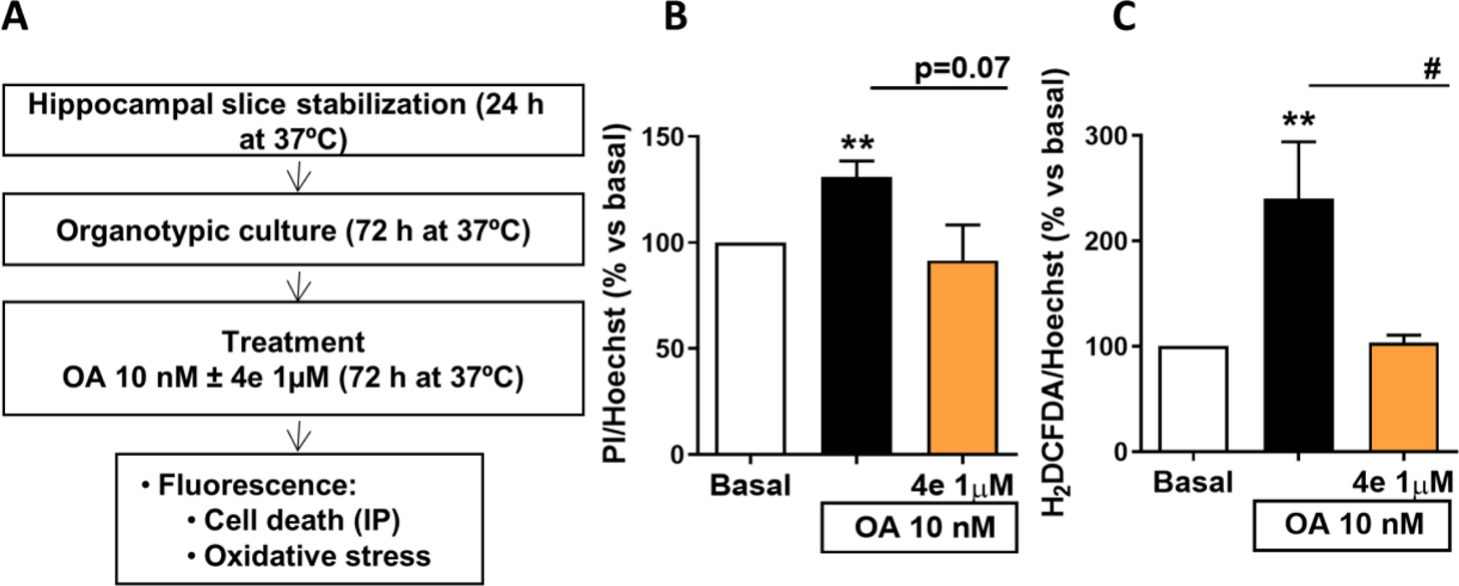
Compound **4e** reduces ROS production and cell death in organotypic hippocampal slices subjected to OA-induced toxicity. (A) Protocol scheme followed for the treatment with OA (10 nM) with or without compound **4e** (1 μM) in organotypic cultures. (B) OA (10 nM) significantly increased cell death measured as an increase in the PI mean intensity. Treatment with **4e** (1.0 μM) reduced hippocampal death with a statistical trend (*p* = 0.07). (C) OA (10 nM) significantly increased ROS production measured with the fluorescent dye H_2_DCFDA. Compound **4e** (1 μM) was able to significantly decrease ROS production to basal levels. One-way ANOVA followed by Tukey’s posthoc test. Statistical differences are represented as **p < 0.01 in comparison to the basal condition. #*p* < 0.05 in comparison to OA. Statistical trend was considered when *p* < 0.10. Results are displayed as mean ± SEM (*N* = 5–6).

Since the resveratrol-based MTDL **4e** showed a positive effect against an acute treatment with OA, we aimed to study its effects in a chronic and more complex in vitro model of AD. For this purpose, mice hippocampal organotypic cultures were used, as shown in Figure 10A. Hippocampal mouse slices were stabilized for 24 h, cultured for 72 h, and treated with OA for 72 h with or without compound **4e**. Chronic OA treatment (10 nM) significantly increased cell death (Figure 10B) and ROS production (Figure 10D). Treatment with derivative **4e** was able to reduce cell death; although the differences were not statistically significant, it showed a statistical trend (*p* = 0.07) (Figure 10B). Moreover, **4e** significantly reduced ROS production, as presented in Figure 10C. These results reinforce the neuroprotective and antioxidant properties of **4e** against OA toxicity in acute and chronic in vitro models of AD.

Given that the 4-methoxyphenyl-*N*-propargyloxadiazolone derivative **4e** has emerged as an interesting resveratrol-based MTDL, we explored the phase-I metabolism reactions and their products using BioTransformer, an in silico software tool that predicts small-molecule metabolism and metabolite identification.^86^ According to BioTransformer, there are three main phase-I (CYP450) metabolites of **4e**, as a result of O-dealkylation, epoxidation of alkene, and epoxidation of the phenyl ring (Supporting Information-1, Figure S18). Interestingly, none of these three metabolites gave any toxicological alert after they were filtered through a KNIME workflow (Supporting Information-1, Figure S15). Therefore, **4e** and its metabolites are expected to have low toxicity in the upcoming *in vivo* tests.

## Conclusions

In summary, new resveratrol-based MTDLs have been obtained by the replacement of a phenolic ring of (*E*)-resveratrol with an 1,3,4-oxadiazol-2(3*H*)-one heterocycle or an amide/amine group, including a propargyl amine fragment to increase their potency and selectivity toward MAO-B. New compounds were tested in a battery of biological assays related to NDs (hMAO-A/B, NRF2, QR2, and ROS trapping), then, in experiments of increasing complexity, performed in primary neuronal cultures (neurogenic and neuroprotection assays), and then in tissue-based AD models.

According to our design, compounds bearing a propargyl fragment in the oxadiazolone ring displayed an interesting MTD profile. At low-micromolar and sub-micromolar concentrations, phenyl-*N*-propargyloxadiazolone derivatives showed dual inhibition of hMAO-B and QR2, with high selectivity as they are not active in related targets (e.g., hMAO-A, *h*MT_1_R, and *h*MT_2_R). In contrast to the slight selectivity toward hMAO-A of resveratrol, most of our active derivatives showed a selective inhibition of hMAO-B that could be of interest for the treatment of PD and AD. Enzymatic assays on MAO-B showed that they have a reversible behavior, and therefore, they would avoid the side effects of irreversible inhibitors. Molecular docking studies on MAO-B and QR2 revealed the main interactions with these proteins, which could be used further in structural optimization.

In cell-based assays, phenyl-*N*-propargyloxadiazolone derivatives activated the signaling pathway mediated by the transcription factor NRF2, promoted the maturation of neural stem cells into a neuronal phenotype, and exerted neuroprotective effects against OA-induced toxicity. At high concentrations, new compounds have no toxicity, which differed from the reference compound resveratrol that reduced over 50% cell viability.

The biological properties (NRF2 activation, selective QR2 inhibition, selective and reversible MAO-B inhibition, the best behavior in promoting different neurogenic processes) and drug-like profile (positive in vitro CNS permeability, good physiological solubility, no glutathione conjugation, absence of PAINS, or Lipinski alerts) allowed us to select 5-[(*E*)-2-(4-methoxyphenyl)ethenyl]-3-(prop-2-yn-1-yl)-1,3,4-oxadiazol-2(3*H*)-one (**4e**) for tissue AD models. In mice hippocampal slices that were acutely or chronically exposed to OA, compound **4e** exerted good neuroprotective and antioxidant properties.

These outstanding properties, along with the absence of predicted toxicological alerts, make the resveratrol-based MTDL **4e** an interesting MTDL for the upcoming *in vivo* tests, which could stimulate defensive and regenerative pathways and block early events in neurodegenerative cascades.

## Experimental Section

### Chemistry

#### General Methods

High-grade reagents and solvents were purchased from commercial suppliers and were used without further purification. Reactions were followed by analytical thin-layer chromatography (TLC plates, Merck silica gel 60 F254), and compounds were detected under UV light (λ = 254 or 365 nm) and/or stained with 10% wt. phosphomolybdic acid solution in EtOH. High-performance liquid chromatography coupled to mass spectrometry (HPLC–MS) was performed on a Waters analytical (Alliance Watters 2695) instrument equipped with a SunFire C_18_ (3.5 μm, 4.6 mm × 50 mm) column, a UV–visible photodiode array detector (λ = 190–700 nm), and a quadrupole mass spectrometer (Micromass ZQ). The spectra were acquired in an electrospray ionization (ESI) interface working in the positive- or negative-ion mode. Reactions under microwave (MW) irradiation were performed in a Biotage Initiator 2.5 reactor. Unless otherwise stated, the products were purified by automatized flash chromatography using an IsoleraOne (Biotage) equipment, with cartridges of silica gel Biotage ZIP KP-Sil 50 μm. Alternatively, preparative TLC on Merck silica gel 60 F254 plates or by semipreparative HPLC on a Waters Autopurification system with a UV–visible photodiode array detector (λ = 190–700 nm) coupled to a quadrupole mass spectrometer (3100 Mass Detector) was used. HPLC analyses were used to confirm the purity of all compounds (≥95%) and were performed on Waters 2690 equipment at a flow rate of 1.0 mL/min, with a UV–visible photodiode array detector (λ = 190–700 nm), using a SunFire C_18_ (3.5 μm, 4.6 mm × 50 mm) column. The gradient mobile phase consisted of H_2_O/ACN with 0.1% formic acid as solvent modifiers, and the gradient time (g.t.) is indicated for each compound. The melting point (mp) (uncorrected) was determined using an MP70 apparatus (Mettler Toledo). Nuclear magnetic resonance (^1^H NMR and ^13^C NMR) spectra were obtained in MeOD, acetone-*d*_6_, D_2_O, DMSO-*d*_6_, or CDCl_3_ solutions using the following NMR spectrometers: Varian INOVA-300, Varian INOVA-400, Varian Mercury-400, or Varian Unity-500. The chemical shifts (δ) are reported in parts per million (ppm) relative to the internal tetramethylsilane scale, and coupling constants (*J*) values are expressed in hertz (Hz). 2D NMR experiments, namely, homonuclear correlation spectroscopy (H, H-COSY), heteronuclear multiple quantum correlation (HMQC) spectroscopy, and HMBC spectroscopy, were carried out to assign protons and carbons of new structures. High-resolution mass spectroscopy (HRMS) analyses were carried out in an Agilent 1200 Series LC system (equipped with a binary pump, an autosampler, and a column oven) coupled to a 6520 quadrupole time-of-flight (QTOF) mass spectrometer. ACN/H_2_O (75:25, v/v) was used as the mobile phase at 0.2 mL/min. The ionization source was an ESI interface working in the positive-ion mode. The electrospray voltage was set at 4.5 kV, the fragmentor voltage at 150 V, and the drying gas temperature at 300 °C. Nitrogen (99.5% purity) was used as the nebulizer (207 kPa) and drying gas (6 L/min).

#### Synthesis of 1,3,4-Oxadiazol-2(3*H*)-one Derivatives

To a suspension of the corresponding acid (1 equiv) and activated 4 Å molecular sieves in anhydrous ACN (15 mL/mmol) at rt under a N_2_ atmosphere, HOBt (1.2 equiv), EDC·HCl (1.2 equiv), and DMAP (0.12 equiv) were added orderly. The mixture was stirred until the complete activation of the acid (30 min to 3 h), and then an excess of N_2_H_4_·H_2_O (1.2 equiv) was added at rt. After completion of the reaction (at the end of the addition in most cases), H_2_O was added and the mixture was extracted with DCM (×3) and washed with saturated NaHCO_3_ (aq). The organic layer was dried over MgSO_4_, filtered, and evaporated to dryness under reduced pressure, obtaining *N*-acylhydrazides that were used without further purification. Thus, to a solution of the corresponding hydrazide (1 equiv) in anhydrous DMF (10 mL/mmol), CDI (1.2 equiv) was added under a N_2_ atmosphere. The reaction mixture was heated at 120 °C for 25 min under MW irradiation, and the solvent was removed to dryness under reduced pressure. The residue was redissolved in EtOAc, washed with H_2_O and brine, dried over Mg_2_SO_4_, filtered, and evaporated under reduced pressure. The crude was purified by flash chromatography on silica gel using the adequate gradient to obtain the desired 1,3,4-oxadiazol-2(3*H*)-one derivative.

##### 5-[(*E*)-2-Phenylethenyl]-1,3,4-oxadiazol-2(3*H*)-one (**2a**)

Chromatography: hexane to hexane/EtOAc 80:20. White solid (65% yield) of mp 191–194 °C (lit.^87^ 191–193 °C). ^1^H NMR (500 MHz, MeOD): δ 7.61–7.58 (m, 2H, H_2_,_6_), 7.43–7.37 (m, 3H, H_3–5_), 7.34 (d, *J* = 16.5 Hz, 1H, H_α_), 6.78 (d, *J* = 16.5 Hz, 1H, H_β_). ^13^C NMR (126 MHz, MeOD): δ 156.5 (CO), 156.5 (C_5′_), 138.7 (C_α_), 136.2 (C_1_), 130.8 (C_4_), 130.0 (C_3,5_), 128.5 (C_2,6_), 111.5 (C_β_). HPLC-MS (15:95-g.t.10 min) *t*_R_ 6.41 min, *m*/*z*: 189.30 [M + H]^+^; calcd for [C_10_H_8_N_2_O_2_+H]^+^, 189.19. HRMS [ESI^+^] *m*/*z*: 188.05822 [M]^+^; calcd for [C_10_H_8_N_2_O_2_]^+^, 188.05858.

##### 3-[(*E*)-2-(5-Oxo-4,5-dihydro-1,3,4-oxadiazol-2-yl)ethenyl]benzonitrile (**2b**)

Chromatography: hexane to hexane/EtOAc 75:25. White solid (62% yield) of mp 244–247 °C. ^1^H NMR (400 MHz, MeOD): δ 8.01 (s, 1H, H_2_), 7.92 (d, *J* = 7.8 Hz, 1H, H_4_), 7.71 (d, *J* = 7.8 Hz, 1H, H_6_), 7.59 (t, *J* = 7.8 Hz, 1H, H_5_), 7.37 (d, *J* = 16.4 Hz, 1H, H_α_), 6.94 (d, *J* = 16.4 Hz, 1H, H_β_). HPLC-MS (30:95-g.t.10 min) *t*_R_ 2.18 min, *m*/*z*: 212.26 [M – H]^−^; calcd for [C_11_H_7_N_3_O_2_ – H]^−^, 212.20; purity 97%. HRMS [ESI^+^] *m*/*z*: 213.05295 [M]^+^; calcd for [C_11_H_7_N_3_O_2_]^+^, 213.05383.

##### 5-[(*E*)-2-(3-Nitrophenyl)ethenyl]-1,3,4-oxadiazol-2(3*H*)-one (**2c**)

Chromatography: hexane to hexane/EtOAc 60:40. White solid (92% yield) of mp 232–234 °C. ^1^H NMR (500 MHz, DMSO-*d*_6_): δ 9.38 (t, *J* = 1.9 Hz, 1H, H_2_), 9.04–8.98 (m, 2H, H_4,6_), 8.51 (t, *J* = 8.0 Hz, 1H, H_5_), 8.29 (d, *J* = 16.5 Hz, 1H, H_α_), 8.03 (d, *J* = 16.5 Hz, 1H, H_β_). ^13^C NMR (126 MHz, DMSO-*d*_6_): δ 163.5 (C_2′_), 163.3 (C_5′_), 157.9 (C_3_), 146.1 (C_1_), 143.9 (C_α_), 142.9 (C_6_), 139.8 (C_5_), 133.3 (C_4_), 131.8 (C_2_), 123.3 (C_β_). HPLC-MS (15:95-g.t.10 min) *t*_R_ 6.47 min, *m*/*z*: 232.26 [M – H]^−^; calcd for [C_10_H_7_N_3_O_4_ – H]^−^, 232.18; purity: 98%. HRMS [ESI^+^] *m*/*z*: 233.04337 [M]^+^; calcd for [C_10_H_7_N_3_O_4_]^+^, 233.04366.

##### 5-[(*E*)-2-(3-Methoxyphenyl)ethenyl]-1,3,4-oxadiazol-2(3*H*)-one (**2d**)

Chromatography: hexane to hexane/EtOAc 8:2. White solid (96% yield) of mp 178–180 °C. ^1^H NMR (500 MHz, MeOD): δ 7.32 (d, *J* = 16.5 Hz, 1H, H_α_), 7.31 (t, *J* = 7.9 Hz, 1H, H_5_), 7.17 (d, *J* = 7.9 Hz, 1H, H_6_), 7.15 (br s, 1H, H_2_), 6.94 (t, *J* = 7.9 Hz, 1H, H_4_), 6.78 (d, *J* = 16.5 Hz, 1H, H_β_), 3.83 (s, 3H, CH_3_). ^13^C NMR (126 MHz, MeOD): δ 161.6 (C_3_), 156.6 (C_2′_), 156.5 (C_5′_), 138.6 (C_α_), 137.6 (C_1_), 131.0 (C_5_), 121.1 (C_6_), 116.7 (C_4_), 113.4 (C_2_), 111.8 (C_β_), 55.8 (CH_3_). HPLC-MS (15:95-g.t.5 min) *t*_R_ 4.07 min, *m*/*z*: 219.18 [M + H]^+^; calcd for [C_11_H_10_N_2_O_3_ +H]^+^, 219.21; purity: 100%. HRMS [ESI^+^] *m*/*z*: 218.06887 [M]^+^; calcd for [C_11_H_10_N_2_O_3_]^+^, 218.06914.

##### 5-[(*E*)-2-(4-Methoxyphenyl)ethenyl]-1,3,4-oxadiazol-2(3*H*)-one (**2e**)

Chromatography: hexane to hexane/EtOAc 65:35. White solid (82% yield) of mp 191–193 °C. ^1^H NMR (400 MHz, MeOD): δ 7.53 (d, *J* = 8.8 Hz, 2H, H_2,6_), 7.28 (d, *J* = 16.4 Hz, 1H, H_α_), 6.94 (d, *J* = 8.8 Hz, 2H, H_3,5_), 6.61 (d, *J* = 16.4 Hz, 1H, H_β_), 3.82 (s, 3H, CH_3_). ^13^C NMR (101 MHz, MeOD): δ 162.6 (C_4_), 156.8 (C_5′_), 156.6 (C_2′_), 138.5 (C_α_), 130.1 (C_2,6_), 128.9 (C_1_), 115.4 (C_3,5_), 109.0 (C_β_), 55.8 (CH_3_). HPLC-MS (15:95-g.t.5 min) *t*_R_ 3.98 min, *m*/*z*: 219.34 [M + H]^+^; calcd for [C_11_H_10_N_2_O_3_ + H]^+^, 219.21; purity: 100%. HRMS [ESI^+^] *m*/*z*: 218.06832 [M]^+^; calcd for [C_11_H_10_N_2_O_3_]^+^, 218.06914.

##### 5-[(*E*)-2-(2,4-Dimethoxyphenyl)ethenyl]-1,3,4-oxadiazol-2(3*H*)-one (**2f**)

Chromatography: hexane to hexane/EtOAc, 65:35. White solid (76% yield) of mp 220–222 °C. ^1^H NMR (500 MHz, MeOD): δ 7.49 (d, *J* = 16.4 Hz, 1H, H_α_), 7.49 (s, 1H, H_3_), 6.69 (d, *J* = 16.4 Hz, 1H, H_β_), 6.60–6.54 (m, 2H, H_5,6_), 3.91 (s, 3H, C_2_OCH_3_), 3.84 (s, 3H, C_4_OCH_3_). ^13^C NMR (126 MHz, MeOD): δ 164.1 (C_4_), 160.8 (C_2_), 157.4 (C_5′_), 156.8 (C_2′_), 134.1 (C_α_), 130.7 (C_6_), 117.7 (C_1_), 109.1 (C_β_), 106.9 (C_3_), 99.2 (C_5_), 56.1(C_2_OCH_3_), 55.9 (C_4_OCH_3_). HPLC-MS (15:95-g.t.5 min) *t*_R_ 4.13 min, *m*/*z*: 249.15 [M + H]^+^; calcd for [C_12_H_12_N_2_O_4_ + H]^+^, 249.24; purity: 99%. HRMS [ESI^+^] *m*/*z*: 248.07922 [M]^+^; calcd for [C_12_H_12_N_2_O_4_]^+^, 248.07971.

##### 5-[(*E*)-2-(2,5-Dimethoxyphenyl)ethenyl]-1,3,4-oxadiazol-2(3*H*)-one (**2g**)

Chromatography: hexane to hexane/EtOAc 65:35. White solid (79% yield) of mp 124–126 °C. ^1^H NMR (400 MHz, MeOD): δ 7.58 (d, *J* = 16.6 Hz, 1H, H_α_), 7.15 (d, *J* = 2.9 Hz, 1H, H_6_), 6.99 (d, *J* = 9.0 Hz, 1H, H_3_), 6.95 (dd, *J* = 9.0, 2.9 Hz, 1H, H_4_), 6.84 (d, *J* = 16.6 Hz, 1H, H_β_), 3.88 (s, 3H, C_2_OCH_3_), 3.80 (s, 3H, C_5_OCH_3_). ^13^C NMR (101 MHz, MeOD): δ 156.9 (C_5′_), 156.7 (C_2′_), 155.2 (C_5_), 153.8 (C_2_), 133.8 (C_α_), 125.3 (C_1_), 117.7 (C_4_), 113.8 (C_6_), 113.7 (C_3_), 112.0 (C_β_), 56.6 (C_2_OCH_3_), 56.2 (C_5_OCH_3_). HPLC-MS (15:95-g.t.5 min) *t*_R_ 1.02 min, *m*/*z*: 249.15 [M + H]^+^; calcd for [C_12_H_12_N_2_O_4_+H]^+^, 249.24; purity > 99%. HRMS [ESI^+^] *m*/*z*: 248.08094 [M]^+^; calcd for [C_12_H_12_N_2_O_4_]^+^, 248.07971.

##### 5-[(*E*)-2-(3,4-Dimethoxyphenyl)ethenyl]-1,3,4-oxadiazol-2(3*H*)-one (**2h**)

Chromatography: hexane to hexane/EtOAc 65:35. White solid (64% yield) of mp 248–251 °C. ^1^H NMR (500 MHz, DMSO-*d*_6_): δ 7.36 (d, *J* = 2.0 Hz, 1H, H_2_), 7.23 (d, *J* = 16.5 Hz, 1H, H_α_), 7.20 (dd, *J* = 8.4, 2.0 Hz, 1H, H_6_), 6.97 (d, *J* = 8.4 Hz, 1H, H_5_), 6.87 (d, *J* = 16.4 Hz, 1H, H_β_), 3.81 (s, 3H, C_3_OCH_3_), 3.78 (s, 3H, C_4_OCH_3_). ^13^C NMR (126 MHz, DMSO-*d*_6_): δ 154.6 (C_5′_), 154.1 (C_2′_), 150.3(C_4_), 149.0 (C_3_), 136.9 (C_α_), 127.6 (C_1_), 122.0 (C_6_), 111.5 (C_5_), 109.7 (C_2_), 108.5 (C_β_), 55.6 (C_3_OCH_3_), 55.5 (C_4_OCH_3_). HPLC-MS (15:95-g.t.5 min) *t*_R_ 3.63 min, *m*/*z*: 249.15 [M + H]^+^; calcd for [C_12_H_12_N_2_O_4_+H]^+^, 249.24; purity: 97%. HRMS [ESI^+^] *m*/*z*: 248.08081 [M]^+^; calcd for [C_12_H_12_N_2_O_4_]^+^, 248.07971.

##### 5-[(*E*)-2-(2*H*-1,3-Benzodioxol-5-yl)ethenyl]-1,3,4-oxadiazol-2(3*H*)-one (**2i**)

Chromatography: hexane to hexane/EtOAc 65:35. White solid (86% yield) of mp 245–248 °C. ^1^H NMR (400 MHz, MeOD): δ 7.28 (d, *J* = 16.4 Hz, 1H, H_α_), 7.19 (d, *J* = 1.8 Hz, 1H, H_2_), 7.08 (dd, *J* = 8.0, 1.8 Hz, 1H, H_6_), 6.86 (d, *J* = 8.0 Hz, 1H, H_5_), 6.63 (d, *J* = 16.3 Hz, 1H, H_β_), 6.02 (s, 2H, CH_2_). ^13^C NMR (101 MHz, MeOD): δ 156.7 (C_5′_), 156.6 (C_2′_), 150.7 (C_4_), 150.0 (C_3_), 138.5 (C_α_), 130.7 (C_1_), 124.7 (C_6_), 109.5 (C_β_, C_5_), 106.9 (C_2_), 103.0 (CH_2_). HPLC-MS (15:95-g.t.5 min) *t*_R_ 3.89 min, *m*/*z*: 233.19 [M + H]^+^; calcd for [C_11_H_8_N_2_O_4_+H]^+^, 232.20; purity: 97%. HRMS [ESI^+^] *m*/*z*: 232.04873 [M]^+^; calcd for [C_11_H_8_N_2_O_4_]^+^, 232.04841.

##### 5-[(*E*)-2-(3,5-Dimethoxyphenyl)ethenyl]-1,3,4-oxadiazol-2(3*H*)-one (**2j**)

Chromatography: hexane to hexane/EtOAc 1:1. White solid (74% yield) of mp 156–159 °C. ^1^H NMR (400 MHz, MeOD): δ 7.29 (d, *J* = 16.3 Hz, 1H, H_α_), 6.79 (d, *J* = 16.3 Hz, 1H, H_β_), 6.78 (d, *J* = 2.2 Hz, 2H, H_2,6_), 6.53 (t, *J* = 2.2 Hz, 1H, H_4_), 3.83 (s, 6H, 2CH_3_). ^13^C NMR (101 MHz, MeOD): δ 162.7 (C_3,5_), 156.6 (C_2′_), 156.4 (C_5′_), 138.8 (C_α_), 138.1 (C_1_), 112.0 (C_β_), 106.4 (C_2,6_), 103.0 (C_4_), 55.9 (2CH_3_). HPLC-MS (15:95-g.t.5 min) *t*_R_ 4.17 min, *m*/*z*: 249.23 [M + H]^+^; calcd for [C_12_H_12_N_2_O_4_+H]^+^, 249.24; purity: 99%. HRMS [ESI^+^] *m*/*z*: 248.08033 [M]^+^; calcd for [C_12_H_12_N_2_O_4_]^+^, 248.07971.

##### 5-[(*E*)-2-(3,4,5-Trimethoxyphenyl)ethenyl]-1,3,4-oxadiazol-2(3*H*)-one (**2k**)

Chromatography: hexane to hexane/EtOAc 1:1. White solid (68% yield) of mp 214–217 °C. ^1^H NMR (500 MHz, MeOD): δ 7.43 (d, *J* = 16.3 Hz, 1H, H_α_), 7.06 (s, 2H, H_2,6_), 6.87 (d, *J* = 16.3 Hz, 1H, H_β_), 4.03 (s, 6H, C_3_OCH_3_, C_5_OCH_3_), 3.94 (s, 3H, C_4_OCH_3_). ^13^C NMR (126 MHz, MeOD): δ 156.7 (C_2′_), 156.6 (C_5′_), 154.9 (C_3,5_), 140.9 (C_4_), 138.7 (C_α_), 132.2 (C_1_), 111.0 (C_β_), 106.3 (C_2,6_), 61.2 (C_4_OCH_3_), 56.8 (C_3_OCH_3_, C_5_OCH_3_). HPLC-MS (15:95-g.t.5 min) *t*_R_ 3.77 min, *m*/*z*: 279.12 [M + H]^+^; calcd for [C_13_H_14_N_2_O_5_+H]^+^, 279.26; purity: 99%. HRMS [ESI^+^] *m*/*z*: 278.09113 [M]^+^; calcd for [C_13_H_14_N_2_O_5_]^+^, 278.09027.

#### General Procedure for the Synthesis of Hydroxyl Derivatives from Their Methoxylated Counterparts

To a solution of the corresponding methoxylated compound in the minimum amount of anhydrous DCM, a 1 M BBr_3_ solution in DCM was added dropwise under a N_2_ atmosphere (1 equiv of BBr_3_ per heteroatom present in the molecule). The mixture was left at rt overnight, and then, it was quenched with MeOH (dropwise until end of effervescence). The solvent was evaporated under reduced pressure to remove the remaining BBr_3_, repeating this process several times until no fumes were observed when adding MeOH. Crude materials were purified by flash chromatography or by washing with MeOH, obtaining the corresponding phenolic derivatives.

##### 5-[(*E*)-2-(3-Hydroxyphenyl)ethenyl]-1,3,4-oxadiazol-2(3*H*)-one (**3l**)

Chromatography: hexane to hexane/EtOAc 1:1. White solid (91% yield) of mp 230–233 °C. ^1^H NMR (400 MHz, MeOD): δ 7.29 (d, *J* = 16.4 Hz, 1H, H_α_), 7.24 (t, *J* = 7.7 Hz, 1H, H_5_), 7.08 (d, *J* = 7.7 Hz, 1H, H_6_), 7.02 (t, *J* = 2.0 Hz, 1H, H_2_), 6.83 (dd, *J* = 7.7, 2.0 Hz, 1H, H_4_), 6.72 (d, *J* = 16.4 Hz, 1H, H_β_). ^13^C NMR (101 MHz, MeOD): δ 159.1 (C_3_), 156.5 (C_2′_), 156.5 (C_5′_), 138.9 (C_α_), 137.5 (C_1_), 131.0 (C_5_), 120.1 (C_6_), 118.0 (C_4_), 114.6 (C_2_), 111.3 (C_β_). HPLC-MS (15:95-g.t.5 min) *t*_R_ 3.12 min, *m*/*z*: 205.18 [M + H]^+^; calcd for [C_10_H_8_N_2_O_3_ + H]^+^, 205.19; purity: 99%. HRMS [ESI^+^] *m*/*z*: 204.05432 [M]^+^; calcd for [C_10_H_8_N_2_O_3_]^+^, 204.05349.

##### 5-[(*E*)-2-(4-Hydroxyphenyl)ethenyl]-1,3,4-oxadiazol-2(3*H*)-one (**3m**)

Purification: crude washed with MeOH. White solid (91% yield) of mp 255–258 °C. ^1^H NMR (500 MHz, MeOD): δ 7.44 (d, *J* = 8.4 Hz, 2H, H_2,6_), 7.25 (d, *J* = 16.3 Hz, 1H, H_α_), 6.80 (d, *J* = 8.4 Hz, 2H, H_3,5_), 6.56 (d, *J* = 16.3 Hz, 1H, H_β_). ^13^C NMR (126 MHz, MeOD): δ 160.6 (C_4_), 157.0 (C_5′_), 156.7 (C_2′_), 138.9 (C_α_), 130.3 (C_2,6_), 127.7 (C_1_), 116.8 (C_3,5_), 108.1 (C_β_). HPLC-MS (15:95-g.t.5 min) *t*_R_ 3.12 min, *m*/*z*: 205.18 [M + H]^+^; calcd for [C_10_H_8_N_2_O_3_+H]^+^, 205.19; purity: 98%. HRMS [ESI^+^] *m*/*z*: 204.05403 [M]^+^; calcd for [C_10_H_8_N_2_O_3_]^+^, 204.05349.

##### 5-[(*E*)-2-(3,4-Dihydroxyphenyl)ethenyl]-1,3,4-oxadiazol-2(3*H*)-one (**3o**)

Chromatography: hexane to hexane/EtOAc 1:1. White solid (89% yield) of mp 253–256 °C. ^1^H NMR (500 MHz, MeOD): δ 7.20 (d, *J* = 16.3 Hz, 1H, H_α_), 7.03 (d, *J* = 2.1 Hz, 1H, H_2_), 6.93 (dd, *J* = 8.1, 2.1 Hz, 1H, H_6_), 6.78 (d, *J* = 8.1 Hz, 1H, H_5_), 6.50 (d, *J* = 16.3 Hz, 1H, H_β_).^13^C NMR (126 MHz, MeOD): δ 157.0 (C_5′_), 156.7 (C_2′_), 148.9 (C_4_), 146.8 (C_3_), 139.2 (C_α_), 128.3 (C_1_), 121.9 (C_6_), 116.5 (C_5_), 114.5 (C_2_), 108.0 (C_β_). HPLC-MS (15:95-g.t.5 min) *t*_R_ 2.71 min, *m*/*z*: 221.13 [M + H]^+^; calcd for [C_10_H_8_N_2_O_4_ +H]^+^, 221.18; purity: 100%. HRMS [ESI^+^] *m*/*z*: 220.04861 [M]^+^; calcd for [C_10_H_8_N_2_O_4_]^+^, 220.04841.

##### 5-[(*E*)-2-(3,5-Dihydroxyphenyl)ethenyl]-1,3,4-oxadiazol-2(3*H*)-one (**3p**)

Chromatography: hexane to EtOAc. White solid (66% yield) of mp 264–267 °C. ^1^H NMR (400 MHz, MeOD): δ 7.19 (d, *J* = 16.3 Hz, 1H, H_α_), 6.64 (d, *J* = 16.3 Hz, 1H, H_β_), 6.52 (d, *J* = 2.2 Hz, 2H, H_2,6_), 6.31 (t, *J* = 2.2 Hz, 1H, H_4_). ^13^C NMR (101 MHz, MeOD): δ 160.1 (C_3,5_), 156.7 (C_2′_), 156.5 (C_5′_), 139.1 (C_α_), 138.0 (C_1_), 111.2 (C_β_), 106.9 (C_2,6_). HPLC-MS (15:95-g.t.5 min) *t*_R_ 2.43 min, *m*/*z*: 221.13 [M + H]^+^; calcd for [C_10_H_8_N_2_O_4_+H]^+^, 221.18; purity: 95%. HRMS [ESI^+^] *m*/*z*: 220.04837 [M]^+^; calcd for [C_10_H_8_N_2_O_4_]^+^, 220.04841.

#### General Procedure for the NH-Alkylation of 1,3,4-Oxadiazol-2(3*H*)-one Derivatives

A mixture of 1,3,4-oxadiazol-2(3*H*)-one derivative (1.0 equiv) and K_2_CO_3_ (1.2 equiv) in acetone (7 mL/mmol) was stirred at rt for 10 min. Propargyl bromide (1.2 equiv) was added, and the mixture was heated under MW irradiation at 120 °C for 10 min. In the case of 3-hydroxyphenyl derivative **3l**, the reaction mixture was left at rt overnight instead of using MW. When the reaction was complete, the solvent was evaporated under reduced pressure, EtOAc was added, and the organic layer was washed with H_2_O (×3) and brine, dried over Mg_2_SO_4_, filtered, and evaporated under reduced pressure. The crude was purified by chromatography using an appropriate eluent to afford the corresponding *N*-propargyl derivative.

##### 5-[(*E*)-2-Phenylethenyl]-3-(prop-2-yn-1-yl)-1,3,4-oxadiazol-2(3*H*)-one (**4a**)

Chromatography: hexane to hexane/EtOAc 77:23. White solid (87% yield) of mp 138–140 °C. ^1^H NMR (500 MHz, DMSO-*d*_6_): δ 7.73 (dd, *J* = 8.1, 1.4 Hz, 1H, H_2,6_), 7.45–7.40 (m, 3H, H_3–5_), 7.38 (d, *J* = 16.5 Hz, 1H, H_α_), 7.01 (d, *J* = 16.5 Hz, 1H, H_β_), 4.61 (d, *J* = 2.5 Hz, 2H, CH_2_), 3.48 (t, *J* = 2.5 Hz, 1H, ≡CH). ^13^C NMR (126 MHz, DMSO-*d*_6_): δ 153.1 (C_5′_), 151.9 (C_2′_), 137.8 (C_α_), 134.5 (C_1_), 129.9 (C_4_), 128.9 (C_3,5_), 127.8 (C_2,6_), 110.2 (C_β_), 77.1 (≡C), 76.2 (≡CH), 35.4 (CH_2_). HPLC-MS (15:95-g.t.10 min) *t*_R_ 8.00 min, *m*/*z*: 227.20 [M + H]^+^; calcd for [C_13_H_10_N_2_O_2_ + H]^+^, 227.24; purity: 99%. HRMS [ESI^+^] *m*/*z*: 226.07401 [M]^+^; calcd for [C_13_H_10_N_2_O_2_]^+^, 226.07423.

##### 5-[(*E*)-2-(3-Methoxyphenyl)ethenyl]-3-(prop-2-yn-1-yl)-1,3,4-oxadiazol-2(3*H*)-one (**4d**)

Chromatography: hexane to hexane/EtOAc 85:15. White solid (68% yield) of mp 92–94 °C. ^1^H NMR (500 MHz, MeOD): δ 7.34 (d, *J* = 16.4 Hz, 1H, H_α_), 7.30 (t, *J* = 8.0 Hz, 1H, H_5_), 7.17 (d, *J* = 8.0 Hz, 1H, H_6_), 7.15 (br s, 1H, H_2_), 6.95 (dd, *J* = 8.0, 2.5 Hz, 1H, H_4_), 6.79 (d, *J* = 16.4 Hz, 1H, H_β_), 4.57 (d, *J* = 2.3 Hz, 2H, CH_2_), 3.83 (s, 3H, CH_3_), 2.86 (t, *J* = 2.5 Hz, 1H, ≡CH). ^13^C NMR (126 MHz, MeOD): δ 161.6 (C_6_), 155.1 (C_5′_), 154.0 (C_2′_), 139.5 (C_α_), 137.4 (C_1_), 131.0 (C_5_), 121.3 (C_6_), 116.9 (C_4_), 113.5 (C_2_), 111.2 (C_β_), 77.1 (≡C), 74.9 (≡CH), 55.8 (CH_3_), 36.4 (CH_2_). HPLC-MS (15:95-g.t.10 min) *t*_R_ 8.38 min, *m*/*z*: 257.17 [M + H]^+^; calcd for [C_14_H_12_N_2_O_3_+H]^+^, 257.26; purity: 97%. HRMS [ESI^+^] *m*/*z*: 256.08539 [M]^+^; calcd for [C_14_H_12_N_2_O_3_]^+^, 256.08479.

##### 5-[(*E*)-2-(4-Methoxyphenyl)ethenyl]-3-(prop-2-yn-1-yl)-1,3,4-oxadiazol-2(3*H*)-one (**4e**)

Chromatography: hexane to hexane/EtOAc 85:15. White solid (56% yield) of mp 140–142 °C. ^1^H NMR (500 MHz, DMSO-*d*_6_): δ 7.68 (d, *J* = 8.7 Hz, 2H, H_2,6_), 7.32 (d, *J* = 16.4 Hz, 1H, H_α_), 6.98 (d, *J* = 8.7 Hz, 2H, H_3,5_), 6.84 (d, *J* = 16.4 Hz, 1H, H_β_), 4.60 (d, *J* = 2.5 Hz, 1H, CH_2_), 3.80 (s, 3H, CH_3_), 3.47 (t, *J* = 2.5 Hz, 1H, ≡CH). ^13^C NMR (126 MHz, DMSO-*d*_6_): δ 160.7 (C_4_), 153.4 (C_5′_), 151.9 (C_2′_), 137.6 (C_α_), 129.5 (C_2,6_), 127.2 (C_1_), 114.4 (C_3,5_), 107.6 (C_β_), 77.1 (≡C), 76.1 (≡CH), 55.3 (CH_3_), 35.3 (CH_2_). HPLC-MS (15:95-g.t.10 min) *t*_R_ 8.05 min, *m*/*z*: 257.17 [M + H]^+^; calcd for [C_14_H_12_N_2_O_3_ +H]^+^, 257.26; purity: 97%. HRMS [ESI^+^] *m*/*z*: 256.08363 [M]^+^; calcd for [C_14_H_12_N_2_O_3_]^+^, 256.08479.

##### 5-[(*E*)-2-(3-Hydroxyphenyl)ethenyl]-3-(prop-2-yn-1-yl)-1,3,4-oxadiazol-2(3*H*)-one (**5l**)

Chromatography: hexane to hexane/EtOAc 75:25. White solid (44% yield) of mp 166–168 °C. ^1^H NMR (500 MHz, MeOD): δ 7.30 (d, *J* = 16.4 Hz, 1H, H_α_), 7.22 (t, *J* = 7.9 Hz, 1H, H_5_), 7.06 (dt, *J* = 7.9, 1.1 Hz, 1H, H_6_), 7.00 (t, *J* = 2.1 Hz, 1H, H_2_), 6.81 (ddd, *J* = 7.9, 2.1, 1.1 Hz, 1H, H_4_), 6.72 (d, *J* = 16.4 Hz, 1H, H_β_), 4.57 (s, 2H, CH_2_), 2.85 (s, 1H, ≡CH). ^13^C NMR (126 MHz, MeOD): δ 159.1 (C_3_), 155.1 (C_5′_), 154.0 (C_2′_), 139.7 (C_α_), 137.3 (C_1_), 131.0 (C_5_), 120.2 (C_6_), 118.2 (C_4_), 114.8 (C_2_), 110.7 (C_β_), 76.7(≡C), 74.6 (≡CH), 36.4 (CH_2_). HPLC-MS (15:95-g.t.10 min) *t*_R_ 3.58 min, *m*/*z*: 241.29 [M – H]^−^; calcd for [C_13_H_10_N_2_O_3_ – H]^−^, 241.23; purity > 99%. HRMS [ESI^+^] *m*/*z*: 242.06867 [M]^+^; calcd for [C_13_H_10_N_2_O_3_]^+^, 242.06914.

#### General Procedure for the Synthesis of Propargyl Amides

Under a N_2_ atmosphere, acids (1 equiv) in anhydrous ACN (15 mL/mmol) were activated with HOBt (1.2 equiv), EDC·HCl (1.2 equiv), and DMAP (0.12 equiv) at rt for a period between 30 and 180 min. Propargylamine (1.2 equiv) was added, the mixture was left at rt for 15 min, and then, H_2_O was added. The crude was extracted with DCM (×3) and washed with saturated NaHCO_3_ (aq). The organic layer was dried over MgSO_4_, filtered, and evaporated to dryness under reduced pressure, obtaining the desired propargyl amides that were purified by flash chromatography.

##### (2*E*)-3-(3-Cyanophenyl)-*N*-(prop-2-yn-1-yl)prop-2-enamide (**6b**)

Chromatography: hexane to hexane/EtOAc 1:1. White solid (57% yield) of mp 172–173 °C. ^1^H NMR (500 MHz, MeOD): δ 7.96 (t, *J* = 1.6 Hz, 1H, H_2_), 7.87 (dt, *J* = 7.9, 1.6 Hz, 1H, H_6_), 7.73 (dt, *J* = 7.9, 1.6 Hz, 1H, H_4_), 7.60 (t, *J* = 7.9 Hz, 1H, H_5_), 7.57 (d, *J* = 15.8 Hz, 1H, H_α_), 6.69 (d, *J* = 15.8 Hz, 1H, H_β_), 4.09 (d, *J* = 2.5 Hz, 2H, CH_2_), 2.64 (t, *J* = 2.5 Hz, 1H, ≡CH). ^13^C NMR (126 MHz, MeOD): δ 167.3 (CO), 139.8 (C_α_), 137.7 (C_1_), 134.0 (C_4_), 133.2 (C_6_), 132.2 (C_2_), 131.1 (C_5_), 124.0 (C_β_), 119.2 (CN), 114.3 (C_3_), 80.3 (≡C), 72.5 (≡CH), 29.7 (CH_2_). HPLC-MS (15:95-g.t.10 min) *t*_R_ 5.38 min, *m*/*z*: 211.25 [M + H]^+^; calcd for [C_13_H_10_N_2_O + H]^+^, 211.24. HRMS [ESI^+^] *m*/*z*: 210.07991 [M]^+^; calcd for [C_13_H_10_N_2_O]^+^, 210.07931.

##### (2*E*)-3-(3-Methoxyphenyl)-*N*-(prop-2-yn-1-yl)prop-2-enamide (**6d**)^88^

Chromatography: hexane to hexane/EtOAc 6:4. White solid (83% yield) of mp 105–106 °C. ^1^H NMR (500 MHz, MeOD): δ 7.52 (d, *J* = 15.8 Hz, 1H, H_α_), 7.29 (t, *J* = 7.9 Hz, 1H, H_5_), 7.13 (d, *J* = 7.9 Hz, 1H, H_6_), 7.09 (dd, *J* = 2.6, 1.0 Hz, 1H, H_2_), 6.94 (ddd, *J* = 7.9, 2.6, 1.0 Hz, 1H, H_4_), 6.58 (d, *J* = 15.8 Hz, 1H, H_β_), 4.08 (d, *J* = 2.6 Hz, 2H, CH_2_), 3.81 (s, 3H, CH_3_), 2.62 (t, *J* = 2.6 Hz, 1H, ≡CH). ^13^C NMR (126 MHz, MeOD): δ 168.1 (CO), 161.5 (C_3_), 142.3 (C_α_), 137.5 (C_1_), 130.9 (C_5_), 121.5 (C_β_), 121.4 (C_6_), 116.6 (C_4_), 113.9 (C_2_), 80.4 (≡C), 72.4 (≡CH), 55.7 (CH_3_), 29.6 (CH_2_). HPLC-MS (15:95-g.t.10 min) *t*_R_ 6.10 min, *m*/*z*: 215.99 [M + H]^+^; calcd for [C_13_H_13_NO_2_ +H]^+^, 216.25; purity: 98%. HRMS [ESI^+^] *m*/*z*: 215.09483 [M]^+^; calcd for [C_13_H_13_NO_2_]^+^, 215.09463.

##### (2*E*)-3-(4-Methoxyphenyl)-*N*-(prop-2-yn-1-yl)prop-2-enamide (**6e**)

Chromatography: hexane to hexane/EtOAc 6:4. White solid (79% yield) of mp 123–125 (ref (89): 122.6–123.5 °C). ^1^H NMR (500 MHz, MeOD): δ 7.53–7.49 (m, 3H, H_α,2,6_), 6.94 (d, *J* = 8.8 Hz, 2H, H_3,5_), 6.45 (d, *J* = 15.8 Hz, 1H, H_β_), 4.07 (d, *J* = 2.6 Hz, 2H, CH_2_), 3.82 (s, 3H, CH_3_), 2.61 (t, *J* = 2.6 Hz, 1H, ≡CH). ^13^C NMR (126 MHz, MeOD): δ 168.6 (CO), 162.7 (C_4_), 142.1 (C_α_), 130.5 (C_2,6_), 128.7 (C_1_), 118.6 (C_β_), 115.3 (C_3,5_), 80.6 (≡C), 72.3 (≡CH), 55.8 (CH_3_), 29.6 (CH_2_). HPLC-MS (15:95-g.t.10 min) *t*_R_ 5.91 min, *m*/*z*: 216.23 [M + H]^+^; calcd for [C_13_H_13_NO_2_ +H]^+^, 216.25. HRMS [ESI^+^] *m*/*z*: 215.0946 [M]^+^; calcd for [C_13_H_13_NO_2_]^+^, 215.09463.

##### (2*E*)-3-(3-Hydroxyphenyl)-*N*-(prop-2-yn-1-yl)prop-2-enamide (**6l**)

Chromatography: hexane to hexane/EtOAc 1:1. White solid (80% yield) of mp 158–160 °C. ^1^H NMR (500 MHz, DMSO-*d*_6_): δ 9.58 (s, 1H, OH), 8.53 (t, *J* = 5.5 Hz, 1H, NH), 7.36 (d, *J* = 15.8 Hz, 1H, H_α_), 7.20 (t, *J* = 7.9 Hz, 1H, H_5_), 6.98 (d, *J* = 7.9 Hz, 1H, H_6_), 6.93 (t, *J* = 1.8 Hz, 1H, H_2_), 6.78 (dd, *J* = 7.9, 1.8 Hz, 1H, H_4_), 6.53 (d, *J* = 15.8 Hz, 1H, H_β_), 3.98 (dd, *J* = 5.5, 2.5 Hz, 2H, CH_2_), 3.15 (t, *J* = 2.5 Hz, 1H, ≡CH). ^13^C NMR (126 MHz, DMSO-*d*_6_): δ 164.7 (CO), 157.7 (C_3_), 139.5 (C_α_), 136.0 (C_1_), 129.9 (C_5_), 121.2 (C_β_), 118.8 (C_6_), 116.8 (C_4_), 113.7 (C_2_), 81.0 (≡C), 73.2 (≡CH), 28.0 (CH_2_). HPLC-MS (15:95-g.t.10 min) *t*_R_ 3.55 min, *m*/*z*: 202.22 [M + H]^+^; calcd for [C_12_H_11_NO_2_ + H]^+^, 202.23; purity: 95%. HRMS [ESI^+^] *m*/*z*: 201.07982 [M]^+^; calcd for [C_12_H_11_NO_2_]^+^, 201.07898.

##### (2*E*)-3-(4-Hydroxyphenyl)-*N*-(prop-2-yn-1-yl)prop-2-enamide (**6m**)

Chromatography: hexane to hexane/EtOAc 7:3. White solid (76% yield) of mp 125–128 °C. ^1^H NMR (500 MHz, MeOD): δ 7.48 (d, *J* = 15.7 Hz, 1H, H_α_), 7.42 (d, *J* = 8.7 Hz, 2H, H_2,6_), 6.79 (d, *J* = 8.7 Hz, 2H, H_3,5_), 6.40 (d, *J* = 15.7 Hz, 1H, H_β_), 4.07 (d, *J* = 2.6 Hz, 2H, CH_2_), 2.61 (t, *J* = 2.6 Hz, 1H, ≡CH). ^13^C NMR (126 MHz, MeOD): δ 168.8 (CO), 160.7 (C_4_), 142.5 (C_α_), 130.7 (C_2,6_), 127.6 (C_1_), 117.7 (C_β_), 116.7 (C_3,5_), 80.6 (≡C), 72.2 (≡CH), 29.5 (CH_2_). HPLC-MS (15:95-g.t.10 min) *t*_R_ 2.38 min, *m*/*z*: 202.22 [M + H]^+^; calcd for [C_12_H_11_NO_2_+H]^+^, 202.23. HRMS [ESI^+^] *m*/*z*: 201.07891 [M]^+^; calcd for [C_12_H_11_NO_2_]^+^, 201.07898.

##### (2*E*)-3-(4-Hydroxy-3-methoxyphenyl)-*N*-(prop-2-yn-1-yl)prop-2-enamide (**6n**)

Chromatography: hexane to hexane/EtOAc 1:1. White solid (86% yield) of mp 131–132 °C (ref (90): 128–129 °C). ^1^H NMR (500 MHz, DMSO-*d*_6_): δ 9.47 (br s, 1H, OH), 8.38 (br s, 1H, NH), 7.35 (d, *J* = 15.7 Hz, 1H, H_α_), 7.12 (s, 1H, H_2_), 7.00 (d, *J* = 8.1 Hz, 1H, H_6_), 6.79 (d, *J* = 8.1 Hz, 1H, H_5_), 6.44 (d, *J* = 15.7 Hz, 1H, H_β_), 3.97 (br s, 2H, CH_2_), 3.80 (s, 3H, CH_3_), 3.12 (br s, 1H, ≡CH). ^13^C NMR (126 MHz, DMSO-*d*_6_): δ 165.2 (CO), 148.4 (C_4_), 147.8 (C_3_), 139.8 (C_α_), 126.2 (C_1_), 121.6 (C_6_), 118.1 (C_β_), 115.7 (C_5_), 111.0 (C_2_), 81.2 (≡C), 73.0 (≡CH), 55.6 (CH_3_), 28.0 (CH_2_). HPLC-MS (15:95-g.t.10 min) *t*_R_ 3.00 min, *m*/*z*: 232.10 [M + H]^+^; calcd for [C_13_H_13_NO_3_ + H]^+^, 232.25. HRMS [ESI^+^] *m*/*z*: 231.08981 [M]^+^; calcd for [C_13_H_13_NO_2_]^+^, 231.08954.

##### (2*E*)-3-(3,4-Dihydroxyphenyl)-*N*-(prop-2-yn-1-yl)prop-2-enamide (**6o**)

Chromatography: hexane to hexane/EtOAc 55:45. White solid (56% yield) of mp 169–171 °C (ref (91) 169–170 °C). ^1^H NMR (500 MHz, MeOD): δ 7.42 (d, *J* = 15.7 Hz, 1H, H_α_), 7.01 (d, *J* = 2.1 Hz, 1H, H_2_), 6.91 (dd, *J* = 8.2, 2.1 Hz, 1H, H_6_), 6.77 (d, *J* = 8.2 Hz, 1H, H_5_), 6.36 (d, *J* = 15.7 Hz, 1H, H_β_), 4.06 (d, *J* = 2.6 Hz, 2H, CH_2_), 2.59 (t, *J* = 2.6 Hz, 1H, ≡CH). ^13^C NMR (126 MHz, MeOD): δ 168.8 (CO), 148.8 (C_4_), 146.7 (C_3_), 142.9 (C_α_), 128.1 (C_1_), 122.2 (C_6_), 117.7 (C_β_), 116.4 (C_5_), 115.1 (C_2_), 80.6 (≡C), 72.2 (≡CH), 29.5 (CH_2_). HPLC-MS (5:95-g.t.10 min) *t*_R_ 4.93 min, *m*/*z*: 218.25 [M + H]^+^; calcd for [C_12_H_11_NO_3_ + H]^+^, 218.22; purity: 98%. HRMS [ESI^+^] *m*/*z*: 217.07318 [M]^+^; calcd for [C_12_H_11_NO_3_]^+^, 217.07389.

##### 2-Methoxy-4-{(1*E*)-3-[(prop-2-yn-1-yl)amino]prop-1-en-1-yl}phenol (**8n**)

To a solution of commercial ferulic aldehyde **7n** (100 mg, 0.56 mmol) and molecular sieves of 4 Å in 3 mL (6 mL/mmol) of dry THF, propargylamine (179 μL, 2.81 mmol, 5 equiv) was added. The mixture was stirred at rt overnight, and then, it was filtered, washed with THF several times, and evaporated. The crude imine was not isolated but dissolved in 3.5 mL of MeOH, treated with NaBH_4_ (23 mg, 0.62, 1.1 equiv) at 0 °C, and stirred at rt for 30 min. The solvent was removed, and the residue was dissolved in EtOAc, extracted, washed with H_2_O and brine, dried over MgSO_4_, filtered, and evaporated to dryness. The crude was purified by flash chromatography in EtOAc/TEA 95:5 to obtain **8n** as a yellow pale solid in 62% yield (76 mg, 0.35 mmol) of mp 76–78 °C. ^1^H NMR (500 MHz, DMSO-*d*_6_): δ 8.98 (br s, 1H), 7.63 (br s, 1H), 6.99 (d, *J* = 2.0 Hz, 1H, H_2_), 6.78 (dd, *J* = 8.1, 2.0 Hz, 1H, H_6_), 6.70 (d, *J* = 8.1 Hz, 1H, H_5_), 6.39 (d, *J* = 16.0 Hz, 1H, H_α_), 6.08 (dt, *J* = 16.0, 6.3 Hz, 1H, H_β_), 3.77 (s, 3H, CH_3_), 3.32–3.29 (m, 4H, H_γ_, CH_2_C≡), 3.06 (t, *J* = 2.4 Hz, 1H, ≡CH). ^13^C NMR (126 MHz, DMSO-*d*_6_): δ 147.7 (C_3_), 146.1 (C_4_), 130.7 (C_α_), 128.5 (C_1_), 125.3 (C_β_), 119.3 (C_6_), 115.4 (C_5_), 109.6 (C_2_), 83.0 (≡C), 73.6 (≡CH), 55.5 (CH_3_), 49.6 (C_γ_), 36.6 (CH_2_C≡). HPLC-MS (2:30-g.t.10 min) *t*_R_ 1.57 min, *m*/*z*: 218.10 [M + H]^+^; calcd for [C_13_H_15_NO_2_+H]^+^, 218.27. HRMS [ESI^+^] *m*/*z*: 217.11067 [M]^+^; calcd for [C_13_H_15_NO_2_]^+^, 217.11028.

### Systems Biology Approach. Compilation of Pieces of Evidence from Public Repositories

The relevance of NFE2L2, NQO2, and MAOB as potential targets for a set of NDs has been assessed by accessing pertinent records in Open Targets^23^ and ChEMBL.^45^

The Open Targets DB is a comprehensive tool that integrates different types of data from multiple publicly available repositories. Relevant sources include literature reports, animal models and phenotypes, gene expression, clinical trials, and allelic variants associated with diseases or phenotypes. The platform generates scores and counts the amount of evidence to support associations between target genes and diseases for each type and data source, as explained in the documentation section of the website.^23^ The Open Targets DB was queried to retrieve all the NFE2L2, NQO2, and MAOB matches with a group of NDs identified by a set of keywords, thus obtaining a general association score, the evidence count, and the sources used for building the scores. Genes expressed downstream NFE2L2 have also been incorporated into the search terms. Queries returned results from Europe PMC, PhenoDigm, Expression Atlas, Open Targets Genetics portal, and ChEMBL.

Once the overall scores and data sources for the existing targets and diseases have been obtained, specific queries are applied upon the identified sources to retrieve information details corresponding to each specific source. Queries return direct and indirect pieces of evidence. For the Open Targets DB, the direct pieces of evidence are disease–target association items, where the specific names of genes and diseases are explicit in the body of the evidence. However, if the disease is not in the body of the item but appears in an ontologically related one, as might happen with irritable bowel syndrome and Crohn’s disease, Open Targets classifies the evidence as indirect.

Europe PMC (Europe PubMed Central) accesses a worldwide collection of life science publications and preprints from trusted sources, identifying text co-occurrences between targets and diseases using deep learning algorithms. It provides an assessment of the convergence confidence and number of evidence by the aggregation of all unique co-occurrences for a particular target and disease for each article.^23^ Using NDs’ keywords, the NFE2L2, NQO2, and MAOB queries in this collection retrieved the associations found in PubMed, listing the following data: target and symbol name, disease name, score, section, mined text, PubMed ID number, article title, abstract, date, journal, keywords, and authors’ name (data collected in Supporting Information 2).

PhenoDigm is an algorithm created by the Wellcome Sanger Institute that establishes gene–disease relationships based on information from KO animal models and subsequently maps the mice phenotypes to the corresponding human diseases.^92^ The queries launched in PhenoDigm retrieved the usual target–disease pair, the name and ID of the animal model, the genetic background, and the allelic compositions regarding the queried gene and the phenotypes carried by the animal. The ID can be used to access phenotype details through the Mouse Genome Informatics (MGI) DB, along with the bibliographic references supporting the associations.^93^

Expression Atlas (EMBL-EBI) provides gene–disease association scores and evidence count from RNA expression data, where the genes are differentially expressed in disease versus control samples.^94^ Consultation of Expression Atlas collection provides target and disease names, IDs and descriptors alongside the levels of expression, association score, fold change in the percentile rank, samples to contrast, a description of each expression study, a PubMed ID to identify where the info has been published, and assay ID, which can be used to access to whole assay results stored in the ArrayExpress section of the EMBL-EBI website.^95^

Open Targets Genetics Portal sets gene–phenotype relations from genome-wide association studies identifying allelic variants associated to pathological states by the application of statistical genetics and machine learning.^96^ Queries upon this collection retrieve the usual gene and disease identifiers including the allelic variant, the association score, the PubMed ID when applicable, and the study ID to access the whole data sets in the Open Targets Genetics Portal repository.

ChEMBL data stored in the Open Targets DB include information regarding the mechanisms of action of approved drugs or substances submitted to clinical trials.^45^ ChEMBL is a manually curated DB that gathers information from more than 15 million experimental records carried out with 2 million molecules in 1.4 million different phenotypic or target-based assays. It includes biological properties (protein, cell lines, tissues, and organisms) as molecular and assay descriptors. It annotates the recorded interactions of drugs with gene products, type of study, phase, status, and the corresponding association score based on the achieved phase. Most annotated interactions have a value coming from assays with very different pharmacological evaluation EC_50_s or IC_50_s and their −log *P* transformation, in any kind of molar or weigh/volume definition, in percent inhibition, and many others. ChEMBL provides about 3 million records normalized to the pchembl score as a result of the log transformation of EC_50_s or IC_50_s in the molar scale. However, the DrTarget DB^97^ contains a normalized score for 15 million ChEMBL records, which largely increases the capacity of exploitation of ChEMBL contents. This ChEMBL score is the magnitude used to study the interactions of NFE2L2, NQO2, and MAOB active compounds recorded in the ChEMBL DB either with protein-based or with neurodegeneration assays and typically has a range of values between 1 and 10 with an activity threshold value around 4, which coincides with the intuitive range of activity for a typical pXC_50_ value. ChEMBL assays have been identified by specific keywords and classified into different bins: neurodegeneration, Alzheimer, Parkinson, oxidative assays, neuronal plasticity, memory and cognition, motor activity, and cytoprotection. Besides, to better identify protein aggregation studies, Alzheimer and Parkinson bins have been subclassified in tau, amyloid precursor protein, and synuclein bins. Then, we have studied how the active compounds in NFE2L2, NQO2, and MAOB behave in aggregated neurodegeneration assays at different assay bin levels by consulting the number of target active molecules, the score on the target, and the aggregated score on the assay or assay bin.

### Biological Studies

#### Inhibition of Human Monoamine Oxidases (hMAO-A and hMAO-B)

Assays were performed following the general procedure previously described.^98^ In brief, the tested compounds and adequate amounts of recombinant hMAO-A or hMAO-B (Sigma-Aldrich Chemistry S.A., Alcobendas, Spain) required and adjusted to oxidize 165 pmol of *p*-tyramine/min in the control group were incubated at 27 °C for 30 min in a flat-black-bottom 96-well microtest plate (BD Biosciences, Franklin Lakes, NJ) placed in a dark fluorimeter chamber. (*E*)-Resveratrol, iproniazid, and moclobemide were also assayed. The reaction was started by adding 200 mM Amplex Red reagent (Molecular Probes, Inc., Eugene, OR), 1 U/mL horseradish peroxidase, and 1 mM *p*-tyramine, and the production of resorufin was quantified at 27 °C using a FLUOstar Optima reader (BMG LABTECH GmbH, Offenburg, Germany) based on the fluorescence generated (excitation, 545 nm; emission, 590 nm). The specific fluorescence emission was calculated after subtracting the background activity, which was determined from wells containing all components except the hMAO isoforms, which were replaced with PBS.

#### MAO-B Reversibility Assays

The assays were carried out in Costar 96-well black opaque plates at a final volume of 200 μL/well. The compounds were preincubated with the monoamine oxidase B (hMAO-B; 0.135 U/mL) for 0, 15 or 30 min at 37 °C at a volume of 100 μL. Then, 100 μL of the starting solution was added to start the enzymatic reaction. The starting solution was prepared in 25 mM sodium phosphate at pH 7.4 with 1 mM of MAO substrate tyramine, 0.04 U/mL horseradish peroxidase (HRP), and 25 μM Amplex UltraRed reagent. The coupled enzymatic reaction gives resorufin as a final fluorescent product. Fluorescence production was measured at 530/590 nm (excitation/emission) for 30 min in a FluoStar Optima reader (BMG Labtech). Rasagiline (MAO-B selective inhibitor) was used as a reference of irreversible inhibition for comparative purposes. Each compound was incubated at 1.8 × IC_50_ μM final concentration for achieving sufficient MAO-B inhibition.

#### Molecular Docking on MAO-B

Docking was performed with AutoDock Vina.^99^ Prior to docking calculation, ligand states were generated at pH 7.4 using Epik, and finally, they were prepared and minimized using the LigPrep module of Schrodinger software.^100^ The **6fw0** PDB-ID structure was prepared and minimized using the Protein Preparation Wizard tool in Maestro using the Optimized Potentials for Liquid Simulations 3 (OPLS3) force field for protein and ligand preparation.^101^ The box for docking calculations was placed in the geometric center of the ligand *N*-(3-chlorophenyl)-4-oxo-4*H*-chromene-3-carboxamide crystallized in the **6fw0** structure, and the dimensions were chosen so as to cover the bipartite MAO-B cavity (both entrance and substrate cavities). Best poses were visually inspected and ranked by energy. All images were constructed using PyMOL software.^102^

#### Luciferase Activity: NRF2 Induction

AREc32 cells were plated in 96-well white plates (2 × 10^4^ cells/well). After growing the cells for 24 h, they were treated with increasing concentrations of the tested compound (0.3, 3, 10, 30, and 60 μM) in duplicate for 24 h. (*E*)-Resveratrol and sulforaphane were also evaluated under the same conditions. The AREc32 cells constitutively express the plasmid pGL-8xARE that implements eight copies of the EpRE sequences, followed by the luciferase reporter gene. Therefore, NRF2 induction is related to the activation of EpRE sequences, expressing luciferase at the same extent as EpRE sequences are activated. The Luciferase Assay System (Promega E1500) was used according to the provider’s protocol, and luminescence was quantified in an Orion II microplate luminometer (Berthold, Germany). Fold induction of luciferase activity was normalized to basal conditions. Data are expressed as CD values, expressing the concentration required to double the luciferase activity. CD values were calculated from dose–response curves generated from the fold induction of control conditions *versus* inducer concentration and fitted by nonlinear regression and data interpolated to twofold induction concentration.^68^

#### ORAC Assay

The ORAC method was followed using a Polarstar Galaxy plate reader (BMG LABTECH GmbH, Offenburg, Germany) with 485-P excitation and 520-P emission filters.^55,69^ The equipment was controlled by the Fluorostar Galaxy software (version 4.11-0) for fluorescence measurement. 2,2′-Azobis-(amidinopropane) dihydrochloride (AAPH), trolox, and fluorescein (FL) were purchased from Sigma-Aldrich. The reaction was carried out in a 75 mM phosphate buffer (pH 7.4), and the final reaction mixture was 200 μL. Antioxidant (20 μL) and FL (120 μL; 70 mM, final concentration) solutions were placed in a black 96-well microplate (96F untreated, Nunc). The mixture was preincubated at 37 °C for 15 min, and then, AAPH solution (60 μL, 12 mM, final concentration) was added rapidly using a multichannel pipette. The microplate was immediately placed in the reader and the fluorescence recorded every minute for 80 min. The microplate was automatically shaken prior to each reading. The samples were measured at eight different concentrations (0.1–1 μM). A blank (FL + AAPH in phosphate buffer) instead of the sample solution and eight calibration solutions using trolox (1–8 μM) were also carried out in each assay. All the reaction mixtures were prepared in duplicate, and at least three independent assays were performed for each sample. Raw data were exported from the Fluostar Galaxy Software to an Excel sheet for further calculations. Antioxidant curves (fluorescence *vs* time) were first normalized to the curve of the blank corresponding to the same assay, and the area under the curve (AUC) of fluorescence decay was calculated. The net AUC corresponding to a sample was calculated by subtracting the AUC corresponding to the blank. Regression equations between the net AUC and antioxidant concentration were calculated for all the samples. ORAC-FL values were expressed as trolox equivalents by using the standard curve calculated for each assay, where the ORAC-FL value of trolox was taken as 1.0.

#### Assays in Melatonin Receptors: hMT_1_R, hMT_2_R, and QR2

Assays in MT_1_R and MT_2_R were carried out using human receptors that were stably transfected in Chinese hamster ovary cells (https://www.eurofins.fr, catalog refs. 1538 and 1687). The QR2 experiments were performed in membrane homogenates of hamster brains (https://www.eurofins.fr, catalog ref. 0088). In all cases, the displacement of 2-[^125^I]iodomelatonin was measured in the absence or presence of the tested compound and nonspecific binding was determined with melatonin, following described protocols.^103−105^ First, radioligand displacement was measured at a fixed compound concentration (10 μM) in each receptor. Then, IC_50_s were calculated only for compounds with a radioligand displacement exceeding 80% using a range of five different concentrations of the compound in three independent experiments (Supporting Information, Figure S5). (*E*)-Resveratrol and melatonin were tested for comparative purposes.

#### In Vitro Blood–Brain Barrier Permeation Assay (PAMPA-BBB)

Prediction of brain penetration was evaluated using the PAMPA-BBB assay in a similar manner as previously described.^55,70−73^ Pipetting was performed using a semiautomatic robot (CyBi-SELMA) and UV reading using a microplate spectrophotometer (Multiskan Spectrum, Thermo Electron Co.). Commercial drugs, PBS solution at pH 7.4, and dodecane were purchased from Sigma-Aldrich, Acros, and Fluka, respectively. Millex filter units (PVDF membrane, diameter 25 mm, pore size 0.45 μm) were acquired from Millipore. The porcine brain lipid (PBL) was obtained from Avanti Polar Lipids. The donor microplate was a 96-well filter plate (PVDF membrane, pore size 0.45 μm), and the acceptor microplate was an indented 96-well plate, both from Millipore. The acceptor 96-well microplate was filled with 200 μL of PBS/EtOH (70:30), and the filter surface of the donor microplate was impregnated with 4 μL of PBL in dodecane (20 mg mL^–1^). The compounds were dissolved in PBS/EtOH (70:30) at 100 μg mL^–1^, filtered through a Millex filter, and then added to the donor wells (200 μL). The donor filter plate was carefully put on the acceptor plate to form a sandwich, which was left undisturbed for 240 min at 25 °C. After incubation, the donor plate was carefully removed and the concentration of the compounds in the acceptor wells was determined by UV–vis spectroscopy. Every sample was analyzed at five wavelengths in four wells and at least in three independent runs, and the results are given as the mean ± SD. In each experiment, 11 quality control standards of known BBB permeability were included to validate and normalize the analysis set (Supporting Information, Table S2).

#### Molecular Docking on QR2

Molecular docking was performed using AutoDock4.^106^ First, ligand states were generated at pH 7.4 and minimized using OpenBabel 3.0.^107^ The crystal structure of QR2 in complex with resveratrol was obtained from PBD (ID: 4QOH), and the protein was prepared and minimized at pH 7.4 using the Protein Prepare tool from PlayMolecule.^108^ The grid in which the docking was performed had the center in the center of mass of resveratrol from the crystallized 4QOH protein and their dimensions chosen considering ligand size. About 200 simulations were done by each ligand and structure. The results were visually checked, grouped in clusters, and ranked by energy; selected clusters were the most populated. Images were visualized using PyMOL software.^102^

#### In Vitro Study of Glutathione Conjugation by LC-IT-MS Analysis

Glutathione (GSH) (1 mM, final concentration), compound **4e** (100 μM, final concentration), and 10 U of glutathione *S*-transferase (GST) were added to PBS (10 mM, pH 6.5) at a final volume of 500 μL. The reaction was maintained at 37 °C for 1 h. As a control, the nonenzymatic reaction mixture (mixture lacking GST) was incubated for 1 h at 37 °C. The reactions were stopped by adding 100 μL of 20% trifluoroacetic acid. The samples were prepared for analysis by adding 400 μL of a 50:50 mixture of MeOH/CH_3_CN. The reaction mixtures were analyzed using an API QSTAR pulsar I LC–MS/MS system (Applied Biosystems, Madrid, Spain) equipped with an electrospray ionization source and connected to a LC system 1100 series (Agilent Technologies, Madrid, Spain). The sample components were separated in a 150 × 2.1 mm Beta Basic-18 C18 column using a linear gradient mobile phase of 80% water with 0.1% formic acid and 20% acetonitrile. The LC-IT-MS was operated in the positive ion mode.

#### Neurogenic Studies

Adult male C57BL/6 mice (3 months old) were used in order to determine neurogenesis activity. NSCs were isolated from the SGZ of the dentate gyrus of the hippocampus of adult mice and cultured as NS, as previously described.^109,110^ After treatment of NS with the corresponding compounds at 10 μM, the expression of neuronal markers was analyzed by immunocytochemistry according to published protocols^109^ using two well-known neurogenesis-associated markers: Tuj1 to early stages of neurogenesis and MAP-2 to late neuronal maturation. A rabbit anti-β-III-tubulin (TuJ clone; Abcam) polyclonal antibody coupled to an Alexa-488-fluor-labeled secondary antibody (Molecular Probes) and a mouse anti-MAP-2 (Sigma) monoclonal antibody coupled to an Alexa-546-fluor-labeled secondary antibody (Molecular Probes) were used. DAPI staining was used as a nuclear marker. Fluorescent representative images were acquired with a Nikon fluorescence microscope 90i coupled to a digital camera Qi. The microscope configuration was adjusted to produce the optimum signal-to-noise ratio. The number of cells expressing β-III-tubulin (TuJ-1 clone) or MAP-2 leaving the neurosphere core were counted as previously described.^111^ Quantification was undertaken using the image analySIS software (Soft Imaging System Corp., Münster, Germany) and normalized to total nuclei (DAPI-stained). The intensity of immunostaining of neurites from TuJ1-labeled cells and the number of MAP-2-positive cells were estimated from nine neurospheres per condition over three independent experiments. The outgrowth of the neurosphere cells was examined under a phase-contrast microscope and the farthest distance of cell migration was calculated from the edge of the sphere. At least 10 plated neurospheres per treatment were analyzed.

#### Neuroprotection Studies in Models Related to AD

##### SH-SY5Y Cell Culture

The SH-SY5Y human neuroblastoma cell line (ATCC, Virginia, EEUU) was grown in a modified minimum essential medium (MEM) (Invitrogen, Spain) [4.765 g/L MEM; 2.5% minimum essential medium–nonessential amino acids (Invitrogen, Madrid, Spain); Ham’s F12 nutrients mix (Thermo Fisher, Massachusetts, EEUU); 0.5 nM sodium pyruvate (Sigma-Aldrich, Spain); 2 g/L of NaHCO3 (PanReac, Barcelona, Spain); 10% (v/v) filtrated fetal bovine serum (FBS) (Gibco, Invitrogen, Spain); and 100 U/mL of penicillin/streptomycin (Invitrogen, Spain)]. The cells were cultured in flasks (Corning, EEUU) until they reached 80% confluence and subcultured using 0.25% EDTA–trypsin (Thermo Fisher, EEUU) for 5 min. Thereafter, they were centrifuged at 700 rpm for 7 min. The cells from the 3rd to 12th passage were seeded at a density of 80,000 cells/well in 96-well plates and were used to assess the properties of our compounds. The cells were maintained at 37 °C under a humidified atmosphere (5% CO_2_ and 95% relative humidity).

##### Rat Primary Neuronal Culture

Rat cortical neurons were cultured from fetuses obtained from an 18 day pregnant rat. The fetuses were extracted by a cesarean section. Immediately, they were beheaded and their encephalon placed in saline phosphate buffer [NaCl 137 mM, KCl 3 mM, Na_2_HPO_4_ 10 nM, KH_2_PO_4_ 2 mM, bovine serum albumin (BSA) 4 mM, glucose 1.5 mM; pH 7.4]. Thereafter, their brain cortices were extracted, homogenized mechanically, and centrifuged at 800 rpm (Kubota 5100, PACISA) for 10 min. Later, they were resuspended in Dulbecco’s modified Eagle medium (DMEM)/F-12 (Gibco) supplemented with 20% FBS and 0.005% penicillin/streptomycin. Neuronal primary cell cultures were seeded in poly-d-lysine treated wells. For this, the plates were pretreated for a minimum of 2 h under UV light with poly-d-lysine (Sigma-Aldrich, Spain). Then, poly-d-lysine was washed three times with sterile H_2_O and primary cortical neurons seeded at a density of 60,000 cells/well in 96-well plates. After 2 h of incubation, a medium replacement was performed with a neurobasal medium (Gibco, Invitrogen, Spain) supplemented with 10% FBS, 0.005% penicillin/streptomycin, and B-27 (Invitrogen, Spain). The cells were cultured for 7–10 days at 37 °C, 5% CO_2_, and 95% RH. Neuroprotection experiments were performed using a NB medium supplemented with B-27 without antioxidants (AOs) (Invitrogen, Spain).

##### Acute Hippocampal Slice Model

3–4 months old mice (C57BL/6j) were sacrificed, and hippocampi were carefully dissected in a dissection solution (120 mM NaCl; 2 mM KCl; 26 mM NaHCO_3_; 1.18 mM KH_2_PO_4_; 10 mM MgSO_4_; 0.5 mM CaCl_2_; 11 mM glucose; 200 mM sucrose at pH 7.4). 250 μm-thick slices were cut using a McIlwain Tissue Chopper (Cavey Laboratory Engineering, United Kingdom) and stabilized for 45 min at 34 °C with 95% O_2_ and 5% CO_2_ in a preincubation buffer (120 mM NaCl; 2 mM KCl; 26 mM NaHCO_3_; 1.18 mM KH_2_PO_4_; 10 mM MgSO_4_; 0.5 mM CaCl_2_ and 11 mM glucose). Thereafter, the slices were incubated for 6 h at 37 °C in humidified 5% CO_2_/95% air with or without OA alone (OA: 1 μm, Sigma-Aldrich) or combined with derivative **4e** (1 μm) in the control buffer (120 mM NaCl; 2 mM KCl; 26 mM NaHCO_3_; 1.18 mM KH_2_PO_4_; 10 mM MgSO_4_; 2 mM CaCl_2_; 11 mM glucose) and DMEM (Invitrogen, Spain) (1:1 ratio). Once the treatment was ended, the cell viability was measured by the MTT method, cell death with the fluorescent probe PI (1 μg/mL), and ROS production with the fluorescent probe H_2_DCFDA (10 μL/mL).

##### Organotypic Hippocampal Culture

6–10 days old mice (C57BL/6j) were sacrificed, and hippocampi were carefully removed in Hank’s balanced salt solution (HBBS). 300 μm slices were cut and placed in 0.4 μm culture inserts (Merck-Millipore, Germany) and stabilized for 24 h at 37 °C in neurobasal media (Gibco, Invitrogen) supplemented with 10% FBS (Gibco, Invitrogen). After stabilization, the medium was carefully removed and replaced with a new medium containing the neurobasal mixture supplemented with B27 nutrients and antioxidants (B27 + AO, Invitrogen) and the slices were incubated for 72 h at 37 °C in humidified 5% CO_2_/95% air. Thereafter, treatment with the neurotoxic OA alone (10 nM) or combined with derivative **4e** (1 μM) was performed for 72 h at 37 °C (humidified 5% CO_2_/95% air) in the neurobasal medium supplemented with B27 without antioxidants (B27-AO, Invitrogen). Finally, cell death and ROS production were determined with the fluorescent probes PI (1 μg/mL) and H_2_DCFDA (10 μg/mL), respectively.

##### MTT Method for Cell Viability

After treatment, the SH-SY5Y cells, rat primary neuronal cultures, and hippocampal slices were incubated for 90 min with tetrazolium salt MTT (0.5 mg/mL) solution. This assay is based on the reduction of MTT (yellow salt) to purple insoluble formazan crystals by oxidoreductase enzymes from living cells. Thereafter, in order to solubilize the formazan crystal, the cells/slices were incubated for another 45 min in dimethyl sulfoxide (DMSO). Finally, absorbance was measured at 535 nm using a microplate reader (SPECTROstar NANO, BMG LABTECH). The basal absorbance was set to 100%, and the results were normalized to basal conditions.

##### Measurement of Cell Death and ROS Production with Fluorescent Dyes in Hippocampal Slices

After acute and chronic treatment with OA (acute hippocampal model and organotypic culture), the slices were incubated with PI (1 μg/mL) to measure cell death and H_2_DCFDA (10 μg/mL) to measure ROS production for 45 min in the control solution at 37 °C in humidified 5% CO_2_/95% air. Fluorescence from whole slices was measured as the mean intensity with an inverted Nikon eclipse T2000-U microscope (Nikon Instruments). The wavelengths of excitation and emission for PI and H_2_DCFDA were 530, 495 and 580, 520, respectively. The results were normalized to the basal condition, which was considered as 100%. Image analysis was performed using Fiji software.

##### Western Blotting

After acute treatment, the hippocampal slices were lysed in a cold AKT lysis buffer (137 mM NaCl, 20 mM NaF, 10% glycerol, 20 mM Tris–HCl, 1% Nonidet P-40, 1 μg/mL leupeptin, 1 mM phenylmethylsulfonylfluoride, 1 mM sodium pyrophosphate, and 1 mM Na_3_VO_4_, pH 7.5). The total protein in each sample was quantified using the Pierce BCA protein kit (Thermo Fisher). 20 μg of protein was resolved by sodium dodecyl sulfate polyacrylamide gel electrophoresis (SDS-PAGE) and transferred to Immobilon-P PVDF membranes (Millipore Corp.). The membranes were activated with methanol and blocked in 4% BSA for 2h at rt. The membranes were incubated overnight at 4 °C with the primary antibodies: anti-iNOS (1:1000, 610432, BD Transduction Laboratories), anti-p65 (1:1000, sc-372, Santa Cruz Biotechnology), anti-HO-1 (1:1000, ab68477, Abcam), and anti-β-actin (1:50000, A3854, Sigma-Aldrich). Afterward, the membranes were washed thrice and then incubated for 90 min with the appropriate peroxidase-conjugated secondary antibodies (1:10,000, Santa Cruz Biotechnology). The membranes were washed, incubated with ECL WB Kit (GE Healthcare, Amersham), and exposed using a ChemiDoc MP system (Bio-Rad Laboratories) for the visualization of the specific bands, which were then analyzed with Fiji software.

##### Statistics

For the biological results, data are represented as mean ± SEM. Multiple groups were compared using one-way analysis of variance test (one-way ANOVA), followed by Tukey’s posthoc test. Statistical significance was set at *p < 0.05, **p < 0.01, and ***p < 0.001. Data was analyzed using GraphPad Prism 8.02 software.

##### Ethics for Animals Used in Neurogenic and Neuroprotective Studies

All animal experimental procedures were previously approved by the Ethics Committees for Animal Experimentation following national normative (RD1386/2018) and international recommendations (Directive 2010/63 from the European Union). The animals were housed in a 12 h light/12 h dark with water and food *ad libitum*. Special care was taken to minimize animal suffering.

## Supporting Information Available

Data mining studies in the Open Targets DB, superposition studies of minimized structures of resveratrol and resveratrol-based MTDLs, theoretical and experimental p*K*_a_s, MAO-B binding mode elucidation by molecular docking studies, assays in the melatonin receptors *h*MT_2_R and QR2, in vitro CNS permeation of commercial drugs used as references in the PAMPA-BBB assay, drug-like property calculations, study of the potential union of **4e** to GSH, western blot analysis of **4e** in mouse hippocampal slices subjected to OA-induced toxicity, in silico metabolism prediction of **4e**, and spectroscopic data (HPLC-MS, ^1^H NMR, ^13^C NMR, HSQC, HMBC, and HRMS) of resveratrol-based MTDLs (PDF)
Full bibliographic data from the Open Targets DB supporting NFE2L2, NQO2, and MAO-B as targets for the NDs (XLSX)
Molecular formula string and data (CSV)
PDB data (ZIP)

## Abbreviations

AD: Alzheimer’s disease
ANOVA: analysis of variance
AREs: antioxidant response elements
Aβ: amyloid β-peptide
CD: concentration needed to duplicate the activity of the luciferase reporter
CNS: central nervous system
COSY: homonuclear correlation spectroscopy
DAPI: 4′,6-diamidino-2-phenylindole
Europe PMC: Europe PubMed Central
GSH: glutathione
GST: glutathione *S*-transferase
H_2_DCFDA: 2′,7′-dichlorodihydrofluorescein diacetate
HMBC: heteronuclear multiple bond correlation
HMOX-1: heme oxygenase 1
HSQC: heteronuclear single quantum correlation
iNOS: inducible nitric oxide synthase
MAOs: monoamine oxidases
MAP-2: microtubule-associated protein 2
MT_1_R and MT_2_R: G protein-coupled melatonin receptors
MTDLs: multitarget-directed ligands
MTT: 3-(4,5-dimethylthiazol-2-yl)-2,5-diphenyltetrazolium bromide
NDs: neurodegenerative diseases
NF-κB: nuclear factor kappa-light-chain-enhancer of activated B cells
NRF2 or NFE2L2: erythroid 2-related factor 2
NS: neurosphere
NSC: neural stem cell
OA: okadaic acid
ORAC: oxygen radical absorbance capacity
PAINS: pan-assay interference compounds
PAMPA-BBB: in vitro parallel artificial membrane permeability assay for the blood–brain barrier permeation
PD: Parkinson’s disease
PDB: Protein Data Bank
QR2 or NQO2: FAD-dependent quinone reductase-2
ROS: radical oxygen species
TuJ-1: human β-III-tubulin
