# Supplementary material for: Resveratrol-Based MTDLs to Stimulate Defensive and Regenerative Pathways and Block Early Events in Neurodegenerative Cascades
Source: J Med Chem. 2022 Mar 4;65(6):4727–51. doi: 10.1021/acs.jmedchem.1c01883 (PMC8958504; doi:10.1021/acs.jmedchem.1c01883)
Supplement: Supplementary file 1 — jm1c01883_si_001.pdf [file jm1c01883_si_001.pdf]

Supporting Information-1 for

Resveratrol-Based MTDLs to Stimulate Defensive and Regenerative Pathways and Block Early Events in Neurodegenerative Cascades

Clara Herrera-Arozamena,[1,2] Martín Estrada-Valencia,[1] Patricia López-Caballero,[1] Concepción Pérez,[1] José A. Morales-García,[3,4] Ana Pérez-Castillo,[3] Eric del Sastre,[5] Cristina Fernández-Mendívil,[5] Pablo Duarte,[5] Patrycja Michalska,[5] José Lombardía,[5] Sergio Senar,[6] Rafael León,[1,5] Manuela G. López,[5,7] and María Isabel Rodríguez-Franco[1,]*

[1]Instituto de Química Médica, Consejo Superior de Investigaciones Científicas (IQM-CSIC), C/ Juan de la Cierva 3, E-28006 Madrid, Spain.

[2]Programa de Doctorado en Química Orgánica, Facultad de Química, Universidad Complutense de Madrid, Avda. Complutense s/n, E-28040 Madrid, Spain.

[3]Instituto de Investigaciones Biomédicas (CSIC-UAM), C/ Arturo Duperier, 4. E-28029 and Centro de Investigación Biomédica en Red sobre Enfermedades Neurodegenerativas (CIBERNED), C/ Valderrebollo 5, E-28031 Madrid, Spain.

[4]Departamento de Biología Celular, Facultad de Medicina, Universidad Complutense de Madrid, Avda. Complutense s/n, E-28040 Madrid, Spain.

[5]Instituto Teófilo Hernando de I+D del Medicamento, Departamento de Farmacología y Terapéutica, Facultad de Medicina, Universidad Autónoma de Madrid, C/ Arzobispo Morcillo 4, E-28029 Madrid, Spain.

[6]DrTarget Machine Learning, C/ Alejo Carpentier 13, E-28806 Alcalá de Henares, Madrid, Spain.

[7]Instituto de Investigación Sanitaria del Hospital Universitario de la Princesa (IIS-IP), C/ Diego de León 62, E-28006 Madrid, Spain.

*Corresponding author: María Isabel Rodríguez-Franco, isabelrguez@iqm.csic.es

Table of Contents

Data Mining Studies in Open Targets Database (https://www.opentargets.org/) 3

Superposition Study of Minimized Structures of Resveratrol and Resveratrol-Based MTDLs 7

Theoretical and Experimental pKas of Resveratrol-Based MTDLs................................ 8

Monoamine Oxidase B (MAO-B) Binding Mode Elucidation: Molecular Docking Studies 9

Assays in Melatonin Receptors: hMT$_2$R and QR2.. 10

In Vitro CNS Permeation of Commercial Drugs Used as References in the PAMPA-BBB Assay 11

Drug-Like Calculations 12

Study of the Potential Union of **4e** to Glutathione 15

Western Blot Analysis of the Resveratrol-Based MTDL **4e** in Mouse Hippocampal Slices Subjected to OA-Induced Toxicity. 17

In Silico Metabolism Prediction of **4e**.. 18

HPLC-MS Data of Resveratrol-Based MTDLs 19

Spectroscopic Data of Resveratrol-Based MTDLs 26

REFERENCES 63

Data Mining Studies in Open Targets Database (https://www.opentargets.org/)

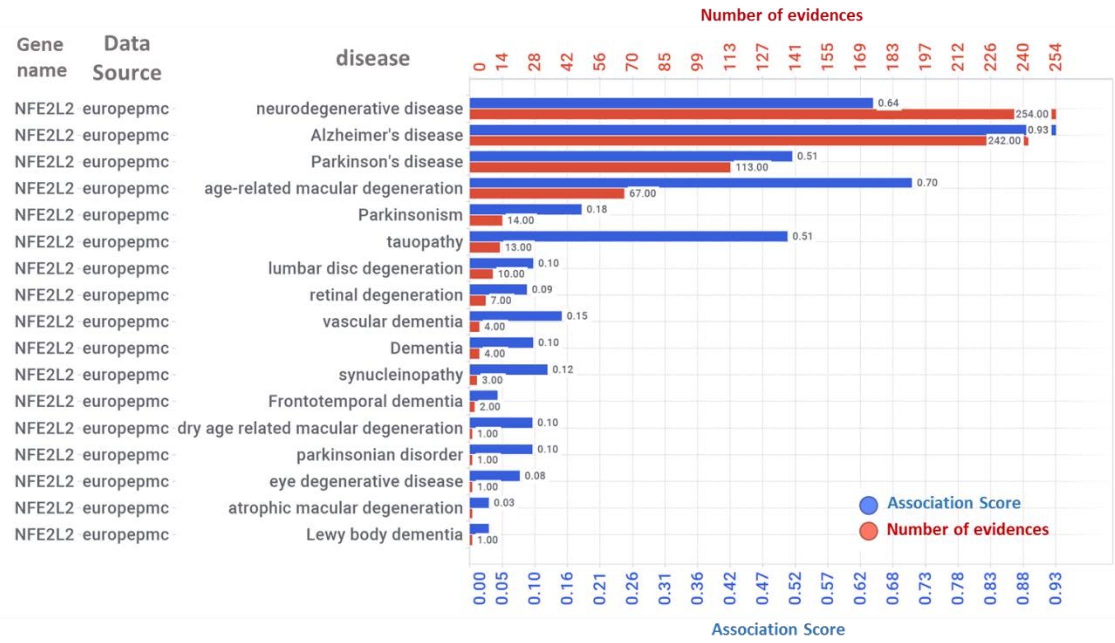

Figure S1. Open targets association scores and evidence counts for co-occurrence of NFE2L2 (NRF2) and NDs in Europe PMC data source. Red bar shows the number of aggregated evidences, while blue bars represent the average score for each relation.

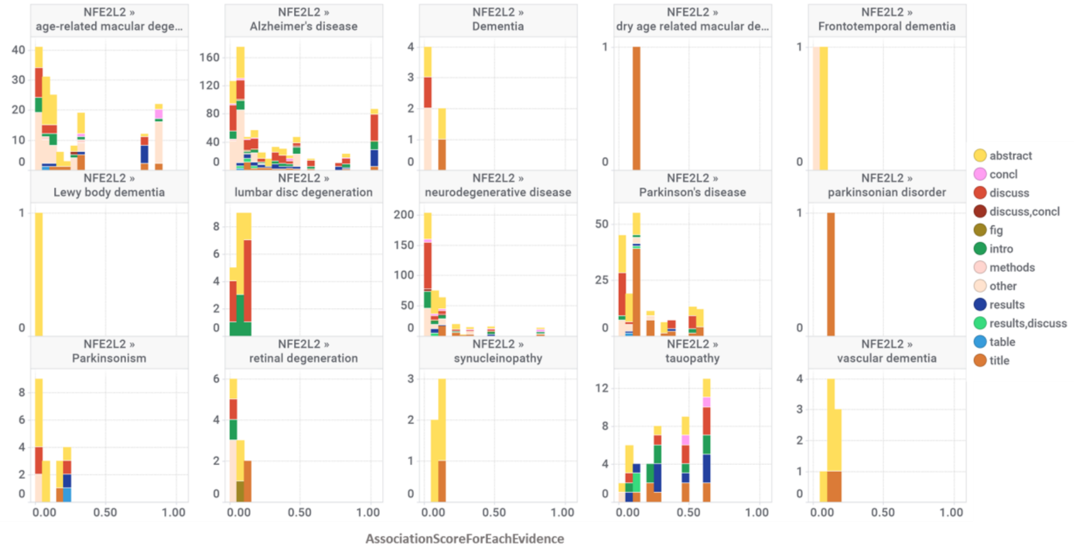

Figure S2. Open targets distribution scores for literature references associating NFE2L2 (NRF2) to NDs. Color by article section as described in legend.

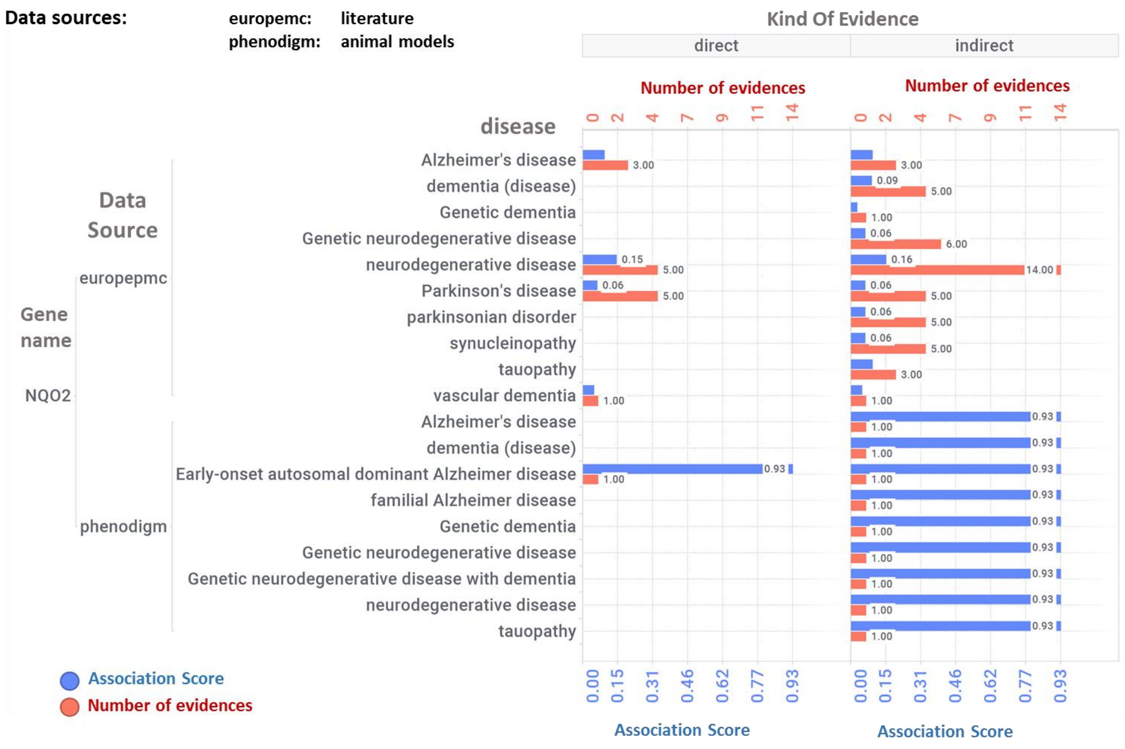

Figure S3. Open targets association scores and evidence counts for NQO2 and NDs in Europe PMC and PhenoDigm. Red bar shows the number of aggregated evidences, while blue bars represent the average score for each relation. Left and right panels correspond to direct and indirect evidences, respectively.

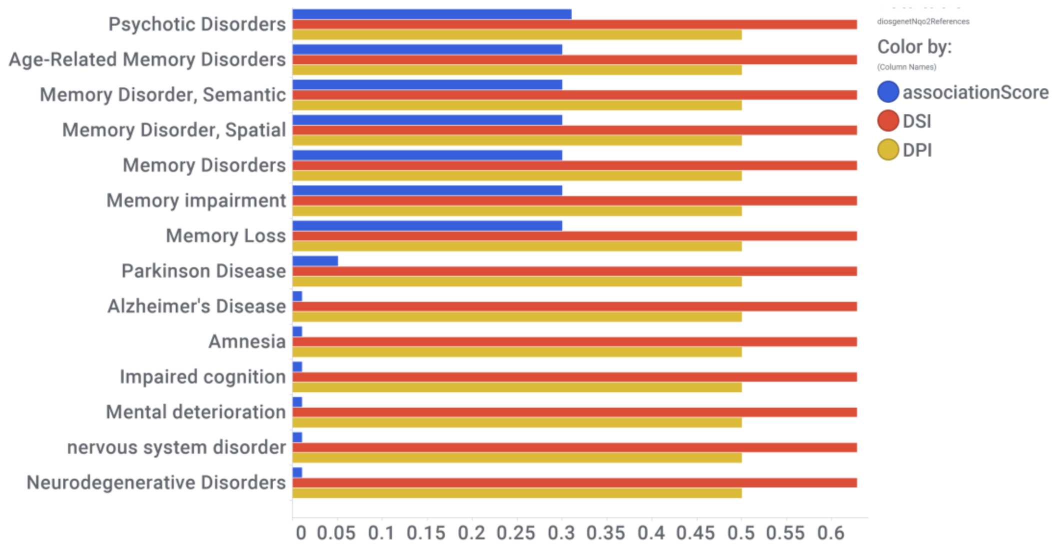

Figure S4. DisGeNet gene-disease association score and indexes for NQO2 association to NDs. The Disease Specificity Index (DSI) reflects if a gene (or variant) is associated to several or fewer diseases, while disease pleiotropy index (DPI) considers if the multiple diseases associated to the gene are similar among them.

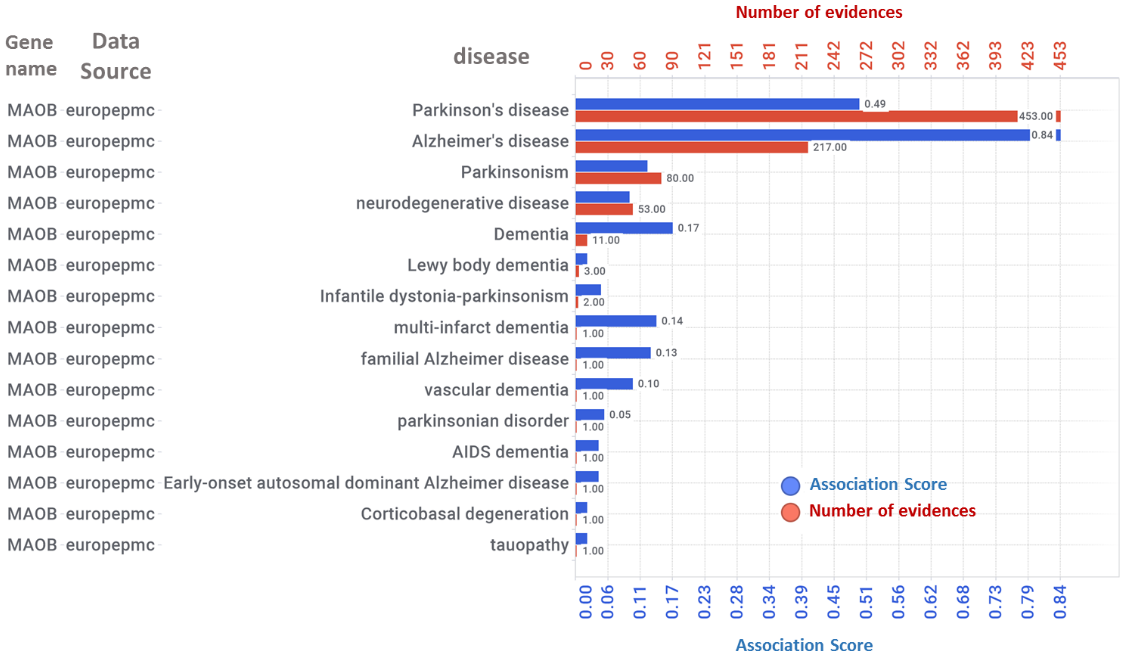

Figure S5. Open targets association scores and evidence counts for co-occurrence of MAO-B and NDs in Europe PMC data source. Red bar shows the number of aggregated evidences, while blue bars represent the average score for each relation.

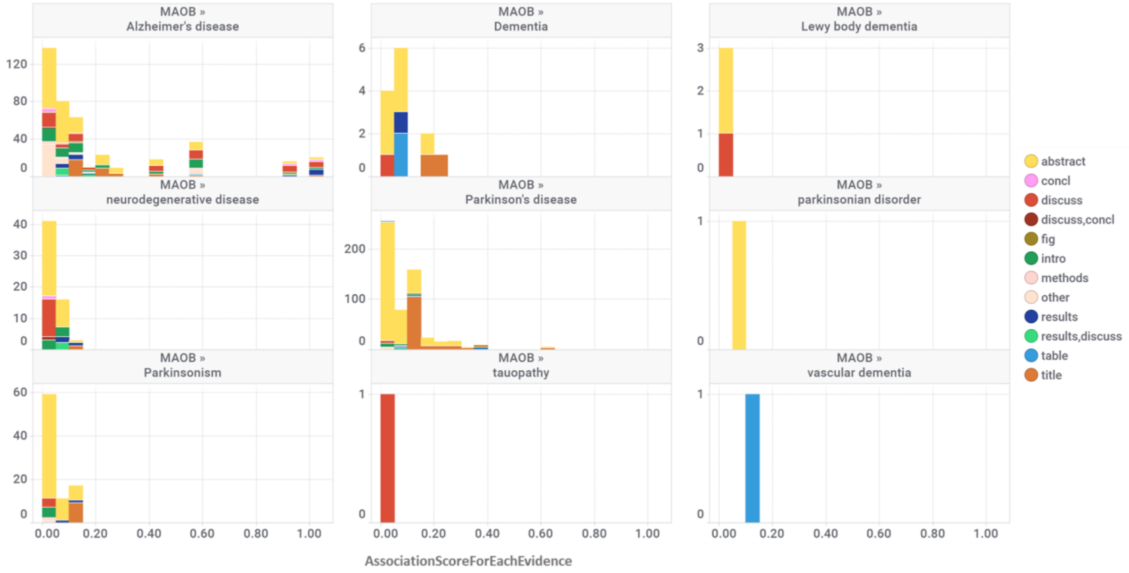

Figure S6. Open targets distribution scores for literature references associating MAO-B to NDs. Color by article section as described in legend.

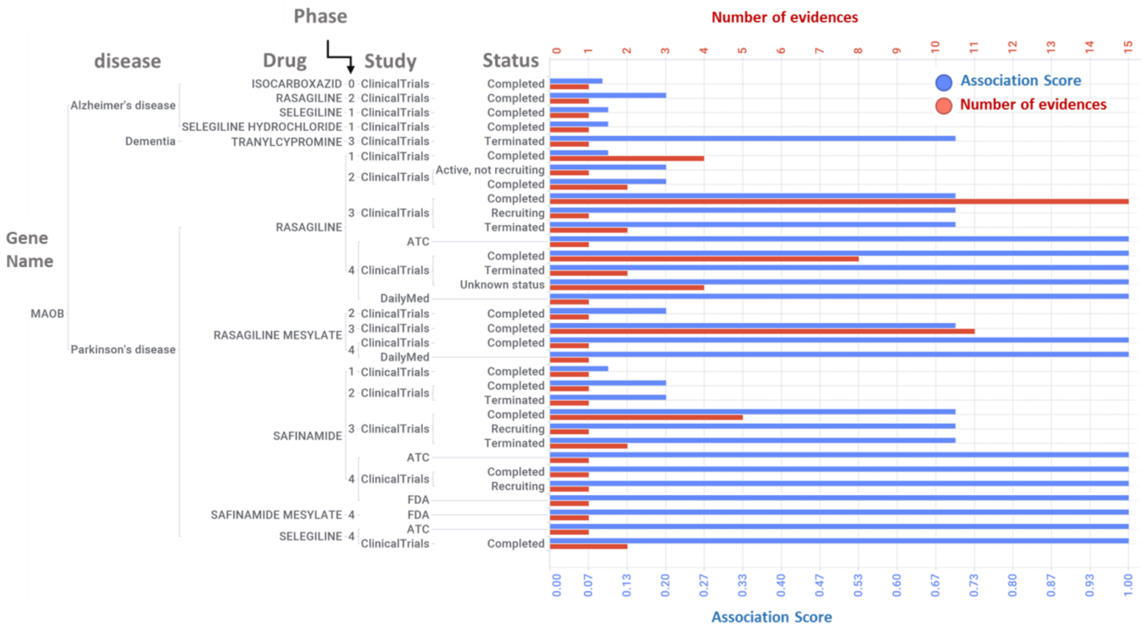

Figure S7. MAO-B inhibitors in clinical trials. Hierarchy by gene, disease, drug, kind of study-and status. Red bar shows the number of aggregated evidences, while blue bars represent the average score for each relation.

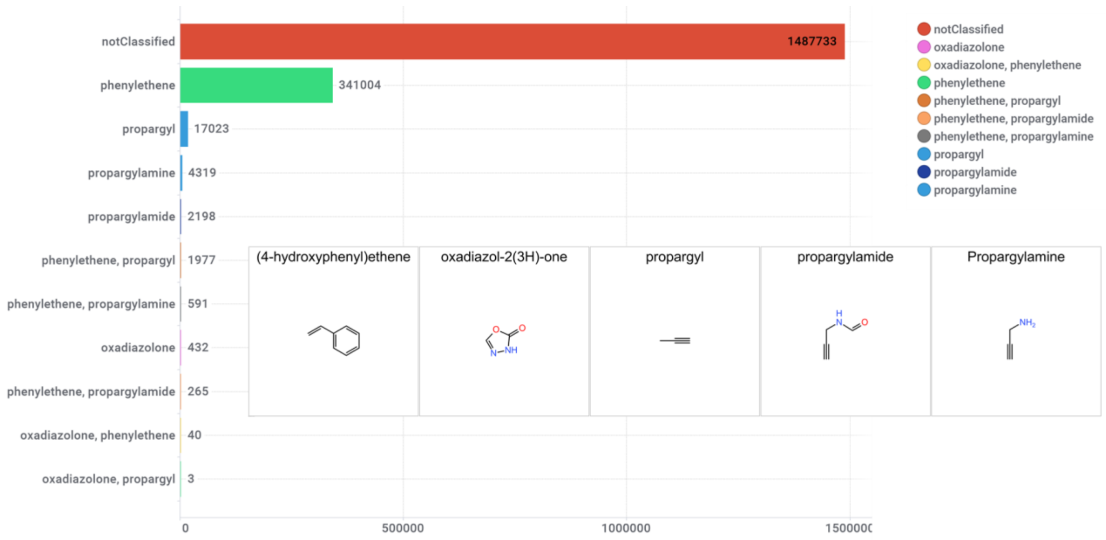

Figure S8. Occurrence of studied substructures in ChEMBL DB. Bar length is the number of molecules containing each substructure or combination of them.

Superposition Study of Minimized Structures of Resveratrol and Resveratrol-Based MTDLs

Energy minimization and pharmacophore analyses were carried out with Molecular Operating Environment software (MOE), Integrated Computer-Aided Molecular Design Platform.[1] Overlay and visualization were performed with Pymol software.[2]

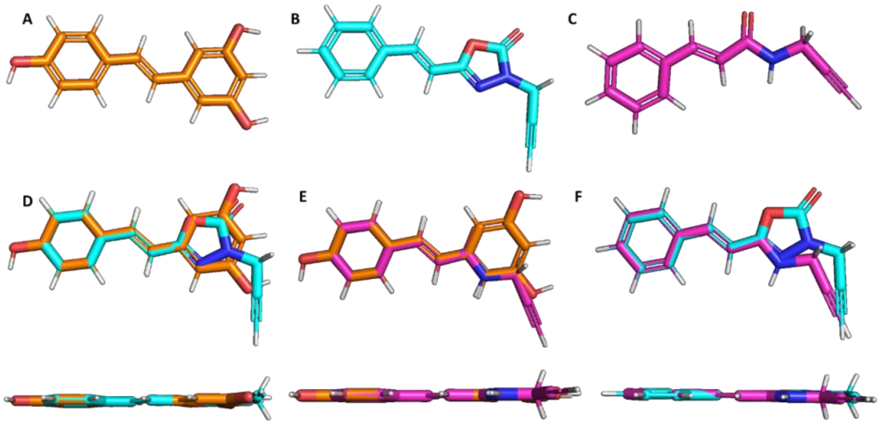

Figure S9. Minimized structure of resveratrol (A), phenyl–*N*-propargyloxadiazolone (B), and phenyl–*N*-propargylamide (C). Overlay of: resveratrol (orange) and phenyl–*N*-propargyloxadiazolone (cyan) (D); resveratrol (orange) and phenyl–*N*-propargylamide (pink) (E); phenyl–*N*-propargyloxadiazolone (cyan) and phenyl–*N*-propargylamide (pink) (F).

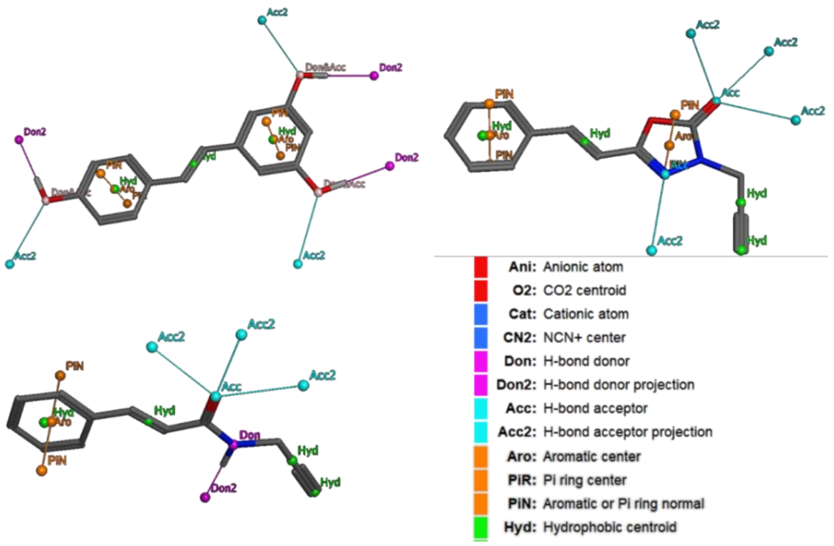

Figure S10. Pharmacophore of resveratrol (A), phenyl–*N*-propargyloxadiazolone (B), and phenyl–*N*-propargylamide (C). All structures are completely flat and share at least a hydrophobic link and an aromatic center. In the case of resveratrol and phenyl–*N*-propargyloxadiazolone, they share another aromatic ring.

Theoretical and Experimental pKas of Resveratrol-Based MTDLs

Table S1. Theoretical and Experimental pKas of Some Resveratrol-Based MTDLs

Compd.	Theoretical pK_as	Experimental pK_as
**2a**	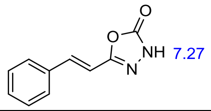	6.46 ± 0.03
**2d**	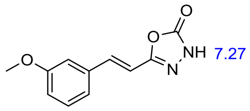	6.89 ± 0.00
**2e**	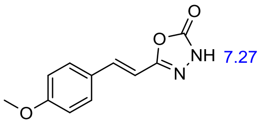	7.05 ± 0.00
**3l**	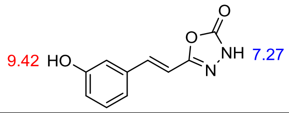	7.04 ± 0.02<br>8.72 ± 0.02
**3m**	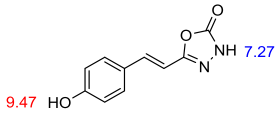	7.12 ± 0.01<br>9.14 ± 0.06
**5l**	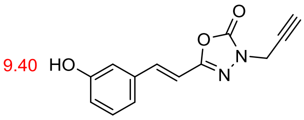	9.42 ± 0.01

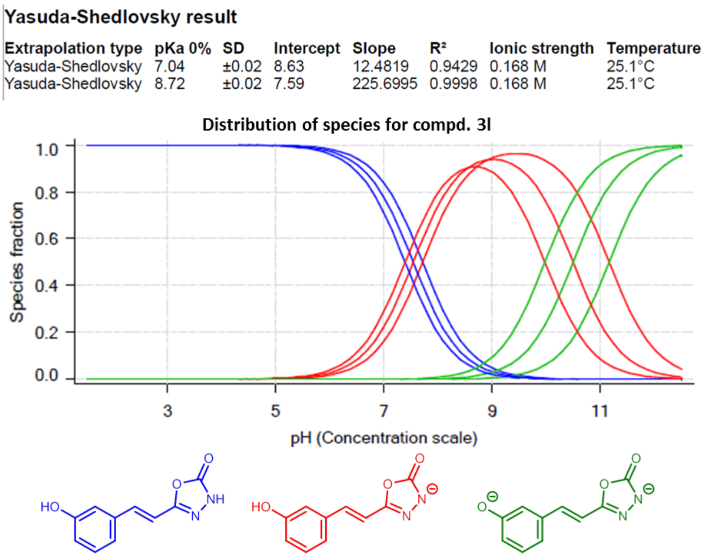

Figure S11. Experimental pK_as of compd. **3l** and distribution of species *vs.* pH.

Monoamine Oxidase B (MAO-B) Binding Mode Elucidation: Molecular Docking Studies

The 3D-structure of *N*-(3-chlorophenyl)-4-oxo-4*H*-chromene-3-carboxamide (**e92**), crystalized with the human monoamine oxidase B (MAO B) (PDB-ID, 6fw0), was submitted to our docking procedure to validate the methodology employed. Results show a similar pose with little atomic deviations compared to the crystal structure (Figure S4A). In addition to this, it is interesting to see how this reference MAO-B inhibitor exhibits a binding mode comparable to that of **2c** and **4e** along the bipartite MAO-B binding pocket (Figure S4B).

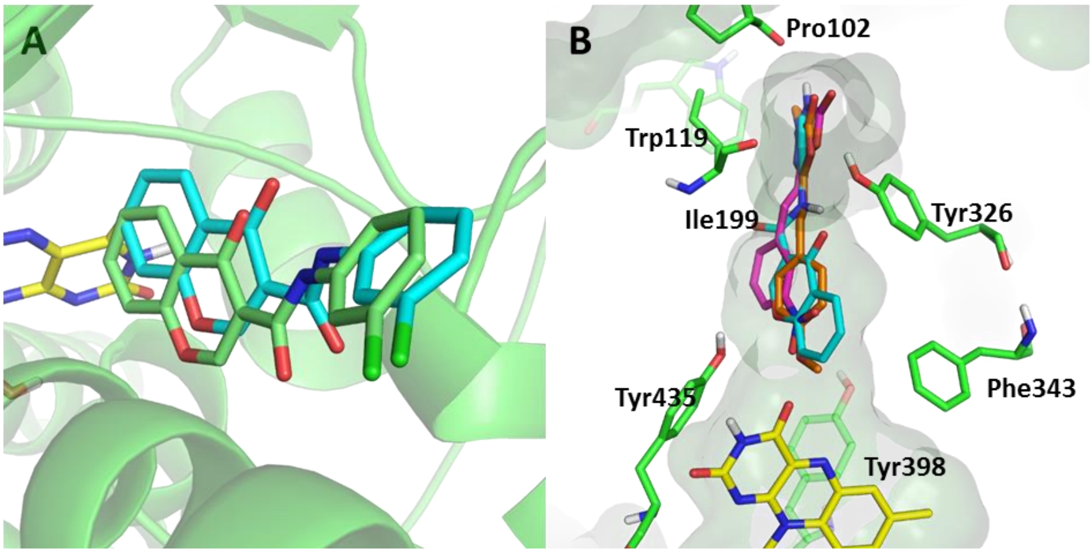

Figure S12. Docking studies in MAO-B. A) Validation of the docking method. Comparison between docking result (showed as blue sticks) and crystal data (showed as pale green sticks) of *N*-(3-chlorophenyl)-4-oxo-4*H*-chromene-3-carboxamide (**e92**) in the MAO-B binding site (PDB-ID 6FW0). MAO-B protein is represented as green cartoon with key residues as green sticks and FAD coenzyme as yellow sticks. B) Surface representation of MAO-B binding site with **2c**, **4e**, and **e92** docking poses along the bipartite cavity. MAO-B protein is represented as gray surface with key residues shown as green sticks and FAD coenzyme as yellow sticks.

Assays in Melatonin Receptors: *h*MT$_2$R and QR2

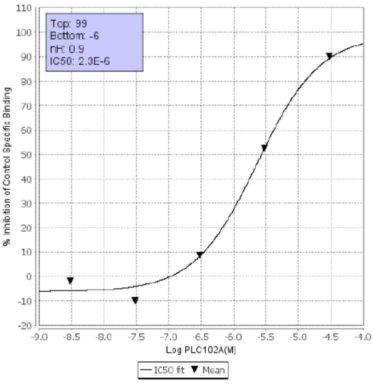

*h*MT$_2$R-Compd. **6d**

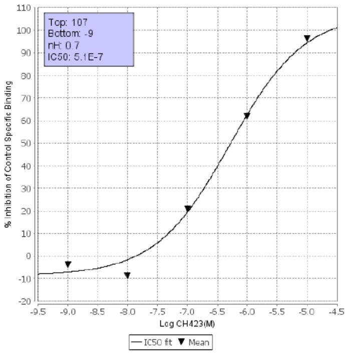

QR2-Compd. **2j**

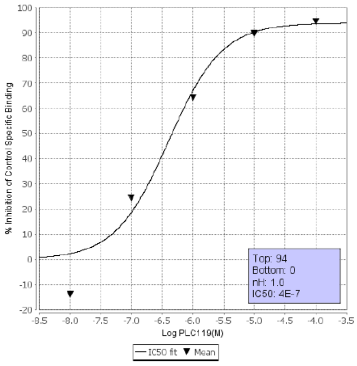

QR2-Compd. **4d**

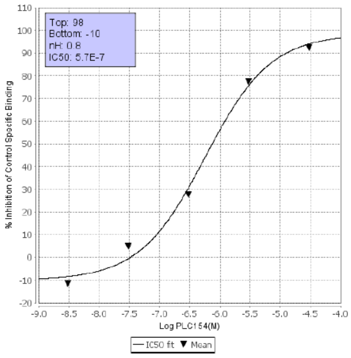

QR2-Compd. **4e**

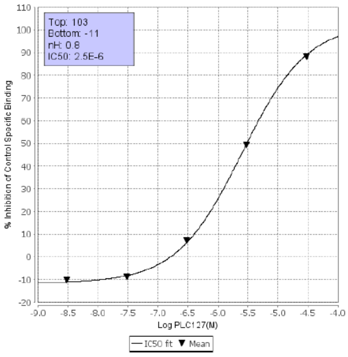

QR2-Compd. **5l**

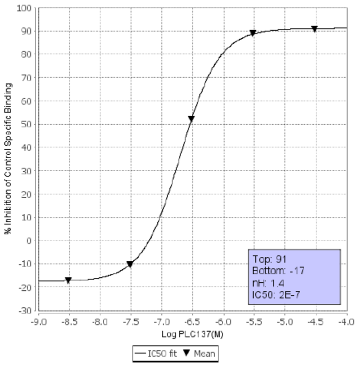

QR2-Compd. **8n**

Figure S13. Dose-response plots used for the calculation of IC$_{50}$s in *h*MT$_2$R and QR2.[3]

In Vitro CNS Permeation of Commercial Drugs Used as References in the PAMPA-BBB Assay

Table S2. Permeability P_e (10^{-6} cm/s) in the PAMPA assay of 11 commercial drugs used in the experimental validation.

Standard	Bibl.[a]	Exp[b]
Testosterone	17.0	15.6±1.3
Verapamil	16.0	18.7±1.3
Imipramine	13.0	9.0±1.0
Desipramine	12.0	12.0±1.3
Promazine	8.8	13.4±0.9
Corticosterone	5.1	5.0±0.2
Piroxicam	2.5	1.5±0.1
Hydrocortisone	1.9	3.8±0.1
Caffeine	1.3	1.2±0.1
Aldosterone	1.2	3.1±0.2
Ofloxacin	0.8	0.5±0.1

[a]Taken from Ref.[4] [b]Data are the mean ± SD of three independent experiments.

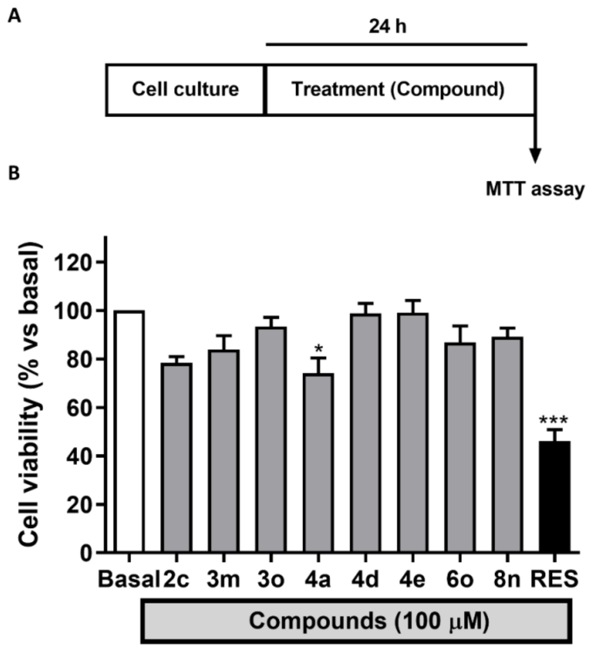

Figure S14. Toxicity *per se* of the resveratrol-based MTDLs in the human neuroblastoma cell line SH-SY5Y. (A) Experimental protocol used to evaluate toxicity of the compounds. (B) Cell viability, determined by MTT assay, after cell incubation with the compounds at 100 μM for 24h. RES = Resveratrol. Data are represented as cell viability normalized to the basal condition (100%). Bars show mean ± SEM. N=3-10 for each experiment that was performed in triplicate. Statistical analysis was performed following one-way ANOVA ($p<0.05$). *p<0. 5 and ***p<0.001 *vs* basal condition after Tukey post-hoc test.

Drug-Like Calculations

Medicinal chemistry alerts and calculated physicochemical properties were determined by KNIME software, an open-source data mining and workflow management system.[5] We carried out the workflow described in Figure S14, using the following nodes:

RDKit Descriptor was used for calculating the descriptors for each molecule in the input table. The user has the option to choose which descriptors need to be calculated.

Lipinski's Rule Filter is used to discard compounds with poor absorption (or permeation): molecules with more than 5 H-bond donors and / or more than 10 H-bond acceptors, a molecular weight over 500, and LogP over 5.

RDKit Molecule Catalog Filter contains a detailed view of the reasons why a molecule was filtered out. The node gives the option to generate one or more atoms list columns with the indexes of the atoms that matched the rule.

RDKit Molecule Highlighting creates an SVG column showing a molecule with highlighted atoms and bonds based on information in the input table.

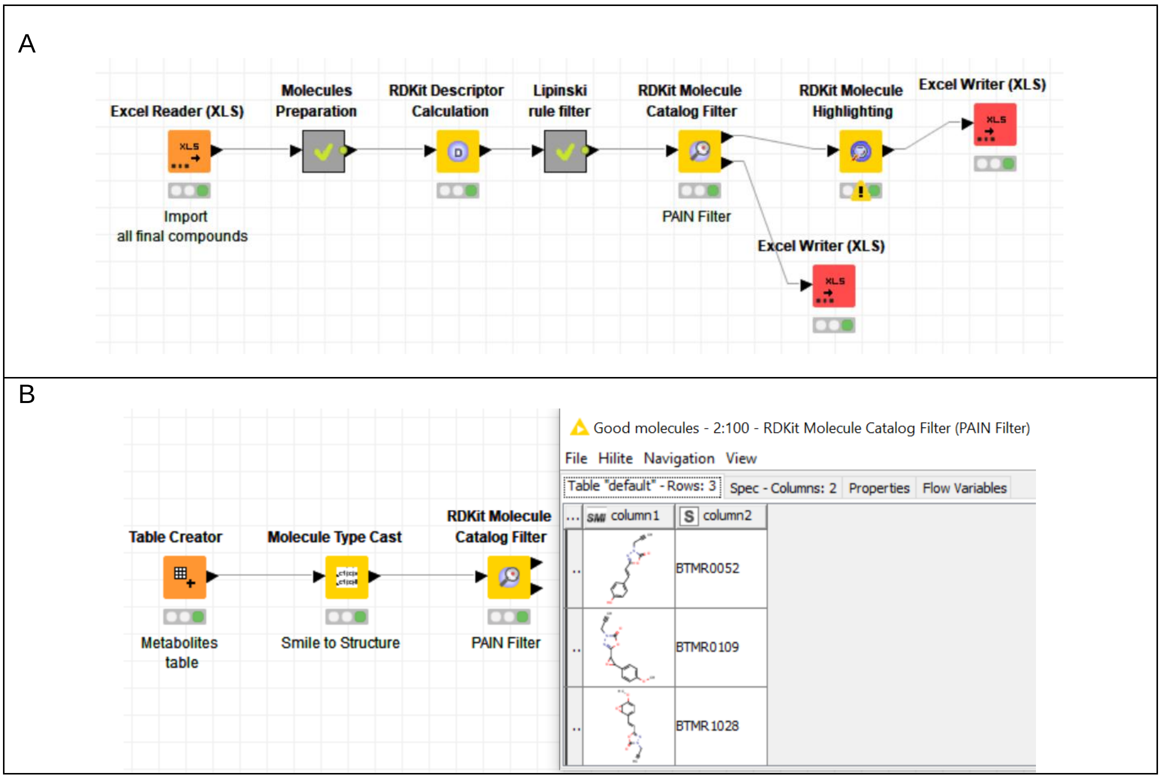

Figure S15. KNIME workflows used in this work. (A) All compounds were filtered according to the pan-assay interference rules (PAINS) and Lipinski guideline. (B) PAINS evaluation of phase-I metabolites of **4e** (no PAINS alerts were predicted).

Table S3. Calculated physicochemical properties of resveratrol-based MTDLs according to the KNIME software.[a]

Compd.	SlogP	SMR	Labute ASA	TPSA	AMW	Exact MW	Num Lipinski HBA	Num Lipinski HBD	Num Rotatable Bonds	Num HBD	Num HBA	Num Amide Bonds	Num Hetero Atoms	Num Heavy Atoms
**2a**	1.5333	52.4937	79.8924	58.89	188.186	188.0586	4	1	2	1	3	0	4	14
**2b**	1.4050	57.2087	90.6499	82.68	213.196	213.0538	5	1	2	1	4	0	5	16
**2c**	1.4415	59.1481	94.5454	102.03	233.183	233.0437	7	1	3	1	5	0	7	17
**2d**	1.5419	59.0457	91.3709	68.12	218.212	218.0691	5	1	3	1	4	0	5	16
**2e**	1.5419	59.0457	91.3709	68.12	218.212	218.0691	5	1	3	1	4	0	5	16
**2f**	1.5505	65.5977	102.8494	77.35	248.238	248.0797	6	1	4	1	5	0	6	18
**2g**	1.5505	65.5977	102.8494	77.35	248.238	248.0797	6	1	4	1	5	0	6	18
**2h**	1.5505	65.5977	102.8494	77.35	248.238	248.0797	6	1	4	1	5	0	6	18
**2i**	1.2620	58.6167	95.4786	77.35	232.195	232.0484	6	1	2	1	5	0	6	17
**2j**	1.5505	65.5977	102.8494	77.35	248.238	248.0797	6	1	4	1	5	0	6	18
**2k**	1.5591	72.1497	114.3278	86.58	278.264	278.0903	7	1	5	1	6	0	7	20
**3l**	1.2389	54.1585	84.6866	79.12	204.185	204.0535	5	2	2	2	4	0	5	15
**3m**	1.2389	54.1585	84.6866	79.12	204.185	204.0535	5	2	2	2	4	0	5	15
**3p**	0.9445	55.8233	89.4808	99.35	220.184	220.0484	6	3	2	3	5	0	6	16
**4a**	1.6399	64.9660	98.1964	48.03	226.235	226.0742	4	0	3	0	4	0	4	17
**4d**	1.6485	71.5180	109.6749	57.26	256.261	256.0848	5	0	4	0	5	0	5	19
**4e**	1.6485	71.5180	109.6749	57.26	256.261	256.0848	5	0	4	0	5	0	5	19
**5l**	1.3455	66.6308	102.9906	68.26	242.234	242.0691	5	1	3	1	5	0	5	18
**6b**	1.3209	62.0337	94.3996	52.89	210.236	210.0793	3	1	3	1	2	1	3	16
**6d**	1.4578	63.8707	95.1205	38.33	215.252	215.0946	3	1	4	1	2	1	3	16
**6e**	1.4578	63.8707	95.1205	38.33	215.252	215.0946	3	1	4	1	2	1	3	16
**6l**	1.1548	58.9835	88.4363	49.33	201.225	201.0790	3	2	3	2	2	1	3	15
**6m**	1.1548	58.9835	88.4363	49.33	201.225	201.0790	3	2	3	2	2	1	3	15
**6n**	1.1634	65.5355	99.9148	58.56	231.251	231.0895	4	2	4	2	3	1	4	17
**8n**	1.6368	65.3955	95.7533	41.49	217.268	217.1103	3	2	5	2	3	0	3	16

[a] Compds **3o** and **6o** are omitted because they show a medicinal chemistry alert due to the presence of a catechol fragment in their structures.

Study of the Potential Union of 4e to Glutathione

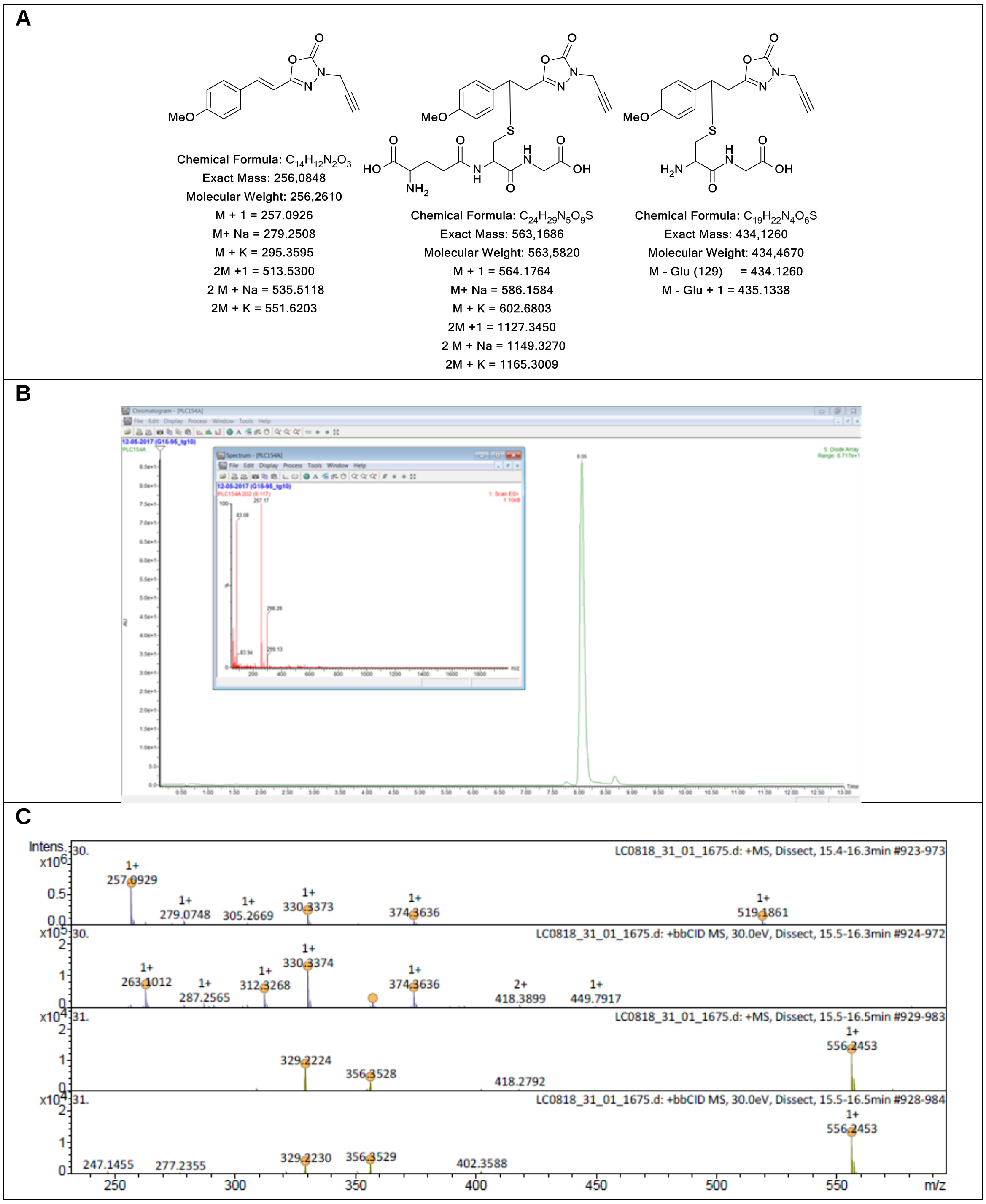

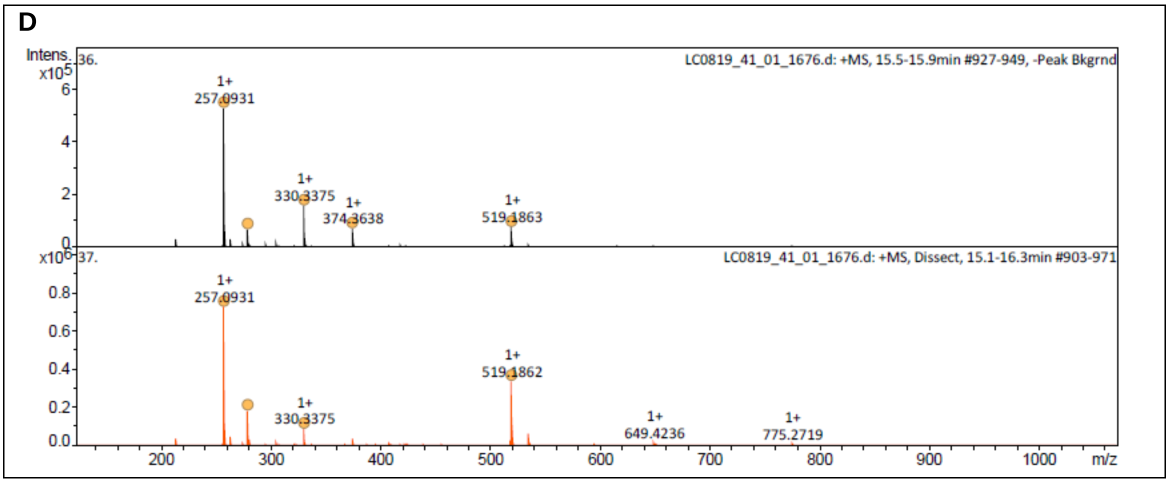

Figure S16. (A) Structures and expected m/z values of compound **4e** and its potential adducts with GSH. (B) HPLC-MS of compound **4e.** (C) Non-enzymatic reaction (**4e** + GSH). (D) Enzymatic reaction (**4e** + GSH + GST).

Western Blot Analysis of the Resveratrol-Based MTDL 4e in Mouse Hippocampal Slices Subjected to OA-Induced Toxicity.

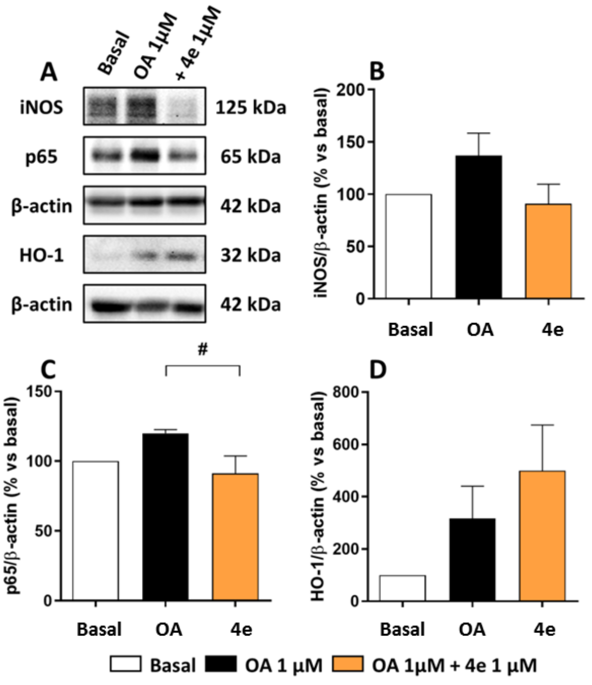

Figure S17. Compound **4e** shows a tendency to reduce inflammatory proteins and to increase antioxidant enzymes. (A) Representative western blot images. Quantification of iNOS (B), p65 (C) and HO-1 (D) in comparison to β-actin. One-way ANOVA followed by Tukey's post-hoc test. Statistical differences are represented as #$p<0.05$ in comparison to OA. Results are displayed as mean ± S.E.M. (N = 4-9).

In Silico Metabolism Prediction of 4e

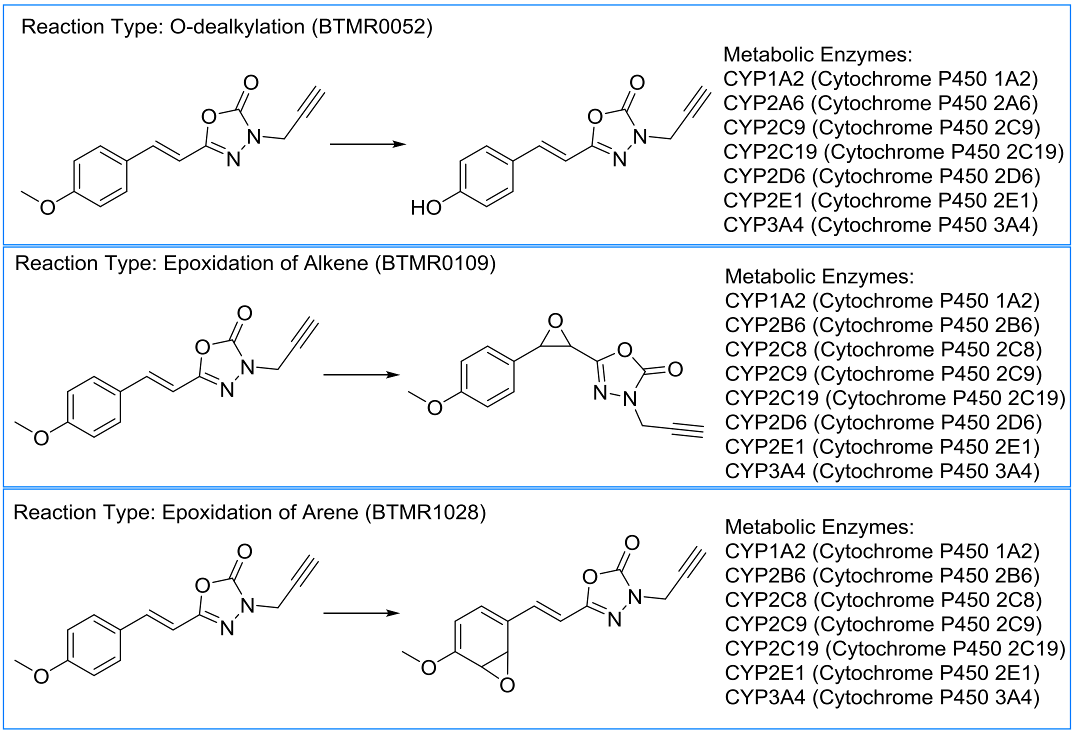

Figure S18. Phase I (CYP450) metabolism prediction of resveratrol-based MTDL **4e**, according to the BioTransformer platform.

HPLC-MS Data of Resveratrol-Based MTDLs

3-[(*E*)-2-(5-Oxo-4,5-dihydro-1,3,4-oxadiazol-2-yl)ethenyl]benzonitrile (**2b**)

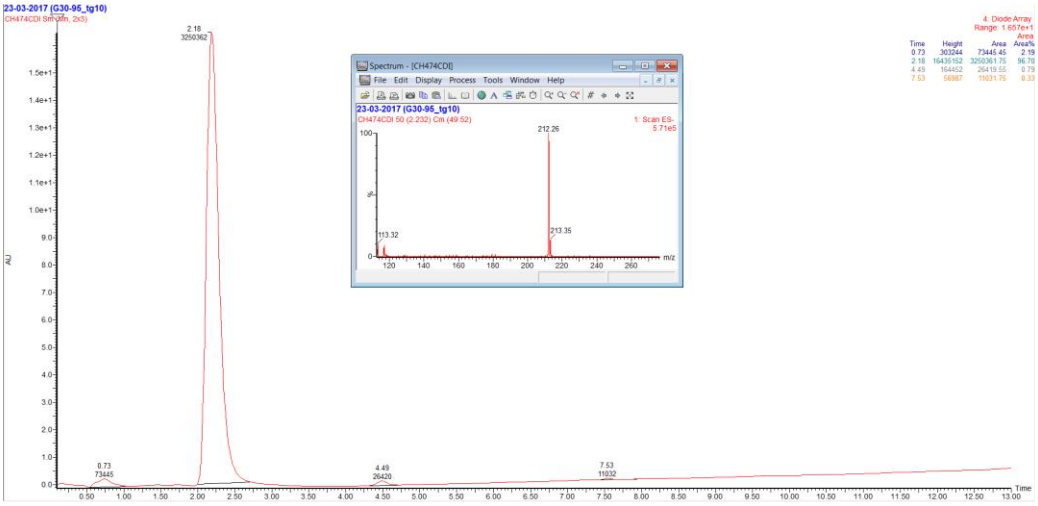

5-[(*E*)-2-(3-Nitrophenyl)ethenyl]-1,3,4-oxadiazol-2(3*H*)-one (**2c**)

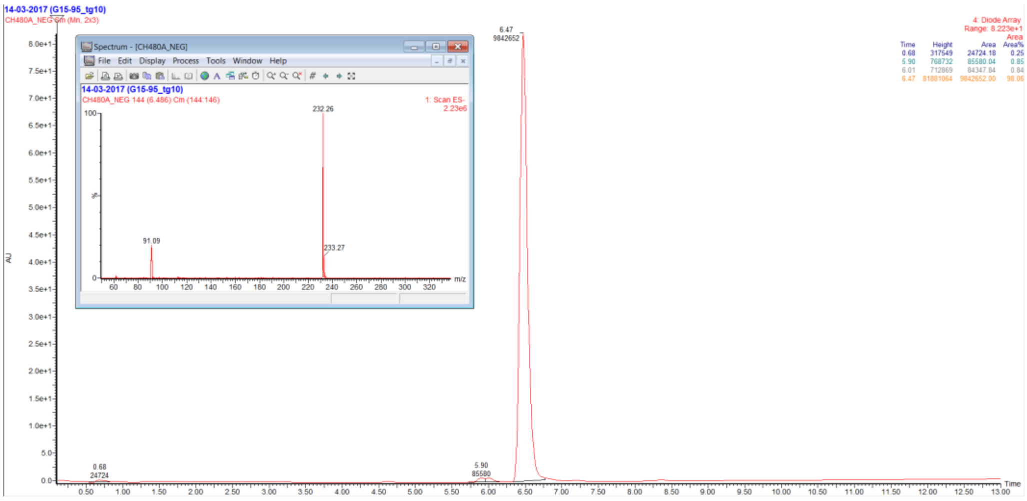

5-[(*E*)-2-(3-Methoxyphenyl)ethenyl]-1,3,4-oxadiazol-2(3*H*)-one (**2d**)

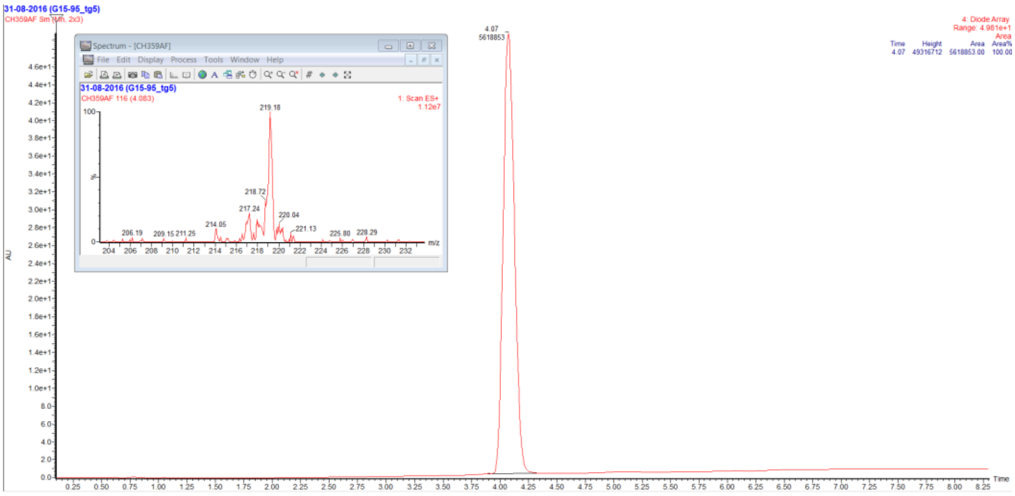

5-[(*E*)-2-(4-Methoxyphenyl)ethenyl]-1,3,4-oxadiazol-2(3*H*)-one (**2e**)

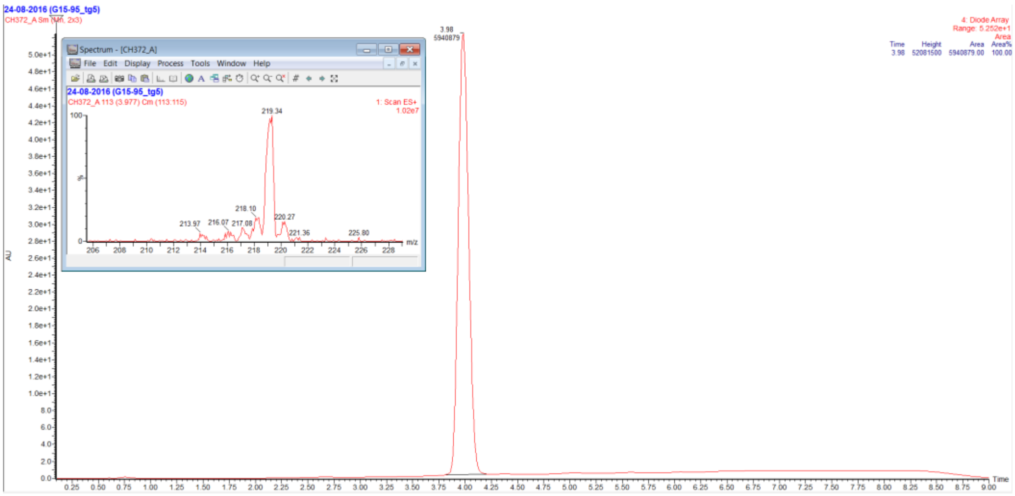

5-[(*E*)-2-(2,4-Dimethoxyphenyl)ethenyl]-1,3,4-oxadiazol-2(3*H*)-one (**2f**)

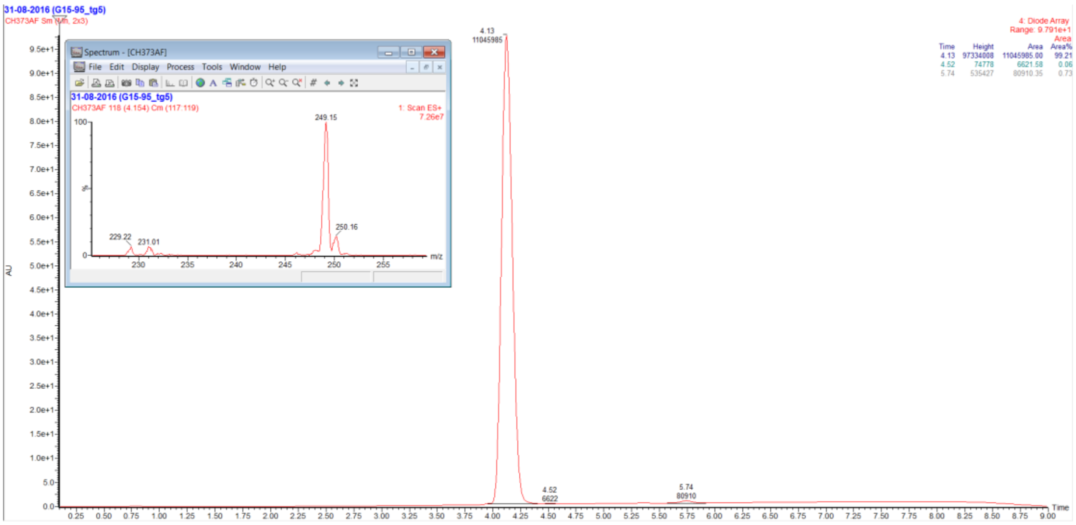

5-[(*E*)-2-(2,5-Dimethoxyphenyl)ethenyl]-1,3,4-oxadiazol-2(3*H*)-one (**2g**)

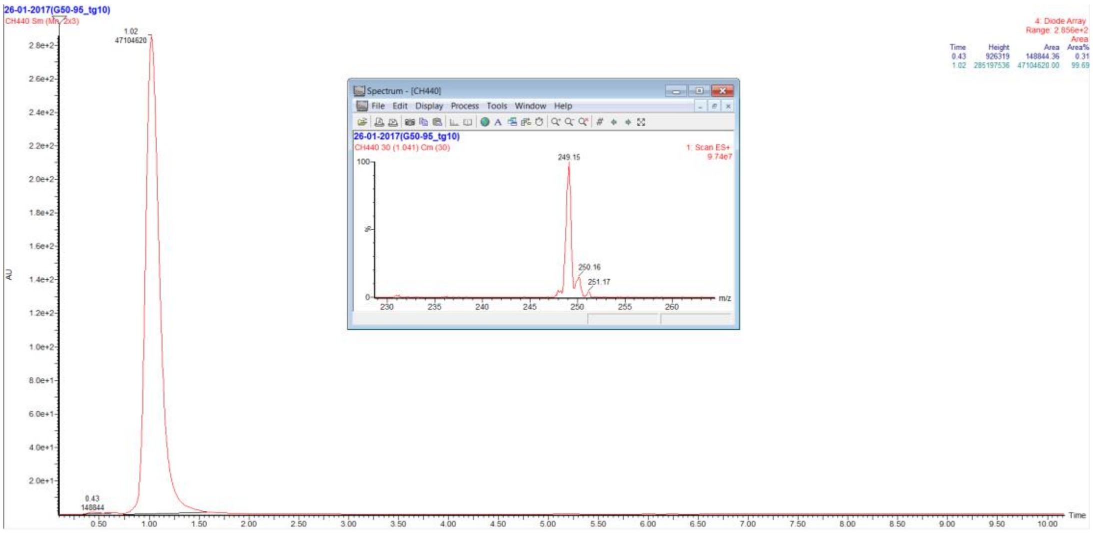

5-[(*E*)-2-(3,4-Dimethoxyphenyl)ethenyl]-1,3,4-oxadiazol-2(3*H*)-one (**2h**)

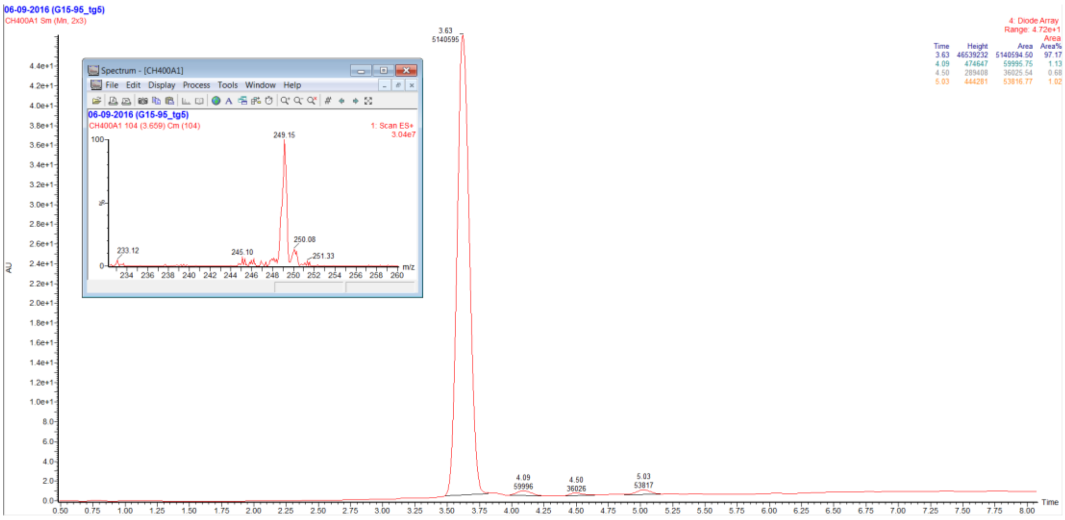

5-[(*E*)-2-(2*H*-1,3-Benzodioxol-5-yl)ethenyl]-1,3,4-oxadiazol-2(3*H*)-one (**2i**)

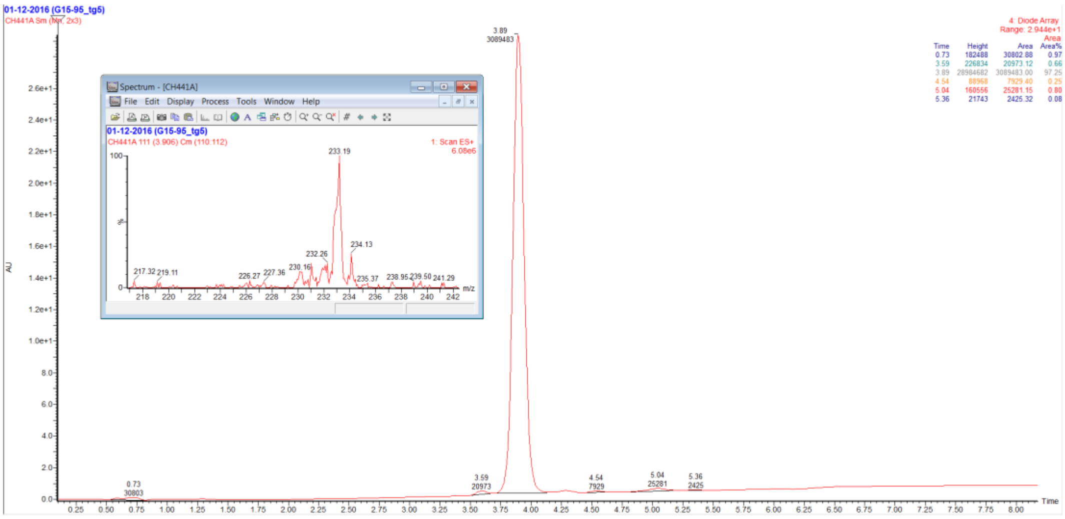

5-[(*E*)-2-(3,5-Dimethoxyphenyl)ethenyl]-1,3,4-oxadiazol-2(3*H*)-one (**2j**)

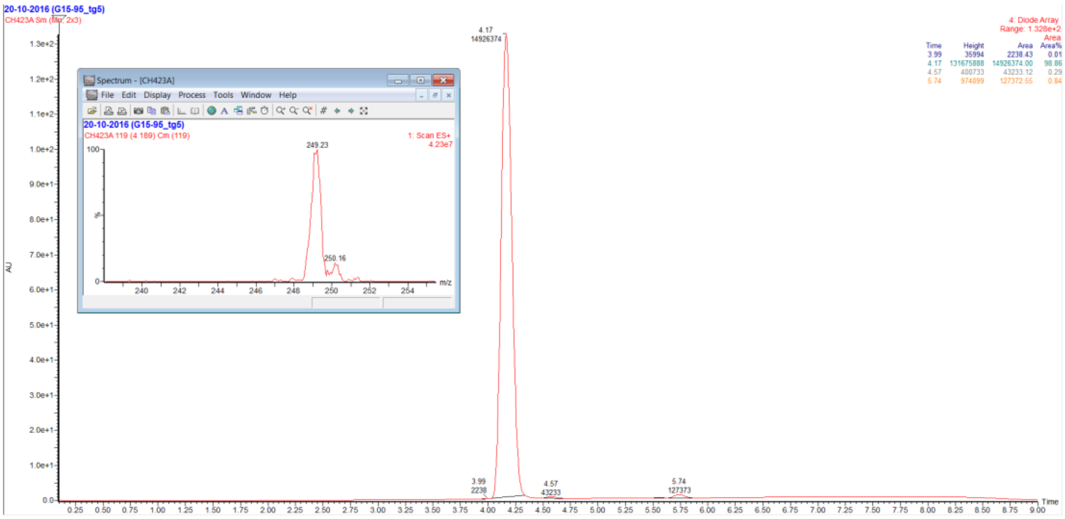

5-[(*E*)-2-(3,4,5-Trimethoxyphenyl)ethenyl]-1,3,4-oxadiazol-2(3*H*)-one (**2k**)

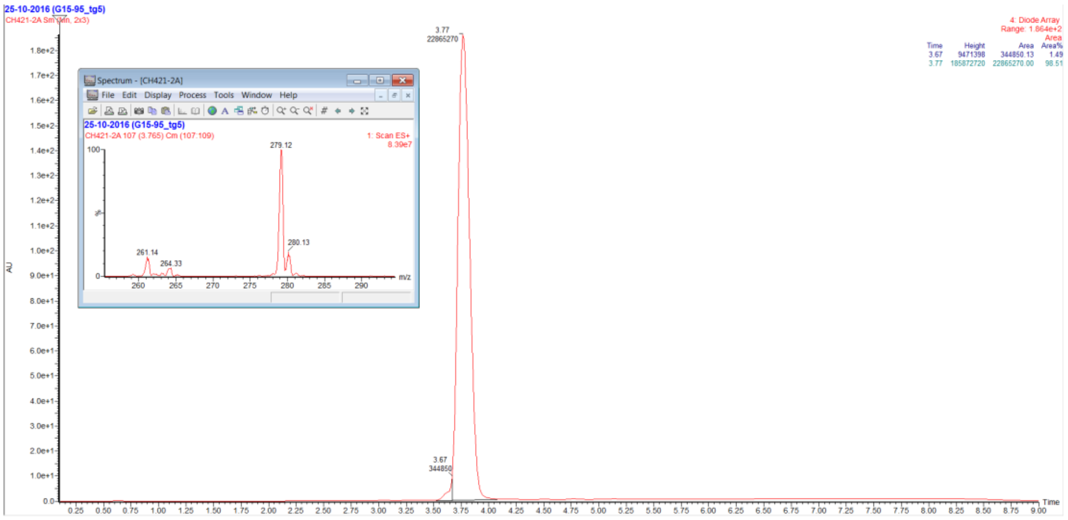

5-[(*E*)-2-(3-Hydroxyphenyl)ethenyl]-1,3,4-oxadiazol-2(3*H*)-one (**3l**)

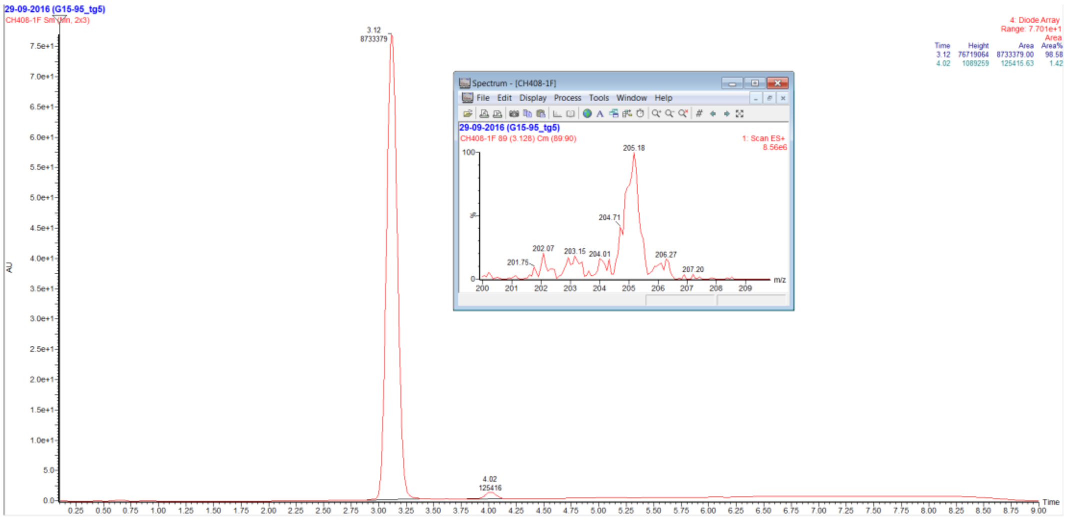

5-[(*E*)-2-(4-Hydroxyphenyl)ethenyl]-1,3,4-oxadiazol-2(3*H*)-one (**3m**)

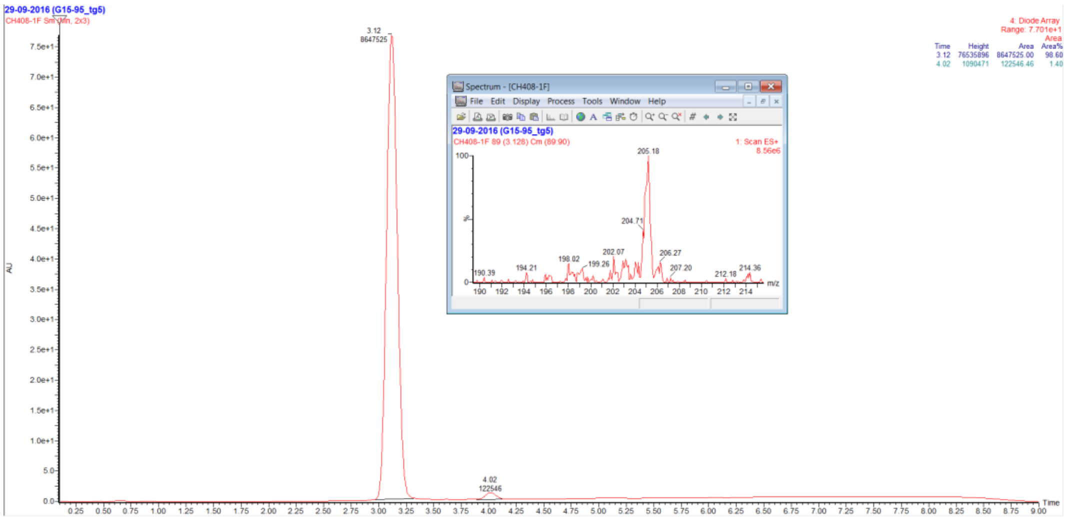

5-[(*E*)-2-(3,4-Dihydroxyphenyl)ethenyl]-1,3,4-oxadiazol-2(3*H*)-one (**3o**)

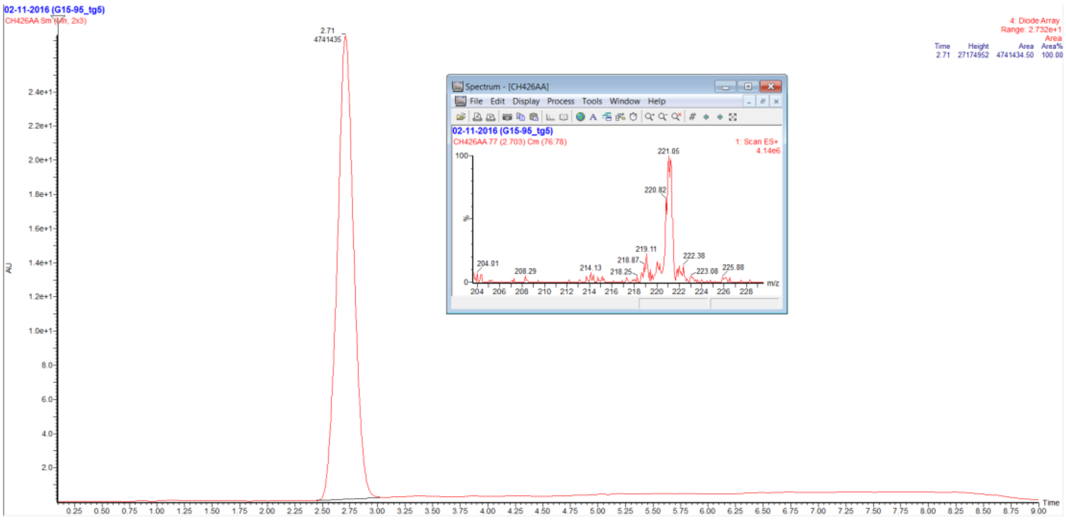

5-[(*E*)-2-(3,5-Dihydroxyphenyl)ethenyl]-1,3,4-oxadiazol-2(3*H*)-one (**3p**)

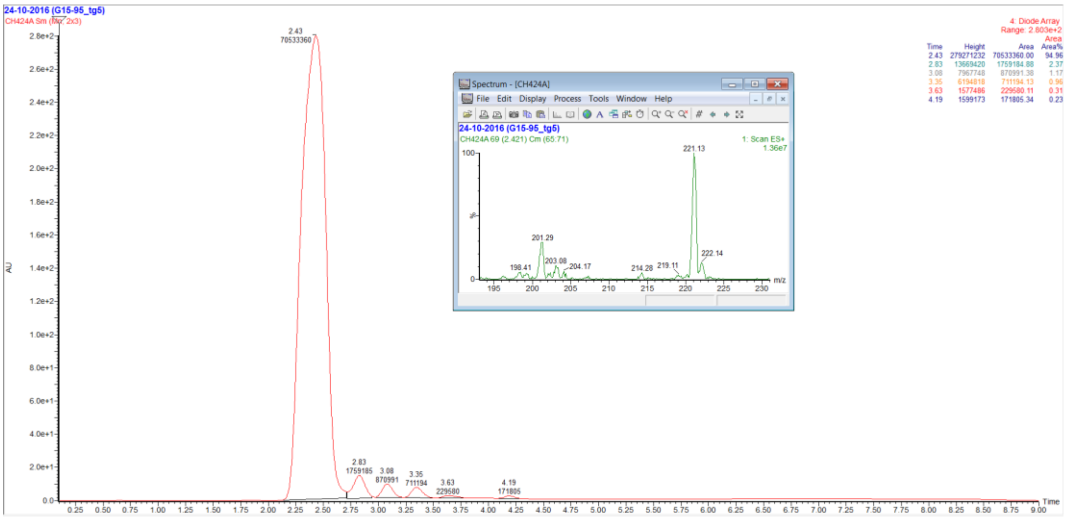

5-[(*E*)-2-Phenylethenyl]-3-(prop-2-yn-1-yl)-1,3,4-oxadiazol-2(3*H*)-one (**4a**)

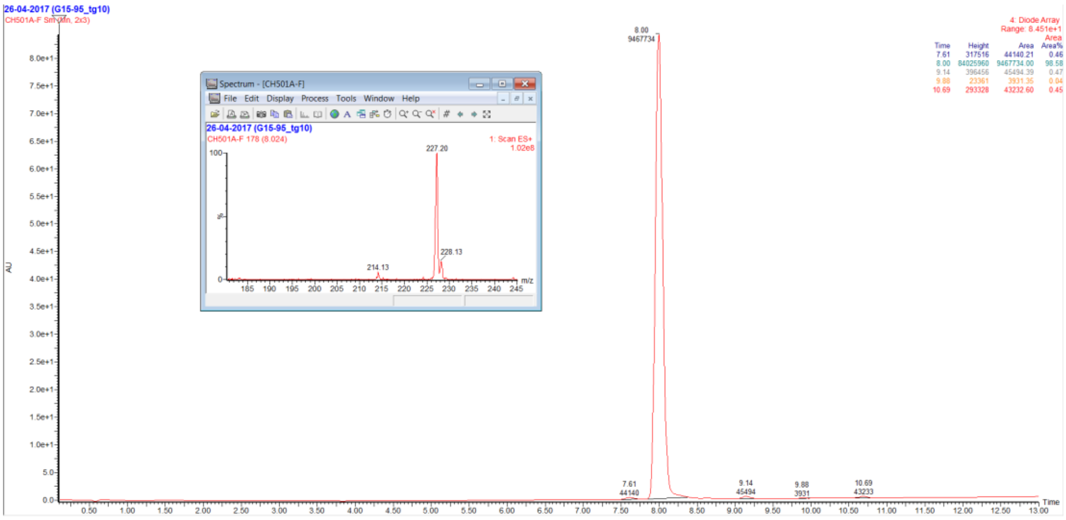

5-[(*E*)-2-(3-Methoxyphenyl)ethenyl]-3-(prop-2-yn-1-yl)-1,3,4-oxadiazol-2(3*H*)-one (**4d**)

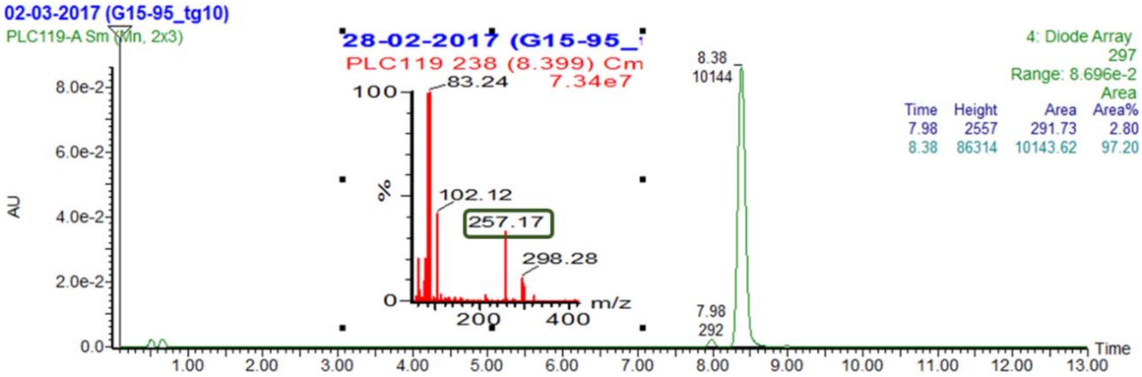

5-[(*E*)-2-(4-Methoxyphenyl)ethenyl]-3-(prop-2-yn-1-yl)-1,3,4-oxadiazol-2(3*H*)-one (**4e**)

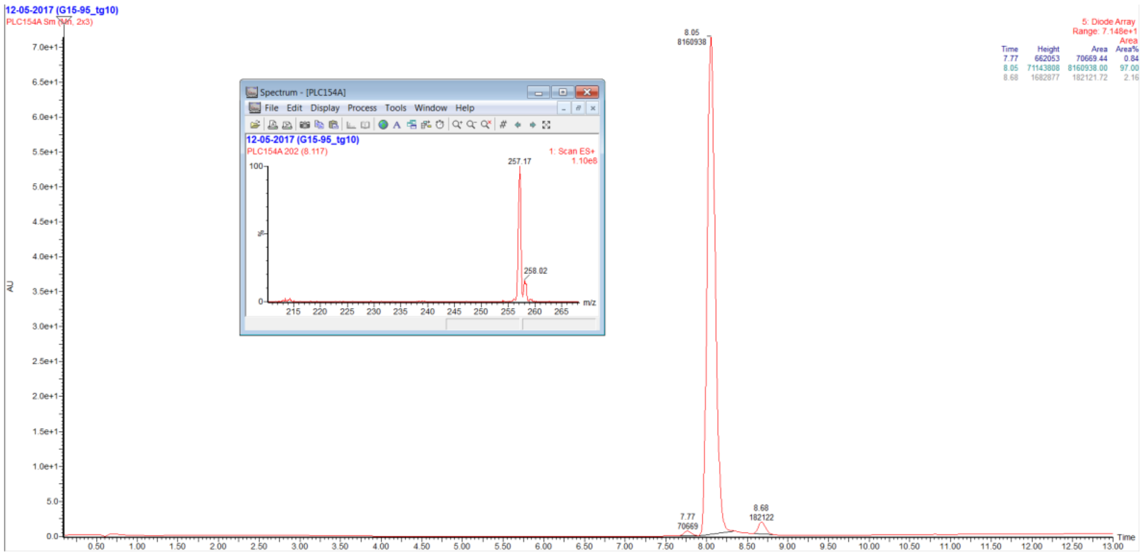

5-[(*E*)-2-(3-Hydroxyphenyl)ethenyl]-3-(prop-2-yn-1-yl)-1,3,4-oxadiazol-2(3*H*)-one (**5l**)

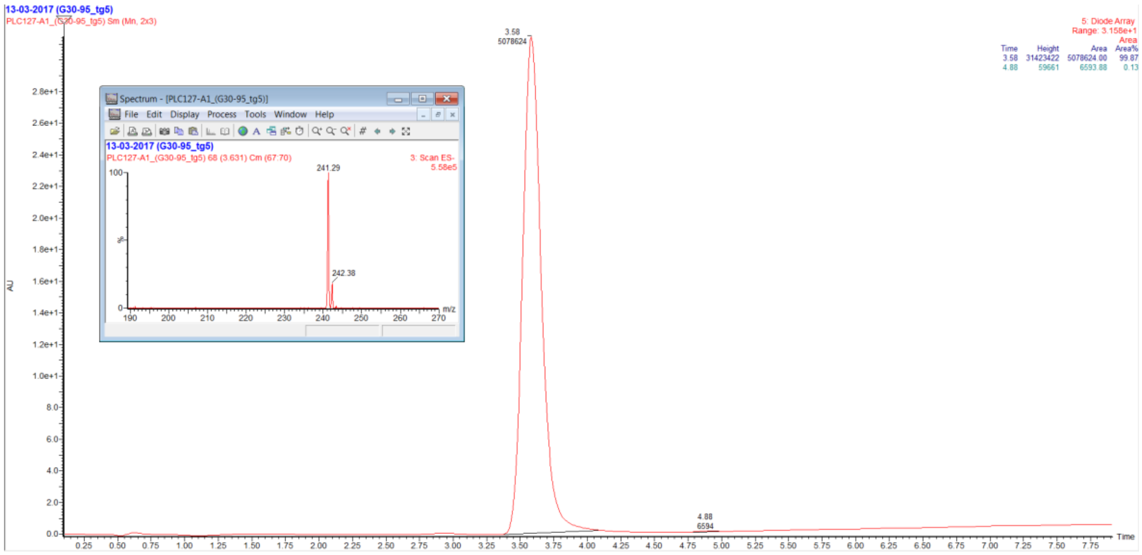

(2*E*)-3-(3-Methoxyphenyl)-*N*-(prop-2-yn-1-yl)prop-2-enamide (**6d**)

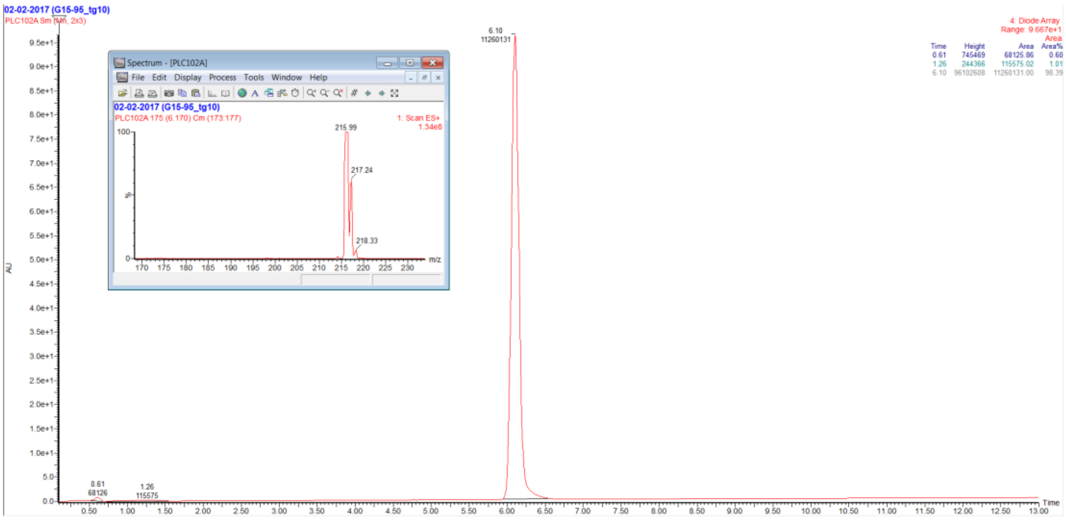

2*E*)-3-(3-Hydroxyphenyl)-*N*-(prop-2-yn-1-yl)prop-2-enamide (**6l**)

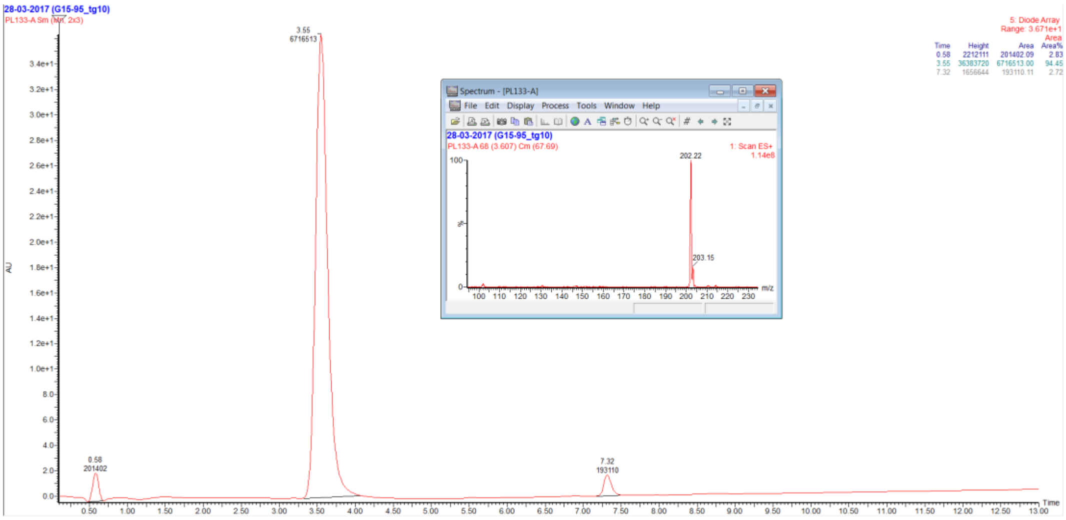

(2*E*)-3-(3,4-Dihydroxyphenyl)-*N*-(prop-2-yn-1-yl)prop-2-enamide (**6o**)

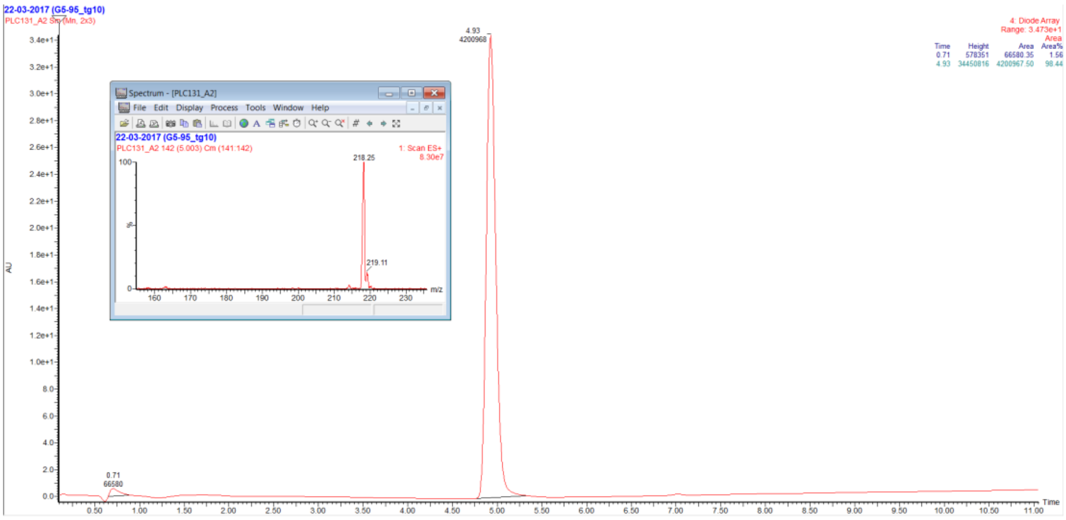

Spectroscopic Data of Resveratrol-Based MTDLs

5-[(*E*)-2-(3-Nitrophenyl)ethenyl]-1,3,4-oxadiazol-2(3*H*)-one (2c)

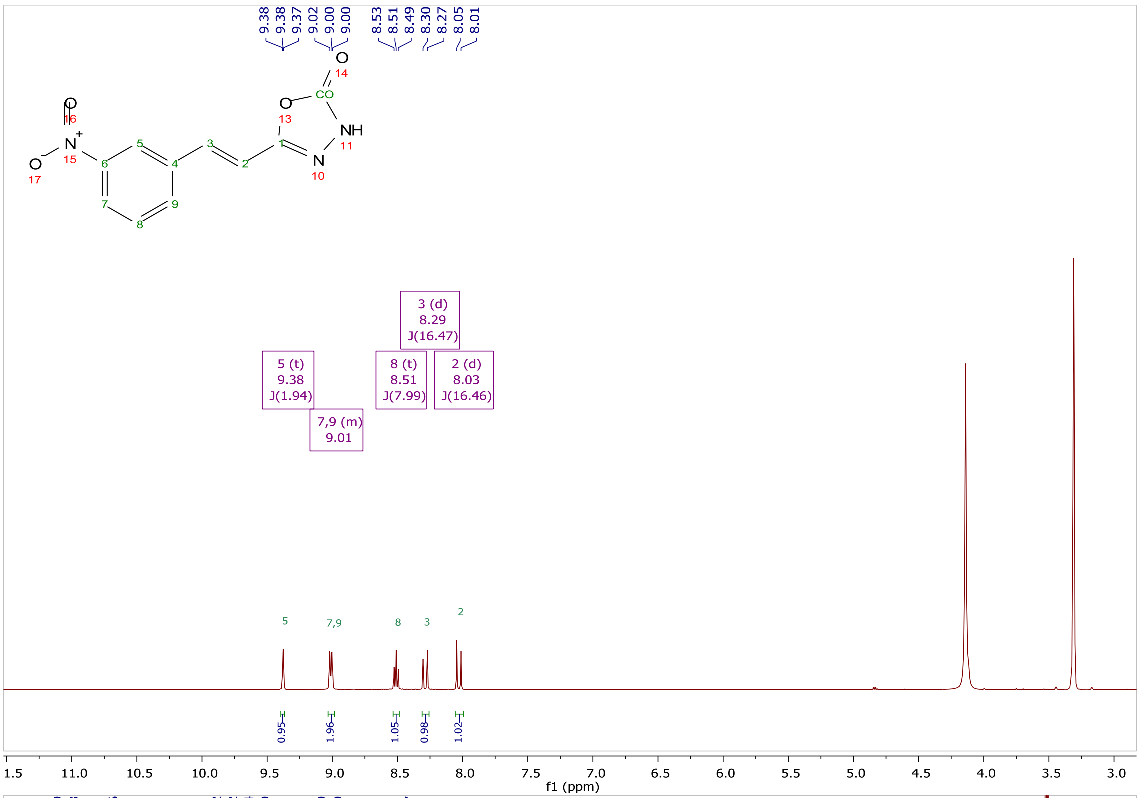

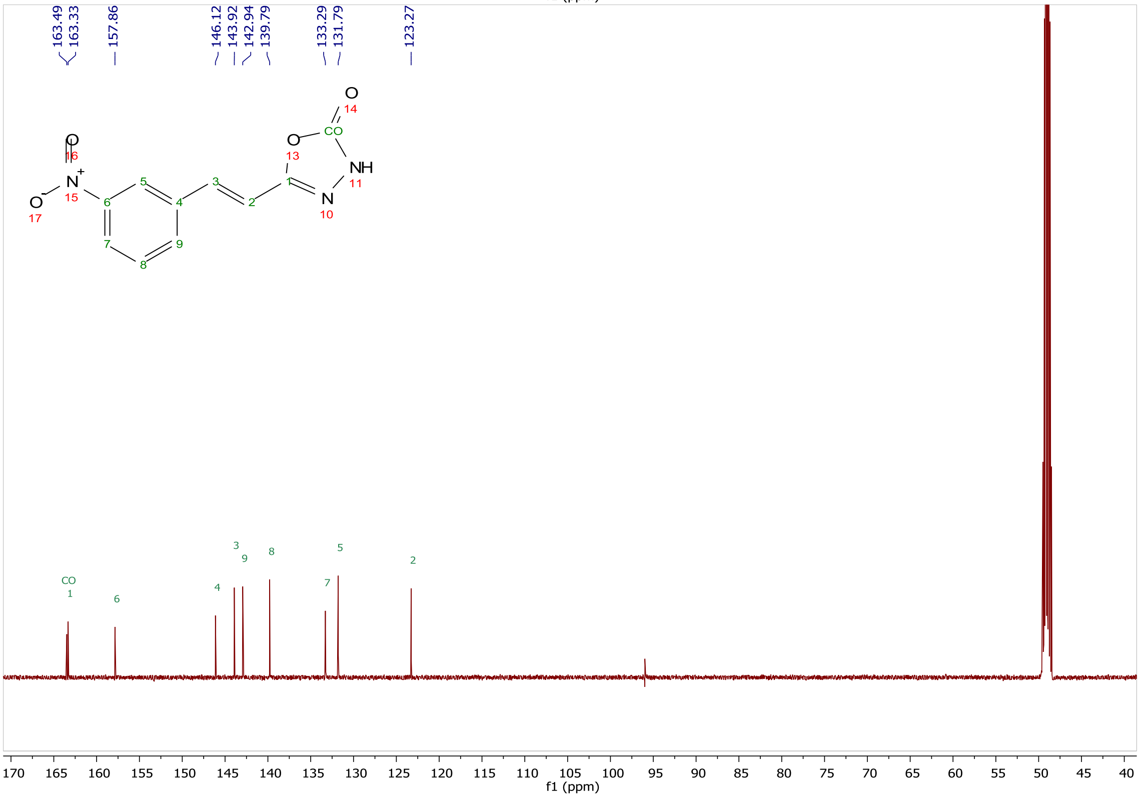

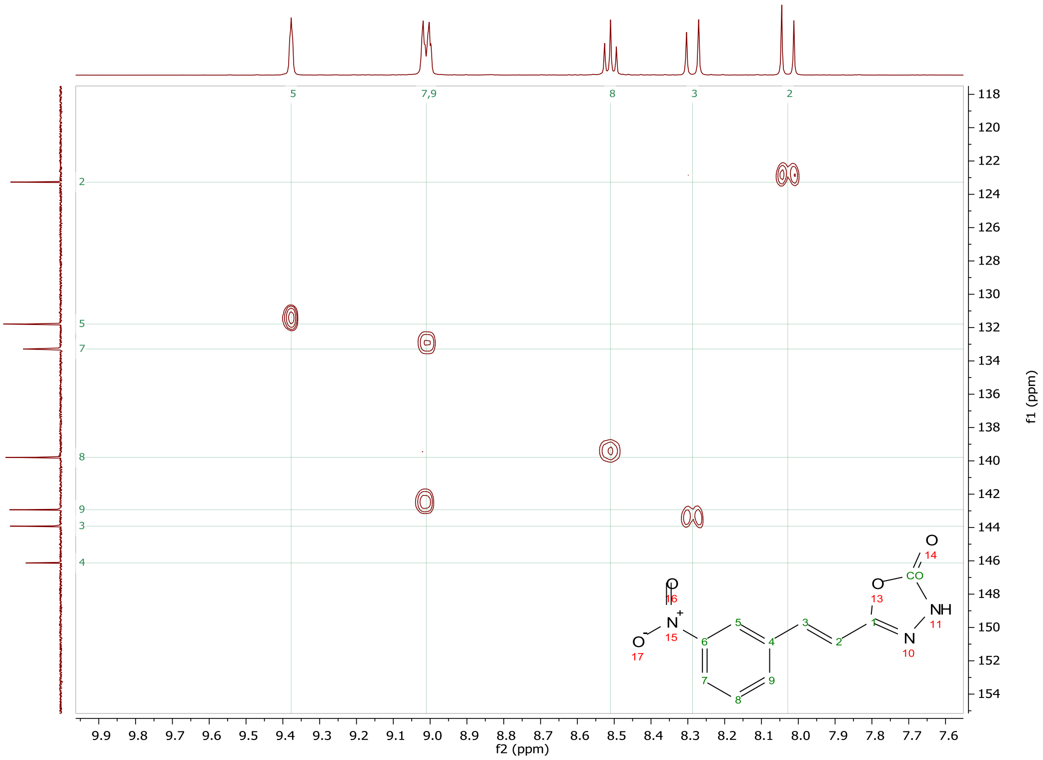
5
7,9
8
3
2
f1 (ppm)
f2 (ppm)
CO
NH
N

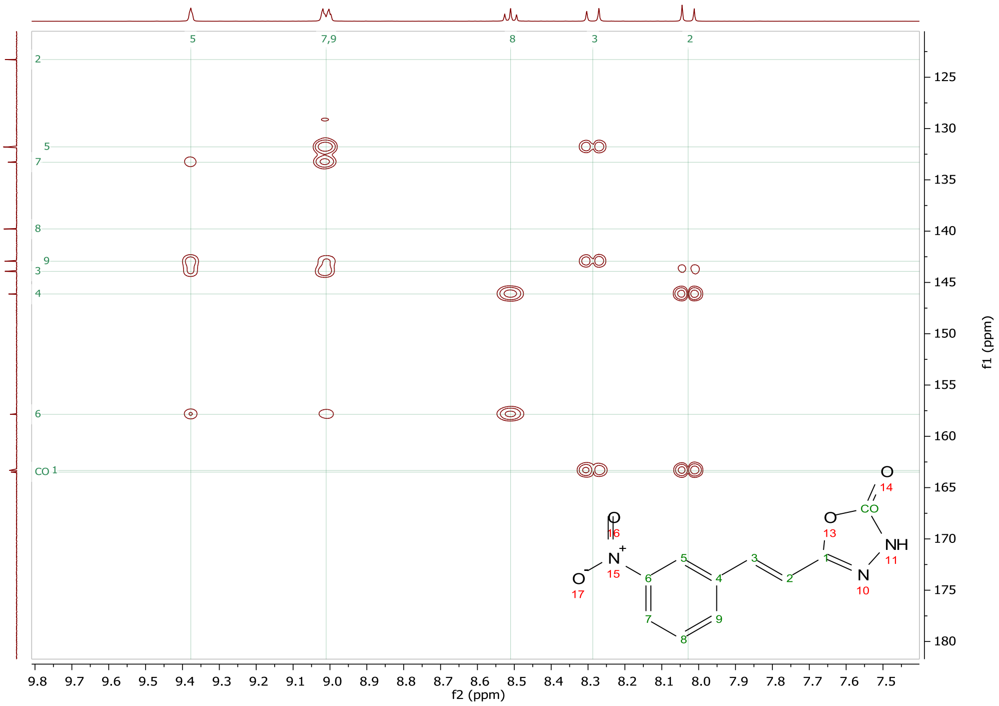
5
7,9
8
3
2
6
CO 1
f1 (ppm)
f2 (ppm)
NH
N

Compound Table

Compound Label	RT	Mass	Abund	Formula	Tgt Mass	Diff (ppm)
Cpd 1: C10 H7 N3 O4	0.22	233.04337	10144	C10 H7 N3 O4	233.04366	-1.22

Compound Label	RT	Algorithm	Mass
Cpd 1: C10 H7 N3 O4	0.22	Find By Formula	233.04337

MS Zoomed Spectrum

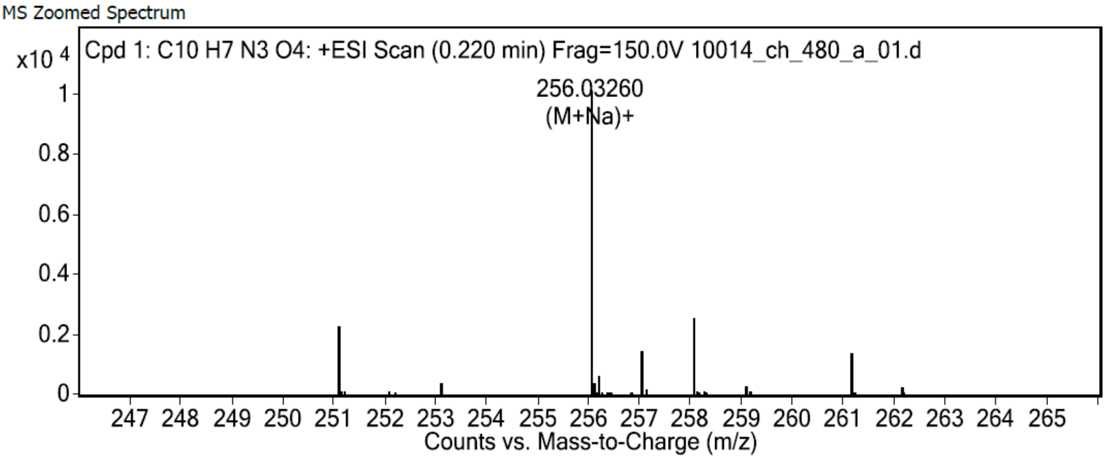

5-[(*E*)-2-(3-methoxyphenyl)ethenyl]-1,3,4-oxadiazol-2(3*H*)-one (2d)

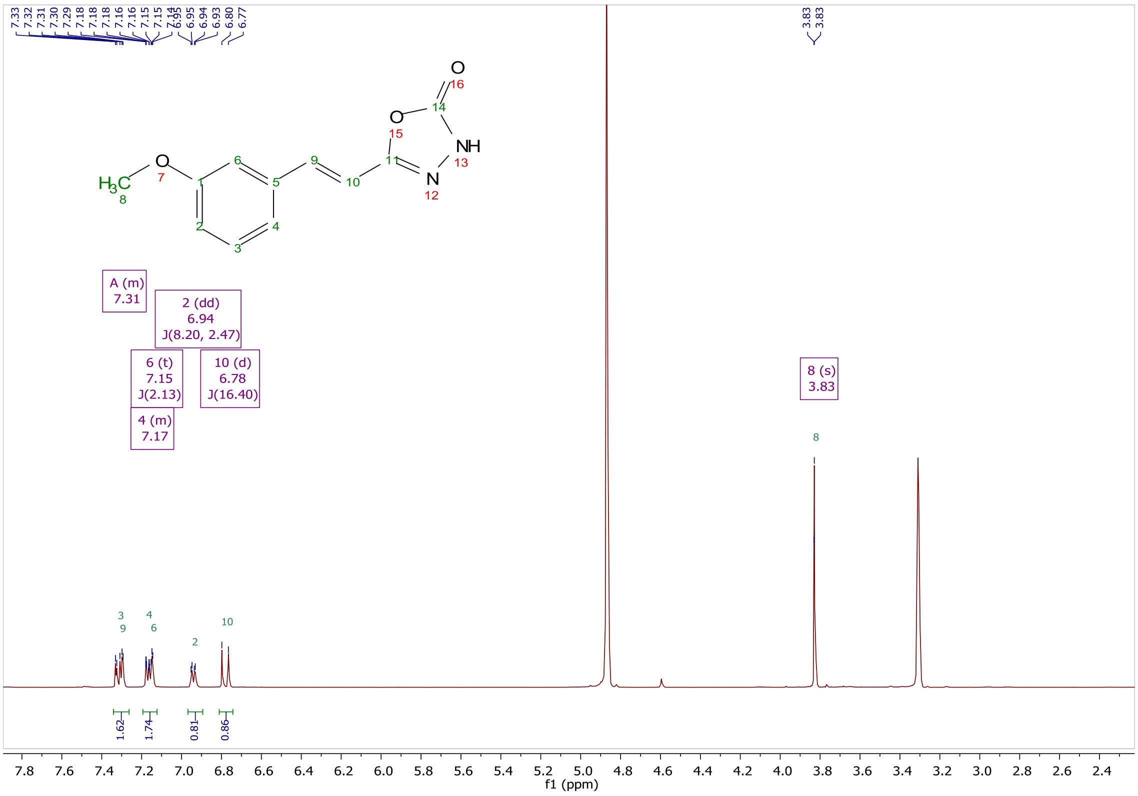

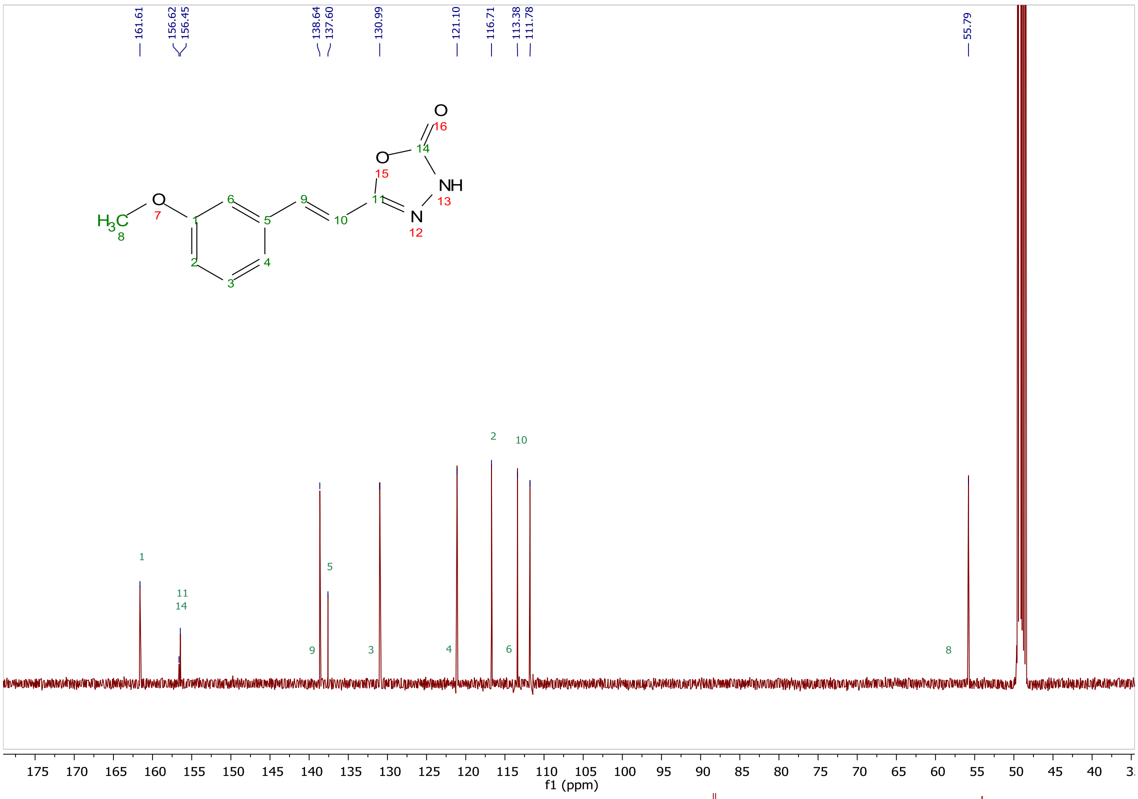
161.61
156.62
156.45
138.64
137.60
130.99
121.10
116.71
113.38
111.78
55.79
f1 (ppm)

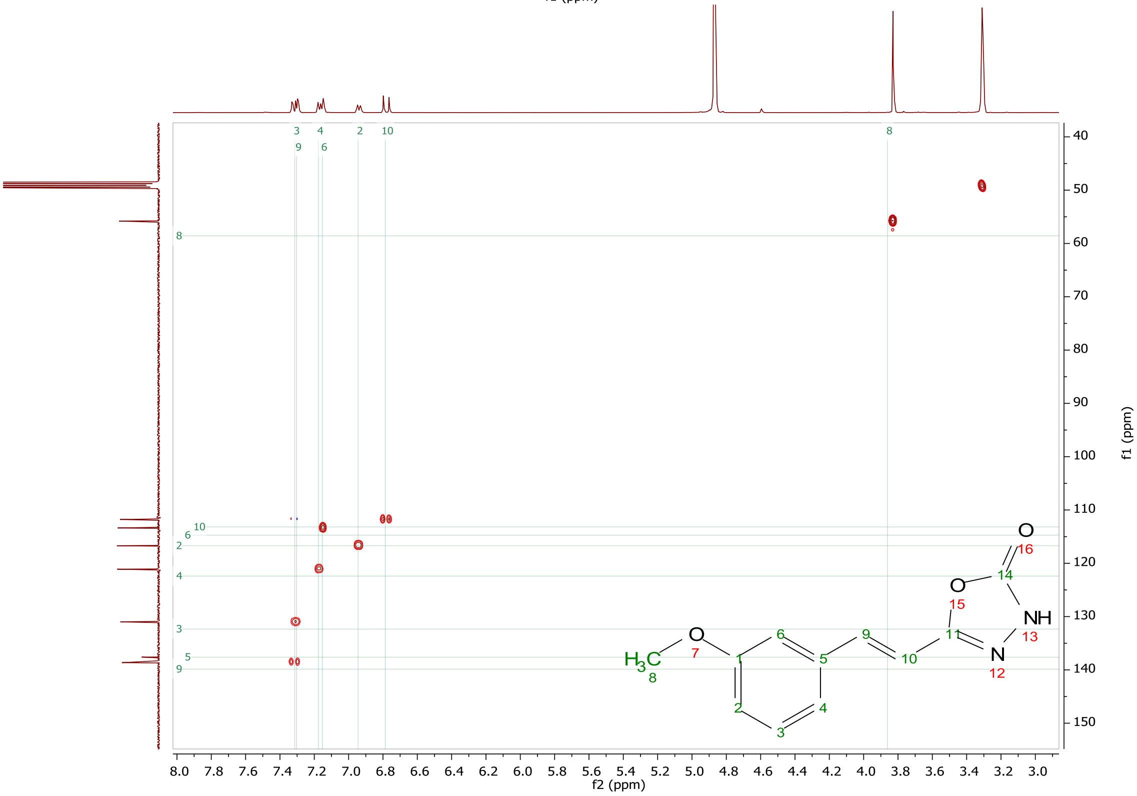
f2 (ppm)
f1 (ppm)

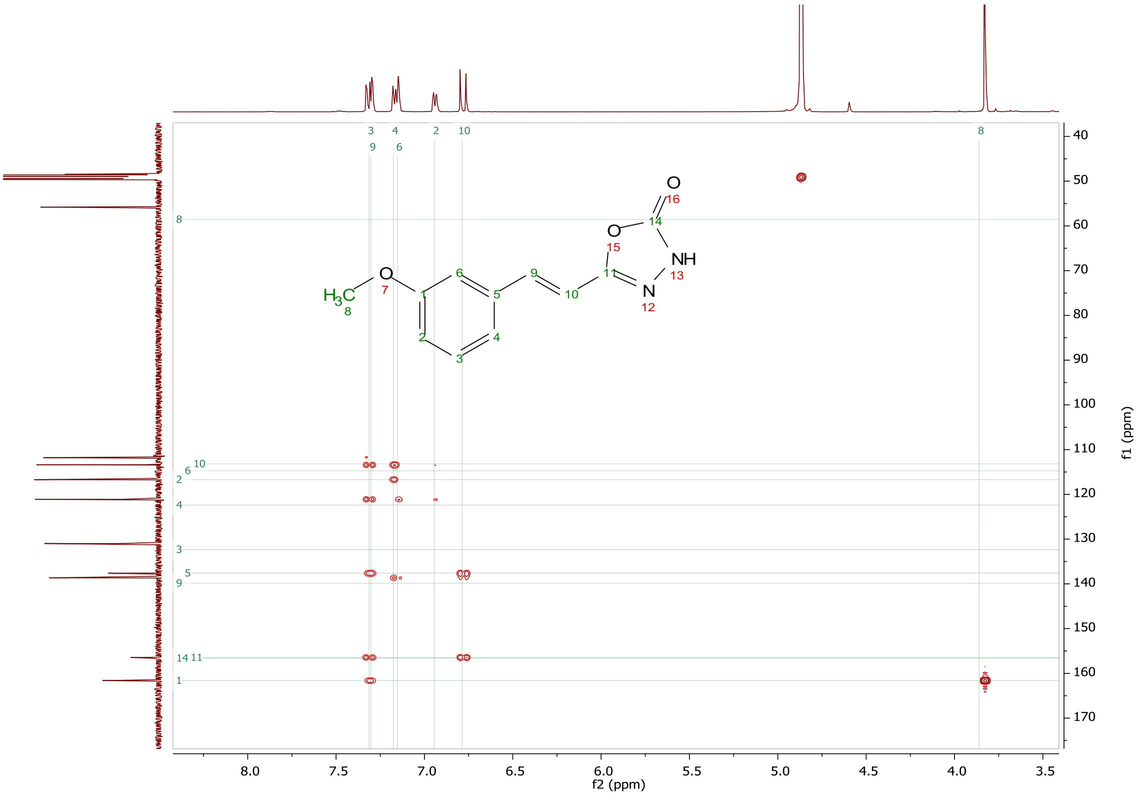

Compound Table

Compound Label	RT	Mass	Abund	Formula	Tgt Mass	Diff (ppm)
Cpd 1: C11 H10 N2 O3	0.264	218.06887	11648	C11 H10 N2 O3	218.06914	-1.23

Compound Label	RT	Algorithm	Mass
Cpd 1: C11 H10 N2 O3	0.264	Find By Formula	218.06887

MS Zoomed Spectrum

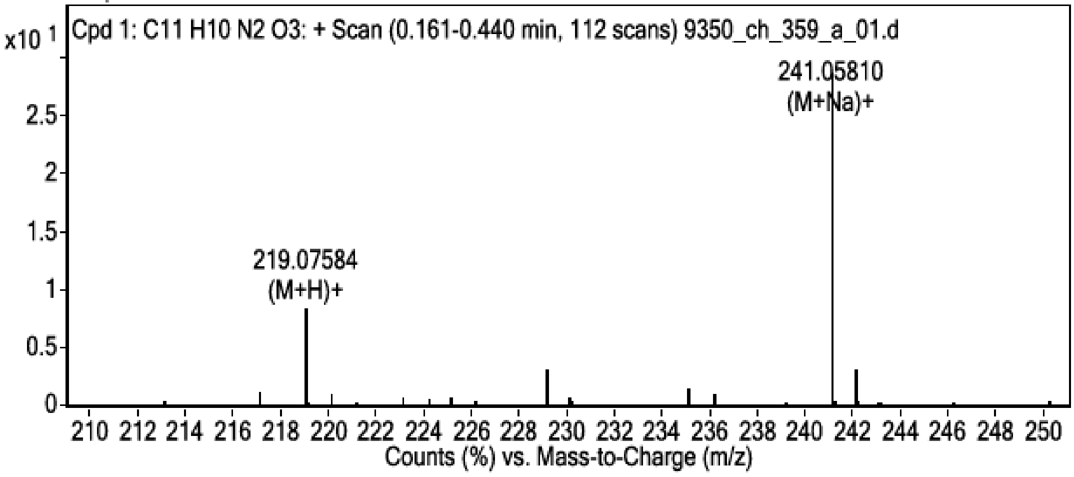

5-[(*E*)-2-(4-methoxyphenyl)ethenyl]-1,3,4-oxadiazol-2(3*H*)-one (2e)

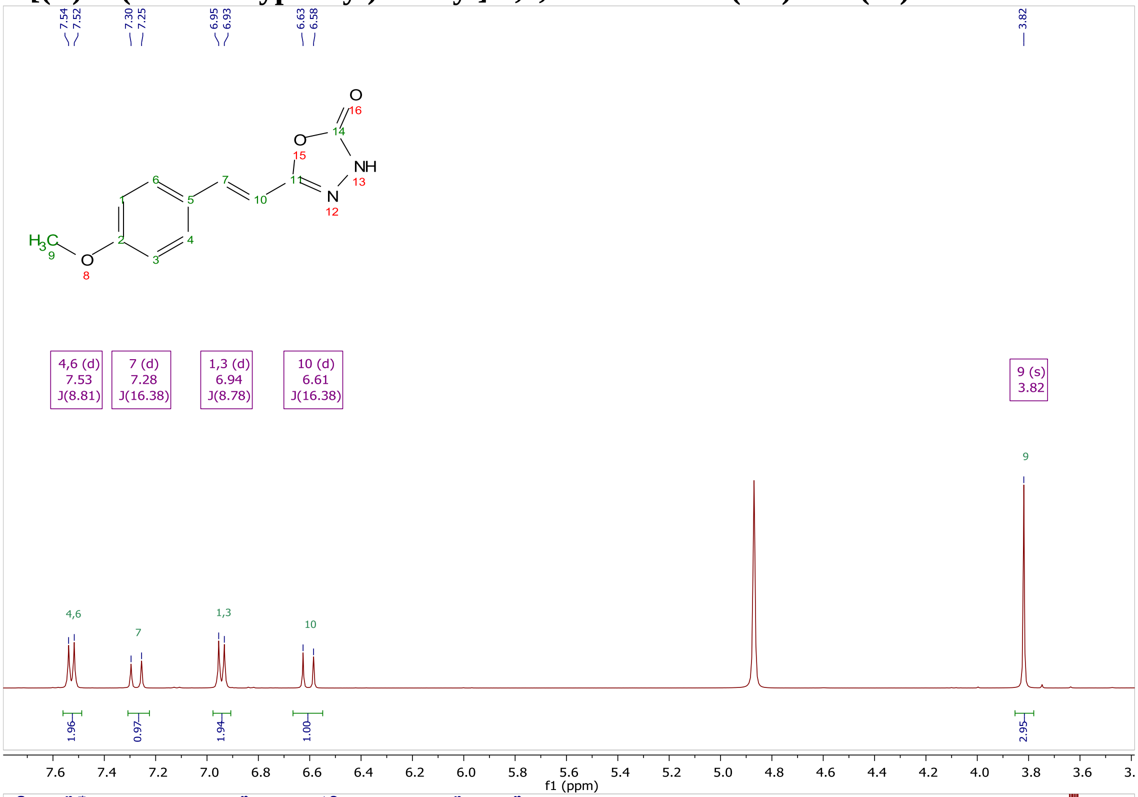

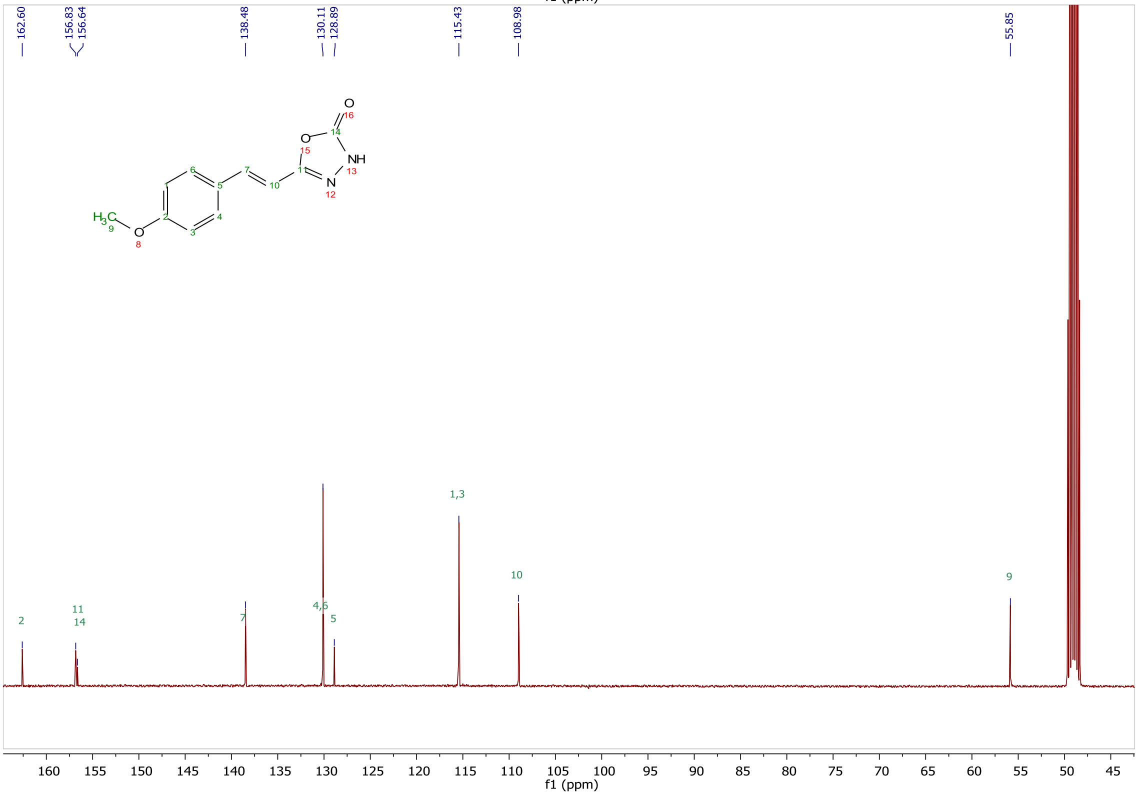

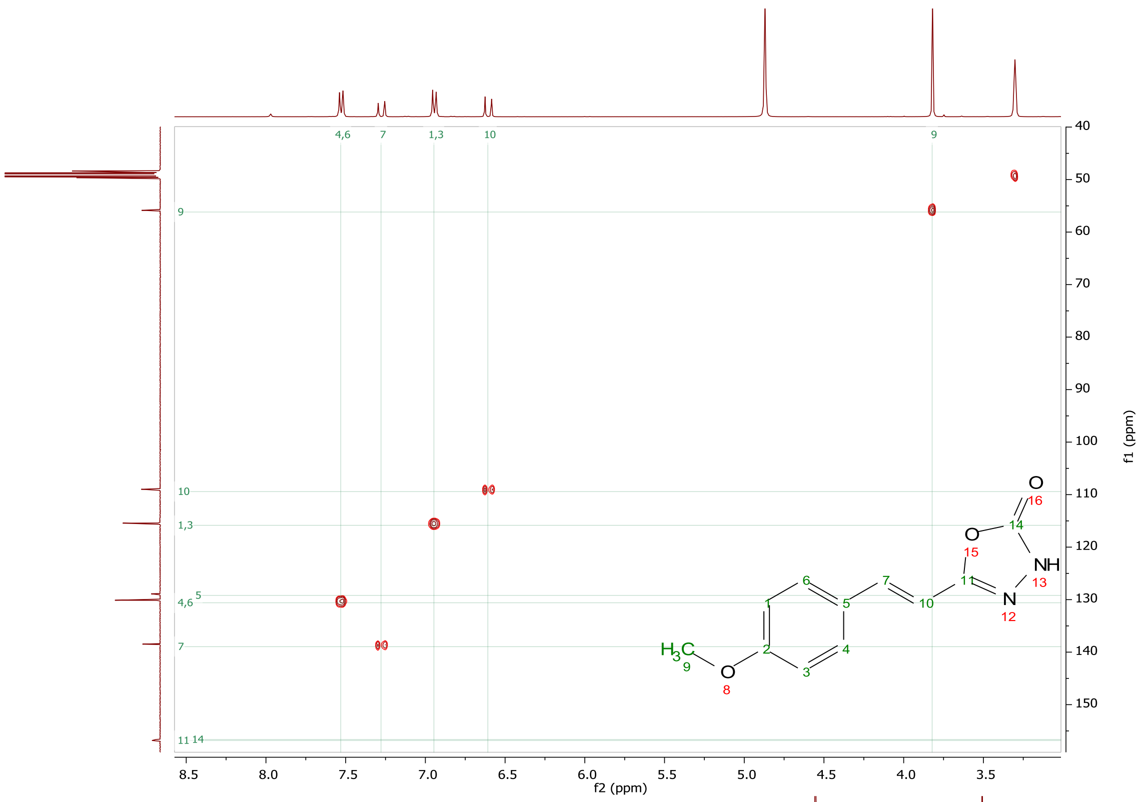
4,6
7
1,3
10
9
9
10
1,3
4,6
5
7
11 14
f1 (ppm)
f2 (ppm)
H3C
O
8
16
14
15
NH
13
N
12

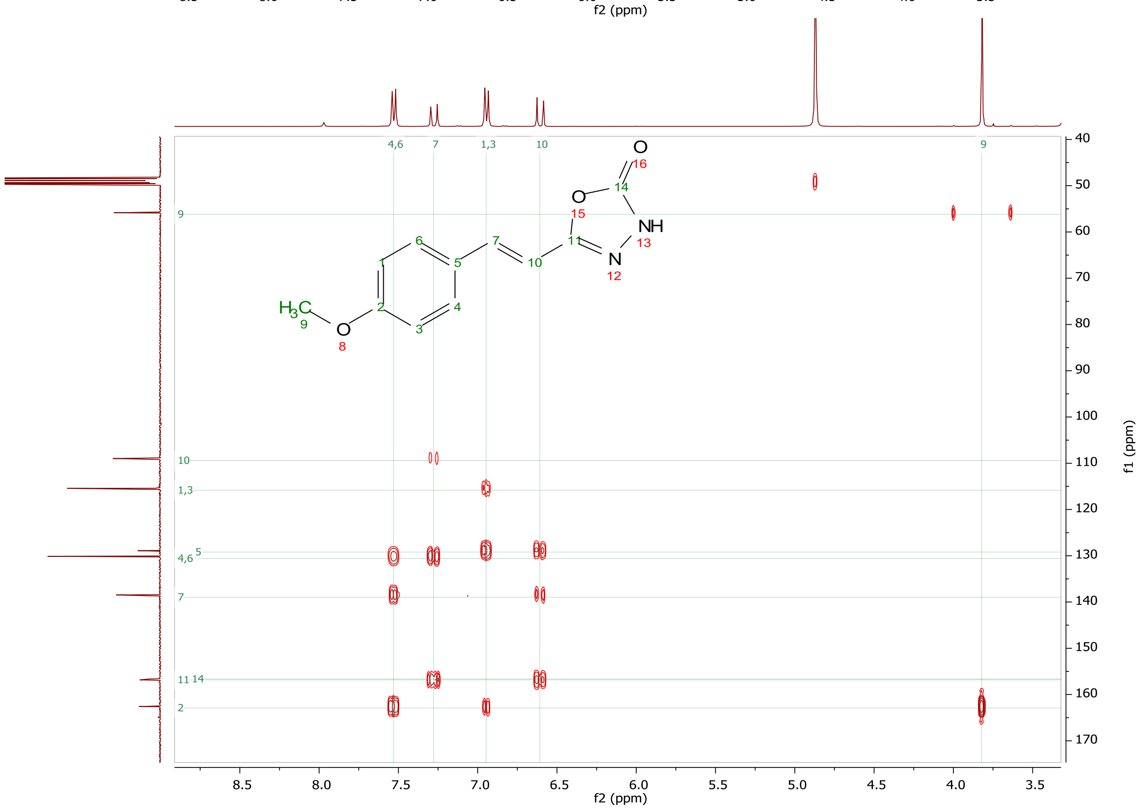
4,6
7
1,3
10
9
9
10
1,3
4,6
5
7
11 14
2
f1 (ppm)
f2 (ppm)
H3C
O
8
16
14
15
NH
13
N
12

Compound Table

Compound Label	RT	Mass	Abund	Formula	Tgt Mass	Diff (ppm)
Cpd 1: C11 H10 N2 O3	0.267	218.06832	45761	C11 H10 N2 O3	218.06914	-3.76

Compound Label	RT	Algorithm	Mass
Cpd 1: C11 H10 N2 O3	0.267	Find By Formula	218.06832

MS Zoomed Spectrum

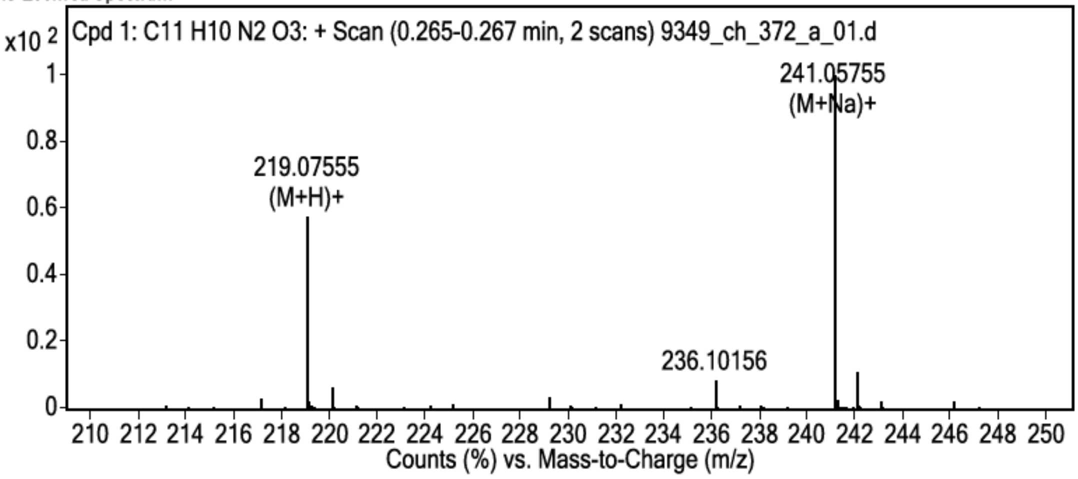

5-[(*E*)-2-(2,4-Dimethoxyphenyl)ethenyl]-1,3,4-oxadiazol-2(3*H*)-one (2f)

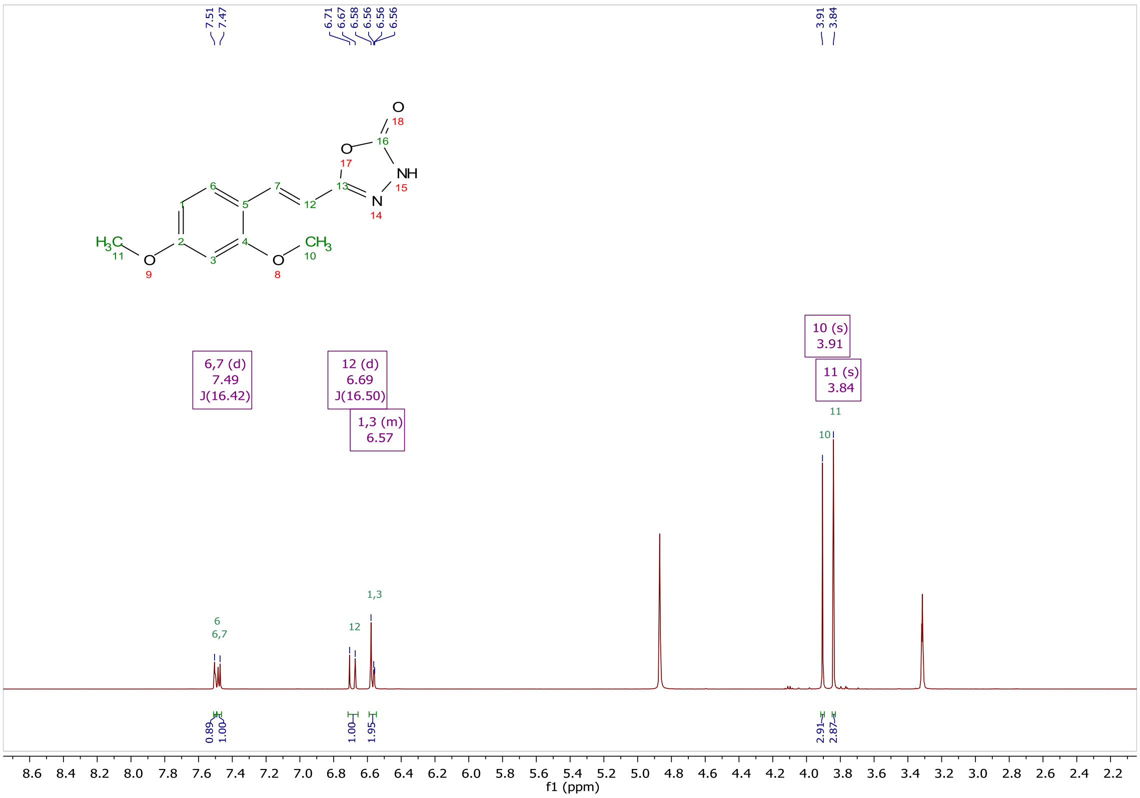

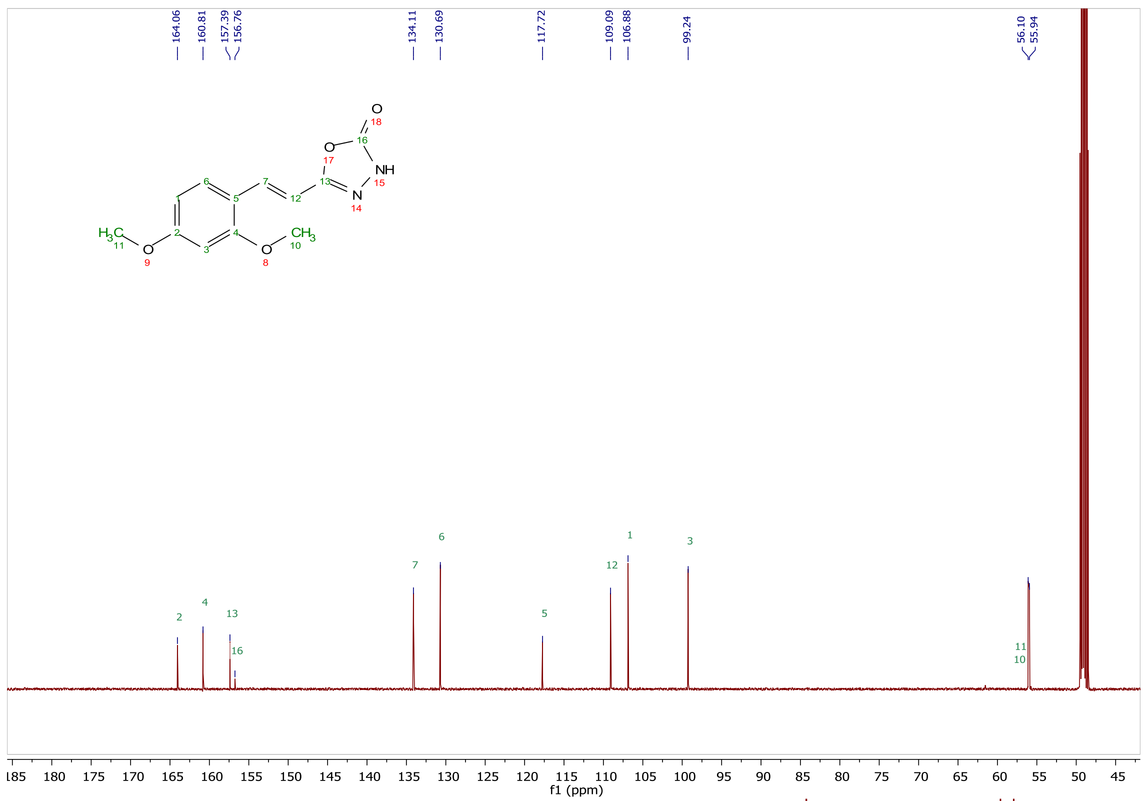
164.06
160.81
157.39
156.76
134.11
130.69
117.72
109.09
106.88
99.24
56.10
55.94
f1 (ppm)

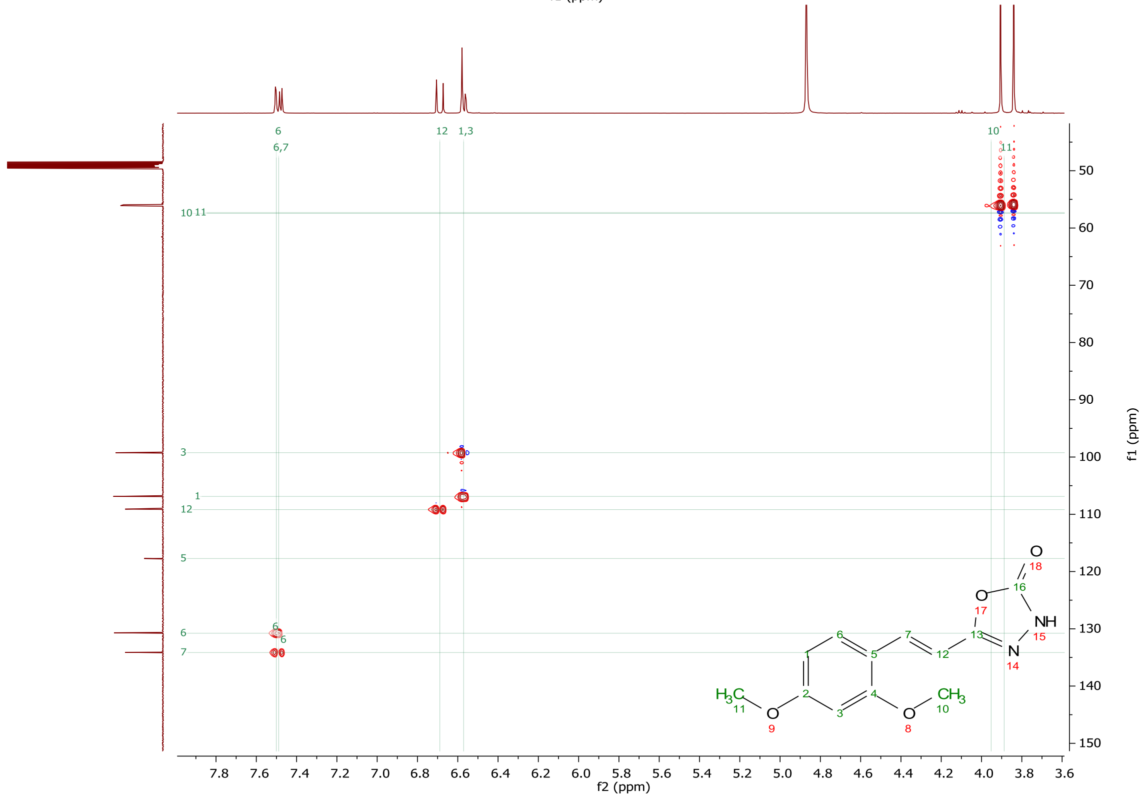
f2 (ppm)
f1 (ppm)

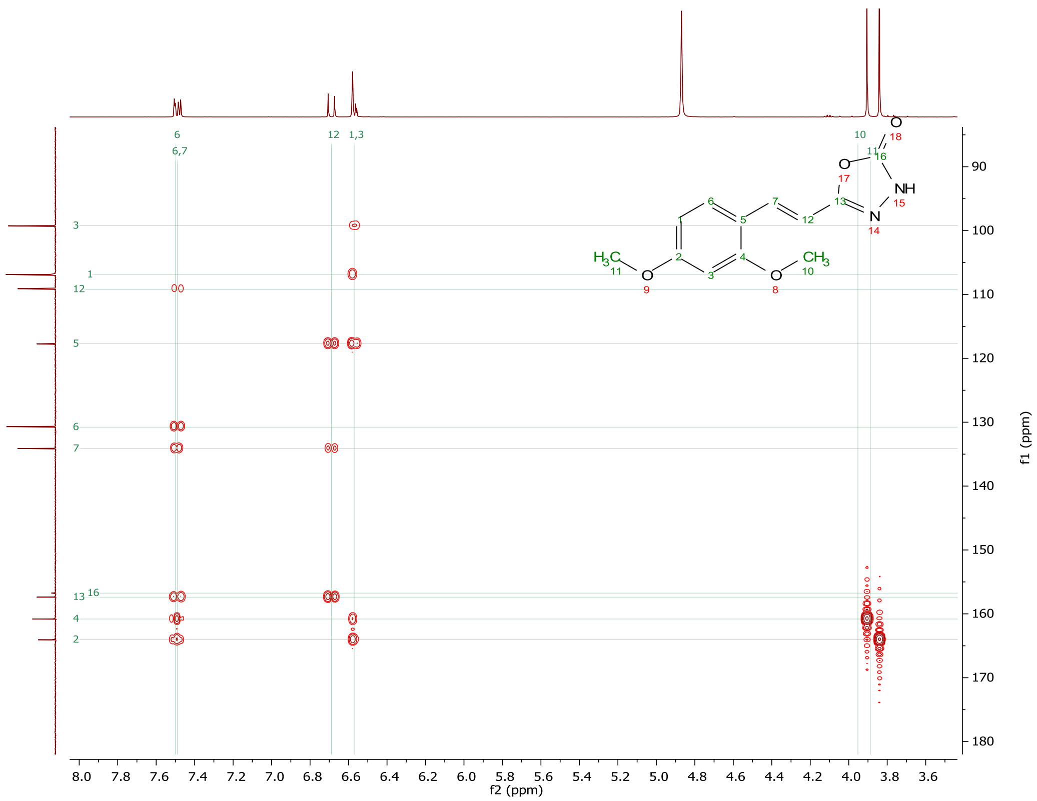

Compound Table

Compound Label	RT	Mass	Abund	Formula	Tgt Mass	Diff (ppm)
Cpd 1: C12 H12 N2 O4	0.265	248.07922	92555	C12 H12 N2 O4	248.07971	-1.97

Compound Label	RT	Algorithm	Mass
Cpd 1: C12 H12 N2 O4	0.265	Find By Formula	248.07922

MS Zoomed Spectrum

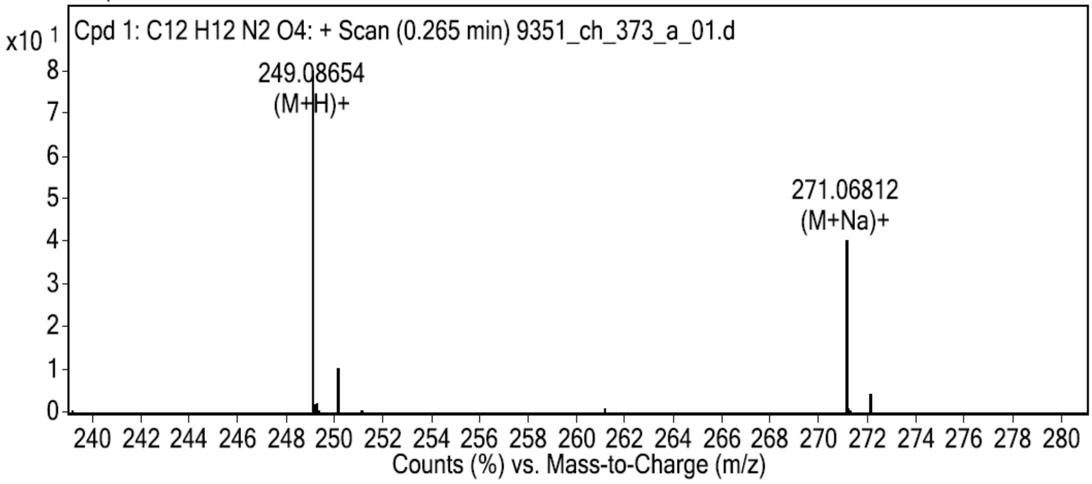

5-[(*E*)-2-(2,5-Dimethoxyphenyl)ethenyl]-1,3,4-oxadiazol-2(3*H*)-one (2g)

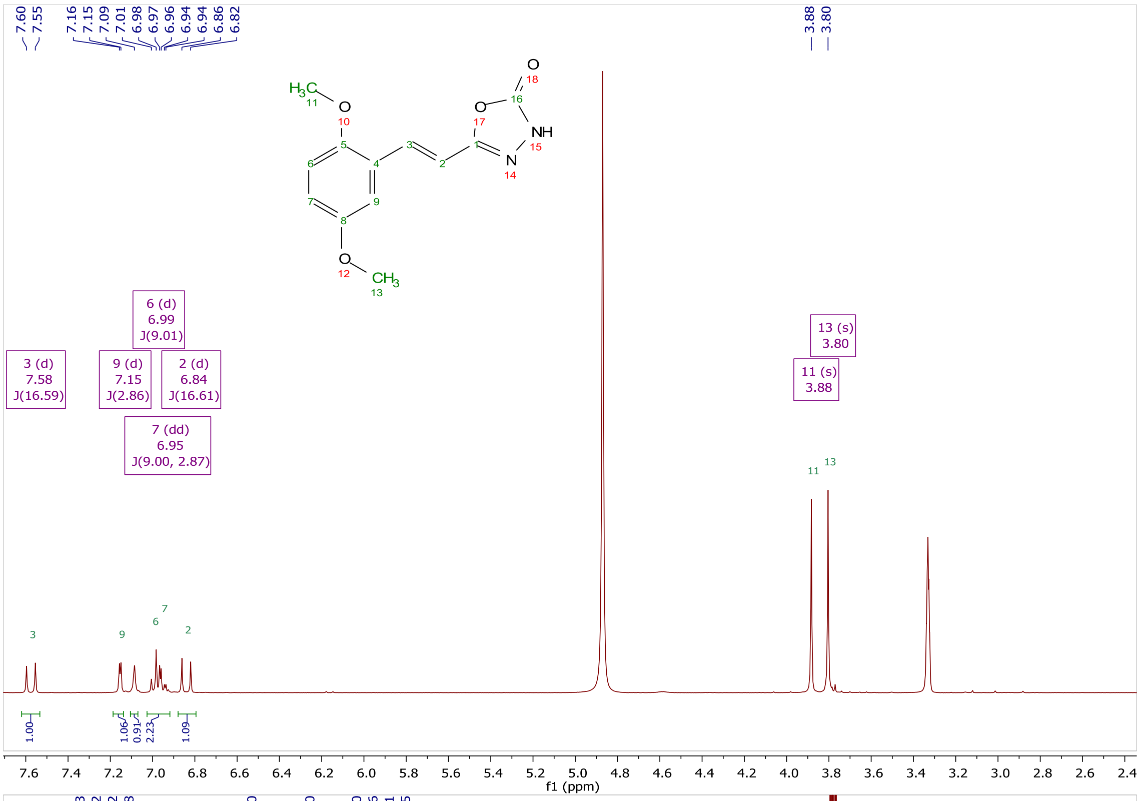

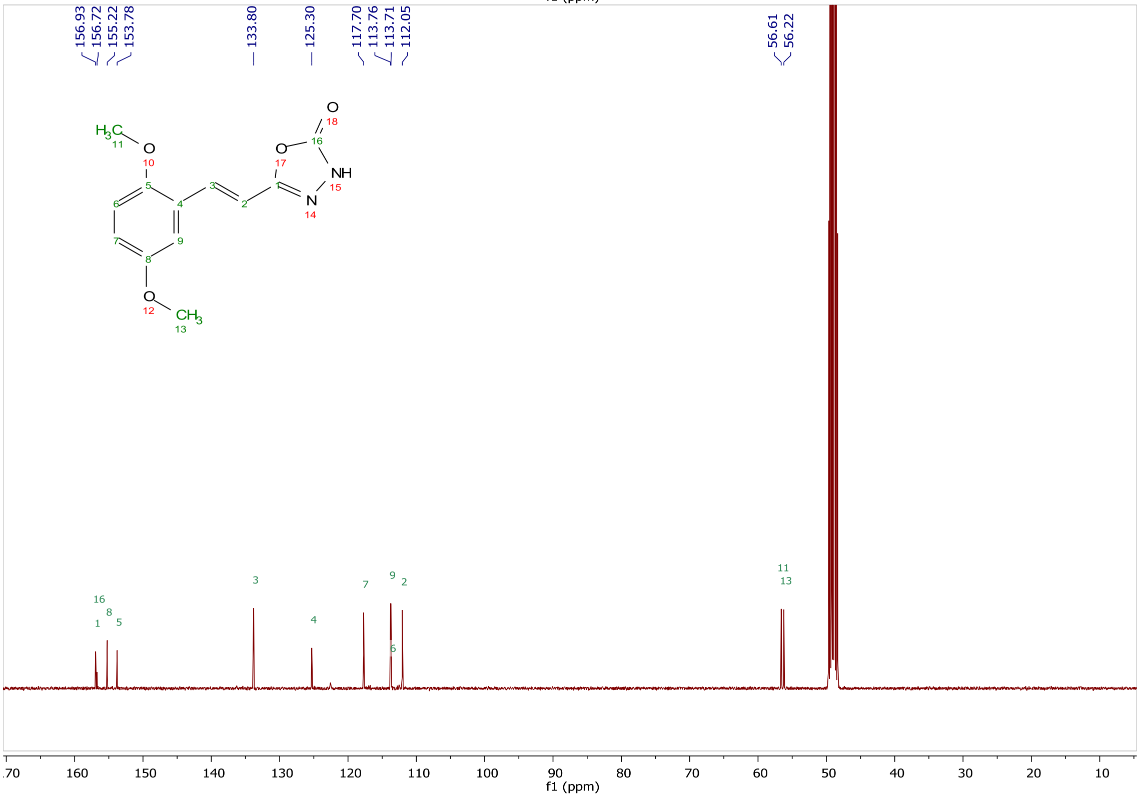

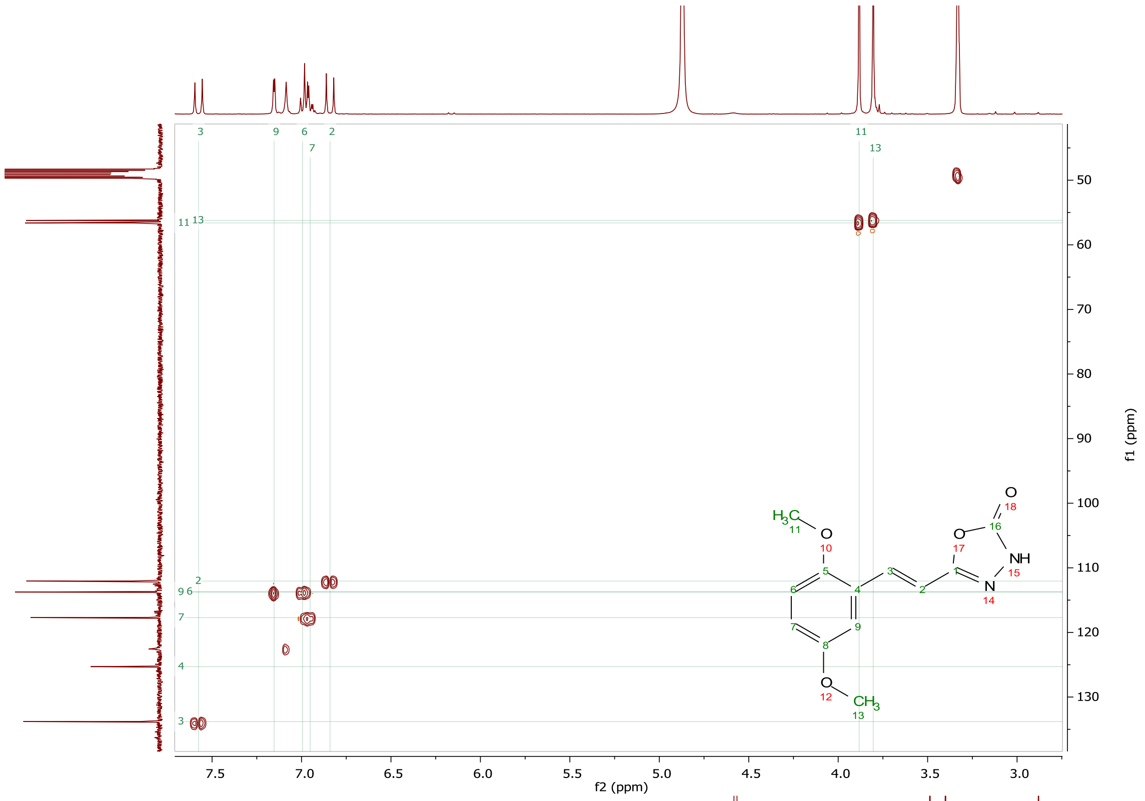
f1 (ppm)
f2 (ppm)
H_3C
O
NH
N
CH_3

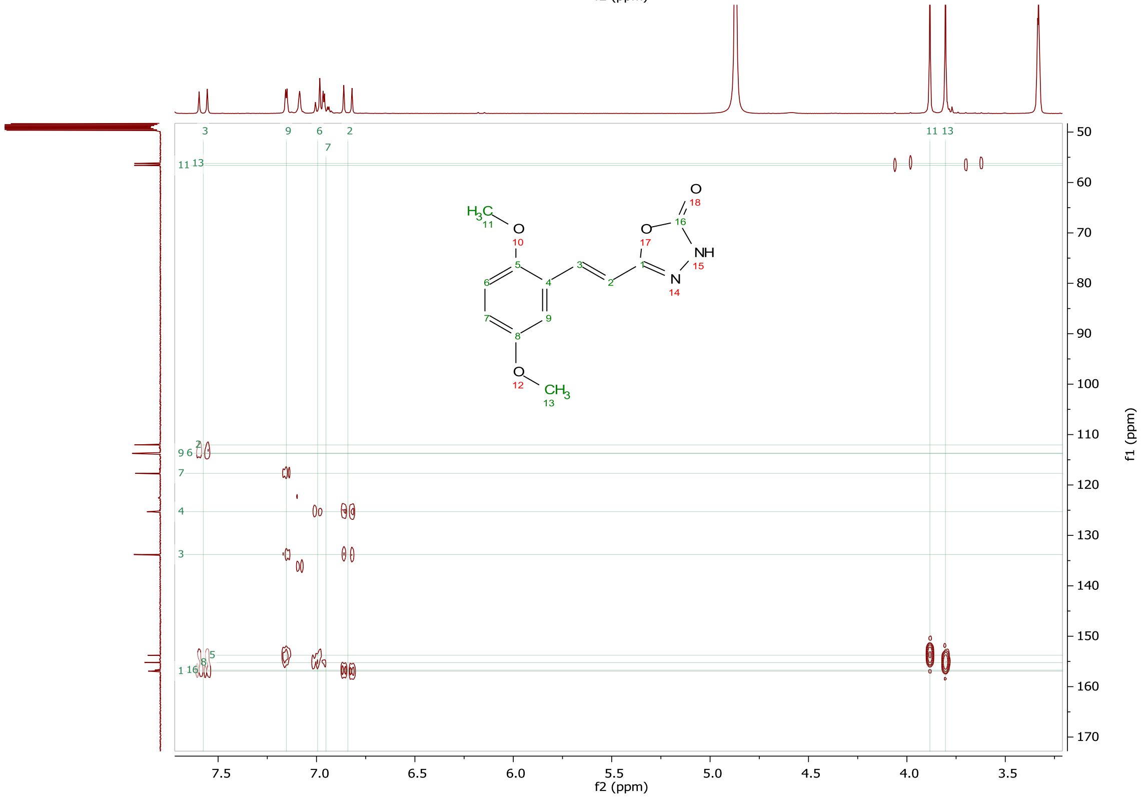
f1 (ppm)
f2 (ppm)
H_3C
O
NH
N
CH_3

Compound Table

Compound Label	RT	Mass	Abund	Formula	Tgt Mass	Diff (ppm)
Cpd 1: C12 H12 N2 O4	0.49	248.08094	11976	C12 H12 N2 O4	248.07971	4.95

Compound Label	RT	Algorithm	Mass
Cpd 1: C12 H12 N2 O4	0.49	Find By Formula	248.08094

MS Zoomed Spectrum

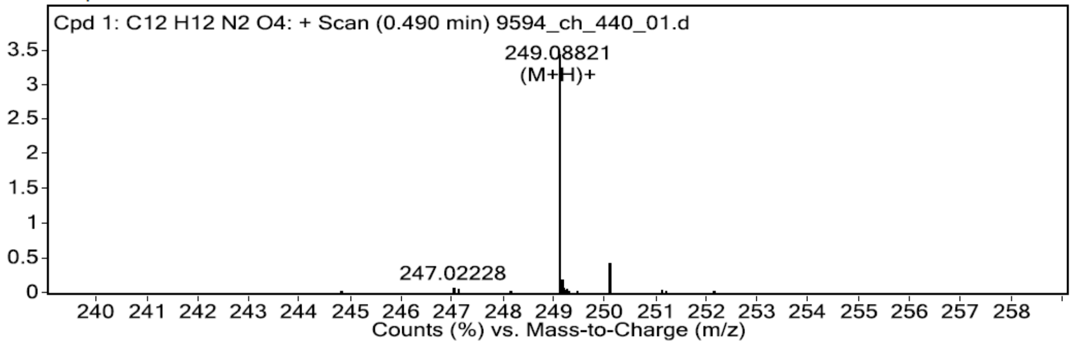

5-[(E)-2-(2H-1,3-Benzodioxol-5-yl)ethenyl]-1,3,4-oxadiazol-2(3H)-one (2i)

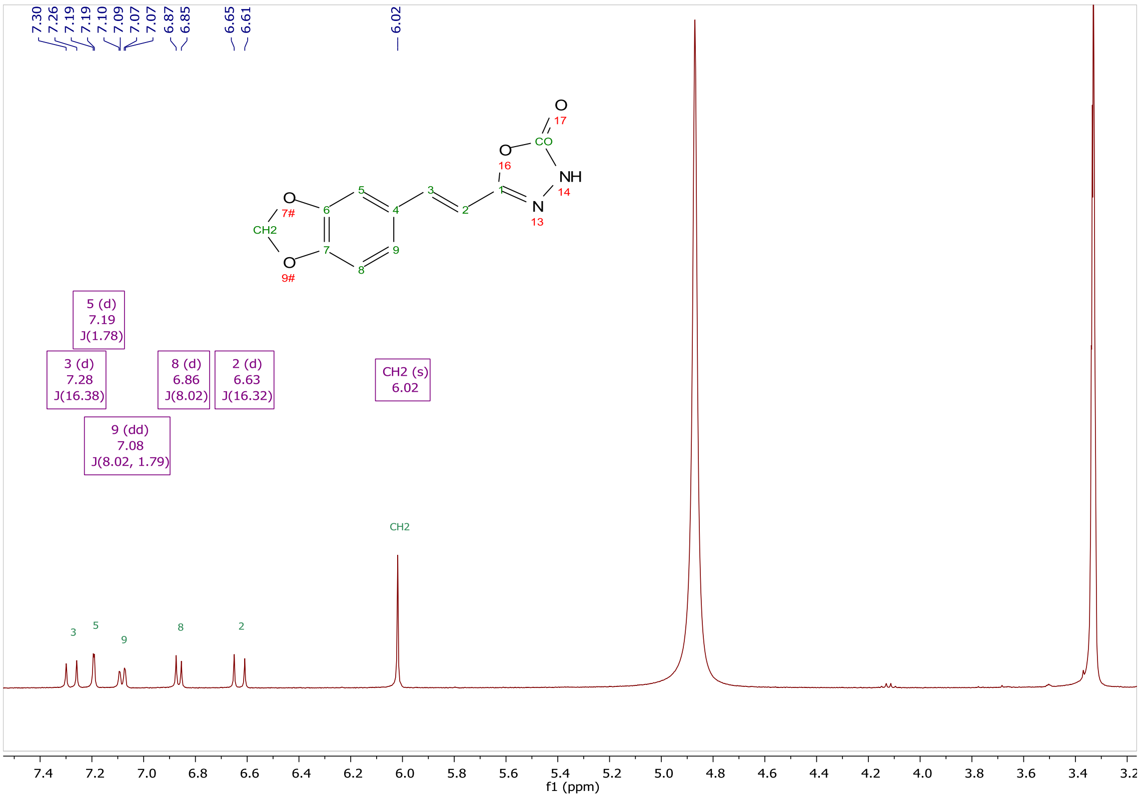

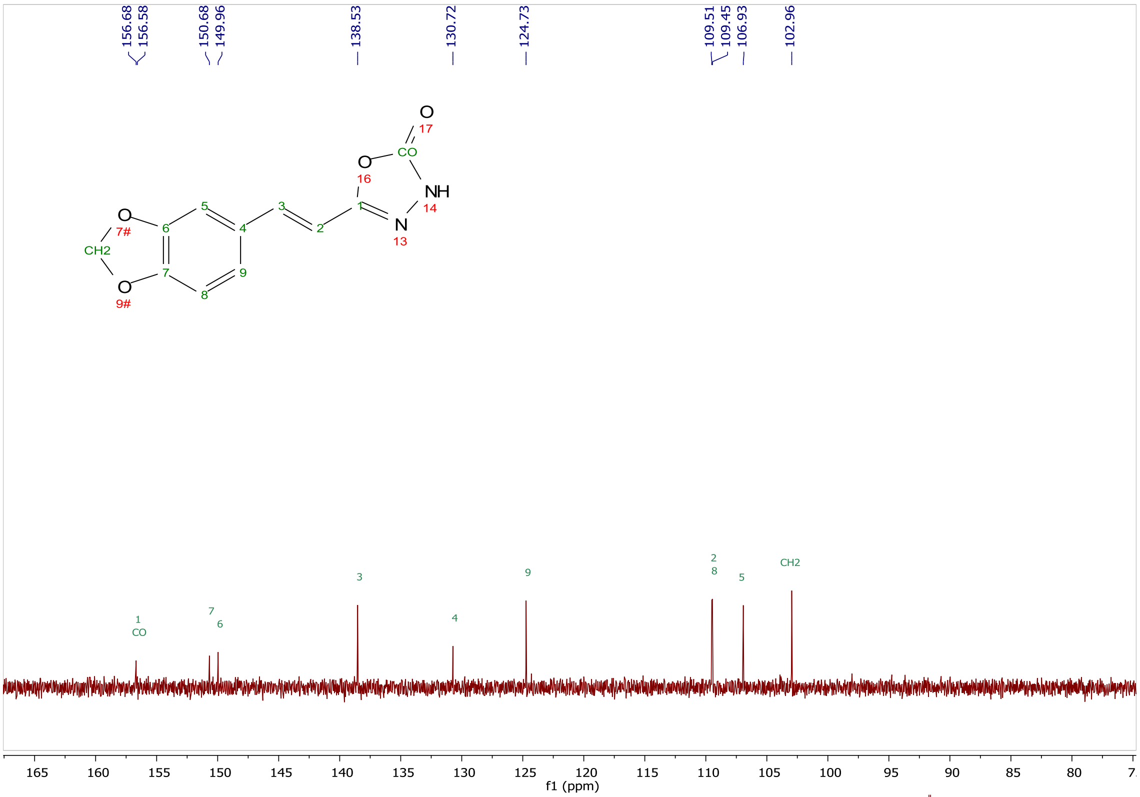
156.68
156.58
150.68
149.96
138.53
130.72
124.73
109.51
109.45
106.93
102.96
f1 (ppm)

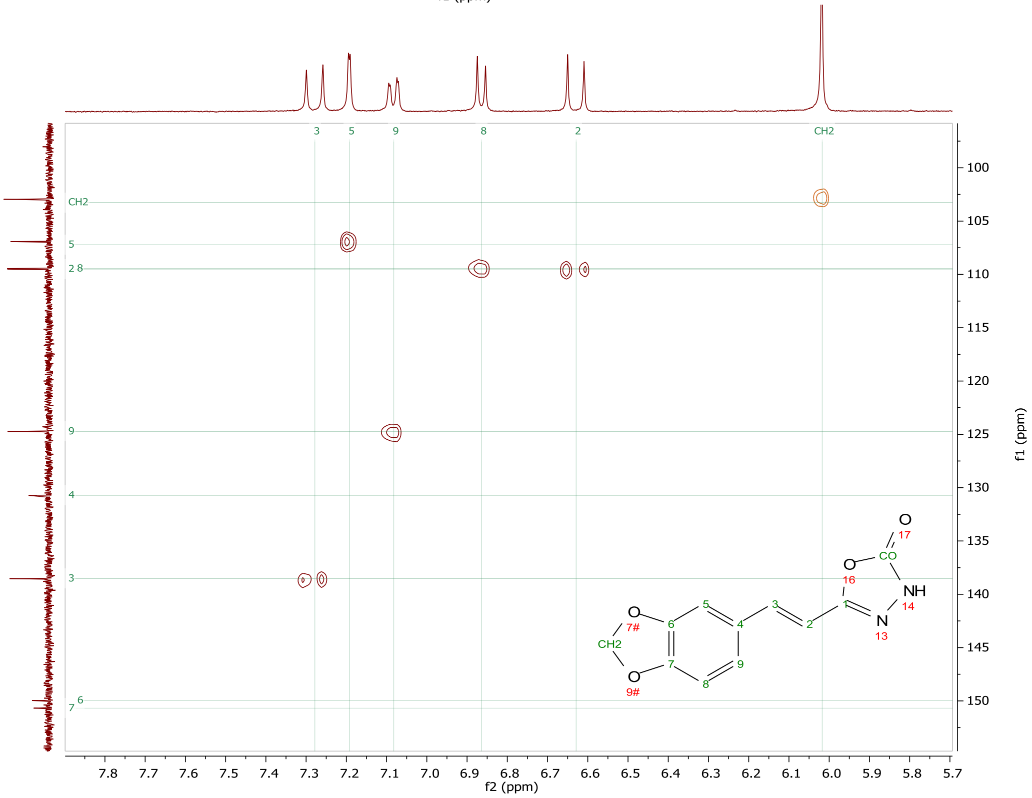
f2 (ppm)
f1 (ppm)

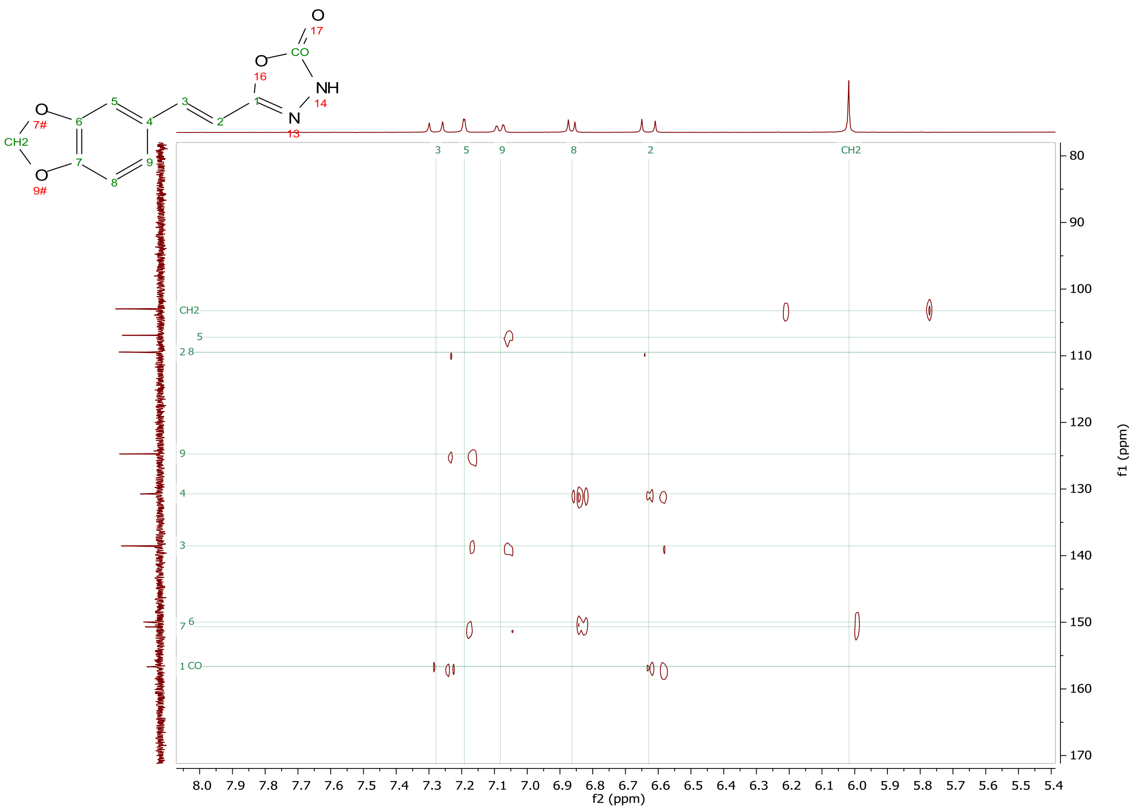

Compound Table

Compound Label	RT	Mass	Abund	Formula	Tgt Mass	Diff (ppm)
Cpd 1: C11 H8 N2 O4	0.263	232.04873	164395	C11 H8 N2 O4	232.04841	1.37

Compound Label	RT	Algorithm	Mass
Cpd 1: C11 H8 N2 O4	0.263	Find By Formula	232.04873

MS Zoomed Spectrum

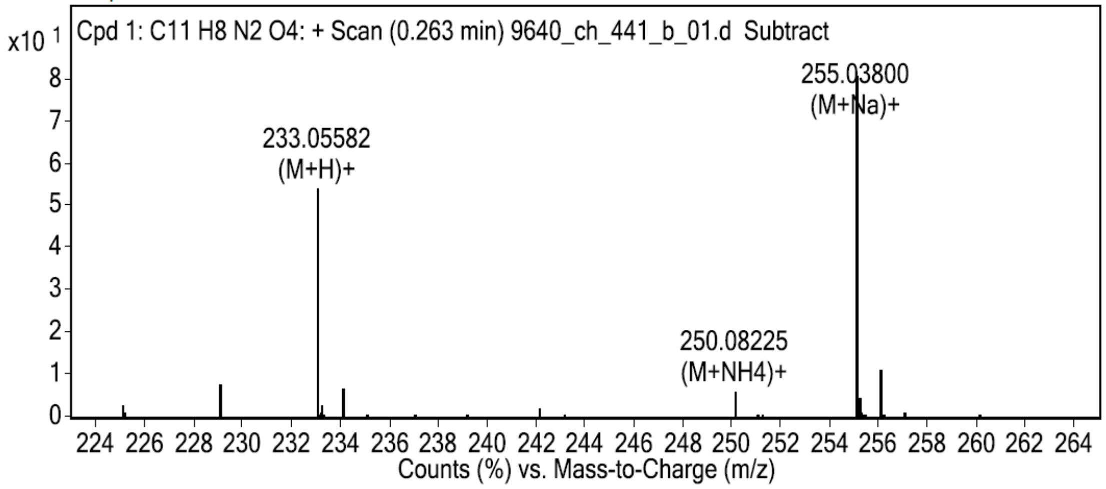

5-[(E)-2-(3,5-Dimethoxyphenyl)ethenyl]-1,3,4-oxadiazol-2(3H)-one (2j)

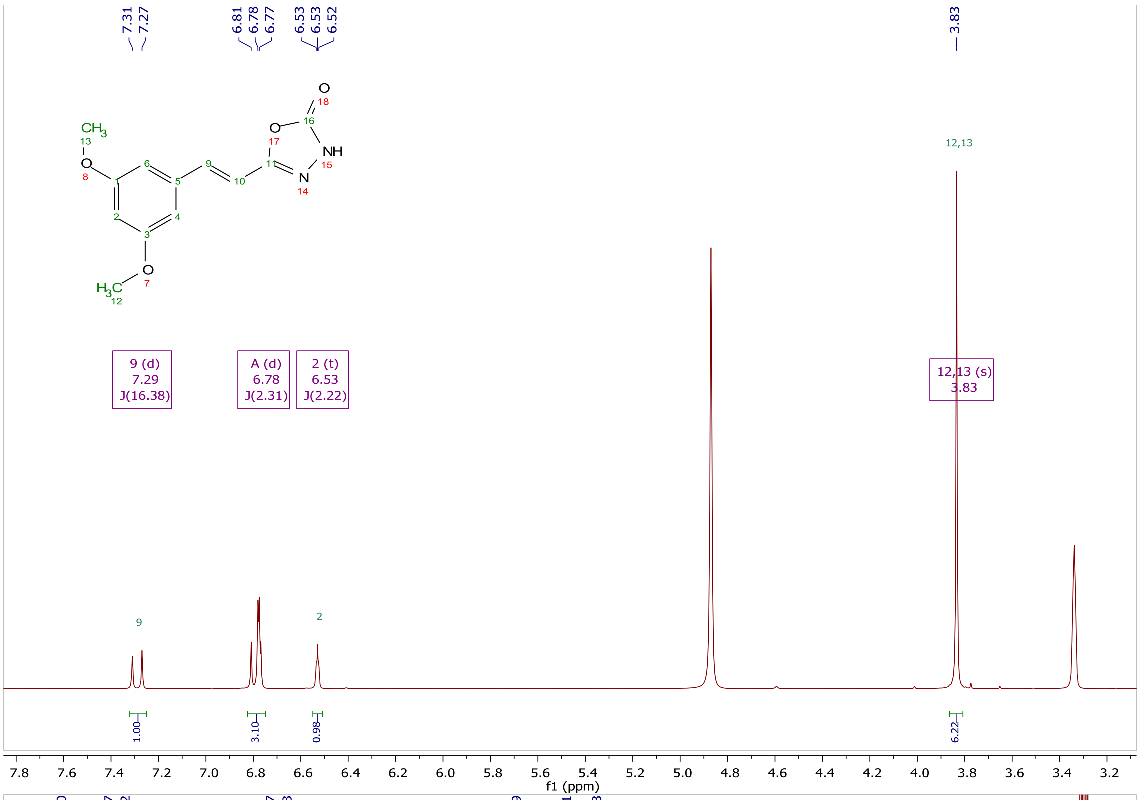

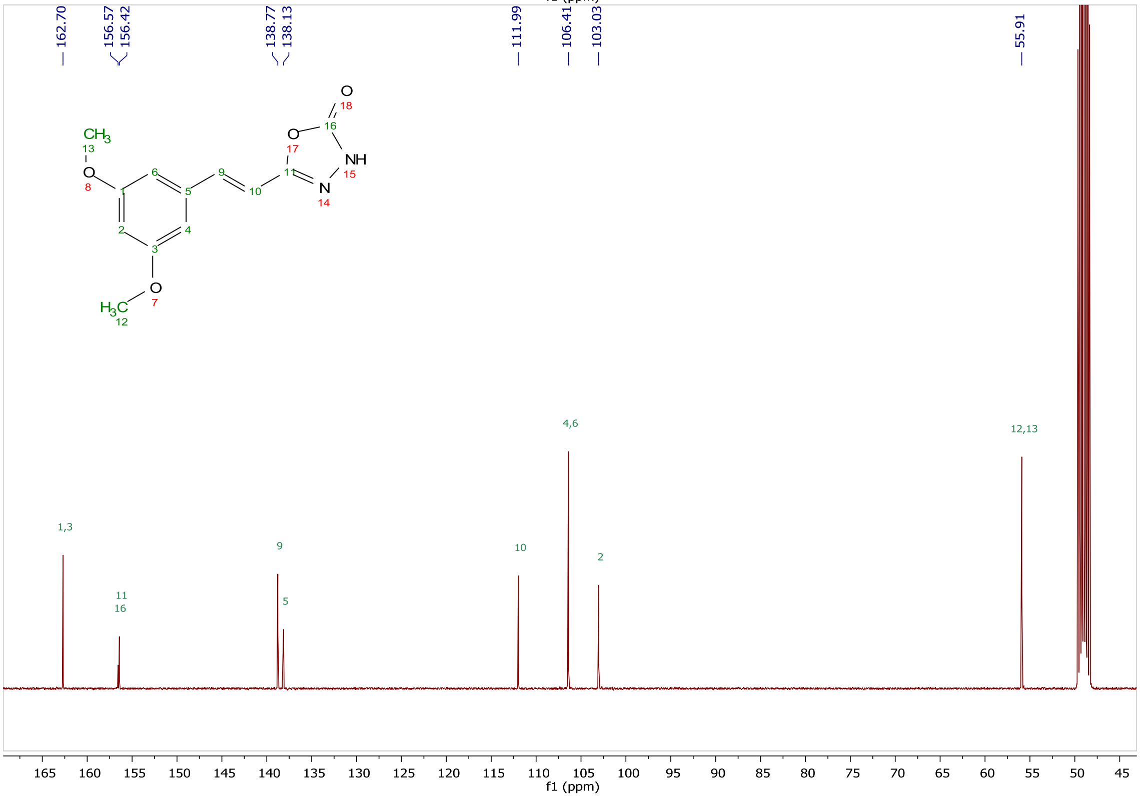

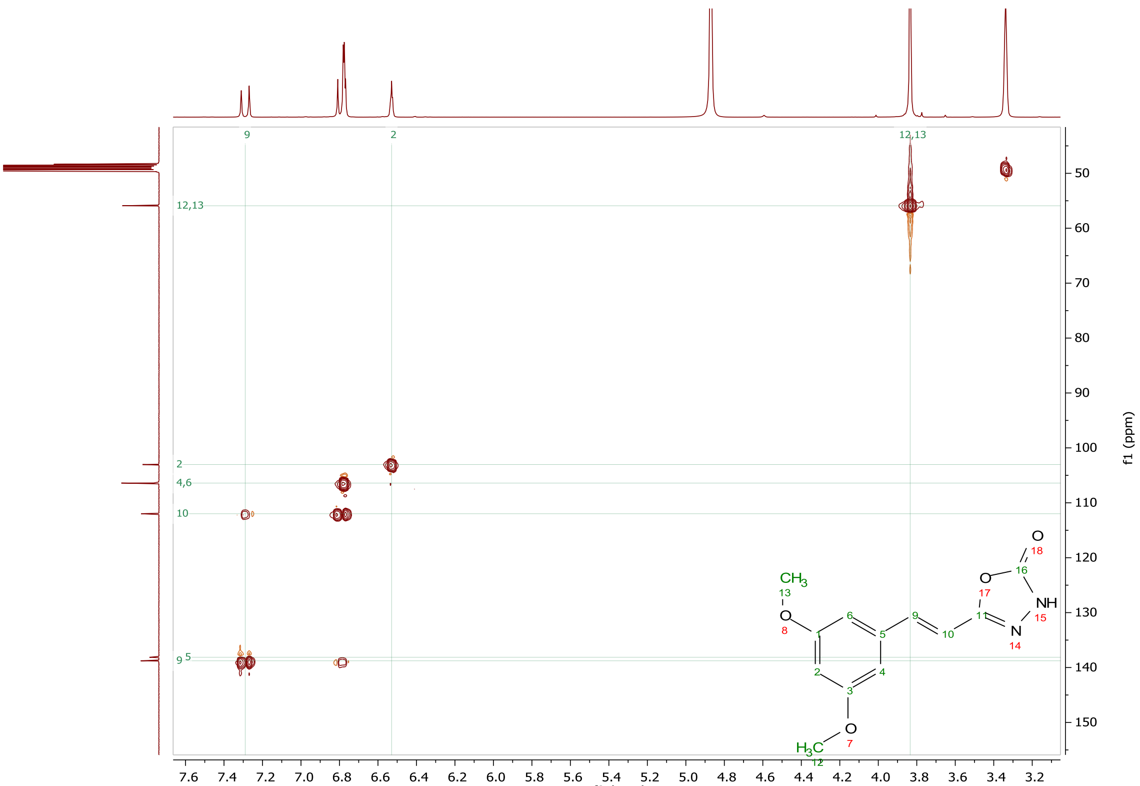
9
2
12,13
12,13
2
4,6
10
9 5
f2 (ppm)
f1 (ppm)

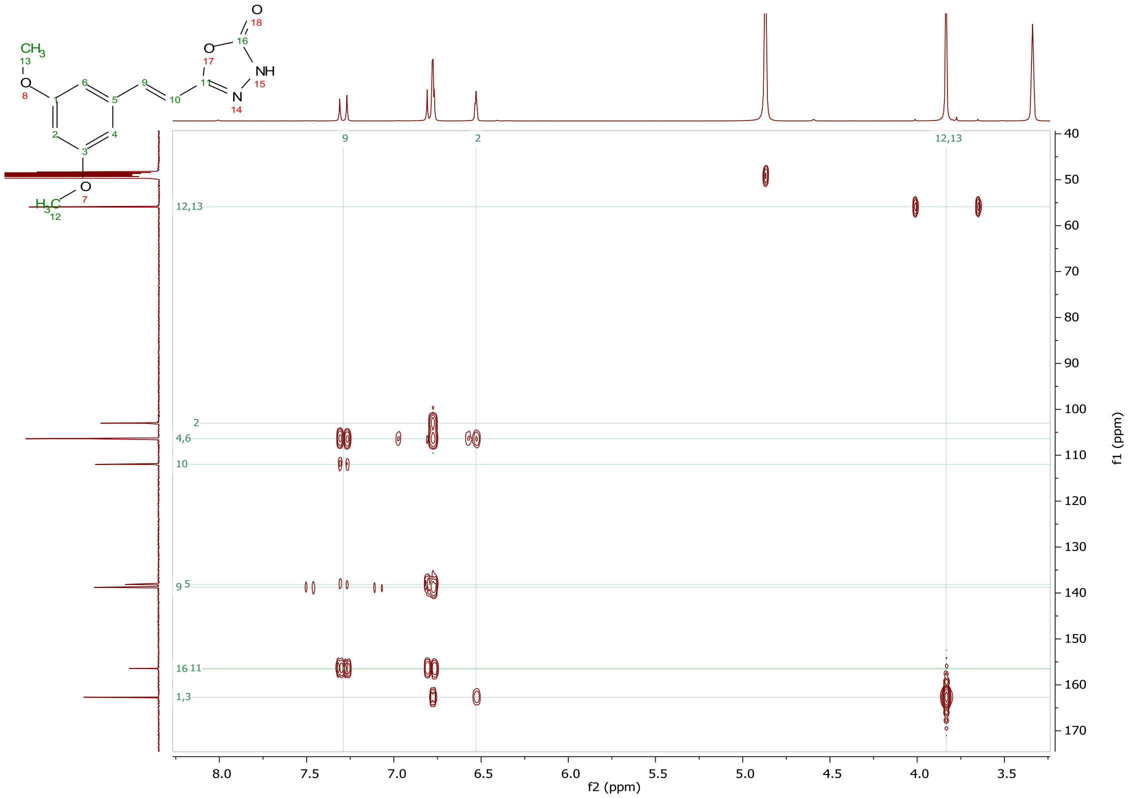
9
2
12,13
12,13
2
4,6
10
9 5
16 11
1,3
f2 (ppm)
f1 (ppm)

Compound Table

Compound Label	RT	Mass	Abund	Formula	Tgt Mass	Diff (ppm)
Cpd 1: C12 H12 N2 O4	0.418	248.08033	5268	C12 H12 N2 O4	248.07971	2.51

Compound Label	RT	Algorithm	Mass
Cpd 1: C12 H12 N2 O4	0.418	Find By Formula	248.08033

MS Zoomed Spectrum

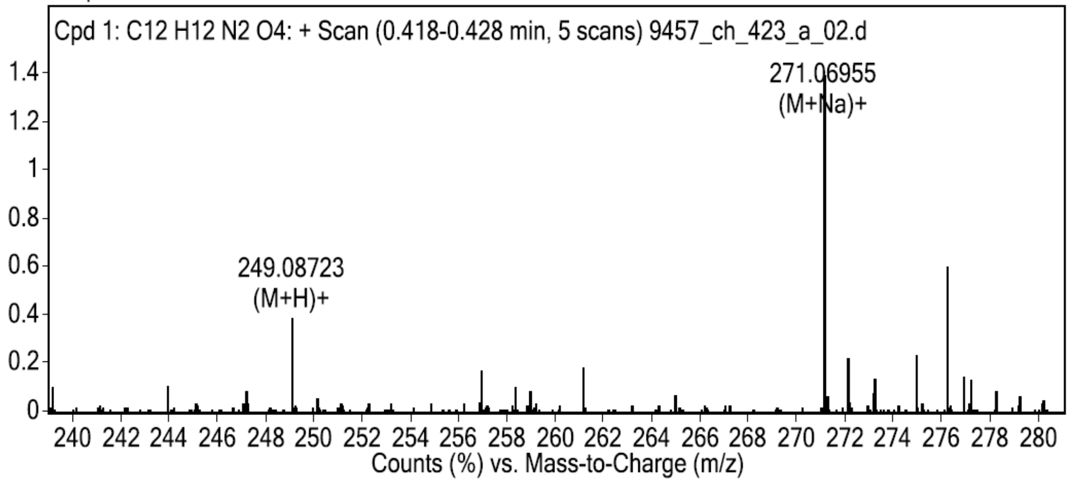

5-[(*E*)-2-(3,4,5-Trimethoxyphenyl)ethenyl]-1,3,4-oxadiazol-2(3*H*)-one (2k)

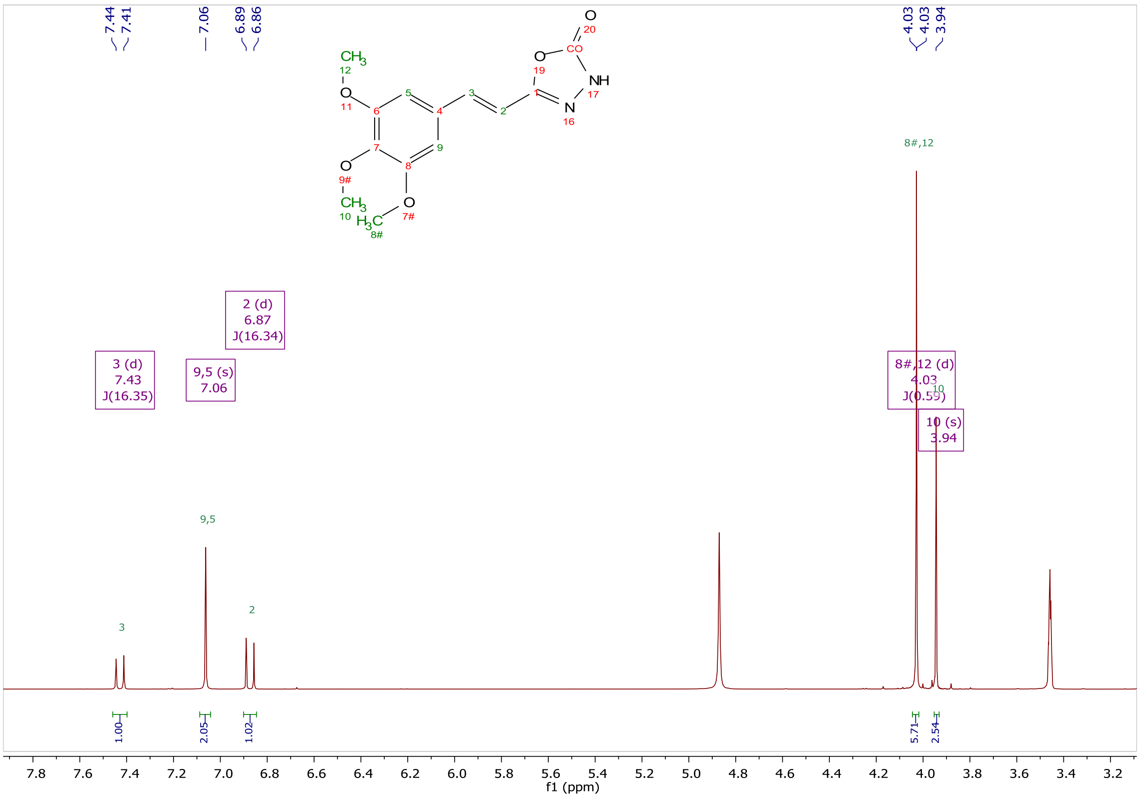

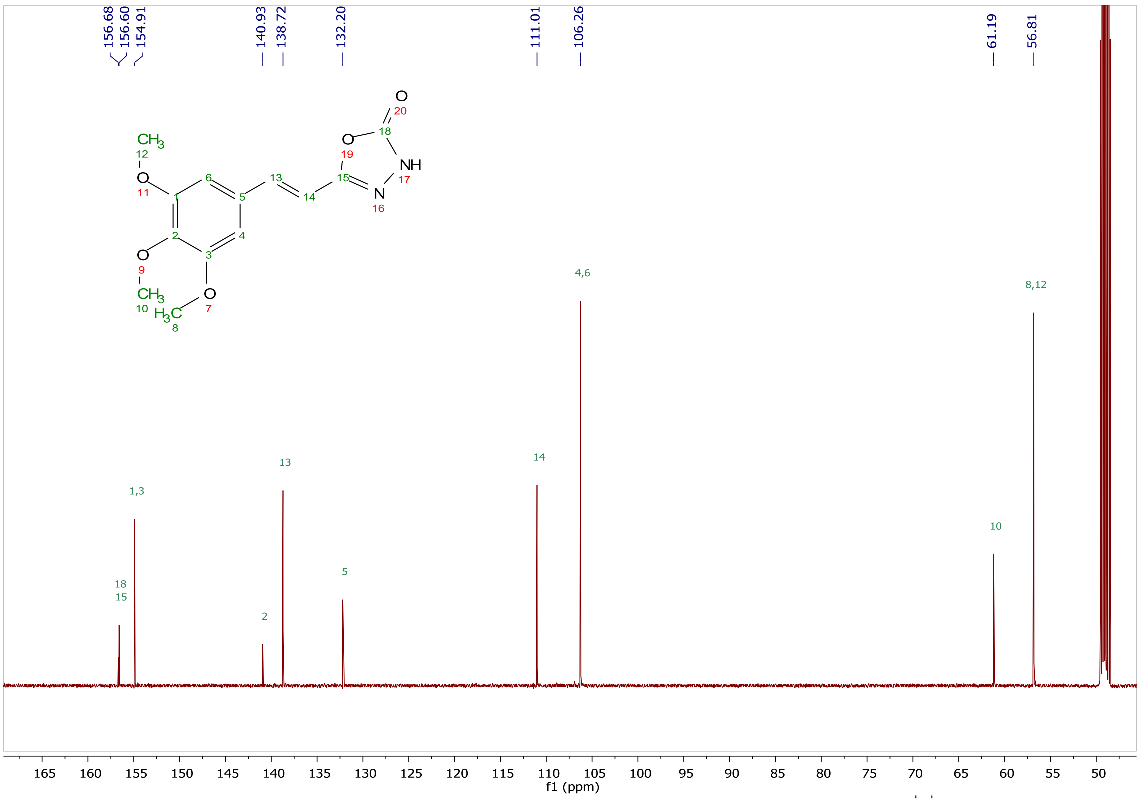
156.68
156.60
154.91
140.93
138.72
132.20
111.01
106.26
61.19
56.81
4,6
8,12
13
14
1,3
10
18
15
5
2
f1 (ppm)

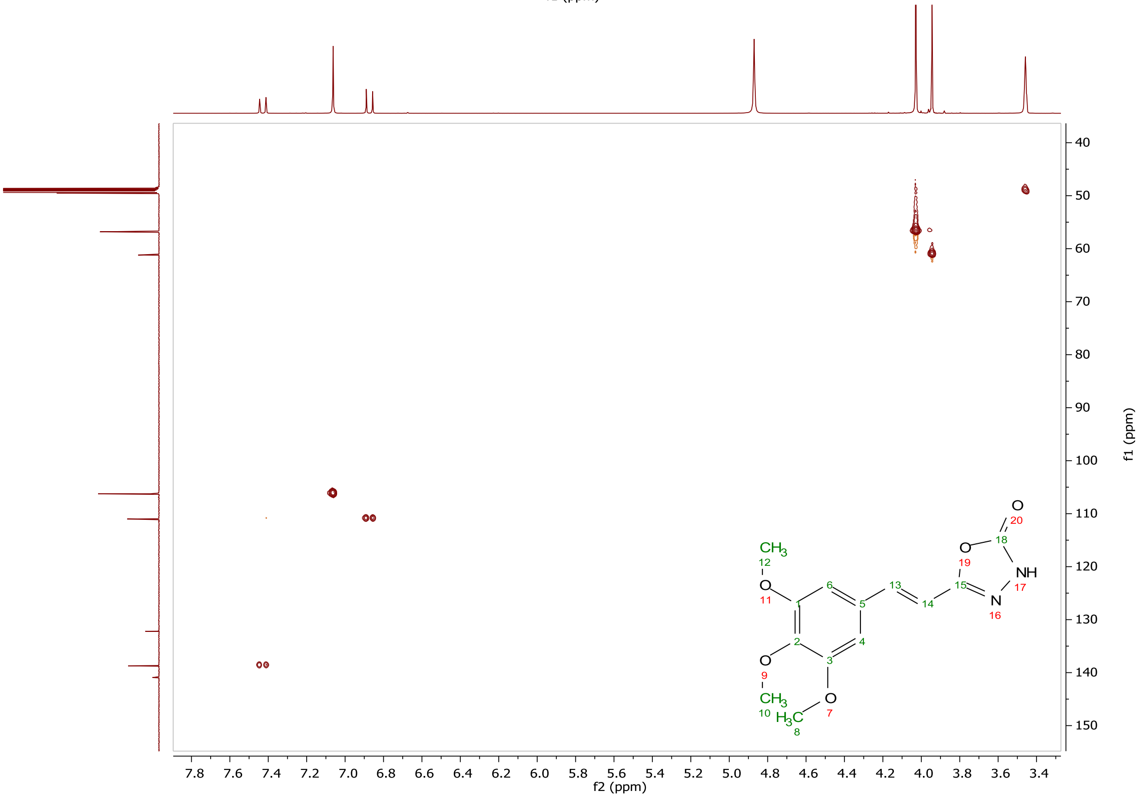
f2 (ppm)
f1 (ppm)

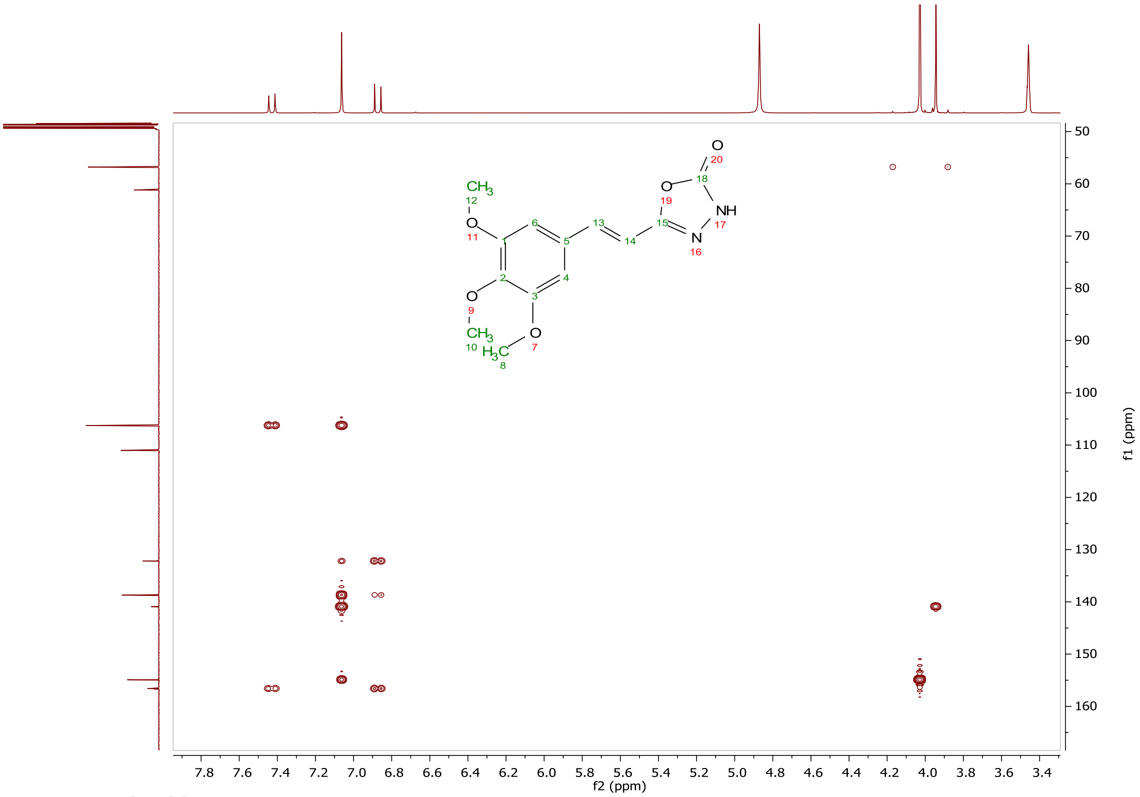

Compound Table

Compound Label	RT	Mass	Abund	Formula	Tgt Mass	Diff (ppm)
Cpd 1: C13 H14 N2 O5	0.269	278.09113	159380	C13 H14 N2 O5	278.09027	3.1

Compound Label	RT	Algorithm	Mass
Cpd 1: C13 H14 N2 O5	0.269	Find By Formula	278.09113

MS Zoomed Spectrum

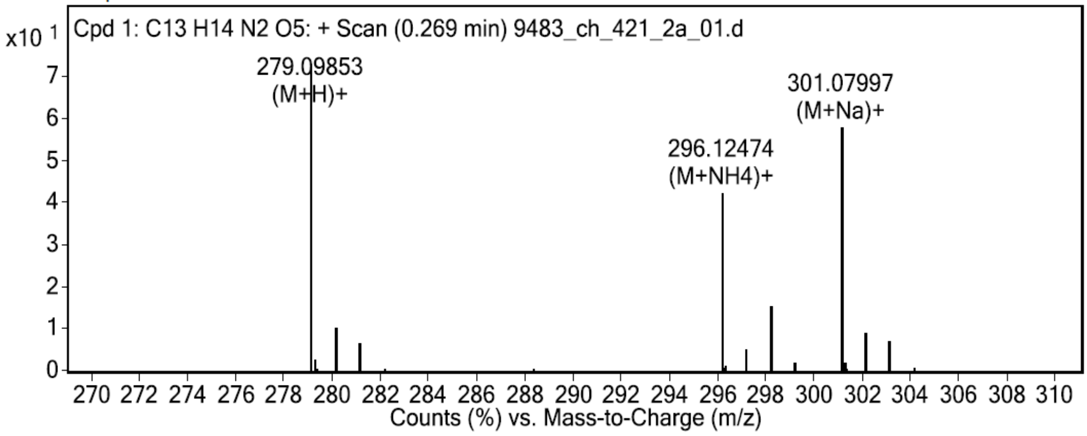

5-[(*E*)-2-(3-Hydroxyphenyl)ethenyl]-1,3,4-oxadiazol-2(3*H*)-one (3l)

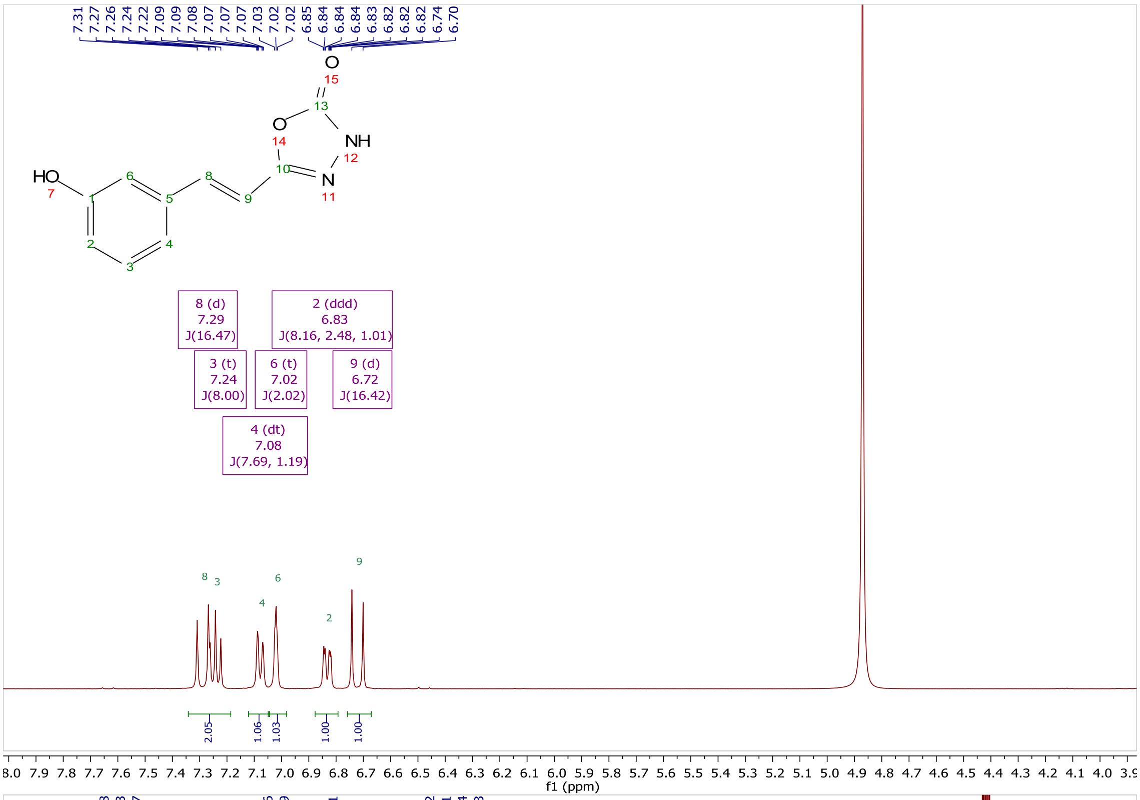

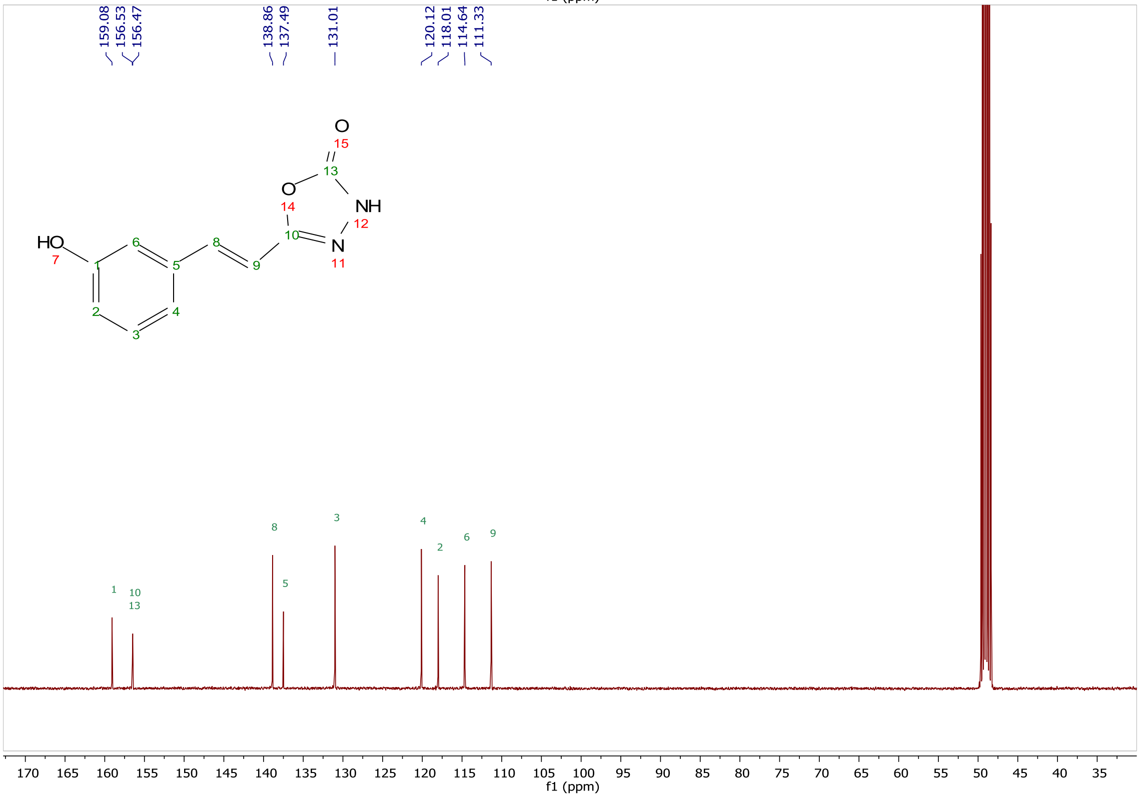

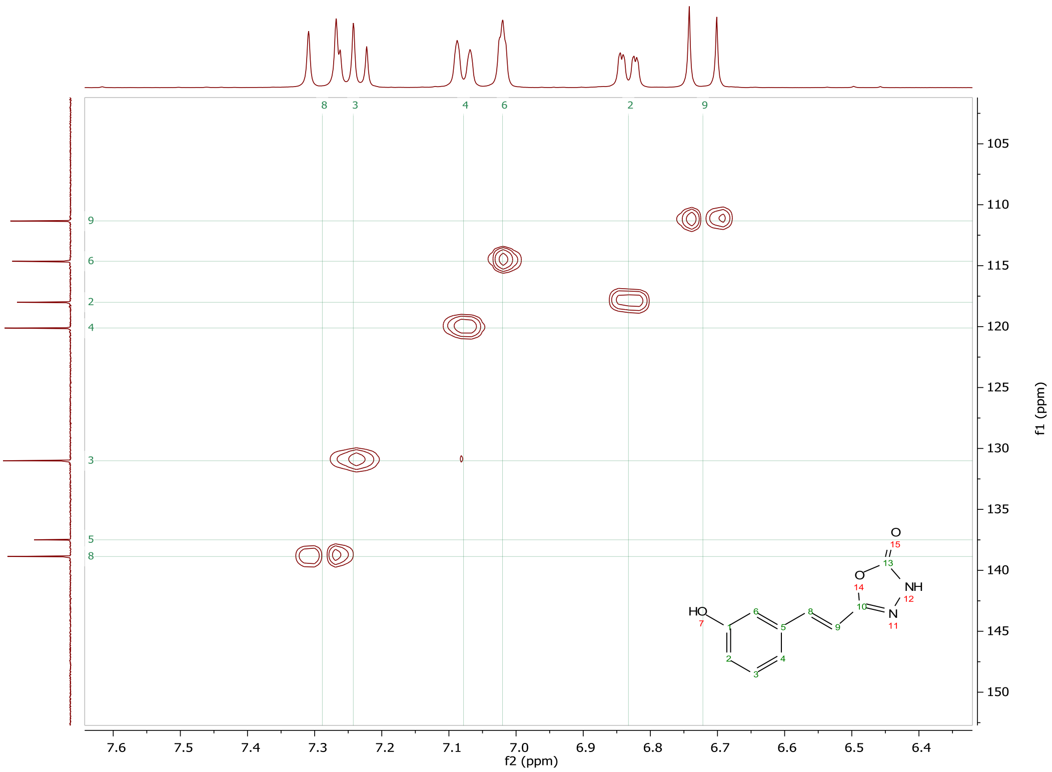

f2 (ppm)
f1 (ppm)
HO
N
NH
O

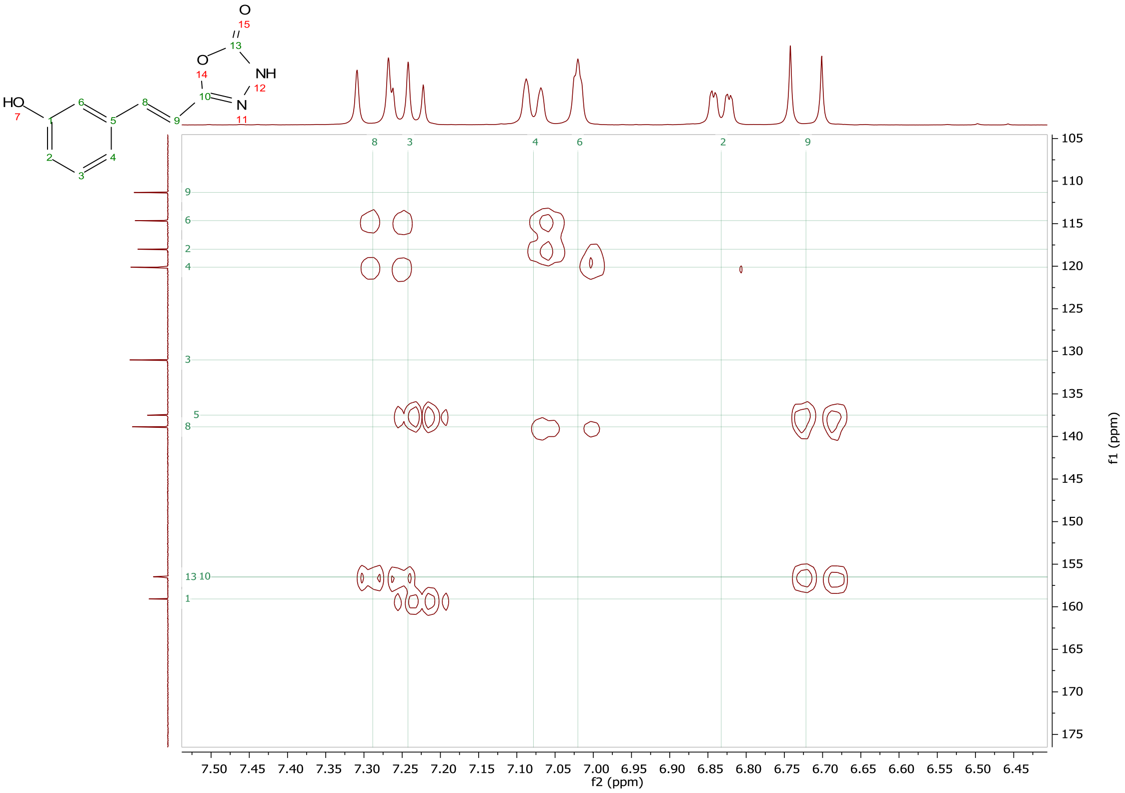

f2 (ppm)
f1 (ppm)
HO
N
NH
O

Compound Table

Compound Label	RT	Mass	Abund	Formula	Tgt Mass	Diff (ppm)
Cpd 1: C10 H8 N2 O3	0.247	204.05432	1965	C10 H8 N2 O3	204.05349	4.06

Compound Label	RT	Algorithm	Mass
Cpd 1: C10 H8 N2 O3	0.247	Find By Formula	204.05432

MS Zoomed Spectrum

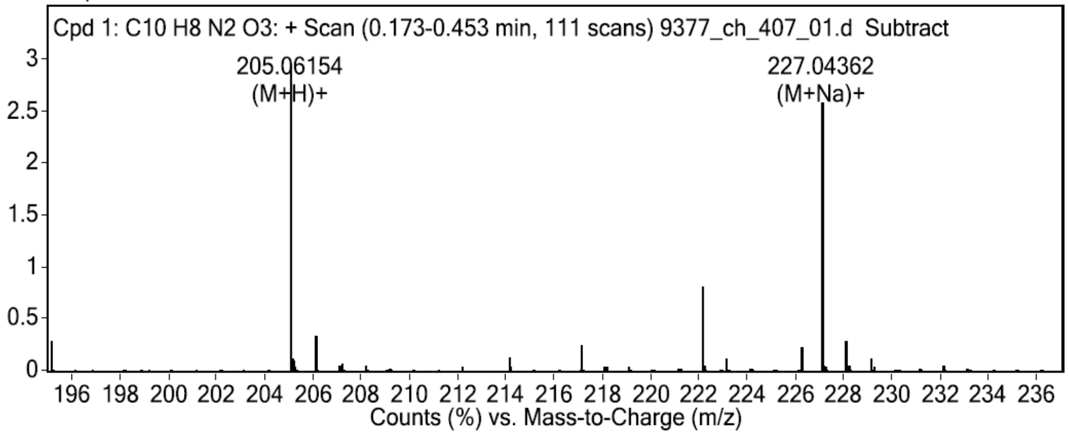

5-[(*E*)-2-(3,5-Dihydroxyphenyl)ethenyl]-1,3,4-oxadiazol-2(3*H*)-one (3p)

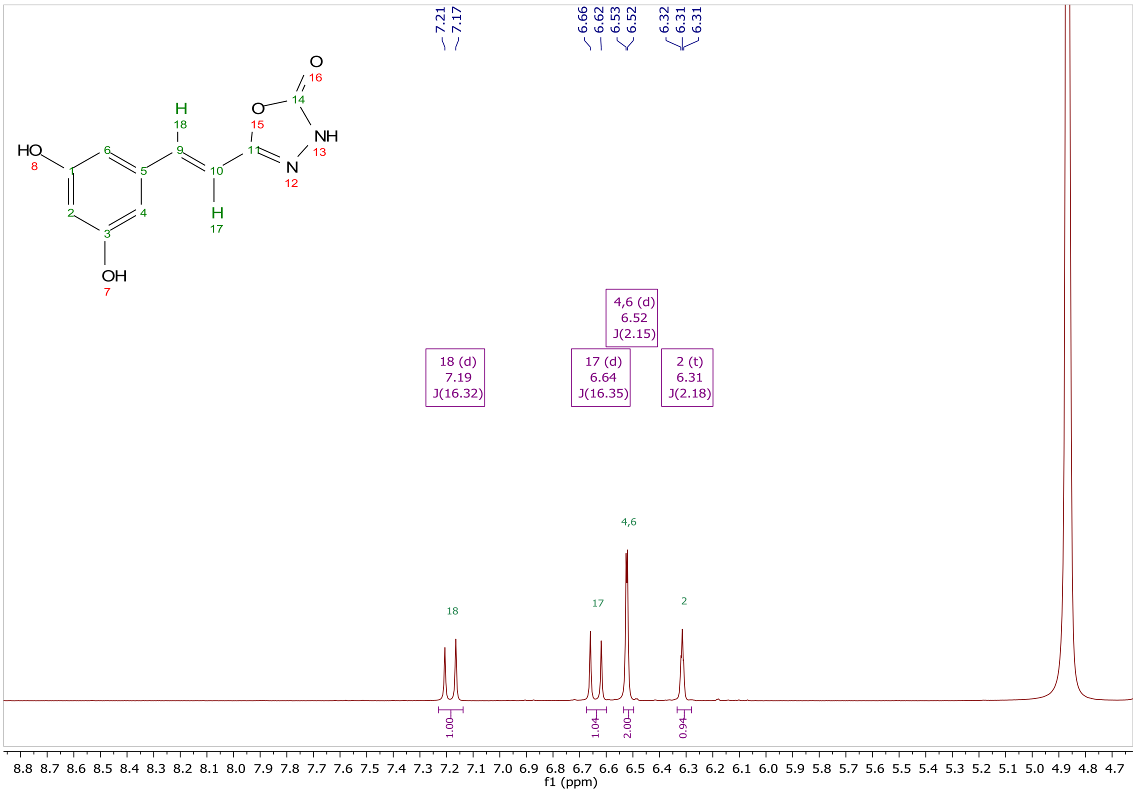

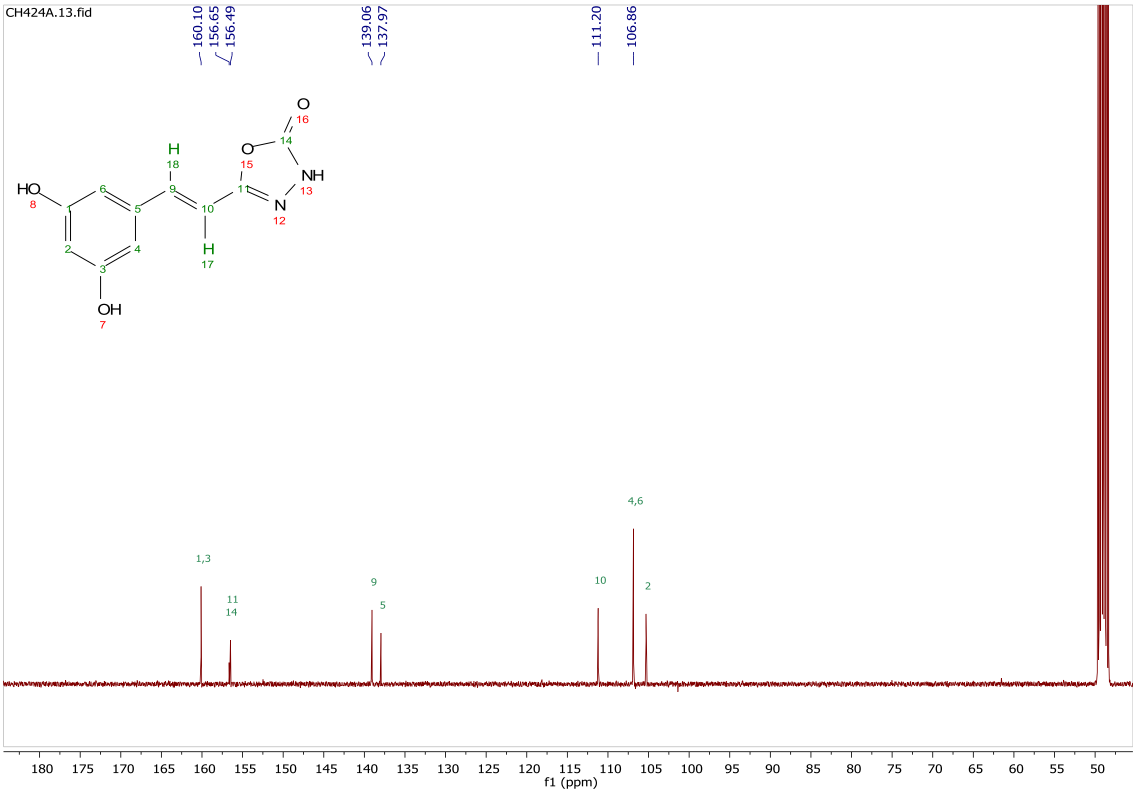
CH424A.13.fid
160.10
156.65
156.49
139.06
137.97
111.20
106.86
HO
OH
NH
N
O
1,3
11
14
9
5
10
4,6
2
f1 (ppm)

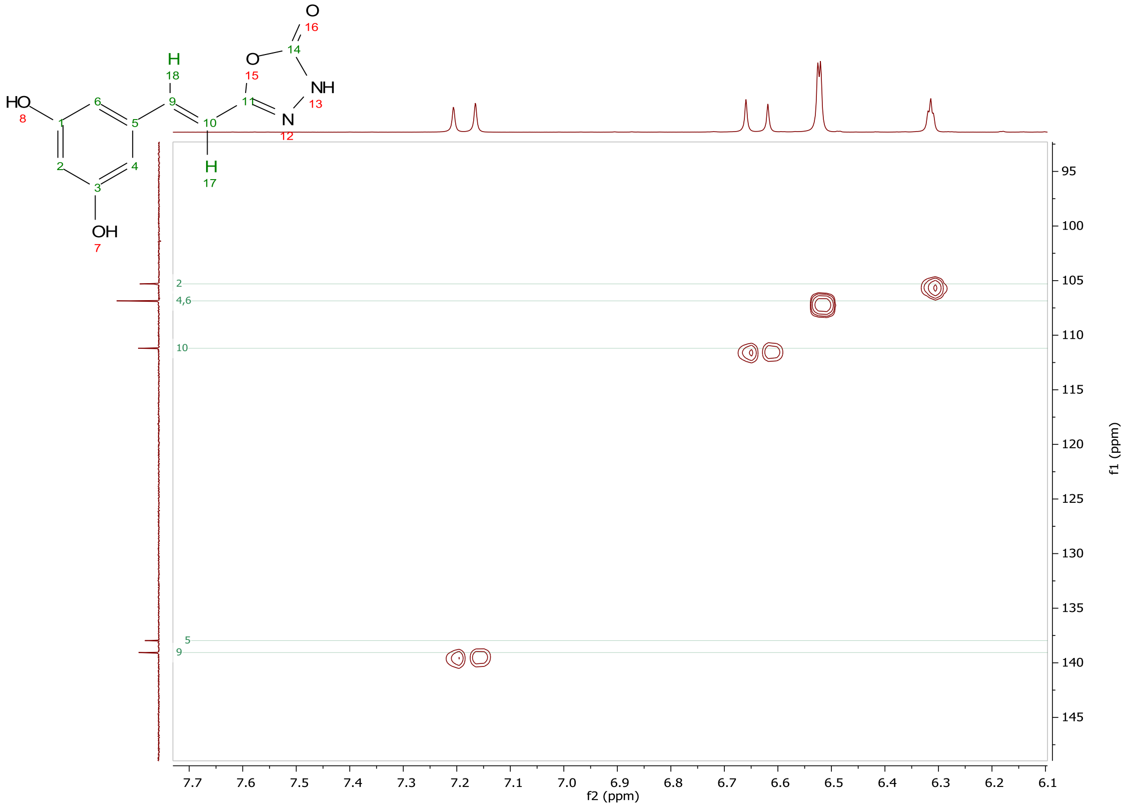
HO
OH
NH
N
O
2
4,6
10
5
9
f2 (ppm)
f1 (ppm)

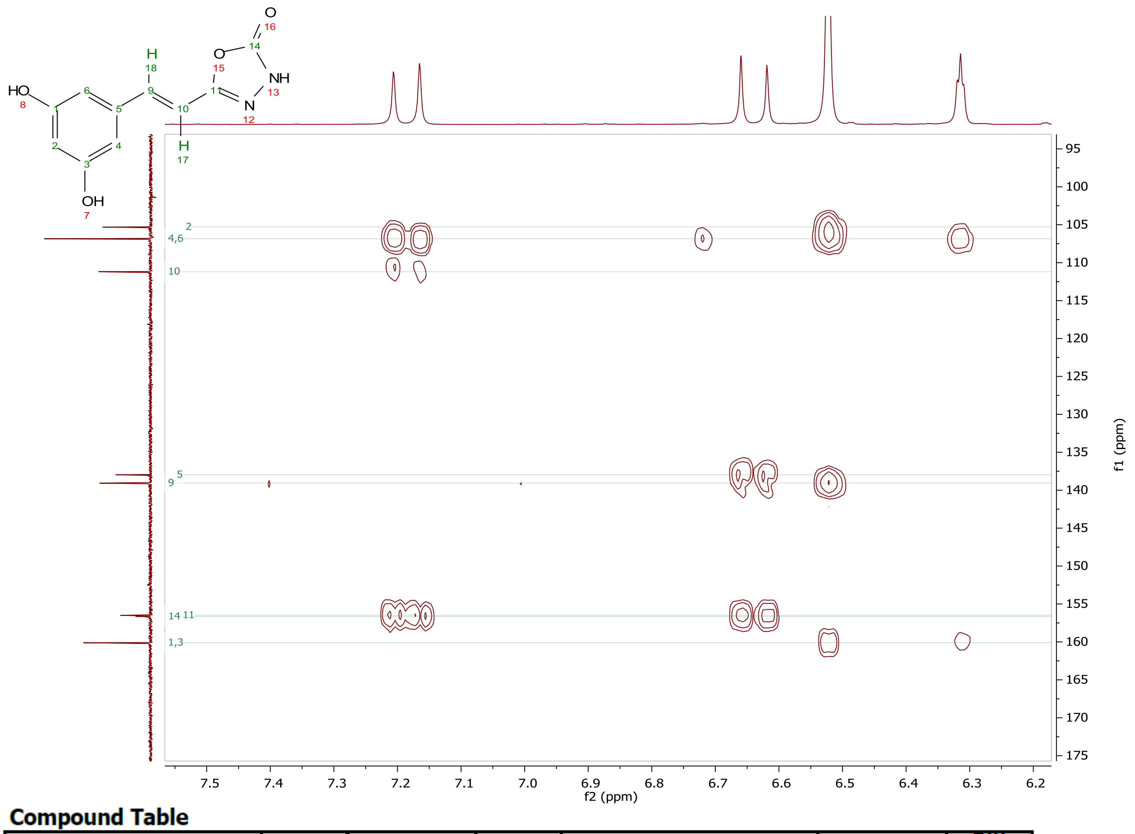

Compound Table

Compound Label	RT	Mass	Abund	Formula	Tgt Mass	Diff (ppm)
Cpd 1: C10 H8 N2 O4	0.298	220.04837	2424	C10 H8 N2 O4	220.04841	-0.18

Compound Label	RT	Algorithm	Mass
Cpd 1: C10 H8 N2 O4	0.298	Find By Formula	220.04837

MS Zoomed Spectrum

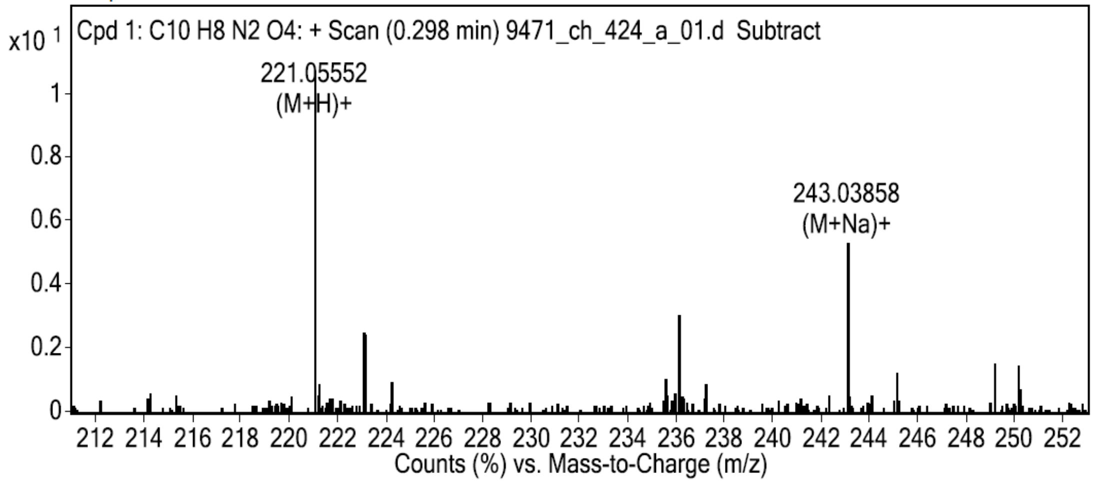

5-[(*E*)-2-(3-methoxyphenyl)ethenyl]-3-(prop-2-yn-1-yl)-1,3,4-oxadiazol-2(3*H*)-one (4d)

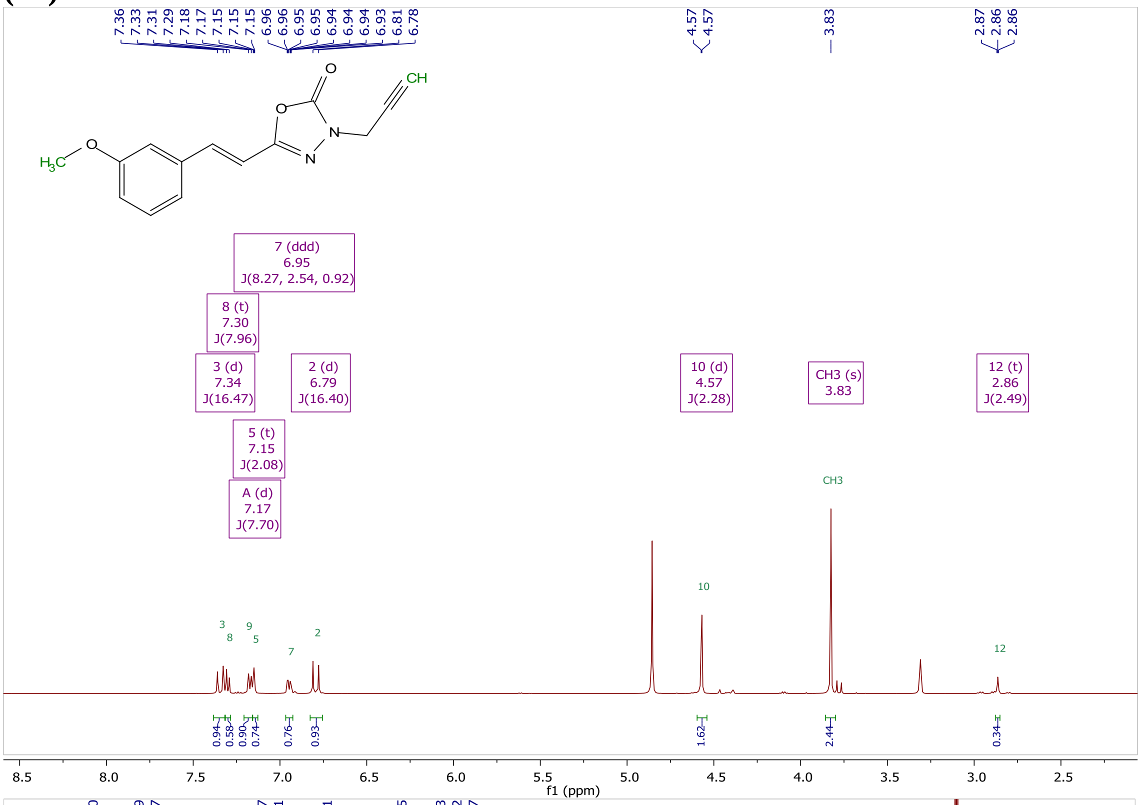

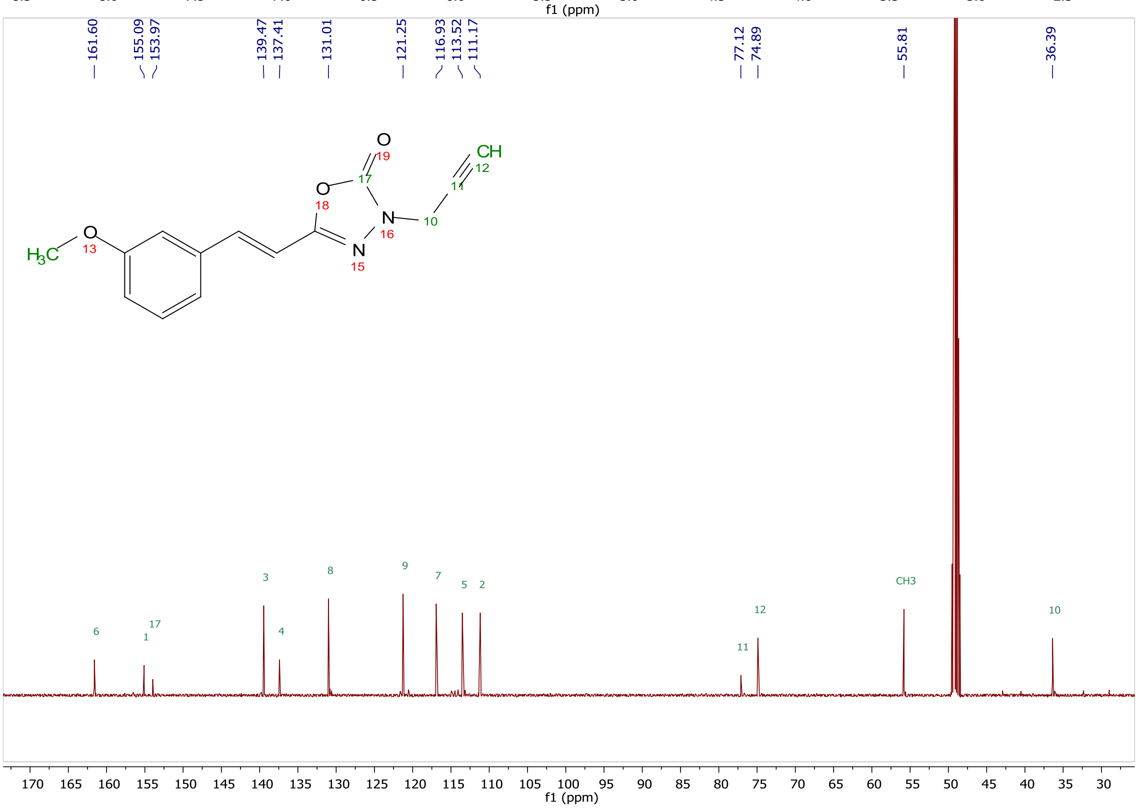

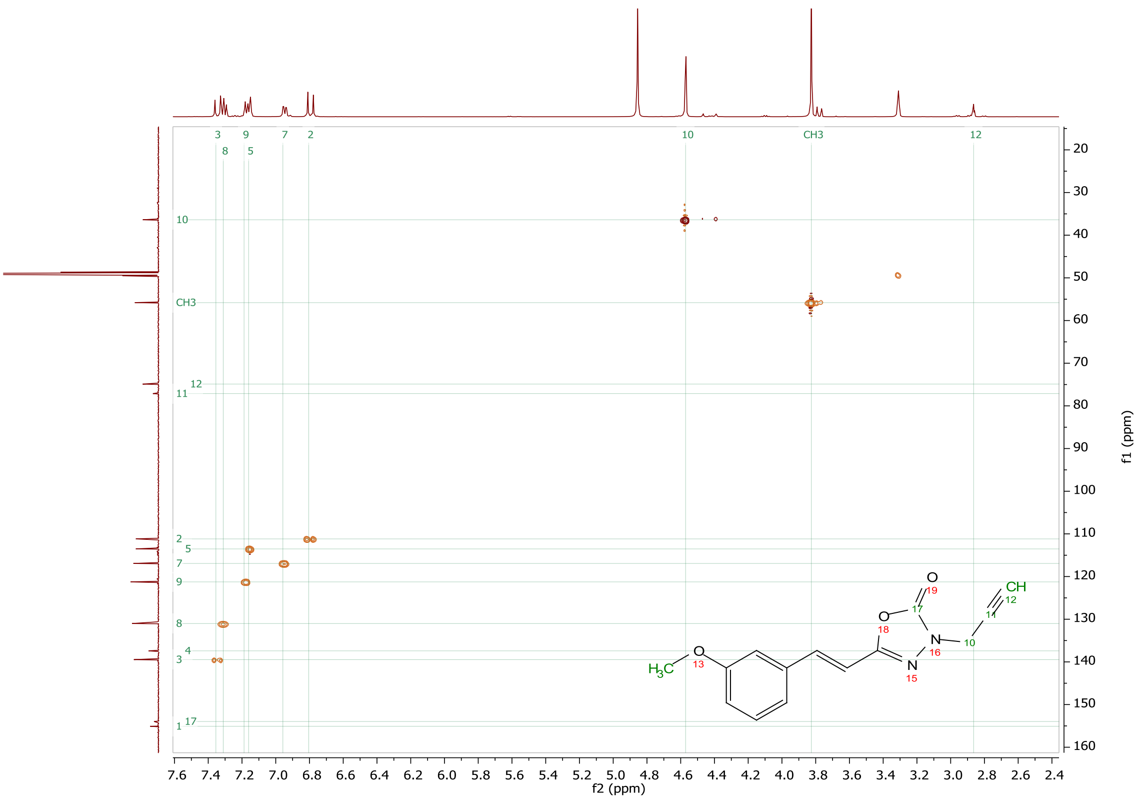
f2 (ppm)
f1 (ppm)

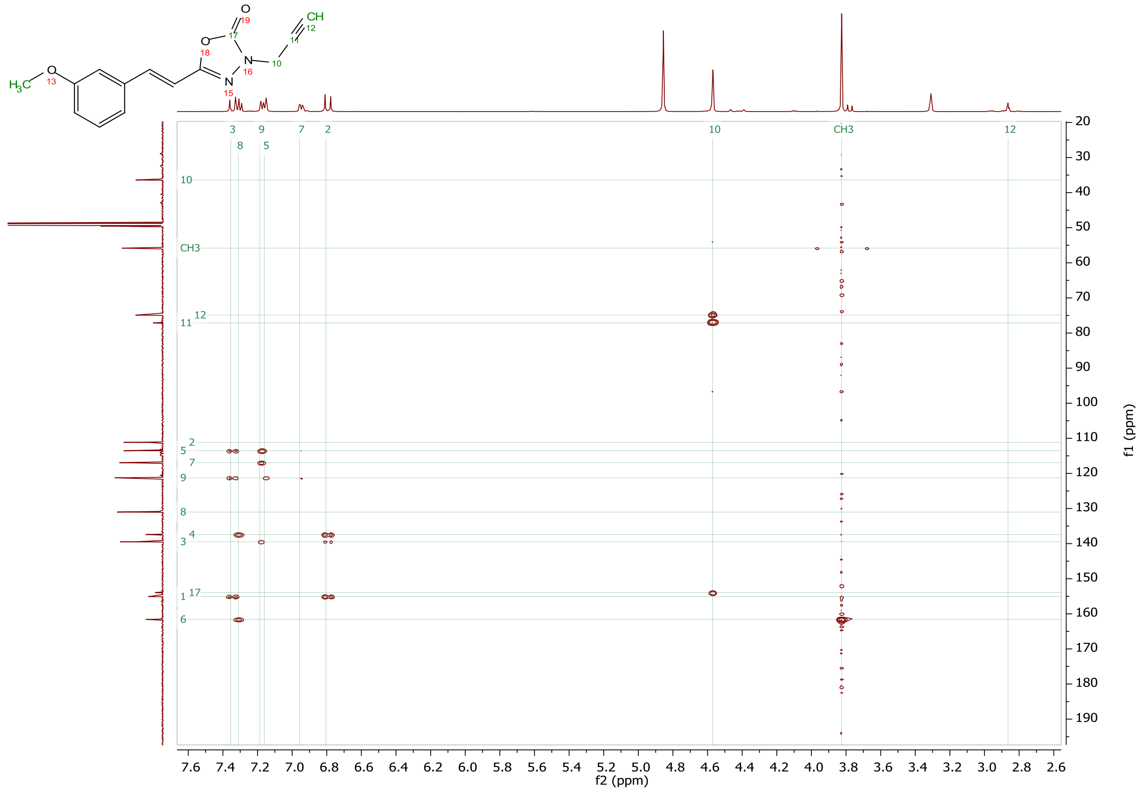
f2 (ppm)
f1 (ppm)

Compound Table

Compound Label	RT	Mass	Abund	Formula	Tgt Mass	Diff (ppm)
Cpd 1: C14 H12 N2 O3	0.251	256.08539	9772	C14 H12 N2 O3	256.08479	2.34

Compound Label	RT	Algorithm	Mass
Cpd 1: C14 H12 N2 O3	0.251	Find By Formula	256.08539

MS Zoomed Spectrum

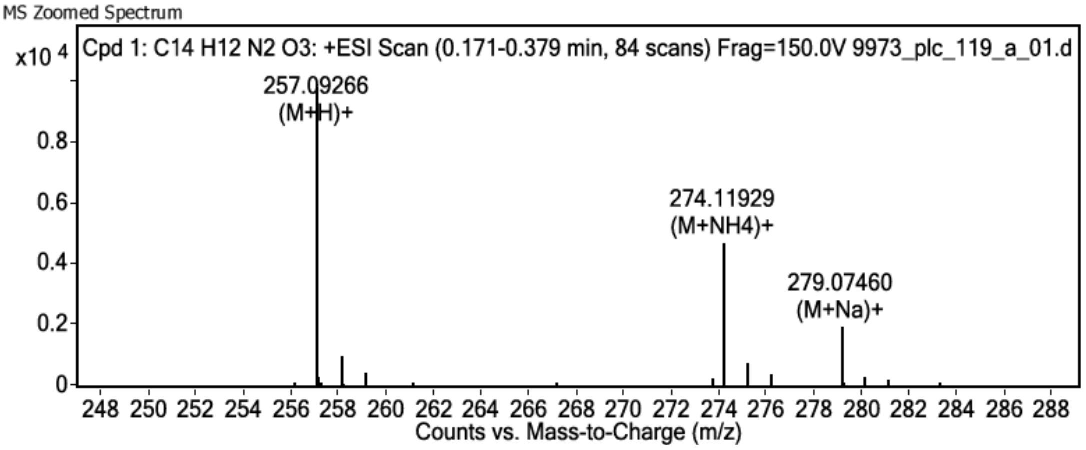

5-[(*E*)-2-(4-methoxyphenyl)ethenyl]-3-(prop-2-yn-1-yl)-1,3,4-oxadiazol-2(3*H*)-one (4e)

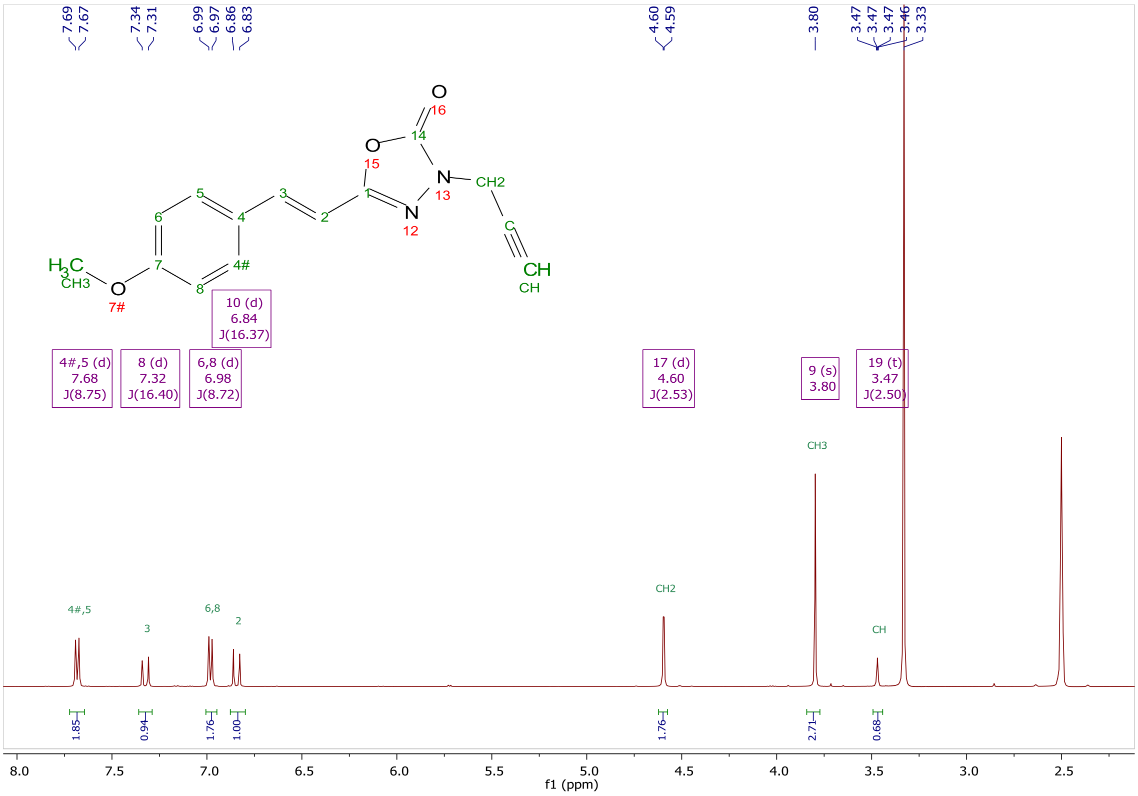

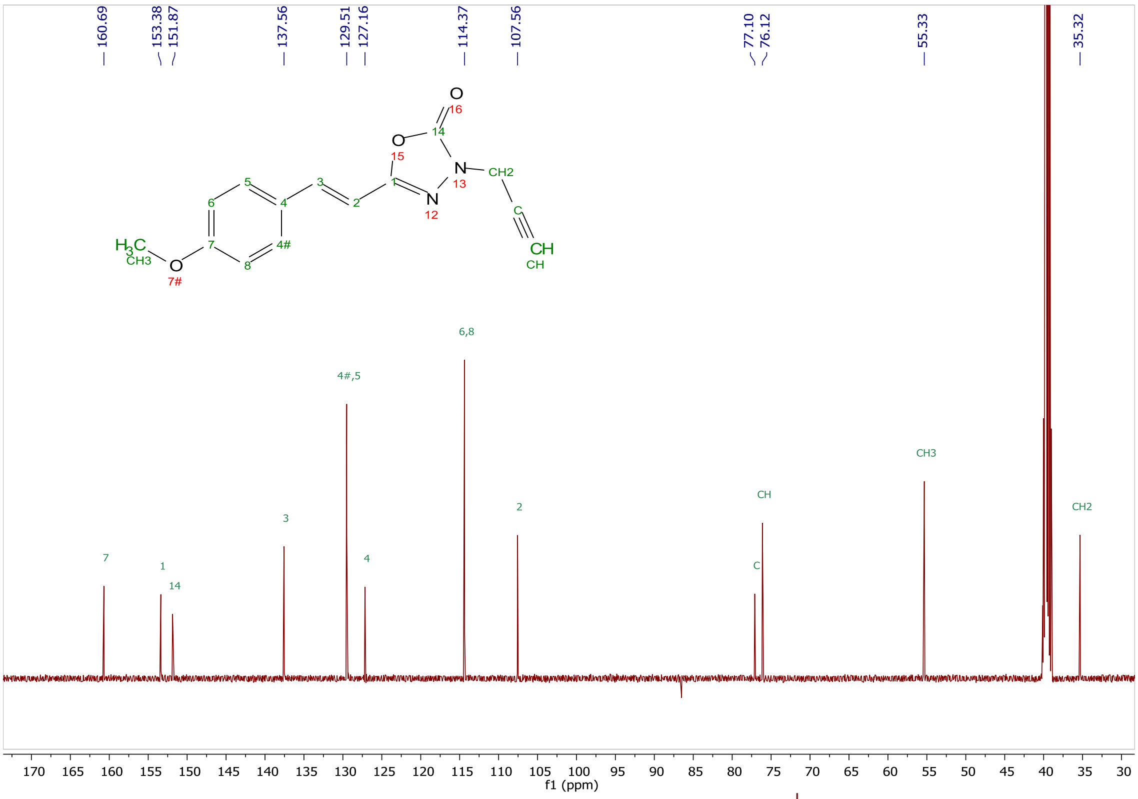

160.69
153.38
151.87
137.56
129.51
127.16
114.37
107.56
77.10
76.12
55.33
35.32
f1 (ppm)

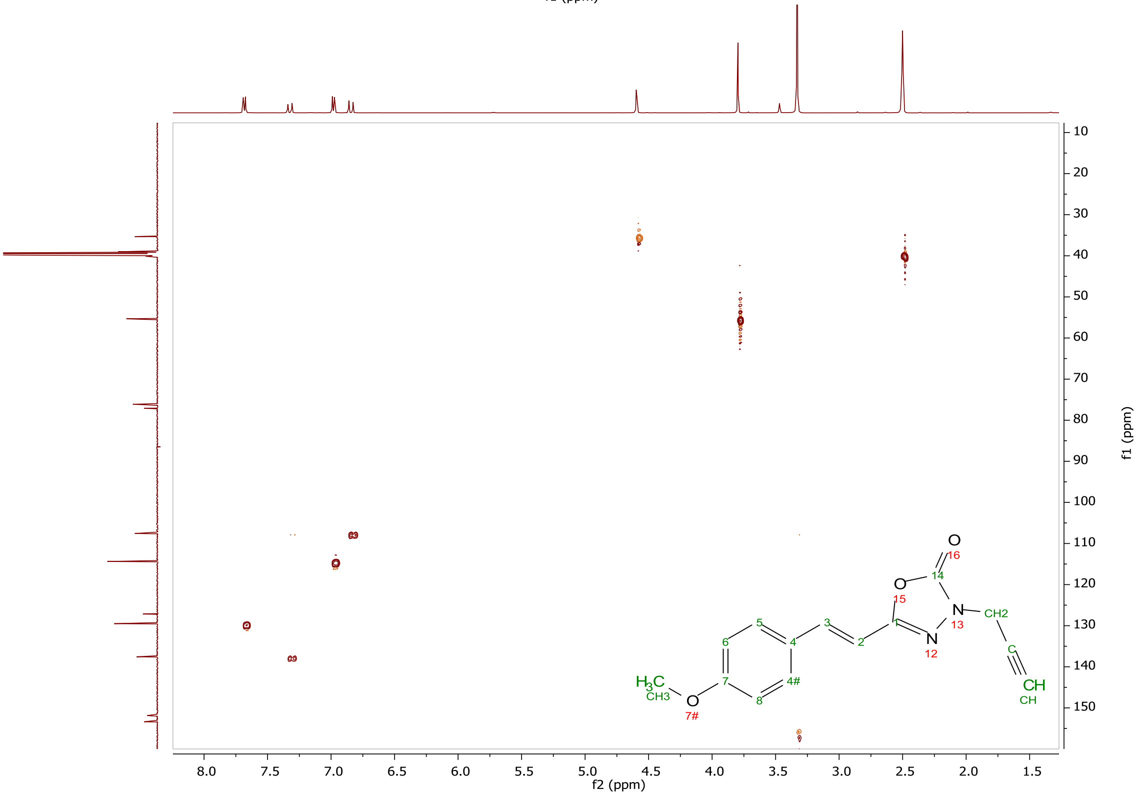

f2 (ppm)
f1 (ppm)

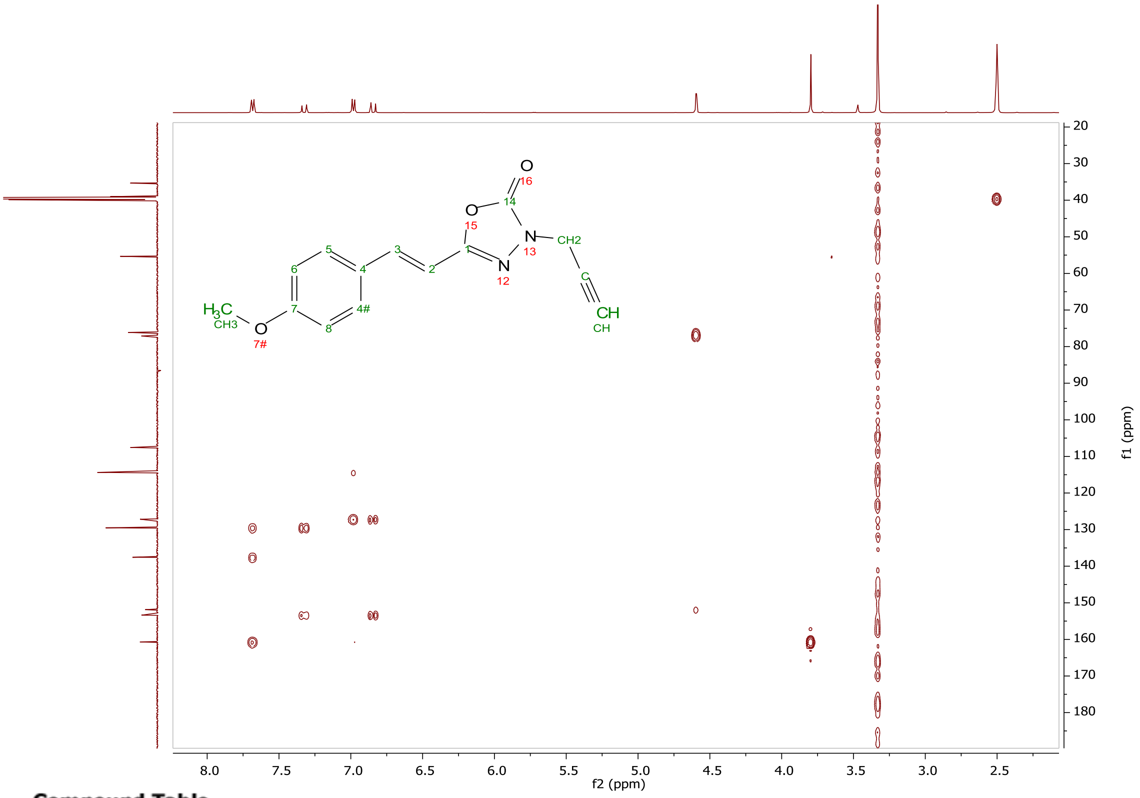

Compound Table

Compound Label	RT	Mass	Abund	Formula	Tgt Mass	Diff (ppm)
Cpd 1: C14 H12 N2 O3	0.481	256.08363	16838	C14 H12 N2 O3	256.08479	-4.52

Compound Label	RT	Algorithm	Mass
Cpd 1: C14 H12 N2 O3	0.481	Find By Formula	256.08363

MS Zoomed Spectrum

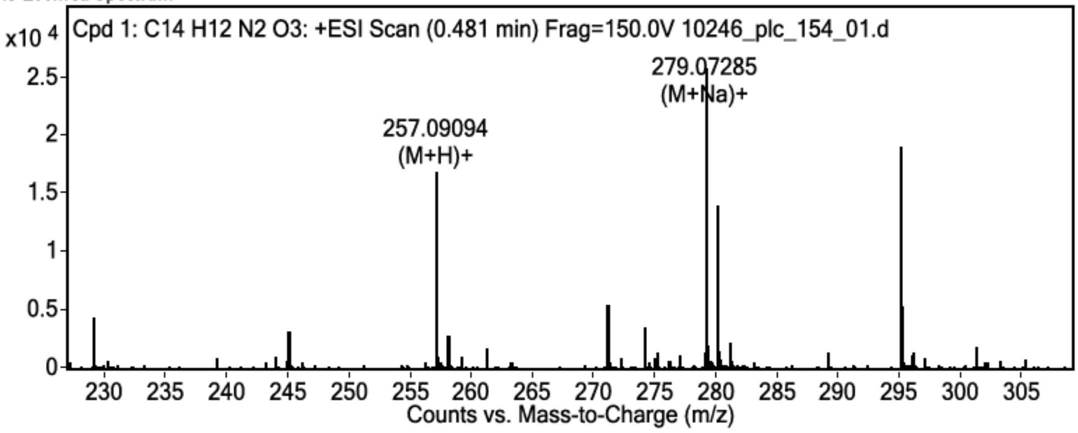

5-[(*E*)-2-(3-hydroxyphenyl)ethenyl]-3-(prop-2-yn-1-yl)-1,3,4-oxadiazol-2(3*H*)-one (5l)

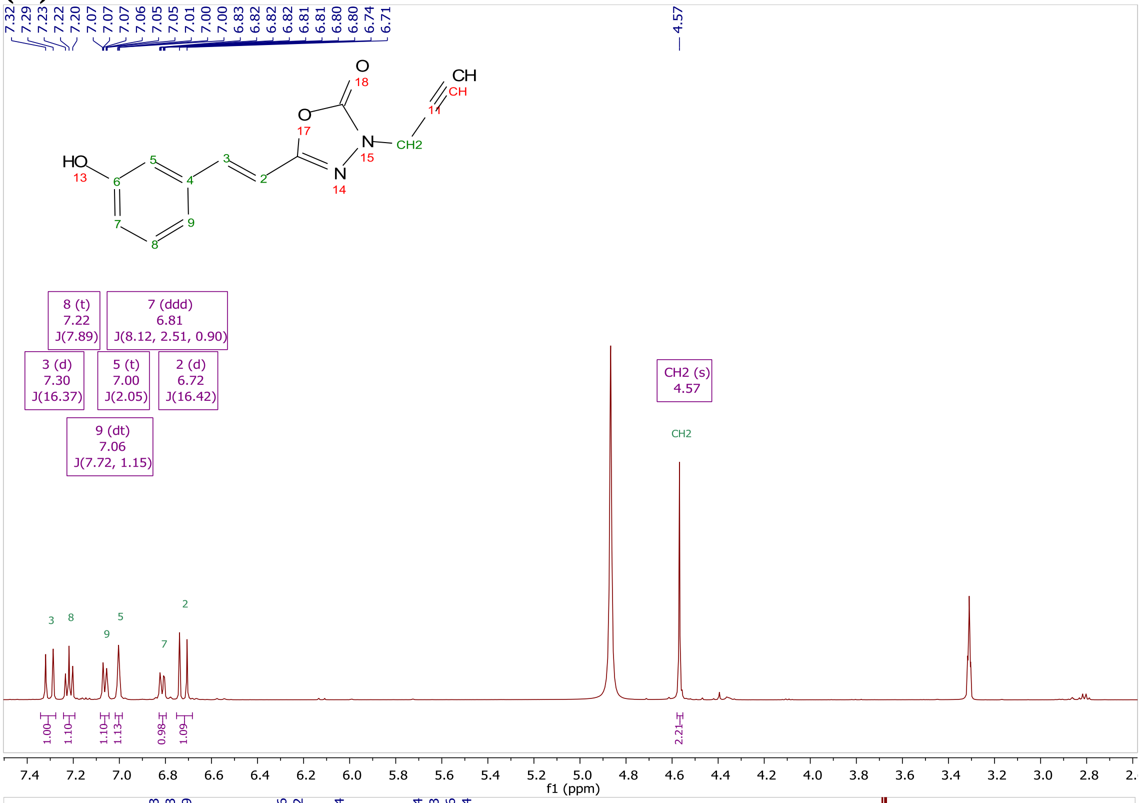

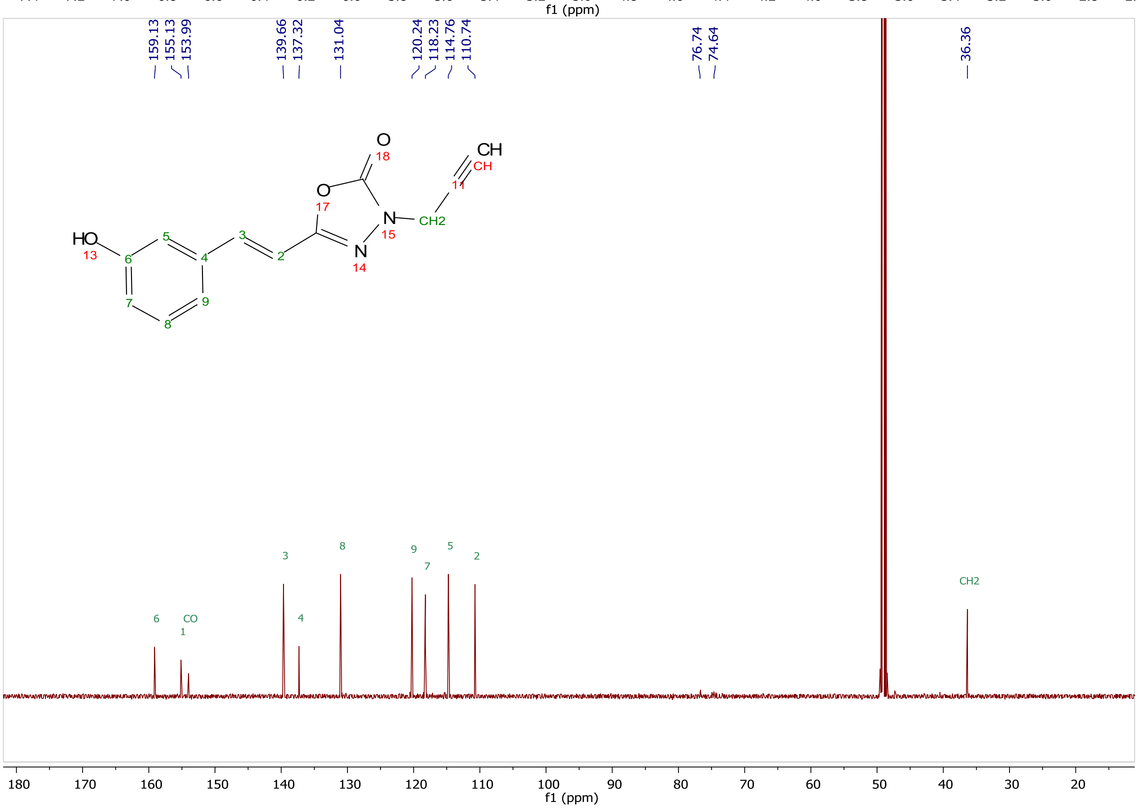

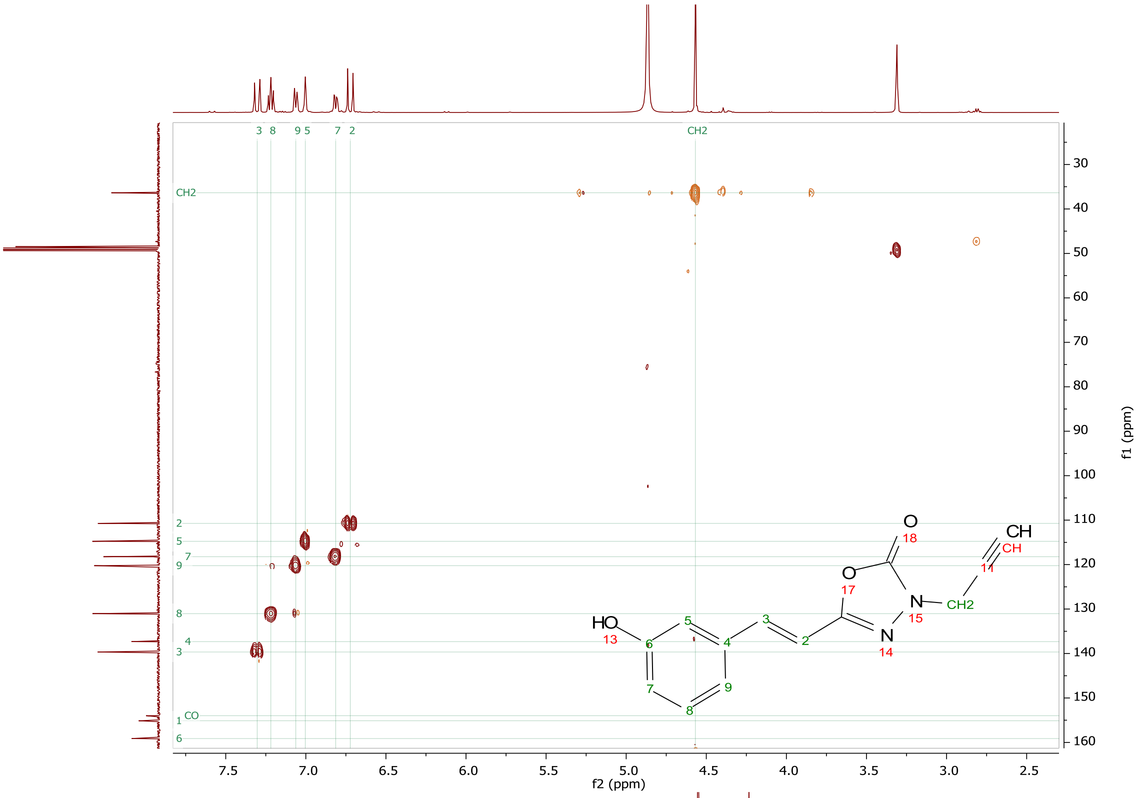

3 8 9 5 7 2
CH2
CO
f1 (ppm)
f2 (ppm)
HO
O
N
CH

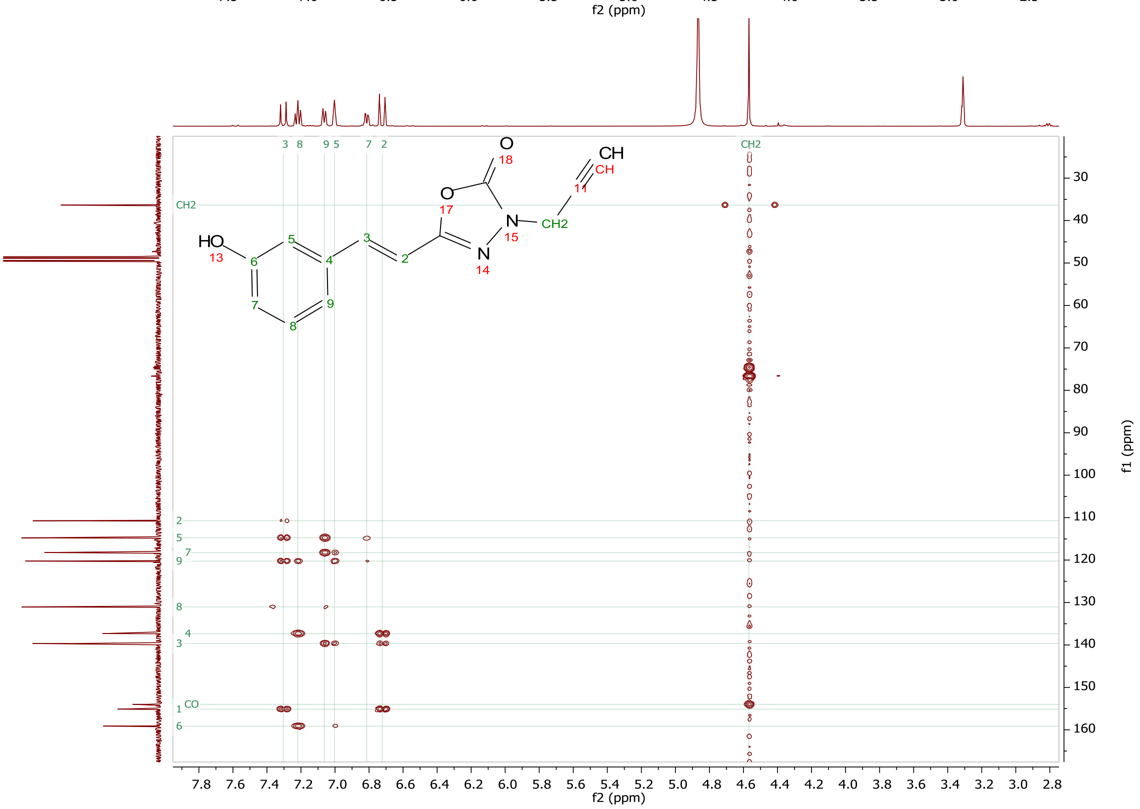

3 8 9 5 7 2
CH2
CO
f1 (ppm)
f2 (ppm)
HO
O
N
CH

Compound Table

Compound Label	RT	Mass	Abund	Formula	Tgt Mass	Diff (ppm)
Cpd 1: C13 H10 N2 O3	0.253	242.06867	39589	C13 H10 N2 O3	242.06914	-1.94

Compound Label	RT	Algorithm	Mass
Cpd 1: C13 H10 N2 O3	0.253	Find By Formula	242.06867

MS Zoomed Spectrum

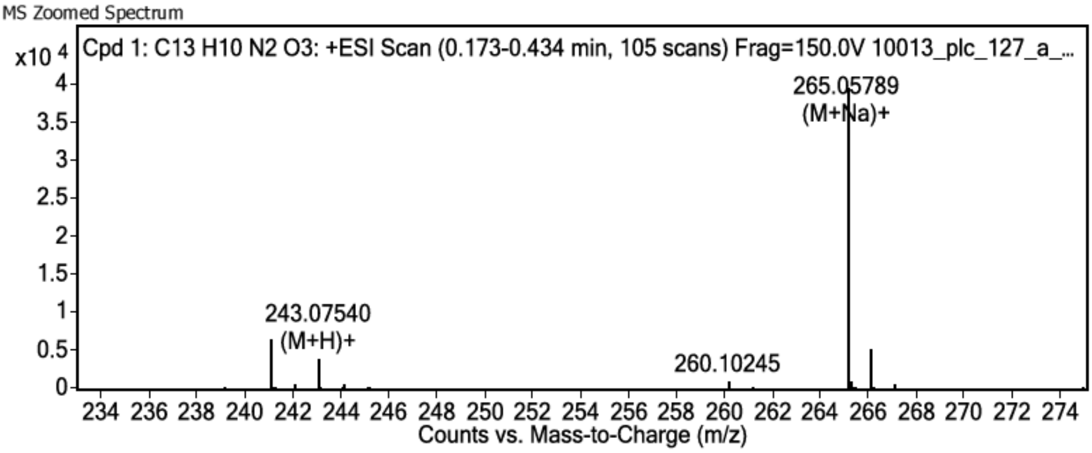

(2*E*)-3-(3-Hydroxyphenyl)-*N*-(prop-2-yn-1-yl)prop-2-enamide (6l)

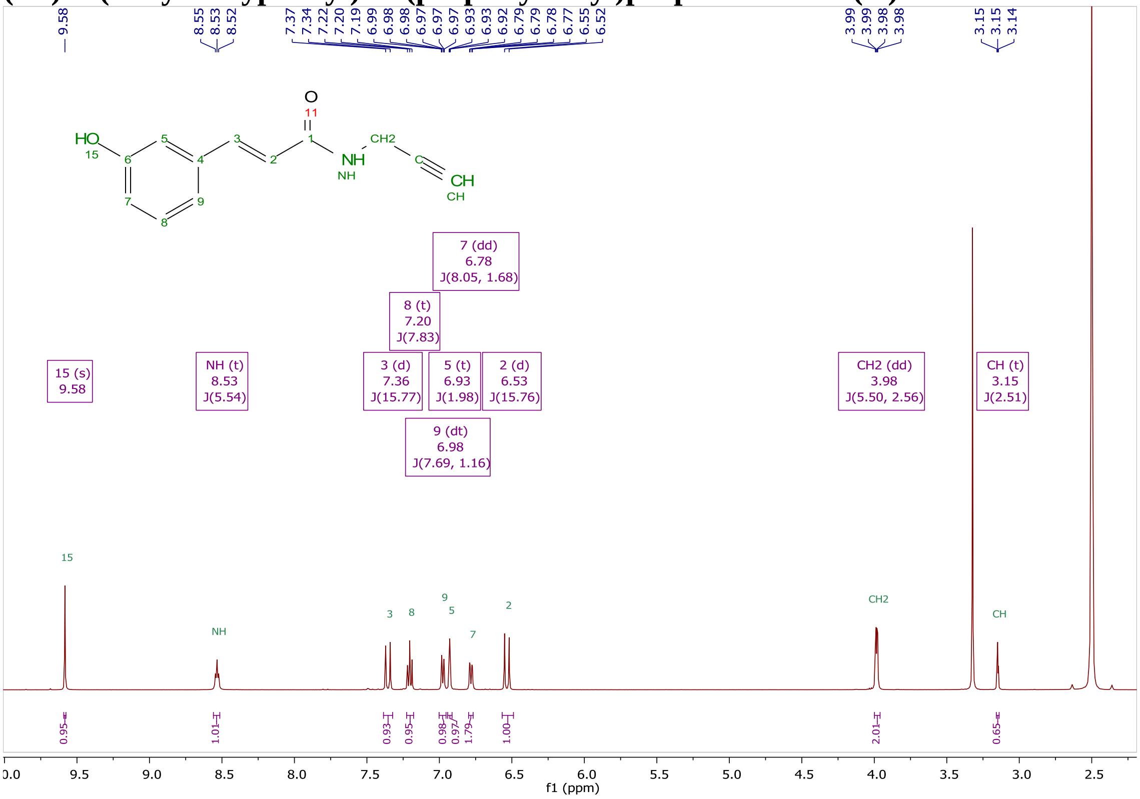

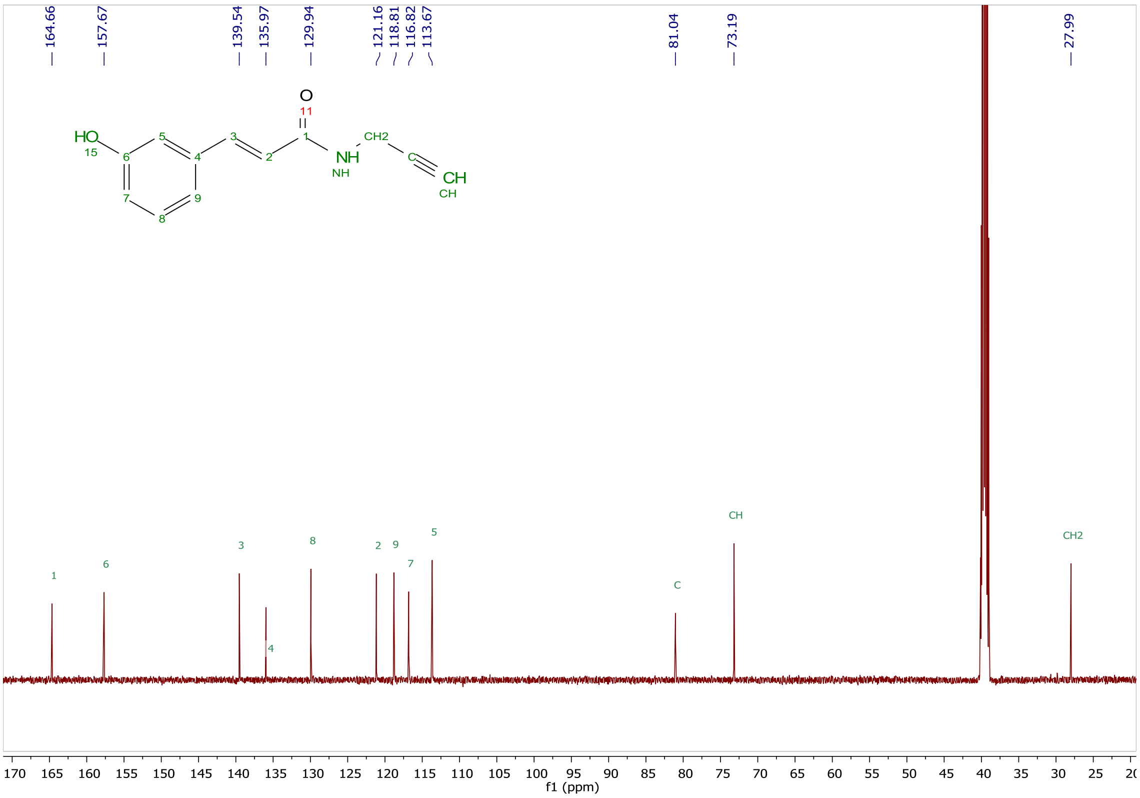
164.66
157.67
139.54
135.97
129.94
121.16
118.81
116.82
113.67
81.04
73.19
27.99
f1 (ppm)

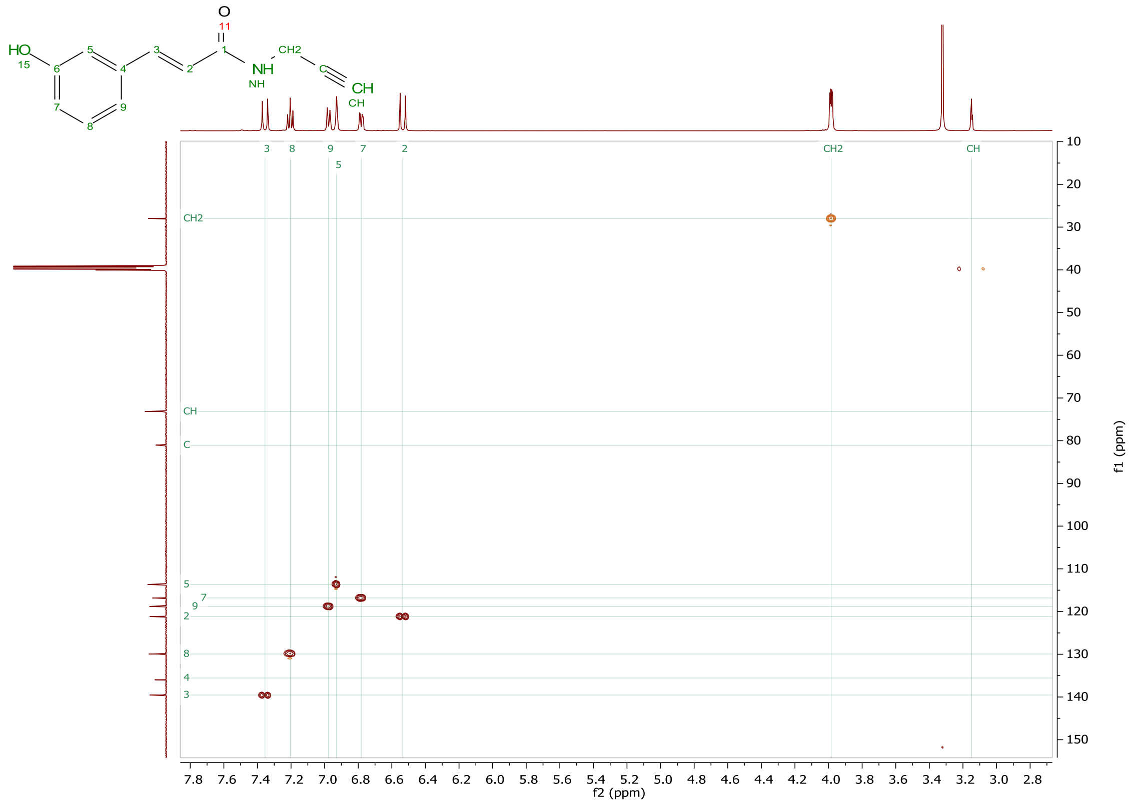
f2 (ppm)
f1 (ppm)

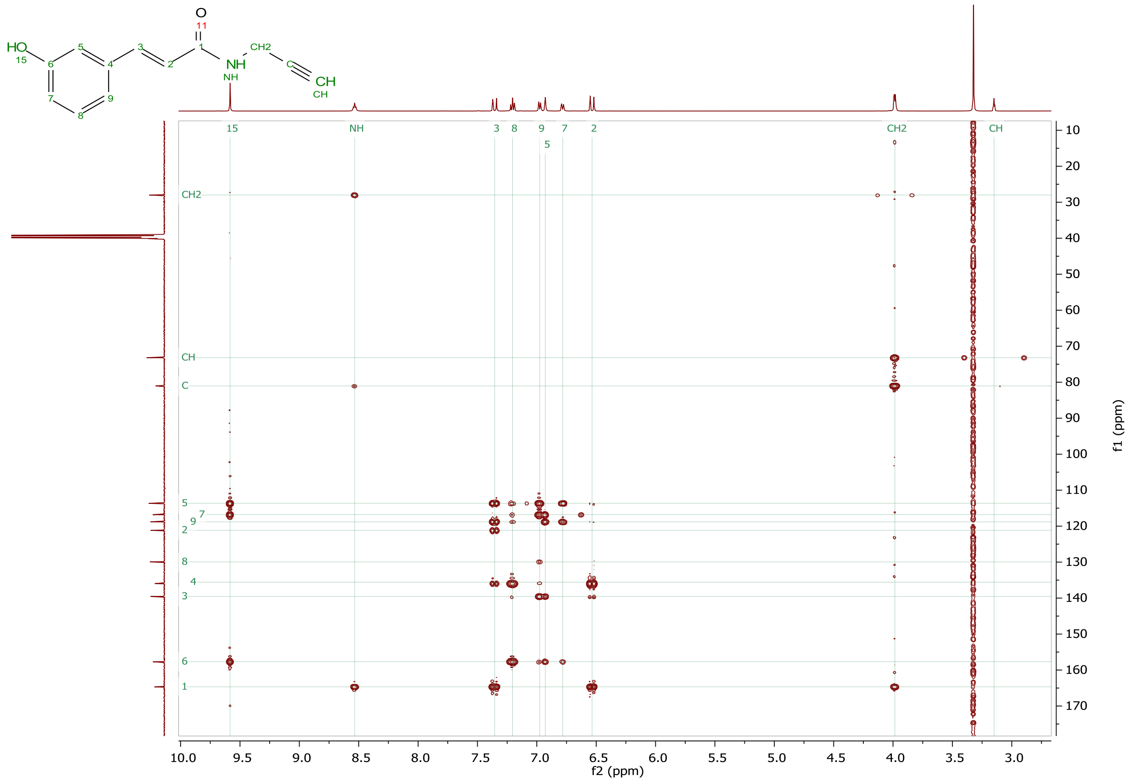

Compound Table

Compound Label	RT	Mass	Abund	Formula	Tgt Mass	Diff (ppm)
Cpd 1: C12 H11 N O2	0.478	201.07982	22055	C12 H11 N O2	201.07898	4.17

Compound Label	RT	Algorithm	Mass
Cpd 1: C12 H11 N O2	0.478	Find By Formula	201.07982

MS Zoomed Spectrum

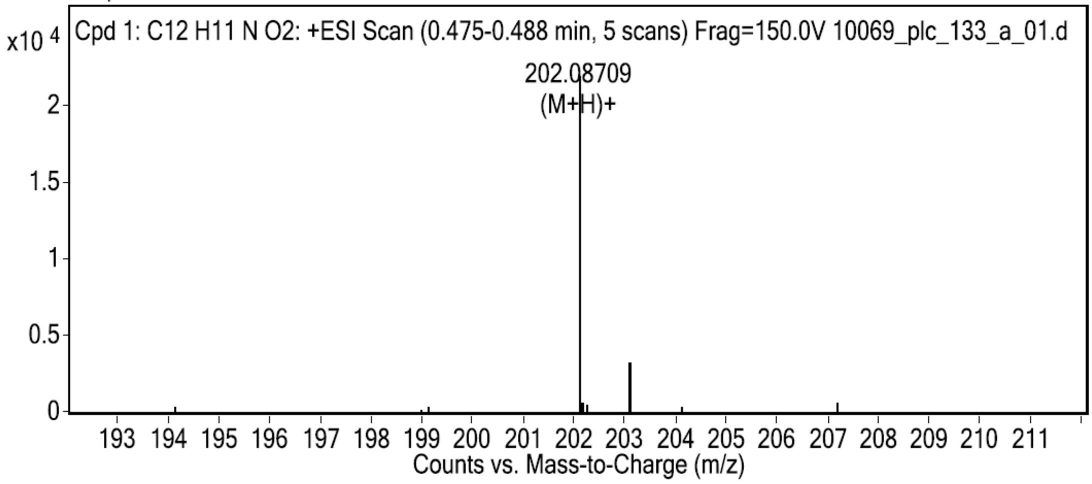

2-methoxy-4-{(1*E*)-3-[(prop-2-yn-1-yl)amino]prop-1-en-1-yl}phenol (8n)

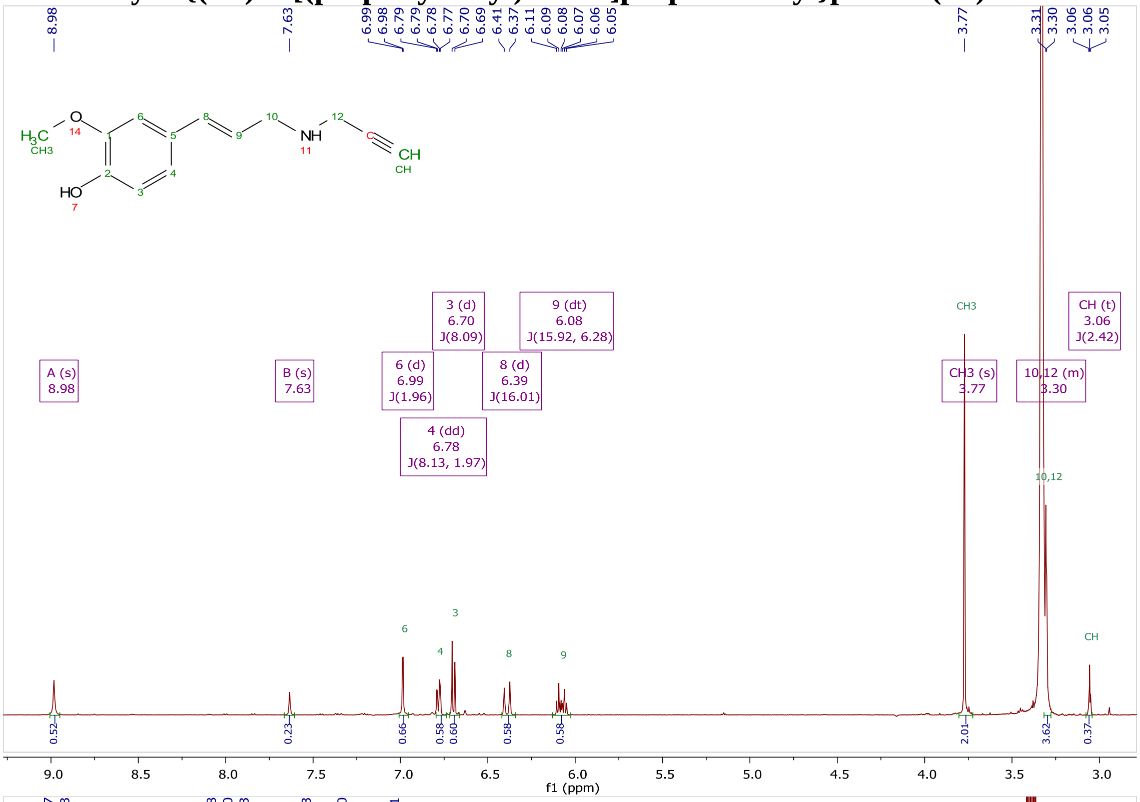

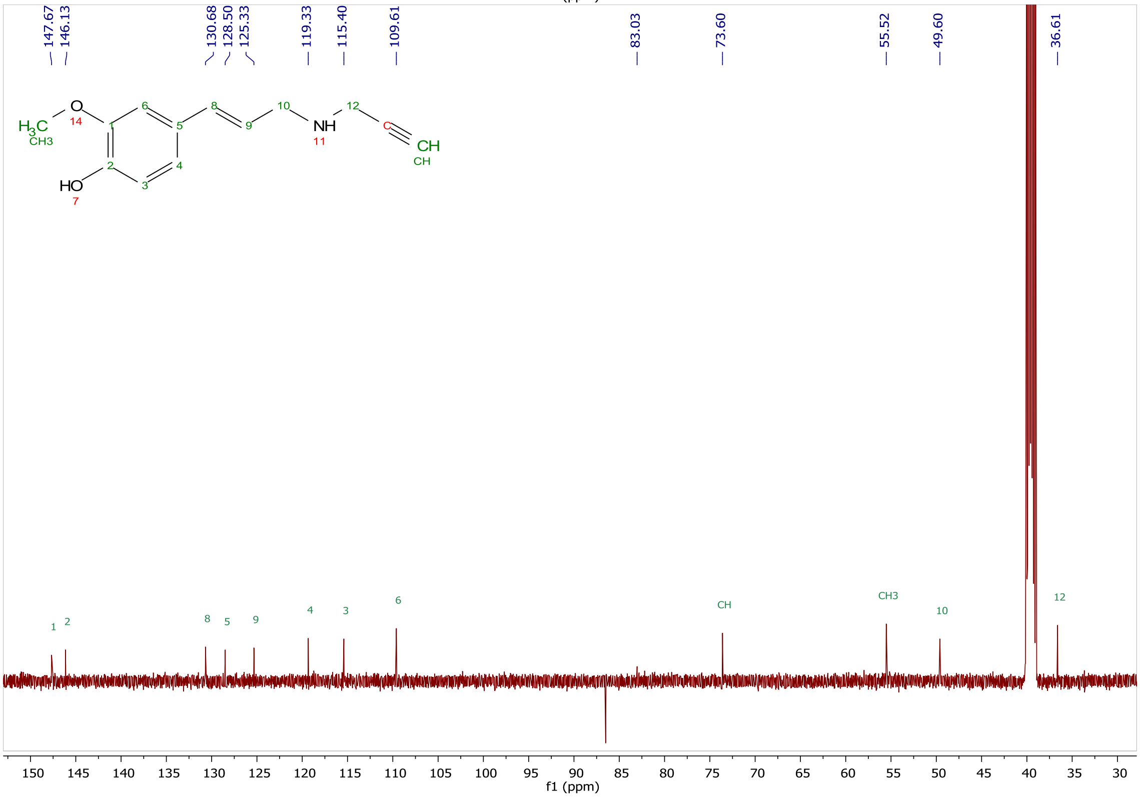

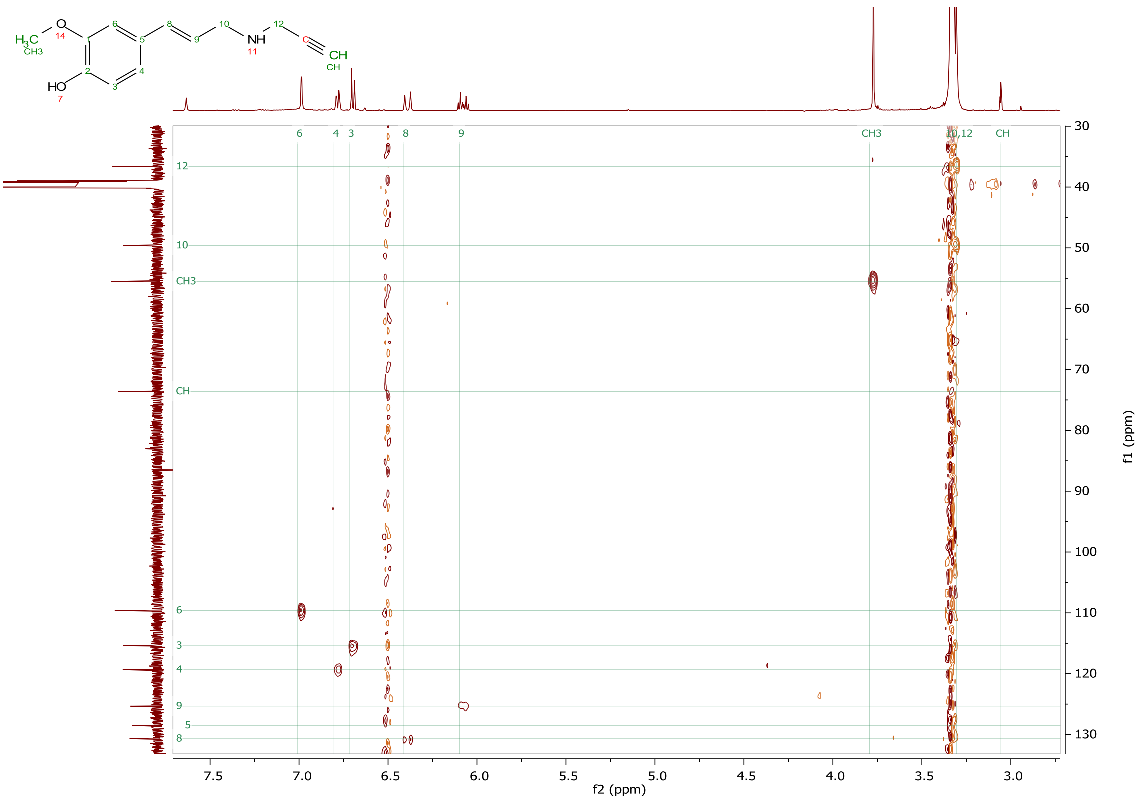

H3C
O
14
CH3
HO
7
NH
11
CH
f2 (ppm)
f1 (ppm)

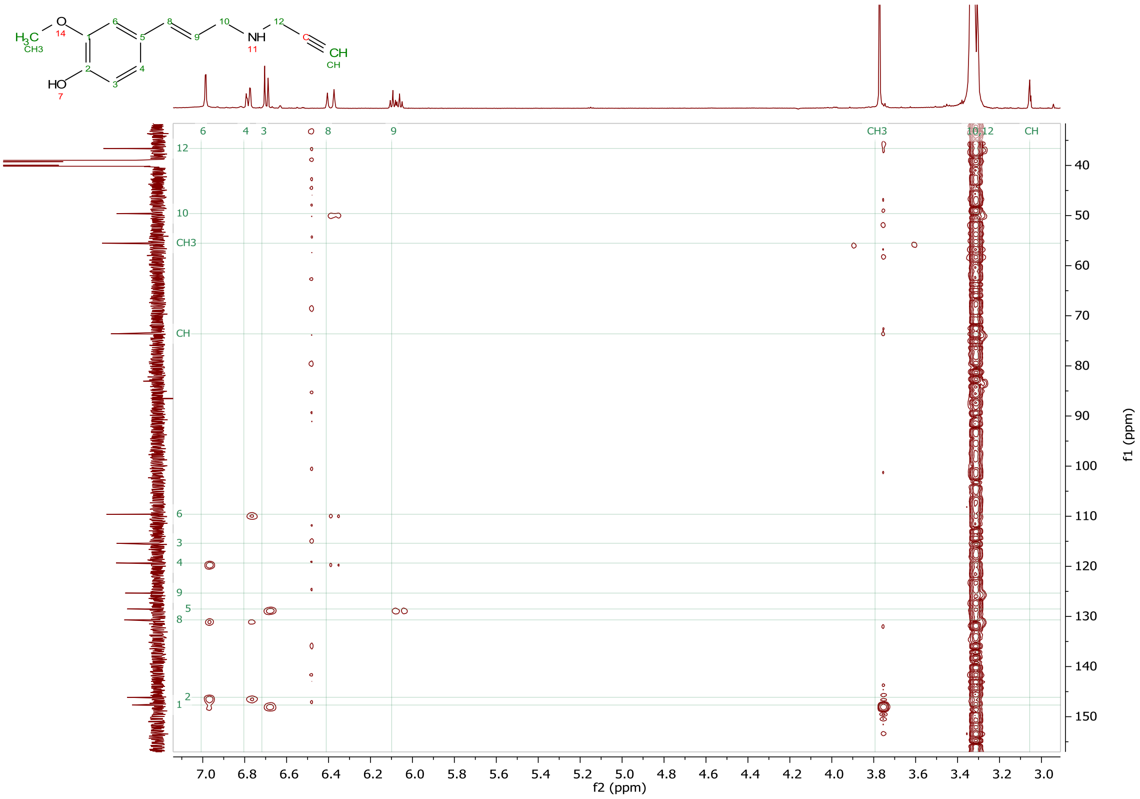

H3C
O
14
CH3
HO
7
NH
11
CH
f2 (ppm)
f1 (ppm)

Compound Table

Compound Label	RT	Mass	Abund	Formula	Tgt Mass	Diff (ppm)
Cpd 1: C13 H15 N O2	0.244	217.11067	19935	C13 H15 N O2	217.11028	1.82

Compound Label	RT	Algorithm	Mass
Cpd 1: C13 H15 N O2	0.244	Find By Formula	217.11067

MS Zoomed Spectrum

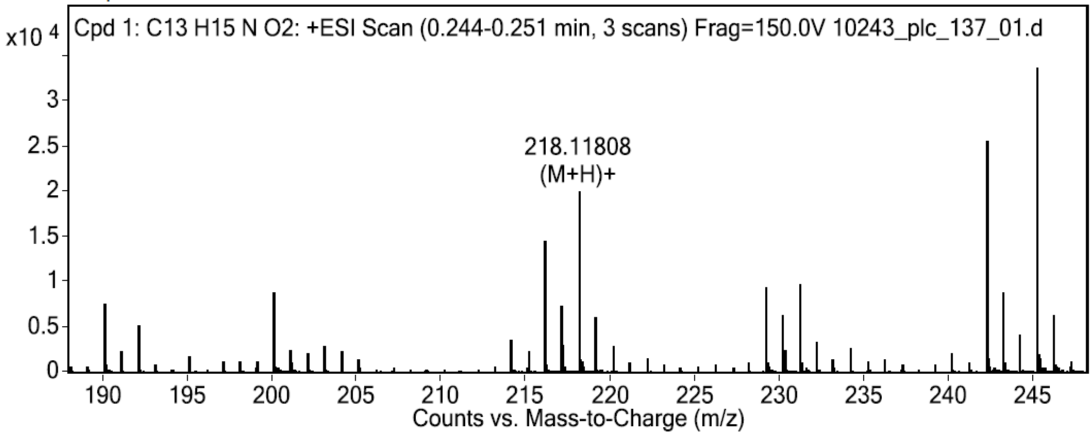

REFERENCES

(1) Molecular Operating Environment software (MOE), Integrated Computer-Aided Molecular Design Platform (https://www.chemcomp.com/Products.htm).

(2) The PyMOL Molecular Graphics System, Version 2.0 Schrödinger, LLC.

(3) Eurofins - CEREP, France (Https://Www.Eurofins.Fr/Contacts/Eurofins-Cerep-France/).

(4) Di, L.; Kerns, E. H.; Fan, K.; McConnell, O. J.; Carter, G. T. High Throughput Artificial Membrane Permeability Assay for Blood-Brain Barrier. *Eur. J. Med. Chem.* **2003**, *38* (3), 223–232.

(5) Mazanetz, M. P.; Marmon, R. J.; Reisser, C. B. T.; Morao, I. Drug Discovery Applications for KNIME: An Open Source Data Mining Platform. *Curr. Top. Med. Chem.* **2013**, *12* (18), 1965–1979. https://doi.org/10.2174/1568026611212180004.
